# *N*,*N*‐Bis(trifluoromethyl)aminoacetonitrile: A Versatile Building Block for the Introduction of the Bis(trifluoromethyl)amino Group

**DOI:** 10.1002/chem.202501550

**Published:** 2025-06-16

**Authors:** Kristina A. M. Maibom, Christoph Breitenstein, Sabine Lorenzen, Tanja Knuplez, Leon N. Schneider, Younes K. J. Bejaoui, Johannes Gierling, Krzysztof Radacki, Holger Braunschweig, Carl Deutsch, Min Shan, Thomas Fuchß, Michael Schulte, Nikolai V. Ignat'ev, Maik Finze

**Affiliations:** ^1^ Institute of Inorganic Chemistry Institute for Sustainable Chemistry & Catalysis with Boron (ICB) University of Würzburg Am Hubland 97074 Würzburg Germany; ^2^ Institute of Inorganic Chemistry and Structural Chemistry II University of Düsseldorf Universitätsstr. 1 40225 Düsseldorf Germany; ^3^ Merck Healthcare KGaA Frankfurter Str. 250 64293 Darmstadt Germany; ^4^ NBE‐Therapeutics AG Technology Park Basel Basel 4057 Switzerland; ^5^ Merck Life Science KGaA Frankfurter Str. 250 64293 Darmstadt Germany; ^6^ Consultant, Merck KGaA 64293 Darmstadt Germany

**Keywords:** amines, perfluoroalkyl groups, perfluoroalkyl nitrogen compounds, structure elucidation, trifluoromethyl groups

## Abstract

*N*,*N*‐Bis(trifluoromethyl)aminoacetonitrile (CF_3_)_2_NCH_2_CN (**1**) that is accessible via a simple single‐step protocol is established as a building block for the introduction of the (CF_3_)_2_N group. The two reaction sites of **1**, the methylene unit and the nitrile (cyano) group, provide access to different organic substance classes. This includes alkenes via Knoevenagel condensations, and *N*‐heterocycles and acid derivatives obtained by addition reactions to the nitrile unit. These new (CF_3_)_2_N derivatives are potential building blocks containing the bis(trifluoromethyl)amino group. Deprotonation of **1** at the methylene group highlights the electron‐withdrawing nature of the (CF_3_)_2_N group. The {(CF_3_)_2_NCHCN}^−^ anion (**1^−^**) is characterized by NMR spectroscopy and its reaction with [Ph_3_PAuCl] results in [Ph_3_PAuCH{N(CF_3_)_2_}(CN)] (**Au1**) and [(Ph_3_PAu)_2_C{N(CF_3_)_2_}(CN)] (**Au_2_1**). Crystal structure analysis of (CF_3_)_2_NCH_2_CN (**1**), **Au_2_1**, and almost all alkenes, *N*‐heterocycles, and acid derivatives with a (CF_3_)_2_N group combined with spectroscopic data and results from DFT calculations provide insight into properties of the highly fluorinated (CF_3_)_2_N substituent. Especially the ability of the amino nitrogen atom to participate in weak hydrogen bonding, albeit its low basicity, is of relevance.

## Graphical abstract

*N*,*N*‐Bis(trifluoromethyl)aminoacetonitrile (CF_3_)_2_NCH_2_CN, which is readily accessible, is established as a versatile starting compound for the synthesis of various types of (CF_3_)_2_N‐containing building blocks, that is, *N*‐heterocycles, alkenes, and acid derivatives such as *N,N*‐bis(trifluoromethyl)glycine. The degradation of (CF_3_)_2_N derivatives in strong acidic and basic media at elevated temperatures to result in nonpersistent compounds is highlighted.

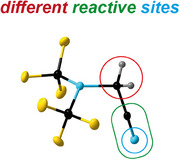

## 1 Introduction

Perfluorinated substituents are essential building blocks for pharmaceuticals,^[^ ^1^, ^2^, ^3^, ^4^, ^5^, ^6^ ^]^ agrochemicals,^[^ ^1^, ^7^, ^8^, ^9^ ^]^ and materials sciences,^[^ ^1^ ^]^ for example, for polymer^[^ ^10^, ^11^, ^12^, ^13^, ^14^ ^]^ and battery applications.^[^ ^15^, ^16^, ^17^, ^18^ ^]^ However, per‐ and polyfluoroalkyl substances (PFAS) are often persistent resulting in environmental concerns and human health risks.^[^ ^19^ ^]^ Hence, there is a demand for fluorinated groups that can undergo degradation while retaining the general advantageous properties of per‐ and polyfluorinated groups. The bis(trifluoromethyl)amino group N(CF_3_)_2_ is such a perfluorinated substituent that (i) provides sufficient stability but (ii) can degrade to nonpersistent products.

The bis(trifluoromethyl)amino group is the perfluorinated analogue of the widely used dimethylamino group (CH_3_)_2_N. Despite their structural similarity, these two groups possess entirely different electronic properties as evident, for example, from their Hammett constants (*σ*_p_) of + 0.50 or + 0.53 (N(CF_3_)_2_)^[^ ^20^, ^21^ ^]^ and −0.83 (N(CH_3_)_2_).^[^ ^22^ ^]^ The Hammett constant of N(CF_3_)_2_ is similar to *σ*_p_ of other well‐established electron‐withdrawing groups such as NO_2_ (+0.78), NHSO_2_CF_3_ (+0.39), CF_3_ (+0.54), SCF_3_ (+0.50), and SF_5_ (+0.68).^[^ ^1^, ^22^ ^]^ Due to the strong electron withdrawing nature of the CF_3_ substituents, *N*,*N*‐bis(trifluoromethyl)amine (CF_3_)_2_NH is a weak Brønsted acid, which is deprotonated by triethylamine.^[^ ^23^ ^]^ In contrast, dimethylamine (CH_3_)_2_NH is a strong organic base. Owing to its basic properties, *N*,*N*‐dimethylaniline C_6_H_5_N(CH_3_)_2_ forms salts with many organic and inorganic acids. In contrast, *N*,*N*‐bis(trifluoromethyl)aniline C_6_H_5_N(CF_3_)_2_ was reported to be nonbasic and not to form salts with acids by protonation.^[^ ^21^ ^]^

Despite its interesting and advantageous properties, the bis(trifluoromethyl)amino group almost vanished from the literature since the 1980s and only a few reports on N(CF_3_)_2_ compounds were published.^[^ ^24^ ^]^ The reason is the hardly accessible and potentially hazardous precursors and a general lack of N(CF_3_)_2_‐containing small building blocks that enable a straight forward and scalable introduction of the N(CF_3_)_2_ group into organic molecules. Most syntheses of bis(trifluoromethyl)amino compounds were based on perfluoro‐2‐azapropene CF_3_─N═CF_2_,^[^ ^24^, ^25^, ^26^ ^]^ which is not a commercial product anymore. Notably, handling of CF_3_─N═CF_2_ requires special equipment because it is a highly moisture sensitive gas (b.p. −33 °C) that releases toxic hydrogen fluoride (HF) upon hydrolysis. A key step of the synthesis of bis(trifluoromethyl)amino derivatives starting from CF_3_─N═CF_2_ is the conversion of perfluoro‐2‐azapropene into the bis(trifluoromethyl)amide anion {(CF_3_)_2_N}**^−^** with CsF or KF.^[^ ^27^, ^28^, ^29^, ^30^ ^]^ The {(CF_3_)_2_N}**^−^** anion is a weak nucleophile similar to the recently reported {(CF_3_)(CF_3_S)N}**^−^** anion.^[^ ^31^ ^]^ The bis(trifluoromethyl)amide anion has been used for the introduction of the bis(trifluoromethyl)amino group.^[^ ^24^, ^25^, ^26^ ^]^ However, the {(CF_3_)_2_N}**^−^** anion undergoes fast reaction with unreacted CF_3_─N═CF_2_ to yield (CF_3_)_2_N─CF═NCF_3_, which often results in low yields of the desired products.^[^ ^28^, ^32^, ^33^, ^34^, ^35^ ^]^

**Scheme 1:**
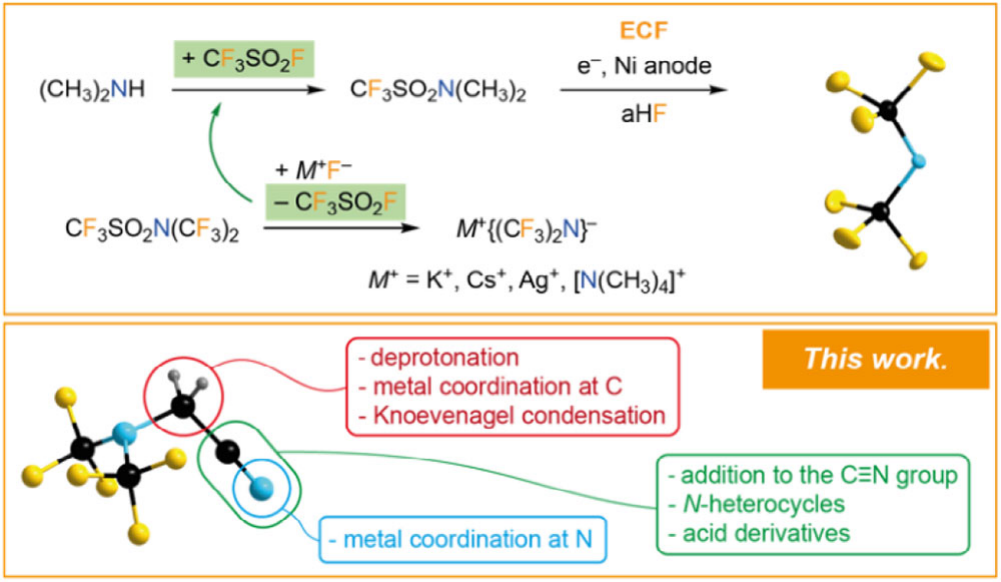
Efficient conversion of dimethylamine (CH_3_)_2_NH into {(CF_3_)_2_N}**^−^** salts via a three‐step protocol^[^ ^36^ ^]^ and structure of the {(CF_3_)_2_N}**^−^** anion in its [Ag(PPh_3_)_4_]^+^ salt^[^ ^44^ ^]^ (top), and an overview on the different reactive sites of (CF_3_)_2_NCH_2_CN (**1**, bottom).

Recently, we developed an atom efficient three‐step protocol for the conversion of dimethylamine (CH_3_)_2_NH into {(CF_3_)_2_N}**^−^** salts (Scheme 1). In the first step, dimethylamine (CH_3_)_2_NH and trifluoromethylsulfonyl fluoride CF_3_SO_2_F or alternatively triflic acid anhydride are reacted to give CF_3_SO_2_N(CH_3_)_2_. Electrochemical fluorination (ECF, Simons process)^[^ ^36^, ^37^ ^]^ of CF_3_SO_2_N(CH_3_)_2_ yields *N,N*‐bis(trifluoromethyl)trifluoromethanesulfonamide.^[^ ^36^, ^38^ ^]^ The perfluorinated amide CF_3_SO_2_N(CF_3_)_2_ and KF, CsF, AgF, or [N(CH_3_)_4_]F result in CF_3_SO_2_F, which can be reused as starting compound, and the corresponding {(CF_3_)_2_N}**^−^** salt.^[^ ^39^, ^40^, ^41^, ^42^ ^]^ Since perfluoro‐2‐azapropene is absent in the reaction mixture, formation of (CF_3_)_2_N─CF═NCF_3_ is suppressed and salts of the {(CF_3_)_2_N}**^−^** anion are generated in high selectivity and very good yield.^[^ ^39^, ^40^, ^41^, ^42^, ^43^ ^]^ The metal salts *M* ^+^{N(CF_3_)_2_}**^−^** (*M* = K, Cs, Ag) are formed almost quantitatively in solution and the tetramethylammonium salt [N(CH_3_)_4_]^+^{(CF_3_)_2_N}**^−^** is obtained in 90% yield as a storable white solid (*T*_mp_ = 120–125 °C).^[^ ^40^, ^43^ ^]^ More recently, stable and storable silver(I) and copper(I) complexes of the bis(trifluoromethyl)amino ligand have been obtained and used as (CF_3_)_2_N transfer reagents.^[^ ^44^ ^]^

Recently, some of us employed {(CF_3_)_2_N}**^−^** salts for the synthesis of aliphatic compounds with the (CF_3_)_2_N group via nucleophilic substitution.^[^ ^45^ ^]^ The nitrile (CF_3_)_2_NCH_2_CN (**1**) is an example for such aliphatic compounds that was obtained via a three‐step protocol for the first time. Here, we describe an improved single‐step synthesis of **1** and demonstrate the synthetic applicability of **1**. The different reactive sites of **1** were chemically addressed selectively, making **1** a versatile building block that provides access to different substance classes with the (CF_3_)_2_N group including *N*‐heterocycles, alkenes, and acid derivatives. General properties of the (CF_3_)_2_N substituent are discussed based on structural and spectroscopic properties aided by theoretical data.

## 2 Results and Discussion

### 2.1 Synthesis and Properties of (CF_3_)_2_NCH_2_CN (**1**)

**Scheme 2:**
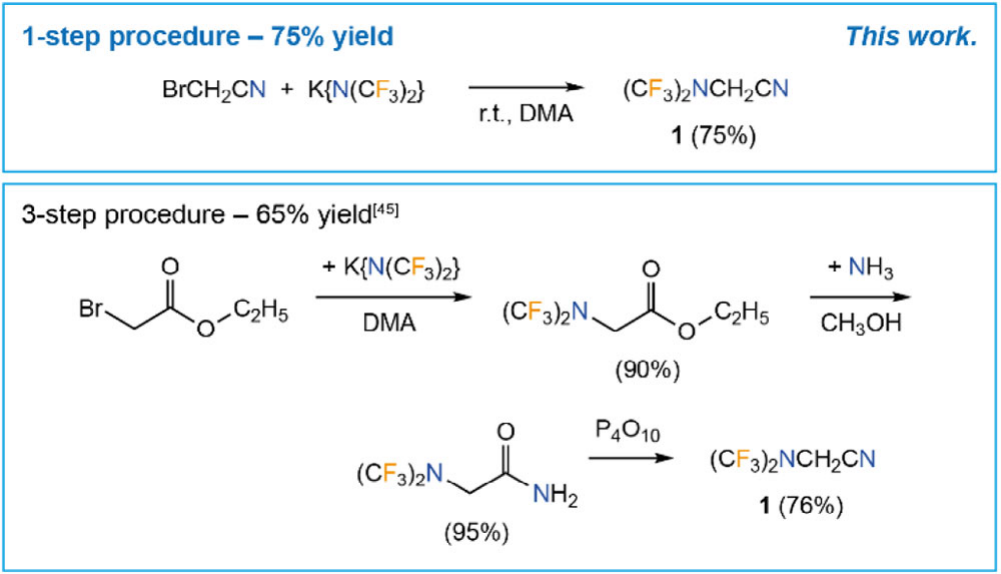
Single‐step synthesis of (CF_3_)_2_NCH_2_CN (**1**, top) and literature‐known three‐step synthesis of **1** (bottom).^[^ ^45^ ^]^

**Figure 1:**
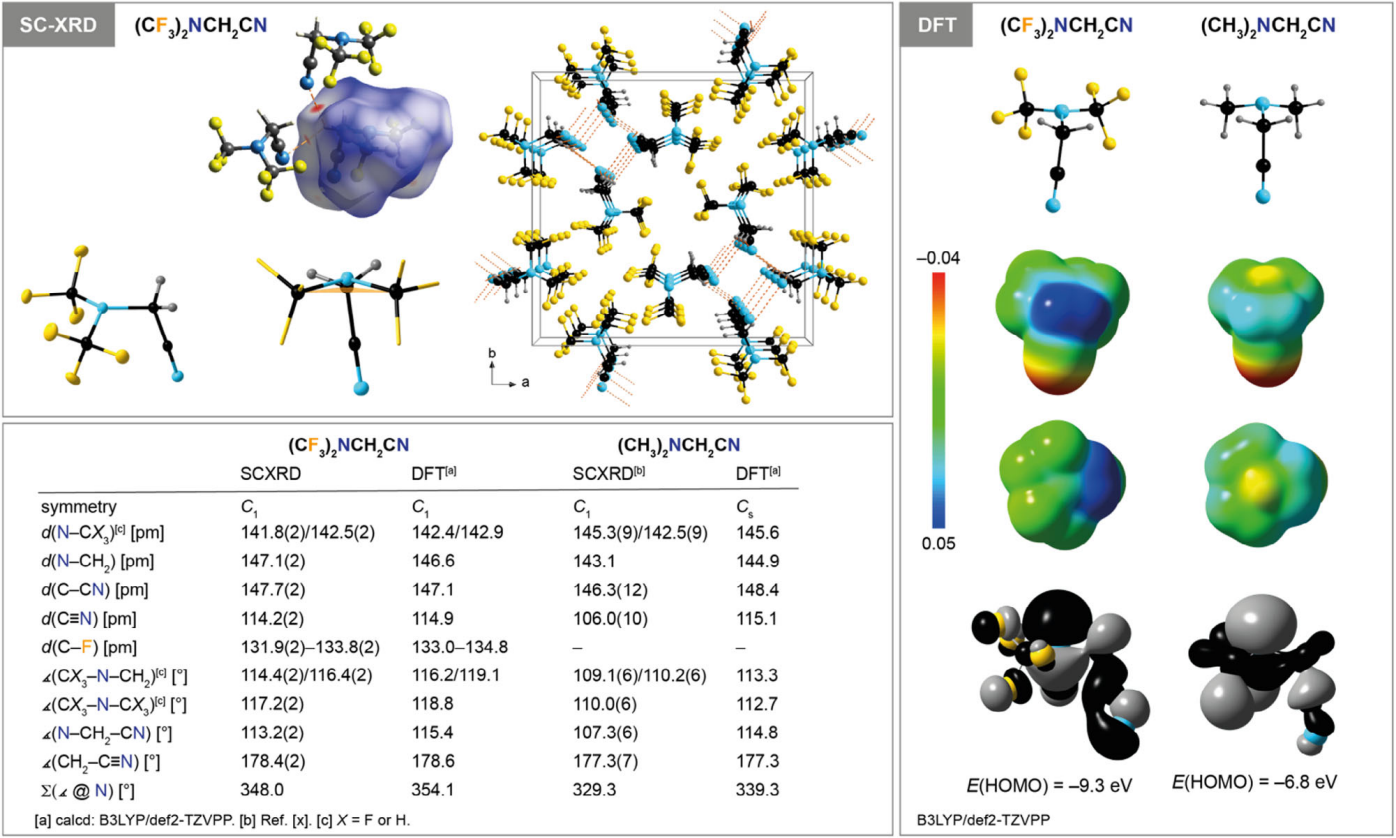
Molecular structure of (CF_3_)_2_NCH_2_CN (**1**; thermal ellipsoids set at 50% probability, H atoms are shown with arbitrary radii) highlighting the pyramidal arrangement around the amino N atom, the H‐bonding in the crystal (*d*_norm_ mapped onto the Hirshfeld surface^[^ ^54^, ^55^ ^]^), and a view along the c axis (atoms depicted with arbitrary radii;^[^ ^56^ ^]^ top, left), plots of the calculated geometries of **1** and (CH_3_)_2_NCH_2_CN, ESP plots^[^ ^57^ ^]^ (isovalue 0.0004), and HOMO plots^[^ ^57^ ^]^ (right), and a table with selected experimental and calculated bonding parameters of **1** and (CH_3_)_2_NCH_2_CN (bottom left).

*N,N*‐bis(trifluoromethyl)aminoacetonitrile (CF_3_)_2_NCH_2_CN (**1**) was obtained in a single‐step in a yield of 75% starting from bromoacetonitrile and K{N(CF_3_)_2_} in dimethylacetamide (DMA) (Scheme 2, top). The potassium salt K{N(CF_3_)_2_} was generated in situ from CF_3_SO_2_N(CF_3_)_2_^[^ ^36^, ^38^ ^]^ and potassium fluoride in DMA (Scheme 1).^[^ ^39^ ^]^ The single‐step protocol for the synthesis of **1** is an improvement compared to the three‐step literature method that gives **1** in a combined yield of 65% (Scheme 2, bottom).^[^ ^45^ ^]^

(CF_3_)_2_NCH_2_CN (**1**) is a liquid with a boiling point of 108–110 °C and a melting point of −63 °C (DSC, onset). A colorless single crystal of **1** suitable for single‐crystal X‐ray diffraction (SC‐XRD) was obtained by in‐situ low‐temperature crystallization. The solid‐state molecular structure of **1**, which crystallizes in the tetragonal space group *P*4_2_/*n* (*Z* = 8), is shown in Figure 1 and selected experimental bond distances and angles are compared to values derived from DFT calculations. The N─CF_3_ distances in **1** are significantly shorter than *d*(N─CH_3_) in its nonfluorinated analogue (CH_3_)_2_NCH_2_CN^[^ ^46^ ^]^ while for the N─CH_2_ distance the opposite trend is observed (Figure 1). Noteworthy, the tetrahedron formed by nitrogen and the carbon atoms of N(CF_3_)_2_ and the methylene unit is significantly compressed compared to the one of (CH_3_)_2_NCH_2_CN. The sum of the three C─N─C angles in **1** is 348.0° whereas for (CH_3_)_2_NCH_2_CN the sum of the C─N─C angles is 329.3°. These experimental values are in agreement to values derived from DFT calculations with 354.1 and 339.3° for **1** and (CH_3_)_2_NCH_2_CN, respectively (Figure 1). Similar observations were reported for related trifluoromethylamines and their nonfluorinated congeners earlier,^[^ ^47^, ^48^, ^49^, ^50^ ^]^ for example, for (CF_3_)*_n_* N(CH_3_)_3−_ *_n_* (*n* = 0–3).^[^ ^47^, ^48^, ^51^ ^]^ The different bond properties of methylamines with different degrees of fluorination, that is, *d*(N─C) and ∡(C─N─C), were attributed to a delicate interplay of electronic and steric factors,^[^ ^48^, ^52^, ^53^ ^]^ which include steric repulsion of the fluorine atoms of CF_3_ groups resulting in a flattening of the NC_3_ pyramid of the amine as observed for **1** compared to (CH_3_)_2_NCH_2_CN (Figure 1).

The nitrile group in (CF_3_)_2_NCH_2_CN (**1**) and (CH_3_)_2_NCH_2_CN adopts an antiperiplanar orientation with respect to the nitrogen lone pair (SC‐XRD and DFT, Figure 1), which is indicative for negative hyperconjugation (anomeric effect).^[^ ^52^, ^58^, ^59^ ^]^ This interpretation is supported by a relatively long C─CN bond in **1** and (CH_3_)_2_NCH_2_CN (Figure 1) compared to acetonitrile (*d*(C─CN) = 145.8, gas electron diffraction, GED)^[^ ^60^ ^]^ and 145.5 pm (B3LYP/def2‐TZVPP)). Furthermore, the shape of the HOMO of both nitriles **1** and (CH_3_)_2_NCH_2_CN supports this assumption as this orbital is antibonding with respect to the C─CN bond (Figure 1). The slightly longer calculated C─CN bond and shorter *d*(N─CH_2_CN) in (CH_3_)_2_NCH_2_CN compared to **1** and the perfect antiperiplanar orientation of the nitrile group are indicative for a more pronounced anomeric effect in the nonfluorinated molecule. This agrees to earlier findings, which showed that a high degree of fluorination in methylamines leads to less pronounced negative hyperconjugation.^[^ ^48^ ^]^ This effect was explained by the lower energy of the nitrogen lone pair with an increasing degree of fluorination,^[^ ^48^ ^]^ which in turn agrees to the relative HOMO energies of **1** and (CH_3_)_2_NCH_2_CN as both HOMOs possess major contributions from the nitrogen lone pair (Figure 1).

**Figure 2:**
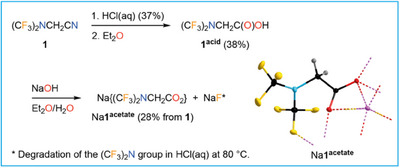
Hydrolysis of **1** to yield (CF_3_)_2_NCH_2_C(O)OH (**1^acid^**), conversion into Na{(CF_3_)_2_NCH_2_CO_2_} (Na**1^acetate^**}, and crystal structure of Na**1^acetate^** (ellipsoids are drawn at the 25% level except for the H atoms that are depicted with arbitrary radii).

The ESP plots in Figure 1 show that (i) the amino group of (CF_3_)_2_NCH_2_CN (**1**) is less basic than the one of (CH_3_)_2_NCH_2_CN and (ii) that the nitrile group is the most basic site in both molecules. This nicely agrees to the crystal packing of **1** that shows C─H···N bonding for the nitrile but not for the (CF_3_)_2_N group (Figure 1). The nitrile group in **1** is less basic than in (CH_3_)_2_NCH_2_CN, which can be concluded from the higher $\tilde{\nu }$(CN) of **1** (2262 cm**^−^** ^1^, Raman; 2365 cm**^−^** ^1^, DFT) compared to (CH_3_)_2_NCH_2_CN (2231 cm**^−^** ^1^ in ethyl acetate (IR/Raman)^[^ ^61^ ^]^ and 2338 cm**^−^** ^1^ (DFT)). Nevertheless, **1** was hydrolyzed in concentrated hydrochloric acid at 80 °C to result in the corresponding carboxylic acid (CF_3_)_2_NCH_2_C(O)OH in 38% yield (**1^acid^**; Figure 2). *N,N*‐bis(trifluoromethyl)glycine (**1^acid^**) was synthesized via an alternative route, earlier.^[^ ^45^ ^]^ It was neutralized with aqueous NaOH to give Na{(CF_3_)_2_NCH_2_CO_2_} (Na**1^acetate^**) in an overall yield of 28% with respect to nitrile **1** (Figure 2). Presumably, the low yield of **1^acid^** obtained from **1** in concentrated hydrochloric acid is due to the instability of the (CF_3_)_2_N group against strong acids at elevated temperature. This assumption is supported by the formation of HF during the reaction as confirmed by ^19^F NMR spectroscopy. Furthermore, a small amount of NaF was obtained during neutralization of **1^acid^** that was prepared by treatment of **1** with conc. aqueous HCl (Figure 2). Hydrolysis of **1** was investigated under basic conditions, that is, aqueous NaOH at 80 °C, as well. A mixture of two products, probably Na**1^acetate^** and (CF_3_)_2_NCH_2_C(O)NH_2_, was obtained. The formation of fluoride ions was proven by ^19^F NMR spectroscopy, indicating a certain instability of the (CF_3_)_2_N group against aqueous basic media at elevated temperature, as well. Hence, the (CF_3_)_2_N substituent can degrade to fluorides under basic or acidic aqueous conditions.

**Figure 3:**
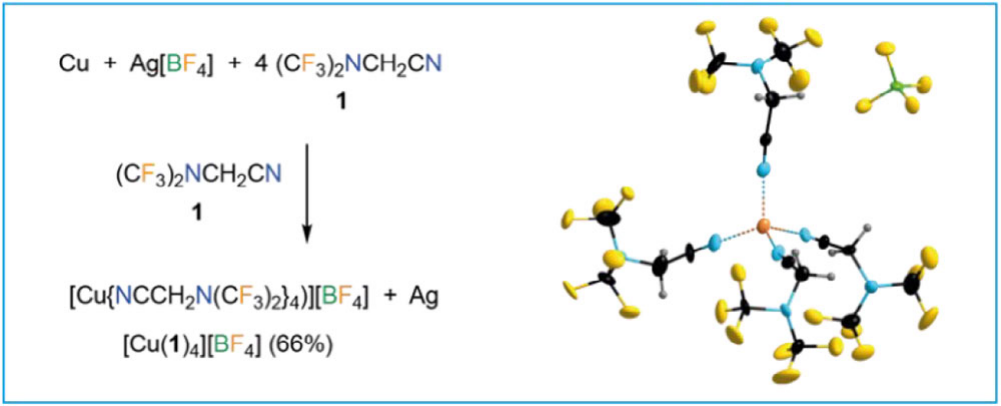
Synthesis and crystal structure of [Cu{NCCH_2_N(CF_3_)_2_}_4_][BF_4_] ([Cu(**1**)_4_][BF_4_]; ellipsoids are drawn at the 25% level except for the H atoms that are depicted with arbitrary radii). Selected distances (pm) and angles (°): Cu···N, 197.4(18)–199.6(17); N···Cu···N, 102.6(7)–118.9(7).

The nitrile group in **1** is capable to coordinate to a metal center as exemplified by the single‐crystal structure of [Cu(**1**)_4_][BF_4_], which was synthesized from elemental copper and silver(I) tetrafluoroborate in **1** as solvent (Figure 3). The Cu^I^ center reveals a distorted tetrahedral surrounding similar to the structure of [Cu(NCCH_3_)_4_][BF_4_].^[^ ^62^ ^]^ The presence of Cu^I^ in the bulk material was verified by ^63^Cu NMR spectroscopy (*δ*(^63^Cu) = 5.7 ppm; multiplet). Coordination of **1** to copper(I) is also evident from $\tilde{\nu }$(C≡N) that is observed at a higher wavenumber (2289 cm**^−^** ^1^) compared to free **1**. [Cu(**1**)_4_][BF_4_] starts to decompose at 110 °C (DSC, onset) by loss of ca. two of the four nitrile ligands **1** (STA measurement). At approximately 200 °C the two remaining (CF_3_)_2_NCH_2_CN ligands and BF_3_ are lost (Figure S17 in the Supporting Information).

### 2.2 Deprotonation of **1** and Coordination to Gold(I)

**Figure 4:**
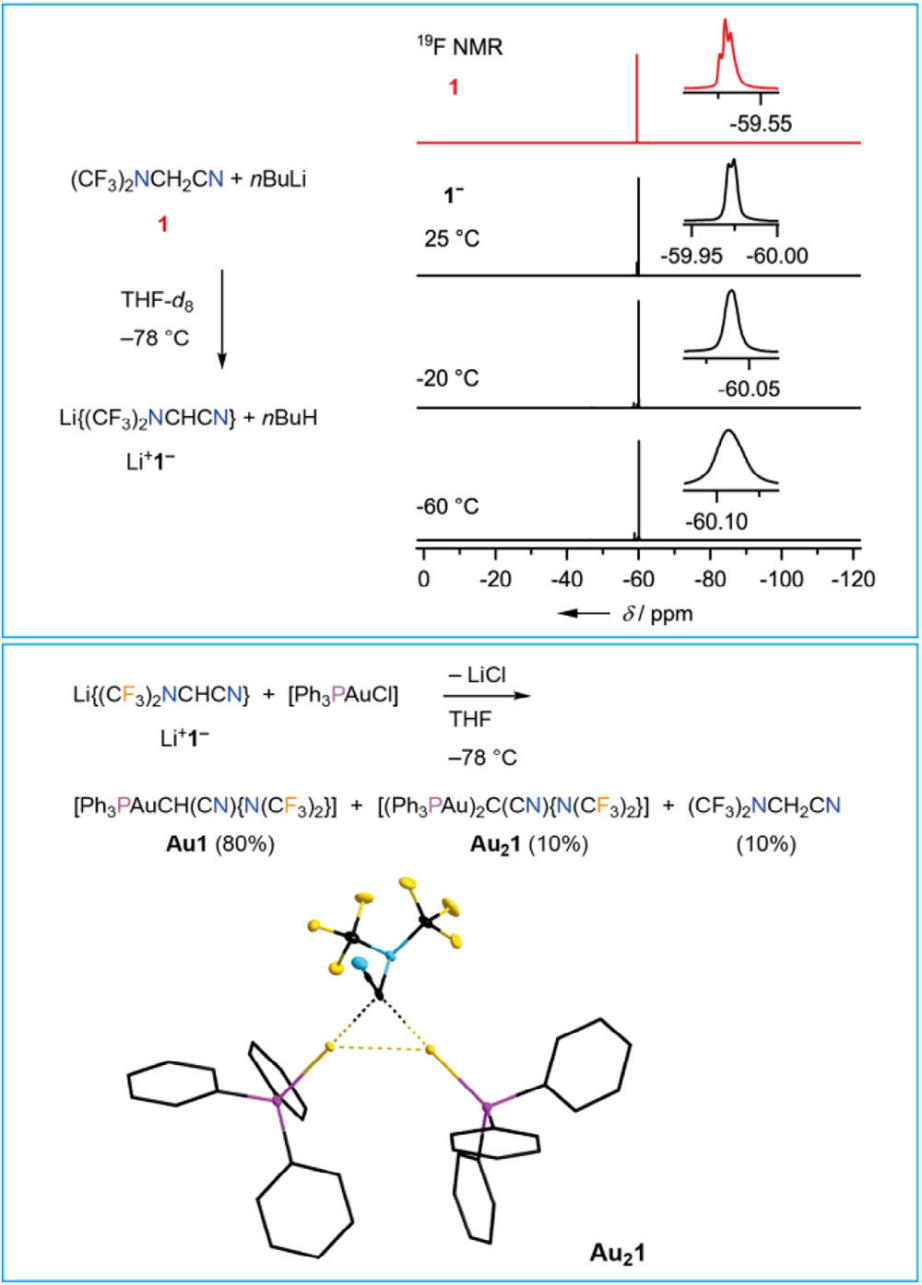
Deprotonation of **1** with *n*BuLi (left top) and VT ^19^F NMR spectra of **1** ^−^ (right top), and synthesis of gold(I) complexes of anion **1** ^−^ and crystal structure of **Au_2_1** (thermal ellipsoids set at 30% probability; H atoms are omitted for clarity; C atoms of the phenyl rings are depicted as stick models; bottom). Selected distances (pm) and angles (°): Au···C, 210.2(13) and 211.4(13); Au···Au, 287.54(7); Au···P, 225.9(3), and 226.8(3); C─CN, 143.1(19); C≡N, 114.7(19); C─N(CF_3_)_2_, 148.4(18); N─CF_3,_ 141(2), and 142.8(19); NC─C─N, 114.4(11); Au···C···Au, 86.0(5).

The aforementioned low basicity of both N atoms in **1**, which is due to the electron withdrawing CF_3_ groups, is in agreement to the positive Hammett constant of the N(CF_3_)_2_ group (+0.50 or + 0.53).^[^ ^20^, ^21^ ^]^ A consequence of the electron‐withdrawing effect of both, the bis(trifluoromethyl)amino and the cyano group is a relatively strong C─H acidity of the methylene unit. So, nitrile **1** can be considered as an analogue of malonic acid dinitrile. Deprotonation of the CH_2_ group was achieved with *n*BuLi in THF at −78 °C to give anion {(CF_3_)_2_NCHCN}^−^ (**1** ^−^) that was characterized by multinuclear NMR spectroscopy in THF‐*d*_8_ in 92% yield (Figure 4, see the Supporting Information). The ^19^F NMR signal of **1** ^−^ is split into a doublet with ^4^ *J*(^19^F,^1^H) of 1.3 Hz due to coupling to the single H atom while the signal of parent **1** reveals coupling to two H atoms with a ^4^ *J*(^19^F,^1^H) constant of 1.1 Hz (Figure 4). The ^1^H NMR signal of **1** ^−^ at 2.79 ppm is a septet due to ^4^ *J* coupling to the fluorine nuclei of the CF_3_ groups. Nitrile **1** was deprotonated with other strong bases, as well, for example, KHMDS (HMDS = hexamethyldisilazide; vide infra).

The lithiated bis(trifluoromethyl)aminonitrile decomposes at room temperature in solution in 30 minutes and even at −78 °C slow decomposition was observed. Thus, all attempts to crystallize Li^+^ **1** ^−^ at different temperatures failed. However, anion **1** ^−^ was trapped as ligand at gold(I), which unambiguously proved the formation of the deprotonated bis(trifluoromethyl)aminoacetonitrile. The reaction of a THF solution of Li^+^ **1** ^−^ with [PPh_3_AuCl] yielded [PPh_3_AuCH(CN){N(CF_3_)_2_}] (**Au1**) as major product (Figure 4). In addition, a minor amount of the dinuclear complex [(PPh_3_Au)_2_C(CN){N(CF_3_)_2_}] (**Au_2_1**) formed together with the parent nitrile **1** (see the Supporting Information for NMR spectroscopic characterization). Formation of **Au_2_1** and **1** is rationalized by deprotonation of **Au1** with **1** ^−^ followed by reaction with a second equivalent of [PPh_3_AuCl]. Removal of THF followed by redissolution in THF resulted in partial conversion of **Au1** into **Au_2_1** and **1**. Single crystals of **Au_2_1** were obtained that prove the coordination of two cationic gold(I) fragments to carbon (Figure 4). The Au···C distances in **Au_2_1** of 210.2(13) and 211.4(13) pm are similar to *d*(Au···C) of related dinuclear gold(I) complexes, that is, obtained from malonic acid dinitrile^[^ ^63^ ^]^ and related compounds, earlier.^[^ ^63^, ^64^, ^65^ ^]^ The Au···Au distance of 287.54(7) pm in **Au_2_1** is indicative for a weak aurophilic interaction.^[^ ^66^, ^67^ ^]^

### 2.3 Knoevenagel Condensation

**Figure 5:**
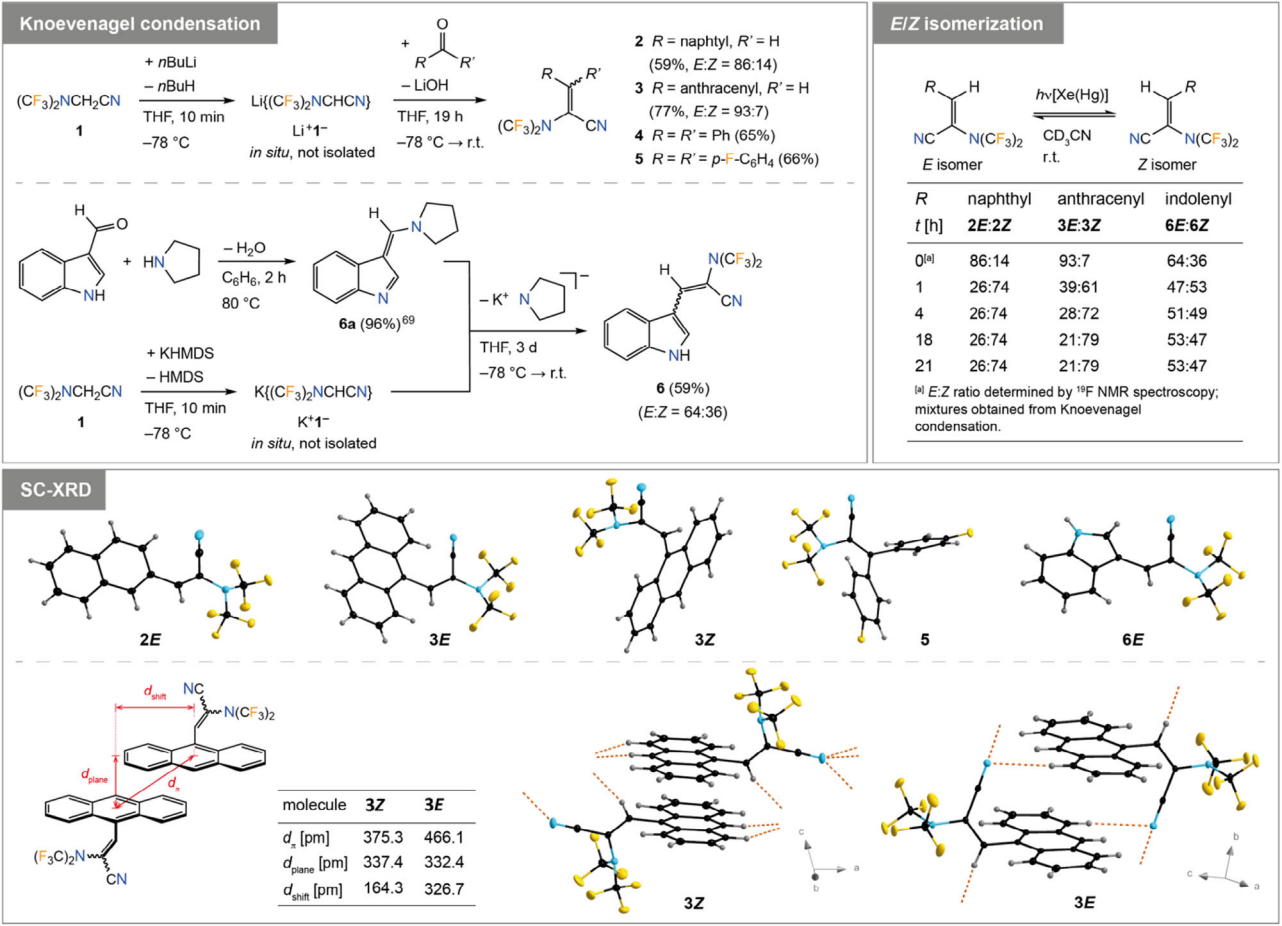
Synthesis of alkenes **2**–**6** via Knoevenagel reaction using deprotonated **1** (top, left), change in *E/Z* isomeric ratio of **2**, **3**, and **6** upon irradiation with a Xe(Hg) lamp (top, right), and crystal structures of **2*E***, **3*E***, **3*Z***, **5**, and **6*E*** including an assessment of the π‐π interaction in **3*E*** and **3*Z*** (bottom; ellipsoids are drawn at the 25% level except for the H atoms that are depicted with arbitrary radii).

Deprotonation of (CF_3_)_2_NCH_2_CN (**1**) was the prerequisite for its utilization in Knoevenagel reactions with aldehydes and ketones providing access to alkenes with the (CF_3_)_2_N group bonded to the double bond. Nitrile **1** was deprotonated with *n*‐butyllithium in THF at −78 °C and subsequently, selected aldehydes or ketones were added to yield alkenes **2**–**5** (Figure 5). Synthesis of the indole‐substituted alkene **6** was accomplished from **6a** ^[^ ^68^, ^69^ ^]^ and K^+^ **1** ^−^, which was prepared from **1** and KHMDS (Figure 5). Alkenes **2**–**6** were isolated in yields of 59─77% (Figure 5). Compounds **2**, **5**, and **6** are colorless solids while **3** is a yellow solid. All four compounds melt without decomposition (*T*_mp_ = 55 °C, **2**; 134 °C, **3**; 54 °C, **5**; 126 °C, **6**) and are thermally very robust (*T*_dec_ = 304 °C, **2**; 250 °C, **3**; 295 °C, **5**; 260 °C, **6**) as determined visually or by DSC (onset). Compound **4** is a colorless liquid (*T*_mp_ = −44 °C) that has a boiling point of 232 °C and starts to decompose at 355 °C (DSC onset).

Since **2**, **3**, and **6** have different substituents *R* and *R*’, mixtures of *E*/*Z* isomers were obtained as evident from two signal sets in the NMR spectra. Presumably, in all cases the *E* isomer is the major isomer since crystallization gave crystals of **2*E***, **3*E***, and **6*E*** (SC‐XRD). In case of the anthracenyl derivative **3**, both isomers were separated by flash chromatography, characterized by NMR spectroscopy, and isomer **3*Z*** was structurally characterized, thus providing unambiguous evidence for the assignment of the two isomers to the *E* and *Z* form of **3**, respectively. Isomerization of the unsymmetrically substituted alkenes **2**, **3**, and **6** was found to occur under irradiation with a Xe(Hg) lamp. In all cases the amount of the *Z* form increased and after some hours steady‐state conditions were reached (Figure 5). In contrast, heating of solutions of **2**, **3**, and **6** in acetonitrile to 80 °C in closed pressure vessels did not lead to any change of the isomeric composition. According to DFT calculations, the *E* isomer is thermodynamically marginally favored with a decrease of the relative stability of the *E* form in the order **3**, **2**, and **6** (Table S1 in the Supporting Information).

In the crystal structures obtained for **2*E***, **3*E***, **3*Z***, **5**, and **6*E***, the bis(trifluoromethyl)amino group is slightly pyramidalized similar to **1** and the lone pair at nitrogen is parallel to the N≡C─C═C plane (Figure 5). Hence, the CF_3_ groups are oriented above and below the N≡C─C═C unit. The naphthyl group in **2*E*** and the indolenyl group in **6*E*** are in plane with the N≡C─C═C unit while in **3*E***, **3*Z***, and **5** the aryl moieties are tilted out of the planar orientation and hence, electronic conjugation is limited due to steric reasons. For example, in **3*E*** and **3*Z*** the tilt angle is 80.17(2) and 69.33(3)°, respectively. Notably, in the related 2‐(anthracene‐9‐ylmethylene)malonnitrile^[^ ^69^ ^]^ the tilt angle is 49.68(5) and 50.75(8) ° for the crystallographically independent molecules, which reflects the smaller steric demand of the ─CH═C(CN)_2_ compared to the ─CH═C(CN){N(CF_3_)_2_} group in **3*E*** and **3*Z***.

The anthracenyl groups in **3*E*** and **3*Z*** reveal π‐π interactions and intermolecular hydrogen boding in the solid state (Figure 5). In **3*E*** the molecules form stacks along the *b* axis via π‐π interactions of neighboring anthracene units that are supported by hydrogen bonds. The alkene CH unit and one of the CH groups of the anthracene moiety act as donors and the nitrile nitrogen atom as acceptor in the bifurcated hydrogen bond motif (Figure 5). In the crystal of **3*Z***, two molecules are associated to dimers by π‐π interactions. These dimers are interconnected to infinite strains along the crystallographic *a* axis via trifurcated hydrogen bonding (Figure 5). Presumably, the π‐π bonding is stronger in **3*Z*** than in **3*E*** since the anthracenyl units are packed closer in **3*Z*** as evident from the *d*_π_ and *d*_shift_ values given in Figure 5.

**Figure 6:**
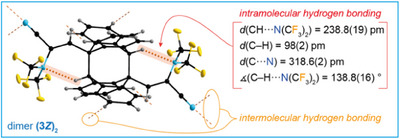
A molecule of **(3*Z*)_2_** in the crystal and selected details on the intra‐ and intermolecular hydrogen bonding (ellipsoids are drawn at the 25% level except for the H atoms that are depicted with arbitrary radii).

During photochemical *E*/*Z* isomerization of **3** (Figure 5), a small fraction of **3*Z*** underwent a head‐to‐tail [4 + 4] cycloaddition to give **(3*Z*)_2_** as evident from a SC‐XRD study (Figure 6). Analogous [4 + 4] cycloadditions under photochemical conditions are well known for anthracene and its derivatives.^[^ ^70^, ^71^, ^72^ ^]^ **(3*Z*)_2_** is located on a center of inversion and the molecules are interconnected by hydrogen bonds. Most interestingly, the crystal structure of **(3*Z*)_2_** provides unique evidence for the participation of the nitrogen atom of the (CF_3_)_2_N groups in hydrogen bonding. The tertiary C─H moieties of the central eight‐membered ring act as hydrogen bond donors and the N atoms of the (CF_3_)_2_N groups as acceptors (Figure 6).

**Figure 7:**
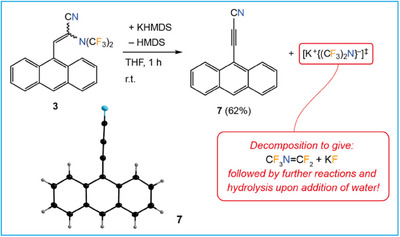
Synthesis of 9‐(cyanoethynyl)anthracene (**7**) by deprotonation of **3** followed by elimination of {(CF_3_)_2_N}^−^. Plane‐to‐plane parameters of the π‐π interaction (not depicted): *d*_π_ = 376.6 pm, *d*_plane_ = 343.3 pm, *d*_shift_ = 154.8 pm.

The anthracene derivative **3** shows that bis(trifluoromethyl)amines are nonpersistent compounds upon targeted design. Treatment of an isomeric mixture of **3** with KHMDS in THF gave 9‐(cyanoethynyl)anthracene^[^ ^73^ ^]^ (**7**) in 62% yield (Figure 7). Similarly, **3** was converted into **7** in methanol with potassium hydroxide. Notably, degradation was faster for **3*Z*** than for **3*E***. The {(CF_3_)_2_N}^−^ anion acted as leaving group and underwent follow‐up degradation under loss of F^−^ as documented in the literature.^[^ ^24^, ^28^, ^32^, ^33^, ^34^, ^35^ ^]^ Compound **7** was characterized by spectroscopic methods and by SC‐XRD (Figure 7). It forms stacks along the crystallographic *b* axis supported by π‐π interactions between neighboring anthracenyl moieties with plane‐to‐plane parameters similar to those of **3*Z*** (Figure 5).

A mixture of **6*E*** and **6*Z*** underwent degradation with NaOH in ethanol at 50–80 °C within 5–6 days similar to the reaction of **3** to yield **7**. The formation of fluoride ions was monitored by ^19^F NMR spectroscopy.

### 2.4 Addition Reactions to the Nitrile Group of (CF_3_)_2_NCH_2_CN (**1**)

**Figure 8:**
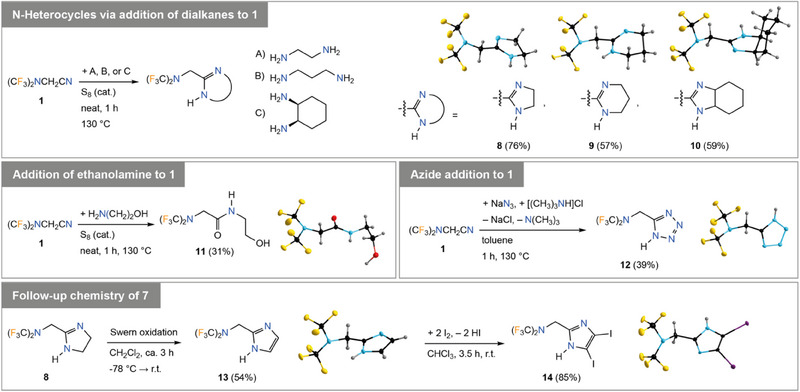
Addition reactions to the nitrile group of **1** providing (CF_3_)_2_NCH_2_‐substituted *N*‐heterocycles, that is, imidazoline **8**, tetrahydropyrimidine **9**, hexahydrobenzoimidazole **10** (top), and tetrazole **12** (middle, right), and acid amide **11** (middle, left), and two‐step functionalization of **8** resulting in imidazoles **13** and **14**. Crystal structures of **8**–**14** (ellipsoids are drawn at the 25% level except for the H atoms that are depicted with arbitrary radii).

The nitrile group of **1** provides a further option for functionalization and thus, for the introduction of the N(CF_3_)_2_ group into organic molecules. For example, the reaction of neat **1** with alkane diamines provides a convenient entry toward (CF_3_)_2_NCH_2_‐substituted heterocycles with two nitrogen atoms as exemplified by the preparation of imidazoline **8**, tetrahydropyrimidine **9**, and hexahydrobenzoimidazole **10** in yields of 57–76% (Figure 8). The reactions proceeded in one hour with elemental sulfur as catalyst. Similarly, the sulfur‐catalyzed addition of ethanolamine to **1** gave ethanolamide **11** in 31% yield (Figure 8). The synthesis of the (CF_3_)_2_NCH_2_‐substituted tetrazole **12** via a click‐type reaction of **1** with sodium azide in 39% yield is a further example for the synthetic versatility of **1** for the preparation of bis(trifluoromethyl)amines. In addition, imidazolyl derivative **8** was oxidized to give the corresponding imidazole **13** under Swern conditions in 54% yield and **13** was reacted with elemental iodine to give the diiodinated imidazole **14** in 85% yield (Figure 8). The colorless solids **8**–**13** melt at approximately 90 °C (DSC, onset). In case of **11** and **12** thermal decomposition starts at ca. 100 °C while **8**–**10** and **13** are stable up to more than 150 °C (DSC, onset). The diiodinated imidazole **14** melts under decomposition at 150 °C (DSC, onset).

All bis(trifluoromethyl)amino derivatives derived via functionalization of the nitrile group of **1** were characterized by SC‐XRD (Figure 8, see the Supporting Information). The (CF_3_)_2_NCH_2_ *R* moieties reveal slight pyramidalization at nitrogen, which agrees to the crystal structures of parent **1** (Figure 1), of the alkenes derived from Knoevenagel condensation (**2*E***, **3*E***, **3*Z***, **5**, and **6*E***, Figure 5), and of **(3*Z*)_2_** (Figure 6). Molecules of **8**–**14** are interconnected via hydrogen bonds in the solid state, respectively. The NH units and the OH group in **11** are involved in the H‐bonding. The imino N atoms of the heterocyclic rings act as H bond donors in the crystals of **8**–**10** and **12**–**14** and in case of **11** the O atoms of the amido unit and the hydroxy group are involved as H bond donors.

## Conclusions

The present study highlights the potential of bis(trifluoromethyl)aminonitrile (CF_3_)_2_NCH_2_CN (**1**) as versatile building block for the introduction of the elusive N(CF_3_)_2_ group into different types of organic molecules that are of potential interest for the design of pharmaceuticals, agrochemicals, and (electronic) materials, alike. The synthetic potential becomes obvious by a comparison of **1** to its close analogue malonic acid dinitrile, which is a widely applied key chemical. Thus, functionalization of **1** is easily achieved at its two reactive sites, (i) the acidic CH_2_ group and (ii) the nitrile (cyano) group. The functionalization at the methylene group was exemplified by the synthesis of the gold(I) complexes **Au1** and **Au_2_1** and Knoevenagel condensations yielding alkenes **2**–**6**. The utilization of the nitrile group was demonstrated by cyclization reactions to give *N*‐heterocycles **8**–**10** and **12**, the formation of the noncyclic acid amide **11**, (CF_3_)_2_NCH_2_C(O)OH (**1^acid^**) and Na{(CF_3_)_2_NCH_2_CO_2_} (Na**1^acetate^**). The general stability of the (CF_3_)_2_N group allows straight‐forward follow‐up reactions, for example the oxidation of imidazoline **8** to give the imidazole **13** that was further diiodinated to yield **14**. The degradation of the (CF_3_)_2_N group to nonpersistent compounds was exemplified by treatment of **1** with concentrated hydrochloric acid and aqueous NaOH at elevated temperature. Furthermore, a targeted molecular design leads to nonpersistent (CF_3_)_2_N compounds as demonstrated by the removal of the (CF_3_)_2_N group from alkenes **3** and **6**. These results highlight the potential of the N(CF_3_)_2_ group with respect to the replacement of long‐chain perfluorinated groups and thus, the importance of the availability of N(CF_3_)_2_‐containing small building blocks. The study also includes a new straight forward synthesis for **1** making it an even more appealing building block.

The combined study on nitrile **1** by SC‐XRD and DFT calculations together with a comparison to its nonfluorinated analogue (CH_3_)_2_NCH_2_CN provides insight into the effect of the strong electron‐withdrawing CF_3_ groups on the amino nitrogen center. E.g., the HOMO energy is lower and the amino N atom is significantly less basic in **1** as it is in its nonfluorinated analogue and **1** has a more planarized amino center than (CH_3_)_2_NCH_2_CN. Furthermore, the anomeric effect was found to be less relevant for the conformation of the CH_2_CN group in **1** than in (CH_3_)_2_NCH_2_CN. However, the crystal structure of **(3*Z*)_2_** shows that the amino N atom of a (CF_3_)_2_N*R* moiety can be involved in (intramolecular) hydrogen bonding, rendering the bis(trifluoromethyl)amino group an even more promising building block.

## Supporting Information

The authors have cited additional references within the Supporting Information.^[^ ^74^, ^75^, ^76^, ^77^, ^78^, ^79^, ^80^, ^81^, ^82^, ^83^, ^84^, ^85^, ^86^, ^87^, ^88^ ^]^ Deposition Numbers 2409465 (**1**), 2409480 ([Cu(**1**)_4_][BF_4_]), 2409481 (**Au_2_1**), 2443819 (Na**1^acid^**), 2409466 (**2*E***), 2409467 (**3*E***), 2409468 (**3*Z***), 2409469 (**(3*Z*)_2_**), 2409470 (**5**), 2409471 (**6*E***), 2409472 (**7**), 2409473 (**8**), 2409474 (**9**), 2409475 (**10**), 2409476 (**11**), 2409477 (**12**), 2409478 (**13**), and 2409479 (**14**) contain the supplementary crystallographic data for this paper. These data are provided free of charge by the joint Cambridge Crystallographic Data Centre and Fachinformationszentrum Karlsruhe Access Structures service.

## Conflict of Interests

The authors declare no conflict of interest.

## Supporting information



Supporting Information

## Data Availability

The data that support the findings of this study are available in the supplementary material of this article.
