# Supplementary material for: N,N‐Bis(trifluoromethyl)aminoacetonitrile: A Versatile Building Block for the Introduction of the Bis(trifluoromethyl)amino Group
Source: Chemistry. 2025 Jun 16;31(39):e202501550. doi: 10.1002/chem.202501550 (PMC12258662; doi:10.1002/chem.202501550)
Supplement: Supplementary file 1 — Supporting Information [file CHEM-31-e202501550-s001.docx]

**Supporting Information**

***N*,*N*-Bis(trifluoromethyl)aminoacetonitrile: A versatile building Block for the introduction of the bis(trifluoromethyl)amino group**

Kristina A. M. Maibom,^[a]^ Christoph Breitenstein,^[b]^ Sabine Lorenzen,^[a]^ Tanja Knuplez,^[a]^
Leon N. Schneider,^[a]^ Younes B. J. Bejaoui,^[a]^ Johannes Gierling,^[a]^ Krzysztof Radacki,^[a]^
Holger Braunschweig,^[a]^ Carl Deutsch,^[c,d]^ Min Shan,^[c]^ Thomas Fuchß,^[c]^ Michael Schulte,^[e]^ Nikolai V. Ignat'ev,^[a,f]^ and Maik Finze*^[a]^

^[a]^ Institute of Inorganic Chemistry, Institute for Sustainable Chemistry & Catalysis with Boron (ICB), Julius-Maximilians-Universität Würzburg, Am Hubland, 97074 Würzburg, Germany.
E-mail: [maik.finze@uni](mailto:maik.finze@uni)-wuerzburg.de

^[b]^ Institute of Inorganic Chemistry and Structural Chemistry II, Heinrich-Heine-Universität Düsseldorf, Universitätsstrasse 1, 40225 Düsseldorf, Germany.

^[c]^ Merck Healthcare KGaA, Frankfurter Strasse 250, 64293 Darmstadt, Germany.

^[d]^ NBE-Therapeutics AG, Technology Park Basel, 4057 Basel, Switzerland.

^[e]^ Purification R&D, Merck Life Science KGaA, Frankfurter Strasse 250, 64293 Darmstadt, Germany.

^[f]^ Consultant, Merck KGaA, 64293 Darmstadt, Germany.

**Table of Contents**

1. General Synthetic Aspects S3

2. Analytical Instruments and Details S3

3. Chemicals S4

4. Synthesis and Characterization S4

5. Quantum Chemical Calculations S71

6. Single Crystal X-ray Diffraction S72

7. References S76

**1. General Synthetic Aspects**

Reactions involving air-sensitive compounds were performed either in round-bottom flasks or in glass tubes equipped with valves with PTFE stems (Rettberg, Göttingen) under argon using standard Schlenk-line techniques.

**2. Analytical Instruments and Details**

^1^H, ^11^B, ^13^C, ^15^N, and ^19^F spectra were recorded at 298 K on a Bruker Avance 500 NMR spectrometer or a Bruker Avance Neo 400 spectrometer. NMR signals were referenced against (CH_3_)_4_Si (^1^H and ^13^C) with *Ξ*(^13^C) = 25.145020 MHz, BF_3_·OEt_2_ in CDCl_3_ with *Ξ*(^11^B) = 32.083974 MHz, MeNO_2_ with *Ξ*(^15^N) = 10.136767 MHz, and CFCl_3_ with *Ξ*(^19^F) = 94.094011 MHz.^[1]^ ^1^H and ^13^C chemical shifts were calibrated against the residual solvent signal and the solvent signal, respectively.^[2]^

Solid-state NMR spectra were recorded on a Bruker Avance Neo 400 NMR spectrometer at 293 K with 4 mm (o.d.) bottom-layer ZrO_2_ rotors containing ca. 100 mg of the sample. The spectra were conducted with MAS spinning rates between 8 and 15 kHz using a Bruker 4 mm WB CP/MAS probe. The following solid-state NMR experiments were performed: ^1^H DP/MAS, ^11^B{^1^H} RSHE/MAS, ^11^B{^19^F} RSHE/MAS, ^13^C{^1^H} CP/MAS, ^13^C{^19^F} CP/MAS, ^15^N CP/MAS, ^19^F ZG/MAS and ^63^Cu RSHE/MAS (DP = direct polarization, MAS = magic angle spinning, RSHE = rotor-synchronized Hahn-echo, CP = cross polarization). All chemical shifts were calibrated by setting the ^13^C low-field signal of ada­mantane *δ* = 38.48 ppm according to IUPAC recommend­da­tions^[1]^ with *Ξ*(^13^C) = 25.145020 MHz, *Ξ*(^11^B) = 32.083974 MHz, *Ξ*(^15^N) = 10.136767 MHz,
*Ξ*(^19^F) = 94.094011 MHz, and *Ξ*(^63^Cu) = 26.515473 **MHz**.

IR spectra were recorded at room temperature with a Bruker Alpha FT IR spectrometer with an apodized resolution of 4 cm^−1^ in the attenuated total reflection (ATR) mode in the region of 4000−400 cm^−1^ using a setup with a diamond crystal. Raman spectra were recorded at room temperature with a Bruker MultiRAM FT Raman spectrometer with an apodized resolution of 4 cm^−1^ using the 1064 nm line of a Nd/YAG laser for excitation of the crystalline samples contained in melting point capillaries.

HRMS ESI and ASAP spectra were recorded using an Exactive Plus mass spectrometer equipped with an Orbitrap mass analyser (Thermo Scientific) and an ESI source (3.5 kV spray voltage) or an APCI probe (4.0 µA discharge current) (HRMS = high resolution mass spectrometry, ESI = electrospray ionization, ASAP = atmospheric pressure solids analysis probe, APCI = atmospheric pressure chemical ionization).

Elemental analyses (C, H, N) were performed with an Elementar Varion Micro Cube instrument.

Thermal analyses were performed with a DSC 204 F1 Phoenix (Netzsch) in the temperature range of –150 to 500 °C with a heating rate of 10 K min^–1^ and DTA measurements were performed with a STA 449 F3 Perseus (Netzsch), connected to a Alpha FTIR spectrometer (Bruker) for the analysis of the gaseous decomposition products, in the temperature range of 30 to 500 °C with a heating rate of 10 K min^–1^.

**3. Chemicals**

All standard chemicals were obtained from commercial sources. Solvents were dried according to standard protocols and stored in flasks equipped with valves with PTFE stems (Rettberg, Göttingen) under an argon atmosphere. Potassium fluoride was obtained from ABCR. It was finely grounded and dried under vacuum at 150 °C for several days. CF_3_SO_2_N(CF_3_)_2_ was synthesized as described, earlier.^[3-4]^ 3-(1-pyrrolidinylmethylene)-*3H*-indole (**6a**)^[5]^ was prepared according to a literature procedure.^[5]^

**4. Synthesis and Characterization**

***N*,*N*-Bis(trifluoromethyl)aminoacetonitrile (1)**

(CF_3_)_2_NCH_2_CN

*N,N*-Bis(trifluoromethyl)trifluoromethanesulfonamide CF_3_SO_2_N(CF_3_)_2_ (12.7 g, 44.6 mmol) was added to a suspension of potassium fluoride (2.40 g, 41.3 mmol) in dimethylacetamide (DMA, 20 mL) at 0 °C. The reaction mixture was slowly heated to room temperature (4 h). After removal of the gaseous by-product trifluoromethanesulfonyl fluoride CF_3_SO_2_F under reduced pressure (5 · 10^−2^ mbar), 2-bromoacetonitrile (4.65 g, 38.8 mmol) was added and the reaction mixture was stirred for further 17 h. Ice water (30 mL) was added to the reaction mixture, the organic phase was separated, washed with ice water (2 x 20 mL) and dried with magnesium sulfate. *N,N*-bis(trifluoromethyl)aminoacetonitrile **1** was isolated by condensation under reduced pressure as a colorless liquid. Yield: 5.63 g (29.3 mmol, 75% calculated for 2-bromoacetonitrile).

^1^H NMR (500 MHz, CD_3_CN): *δ* = 4.42 ppm (sept, 2H, ^4^*J*_F,H_ = 1.2 Hz, C*H*_2_).

^13^C{^1^H} NMR (126 MHz, CD_3_CN): *δ* = 121.0 (q, br, 2C, ^1^*J*_F,C_ = 262 Hz, *C*F_3_), 115.4 (s, 1C, *C*≡N), 32.7 ppm (sept, 1C, ^3^*J*_F,C_ = 2.3 Hz, *C*H_2_).

^13^C{^19^F} NMR (126 MHz, CD_3_CN): *δ* = 121.0 (t, 2C, ^3^*J*_C,H_ = 4.4 Hz, *C*F_3_), 115.4 (t, 1C, ^2^*J*_C,H_ = 7.5 Hz, *C*≡N), 32.7 ppm (t, 1C, ^1^*J*_C,H_ = 153 Hz, *C*H_2_).

^15^N NMR (^1^H-^15^N HMBC, 51 MHz, CD_3_CN): *δ* = −125.4 ppm (s, 1N, C*N*).

^15^N NMR (^19^F-^15^N HMBC, 51 MHz, CD_3_CN): *δ* = −300.3 ppm (s, 1N, *N*(CF_3_)_2_).

^19^F NMR (471 MHz, CD_3_CN): *δ* = 59.1 ppm (t, 6F, ^4^*J*_F,H_ = 1.2 Hz, C*F*_3_).

^19^F{^1^H} NMR (377 MHz, CD_3_CN): *δ* = 59.1 ppm (s, 6F, C*F*_3_).

Raman: $\tilde{\nu}$ = 2262 (s, C≡N) cm^−1^.

Elemental analysis: calculated (%) for C_4_H_2_F_6_N_2_, C 25.01, H 1.05, N 14.59; found, C 24.74, H 1.05, N 14.60.


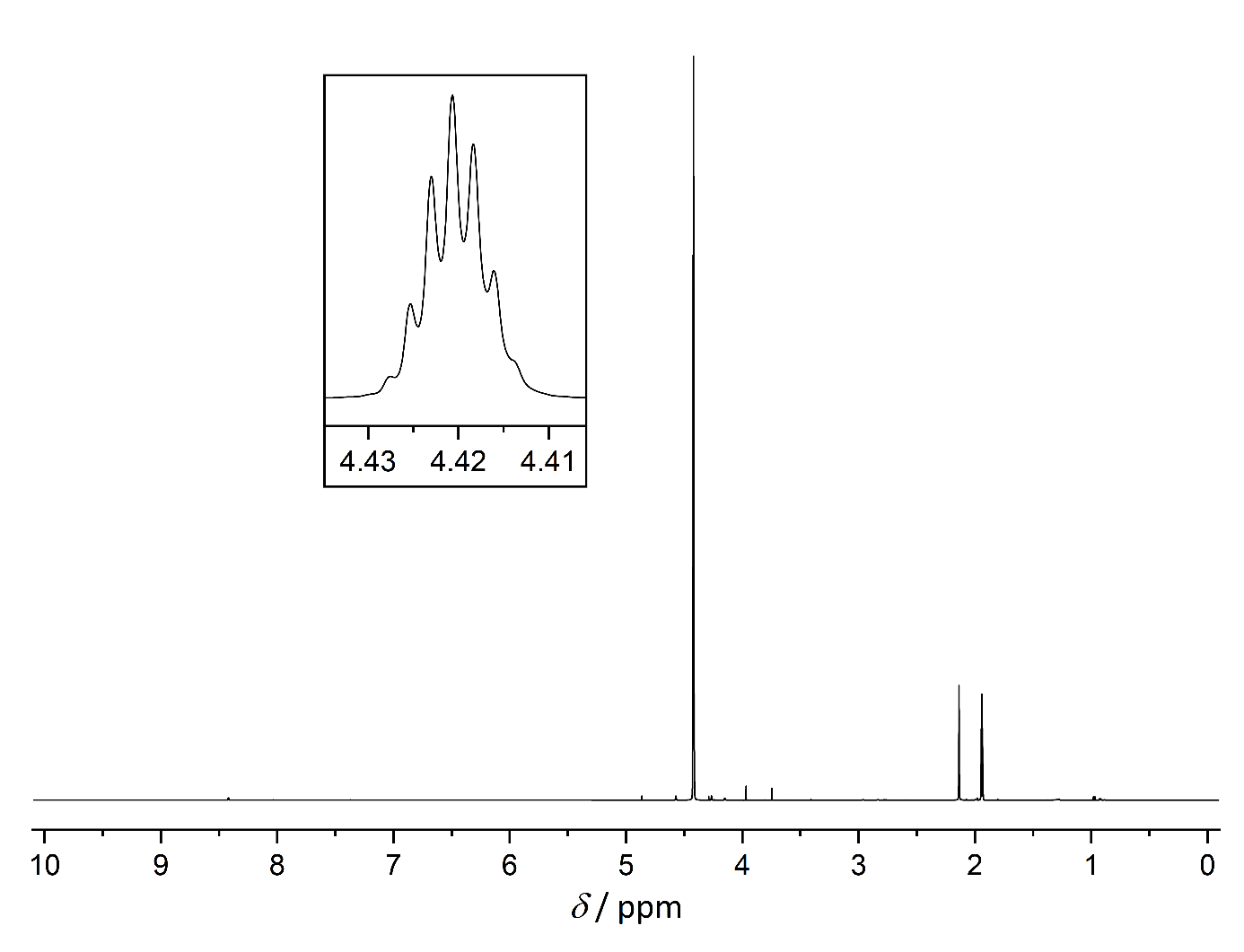


Fig. S1. ^1^H NMR spectrum of (CF_3_)_2_NCH_2_CN (**1**).


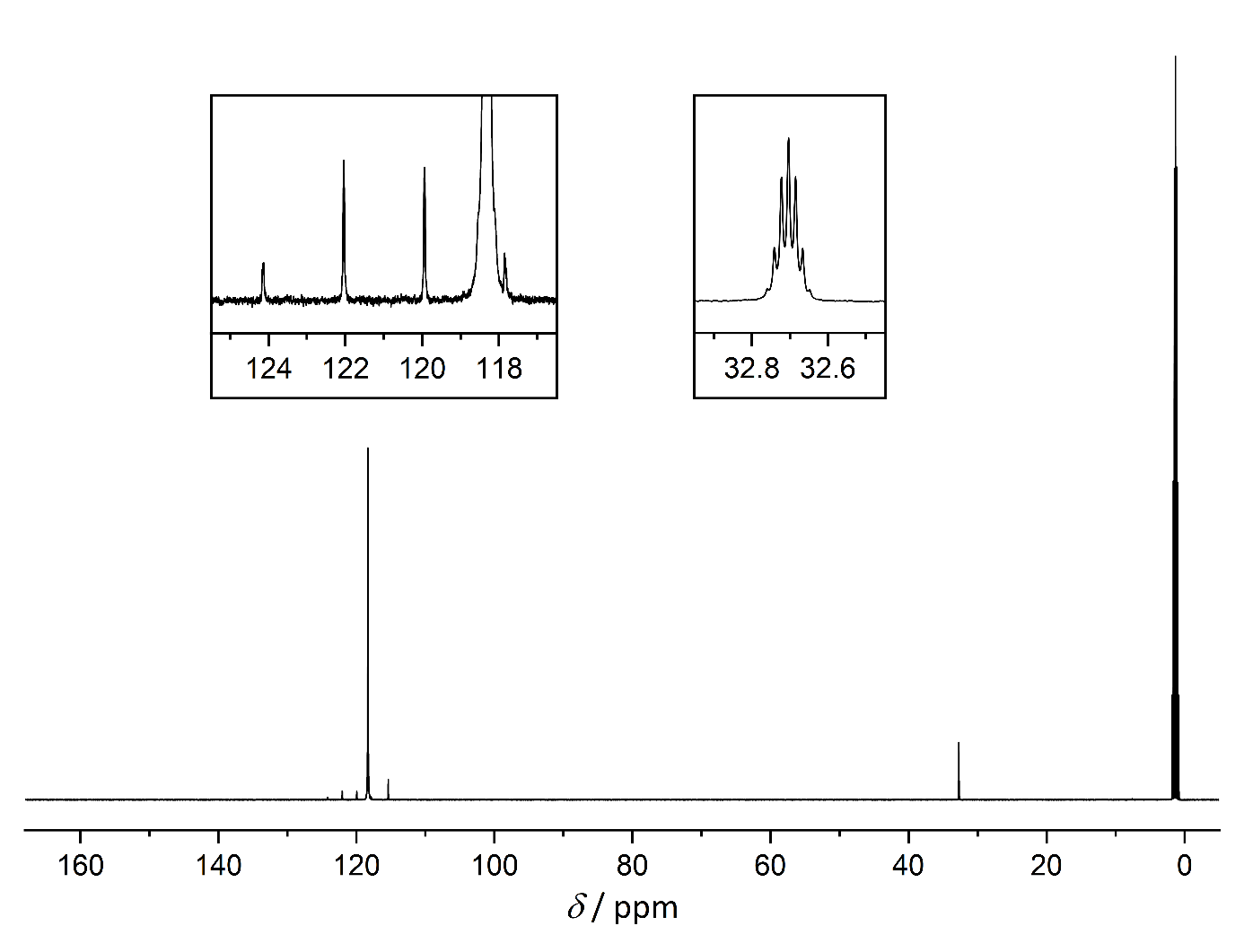


Fig. S2. ^13^C{^1^H} NMR spectrum of (CF_3_)_2_NCH_2_CN (**1**).


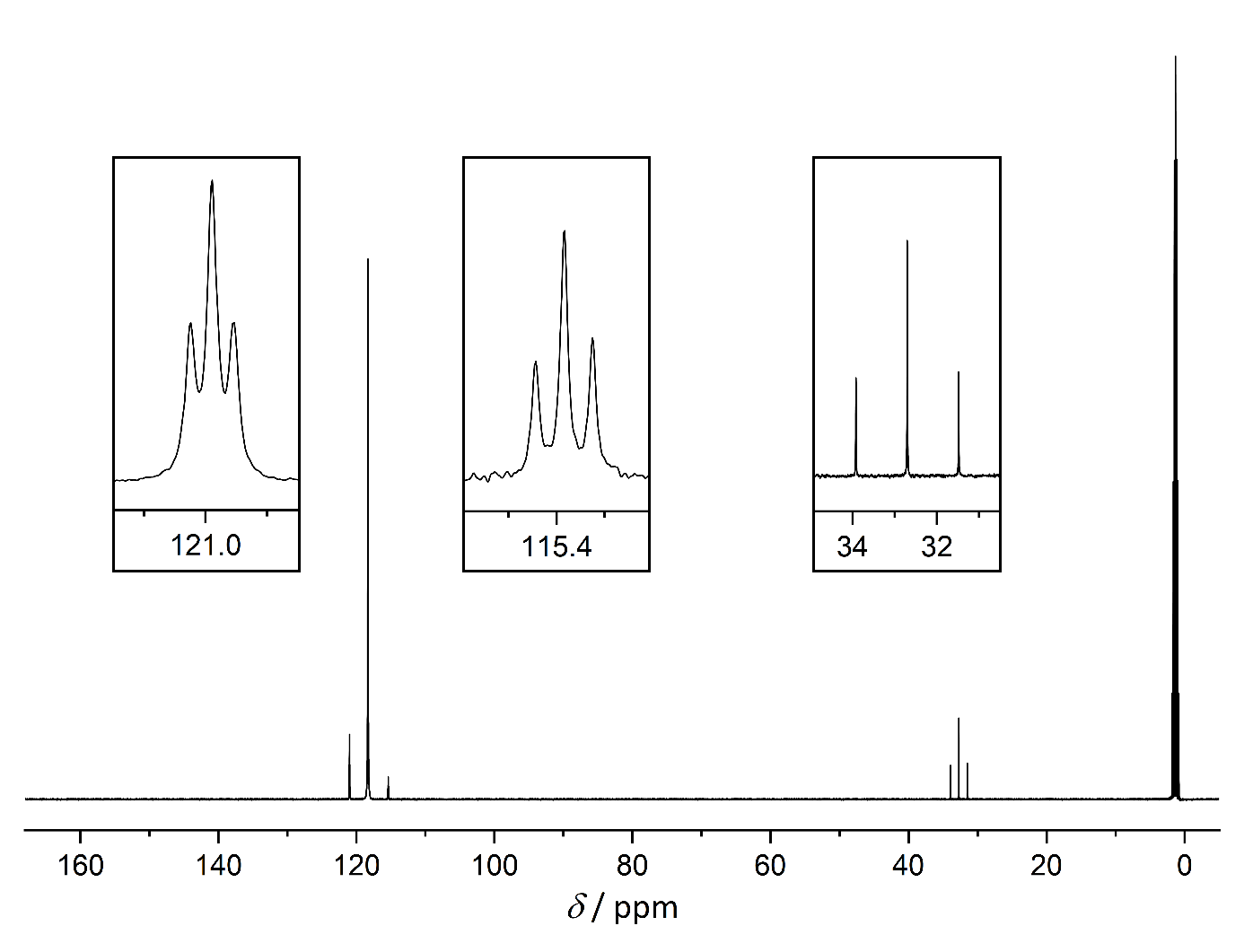


Fig. S3. ^13^C{^19^F} NMR spectrum of (CF_3_)_2_NCH_2_CN (**1**).


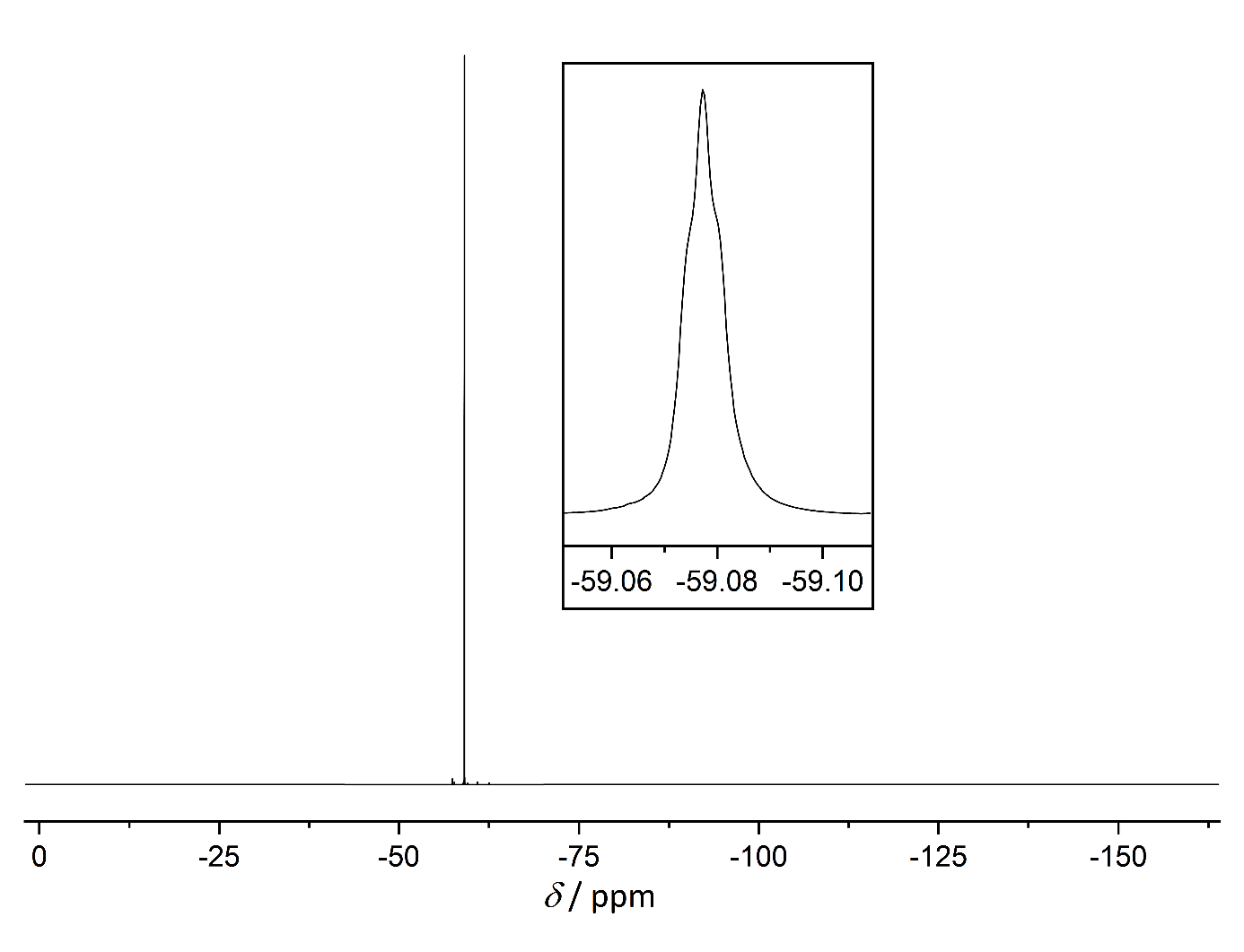


Fig. S4. ^19^F NMR spectrum of (CF_3_)_2_NCH_2_CN (**1**).


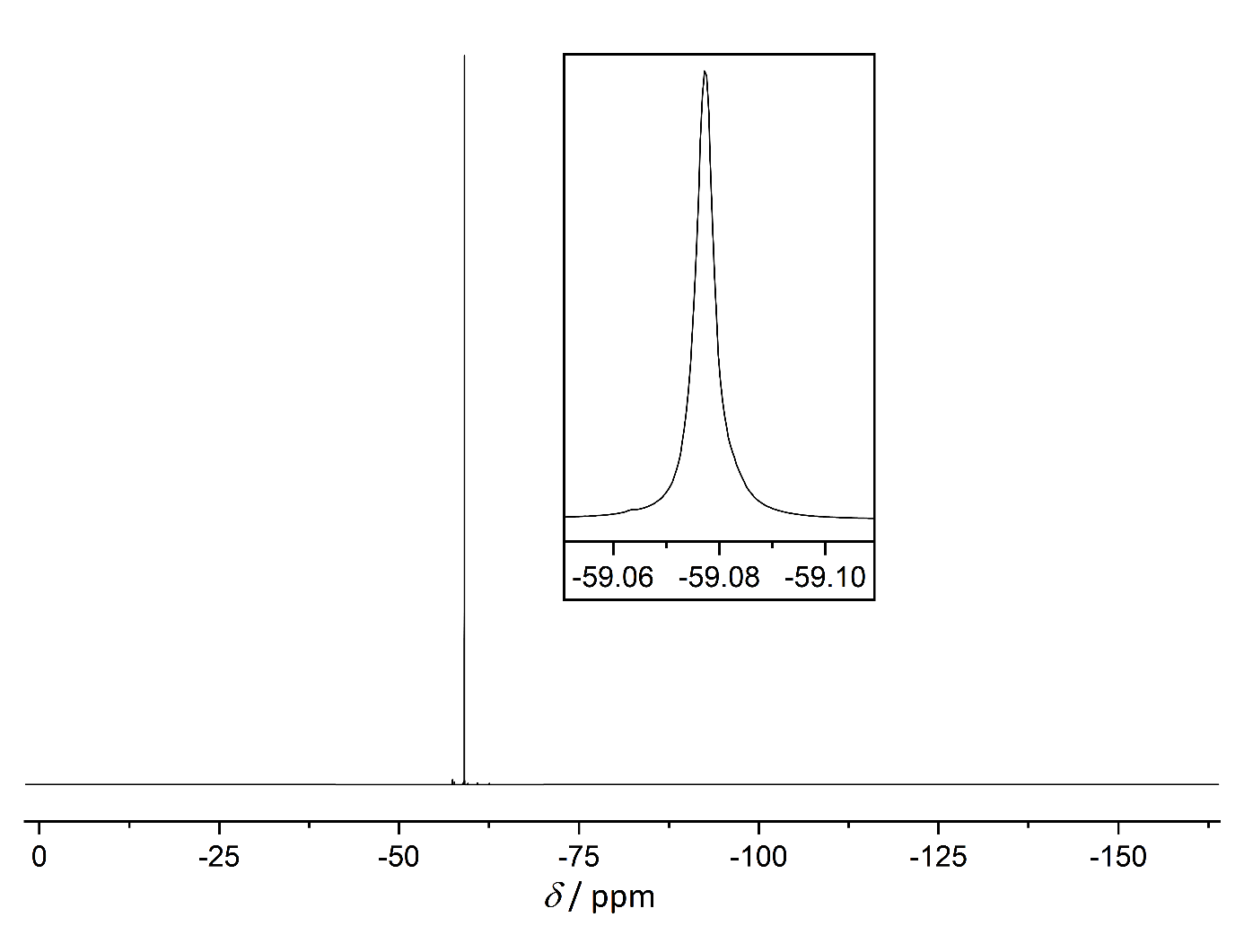


Fig. S5. ^19^F{^1^H} NMR spectrum of (CF_3_)_2_NCH_2_CN (**1**).


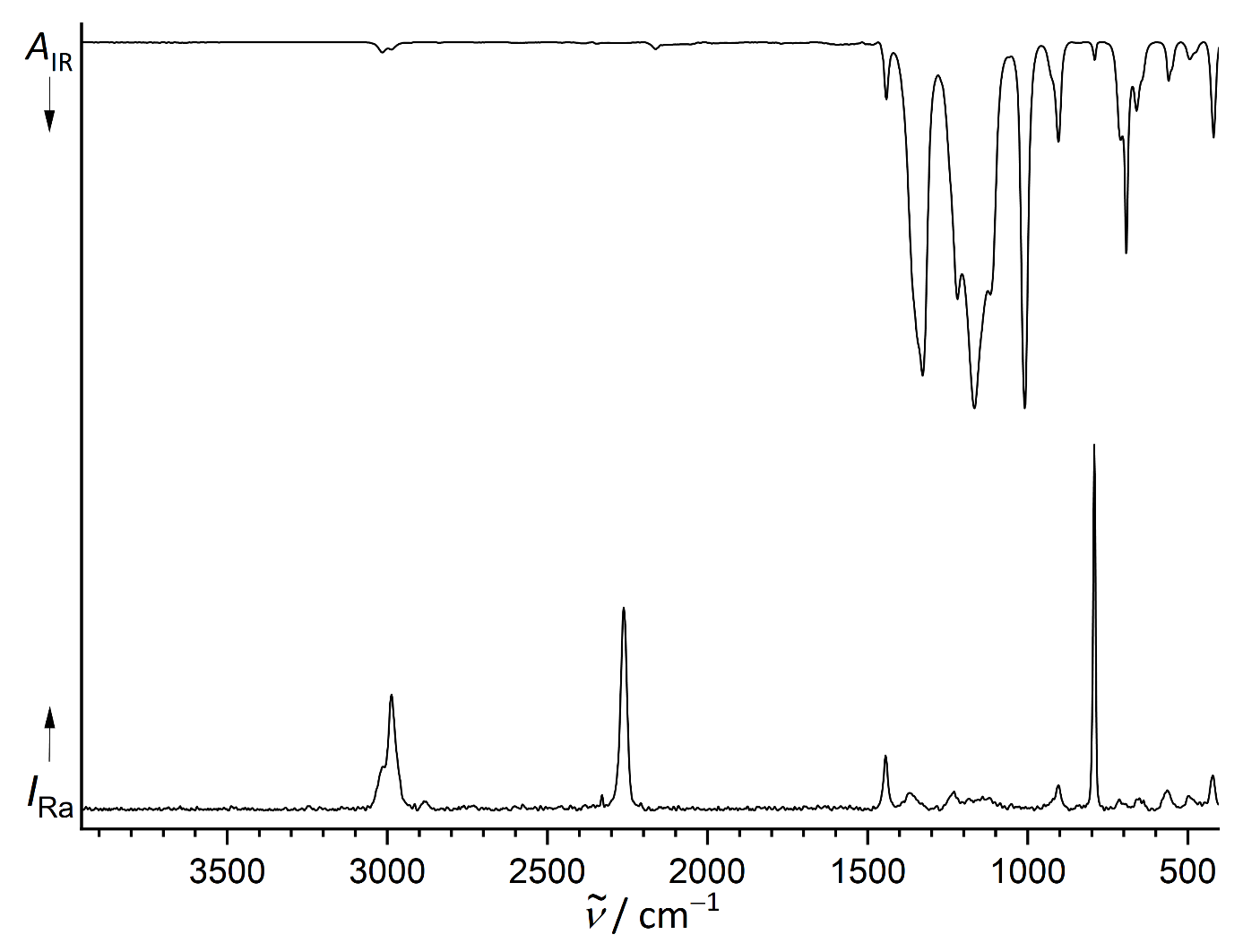


Fig. S6. IR (top) and Raman spectrum (bottom) of (CF_3_)_2_NCH_2_CN (**1**).


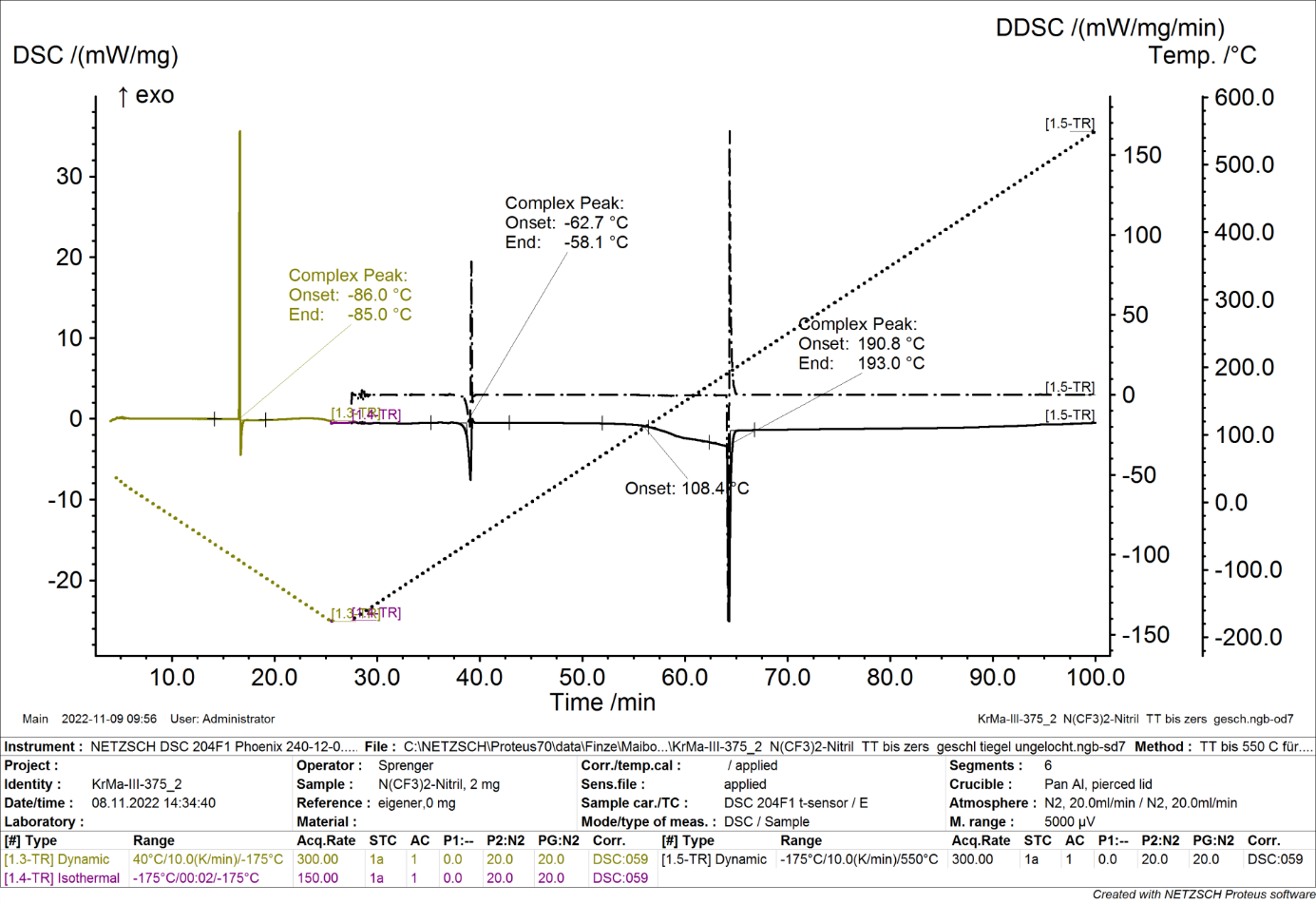


Fig. S7. DSC curve of (CF_3_)_2_NCH_2_CN (**1**).

**Copper(I)-tetrakis{*N*,*N*-bis(trifluoromethyl)aminoacetonitrile} tetrafluoroborate ([Cu(1)_4_][BF_4_])**

[Cu{NCCH_2_N(CF_3_)_2_}_4_)][BF_4_]

Silver tetrafluoroborate (118 mg, 606 µmol) and copper powder (38 mg, 598 µmol) were added to *N*,*N*-bis(trifluoromethyl)aminoacetonitrile (**1**) (2.83 g, 14.7 mmol). The copper powder and the Ag[BF_4_] reacted and a greyish solid formed that was removed by filtration after 30 min. Addition of diethyl ether (10 mL) resulted in a white precipitate that was filtered off and dried under reduced pressure. Yield: 363 mg (395 µmol, 66% calculated for elemental copper).

^1^H DP/MAS NMR (400 MHz, *v*_rot_= 14.8 kHz): *δ* = 5.13 ppm (s, 2H, C*H*_2_).

^13^C{^1^H} CP/MAS NMR (101 MHz, *v*_rot_= 14.0 kHz): *δ* = 120.2 (m, 2C, *C*F_3_), 113.0 (s, 1C, *C*≡N), 31.8 ppm (s, 1C, *C*H_2_).

^13^C{^19^F} CP/MAS NMR (101 MHz, *v*_rot_= 10.0 kHz): *δ* = 120.2 ppm (s, 2C, *C*F_3_).

^11^B{^1^H} RSHE/MAS (128 MHz, *v*_rot_= 14.0 kHz): *δ* = −0.5 ppm (m, 1B, *B*F_4_).

^11^B{^19^F} RSHE/MAS (128 MHz, *v*_rot_= 14.8 kHz): *δ* = −0.5 ppm (s, 1B, *B*F_4_).

^15^N CP/MAS NMR (41 MHz, *v*_rot_= 8.0 kHz): *δ* = −296.1 ppm (s, 1N, *N*(CF_3_)_2_). The signal of the cyano group was not observed.

^19^F ZG/MAS NMR (376 MHz, *v*_rot_= 14.8 kHz): *δ* = −59.8 (s, br, 6F, C*F*_3_), −150.3 ppm (s, 4F, B*F*_4_).

^63^Cu RSHE/MAS (106 MHz, *v*_rot_= 14.8 kHz): *δ* = 5.7 ppm (m, 1Cu, Cu).

Raman: $\tilde{\nu}$ = 2289 (s, C≡N) cm^−1^.

Elemental analysis: calculated (%) for C_16_H_8_BCuF_28_N_8_, C 20.92, H 0.88, N 12.20; found, C 21.29, H 0.64, N 11.89.


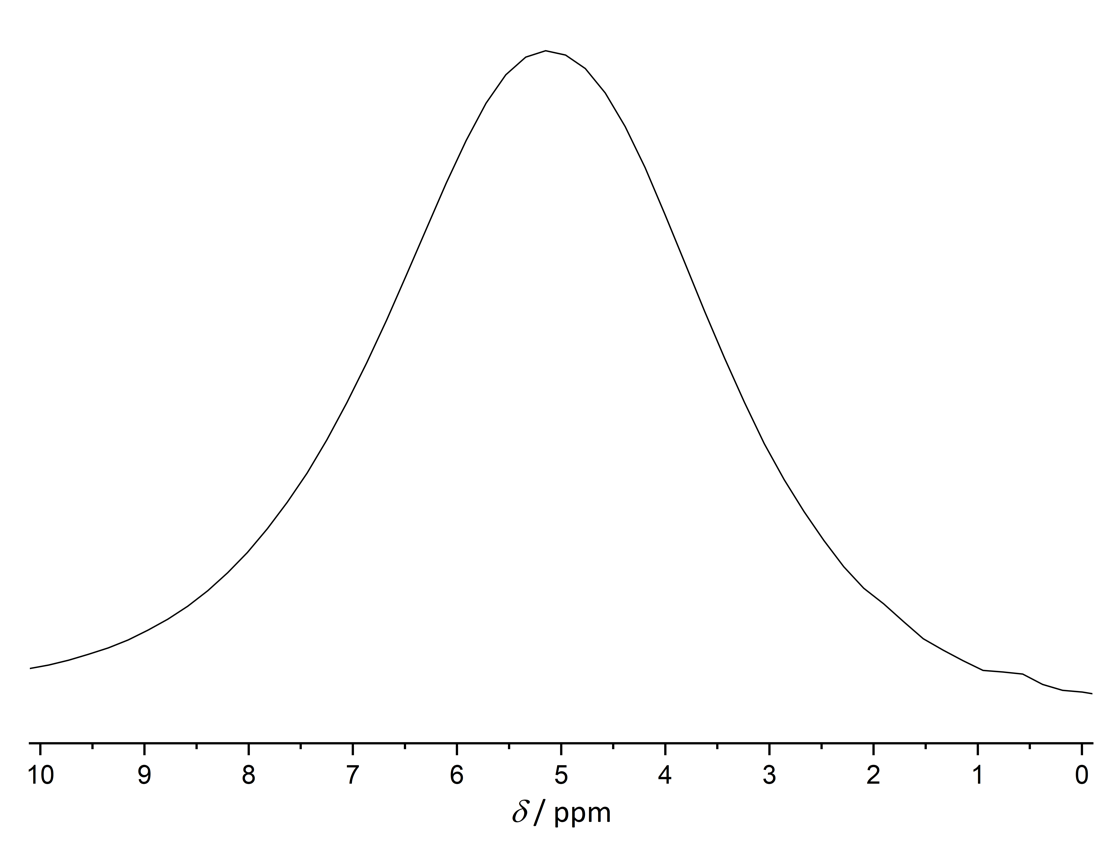


Fig. S8. ^1^H DP MAS NMR spectrum of [Cu{NCCH_2_N(CF_3_)_2_}_4_)][BF_4_] ([Cu(**1**)_4_][BF_4_]).


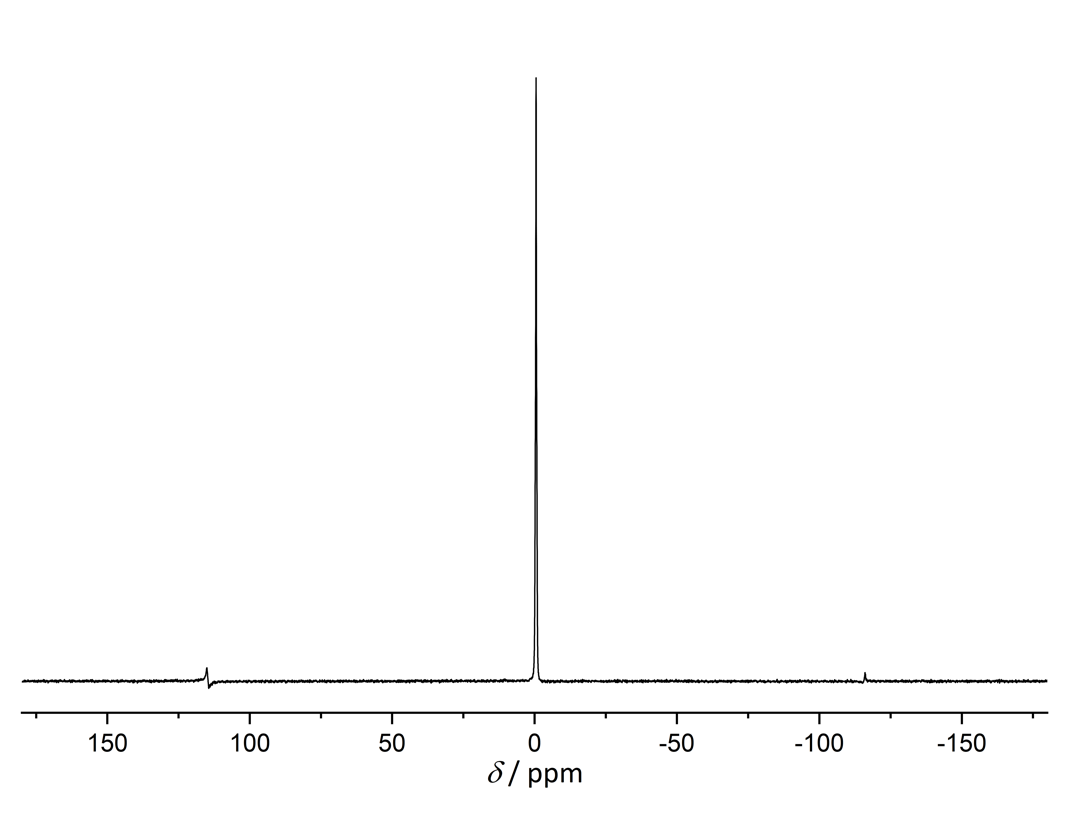


Fig. S9. ^11^B{^1^H} RSHE MAS NMR spectrum of [Cu{NCCH_2_N(CF_3_)_2_}_4_)][BF_4_] ([Cu(**1**)_4_][BF_4_]).


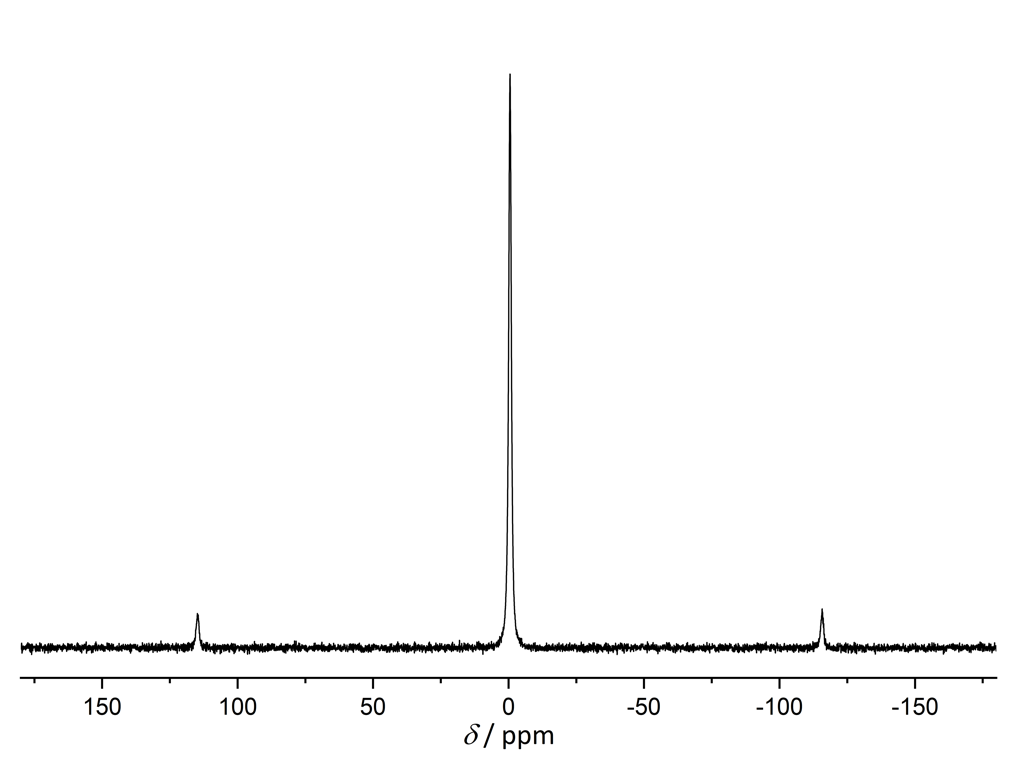


Fig. S10. ^11^B{^19^F} RSHE MAS NMR spectrum of [Cu{NCCH_2_N(CF_3_)_2_}_4_)][BF_4_] ([Cu(**1**)_4_][BF_4_]).


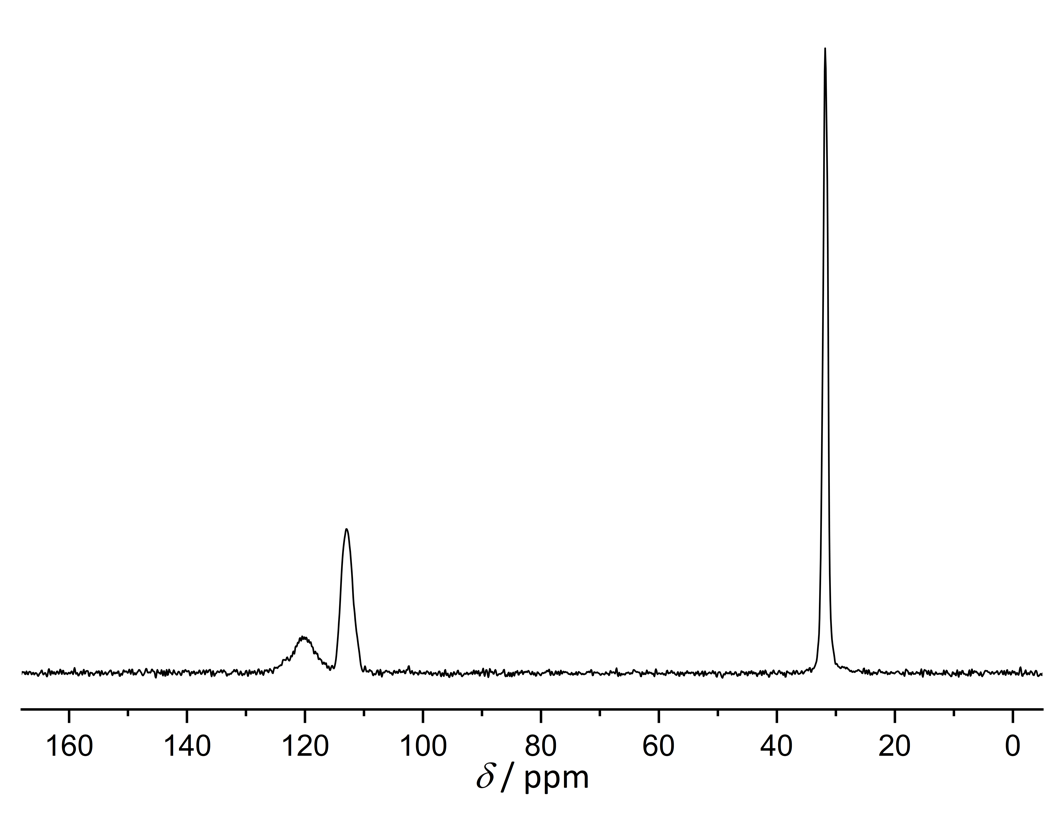


Fig. S11. ^13^C{^1^H} CP MAS NMR spectrum of [Cu{NCCH_2_N(CF_3_)_2_}_4_)][BF_4_] ([Cu(**1**)_4_][BF_4_]).


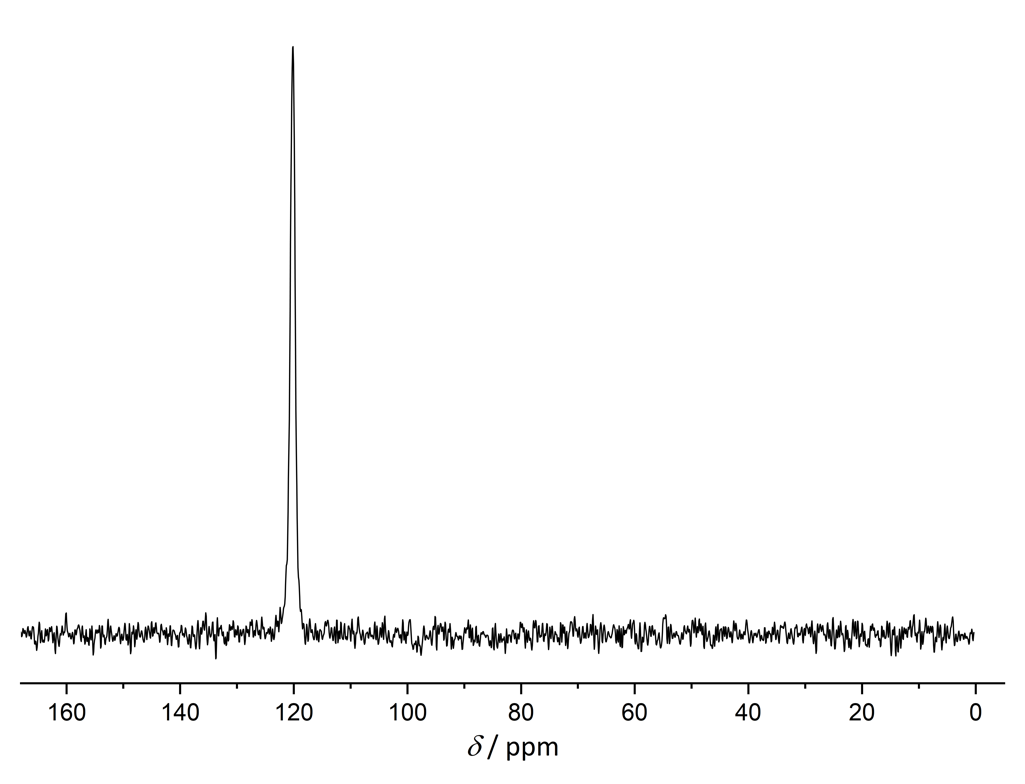


Fig. S12. ^13^C{^19^F} CP MAS NMR spectrum of [Cu{NCCH_2_N(CF_3_)_2_}_4_)][BF_4_] ([Cu(**1**)_4_][BF_4_]).


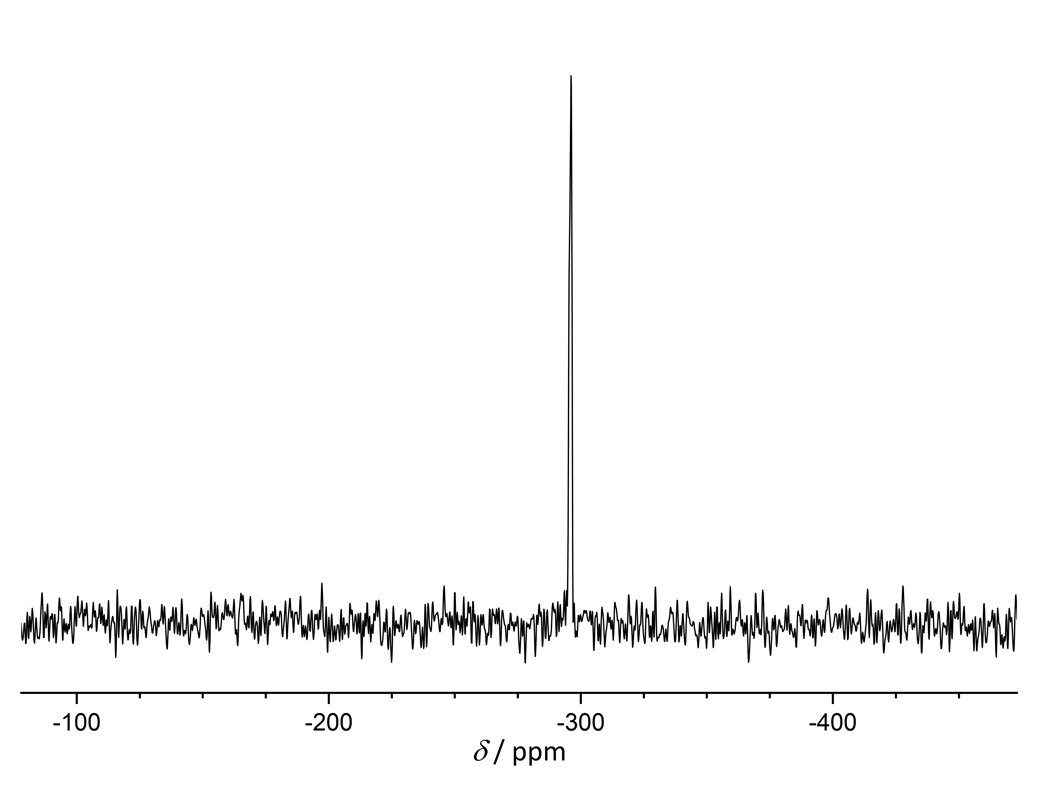


Fig. S13. ^15^N CP MAS NMR spectrum of [Cu{NCCH_2_N(CF_3_)_2_}_4_)][BF_4_] ([Cu(**1**)_4_][BF_4_]).


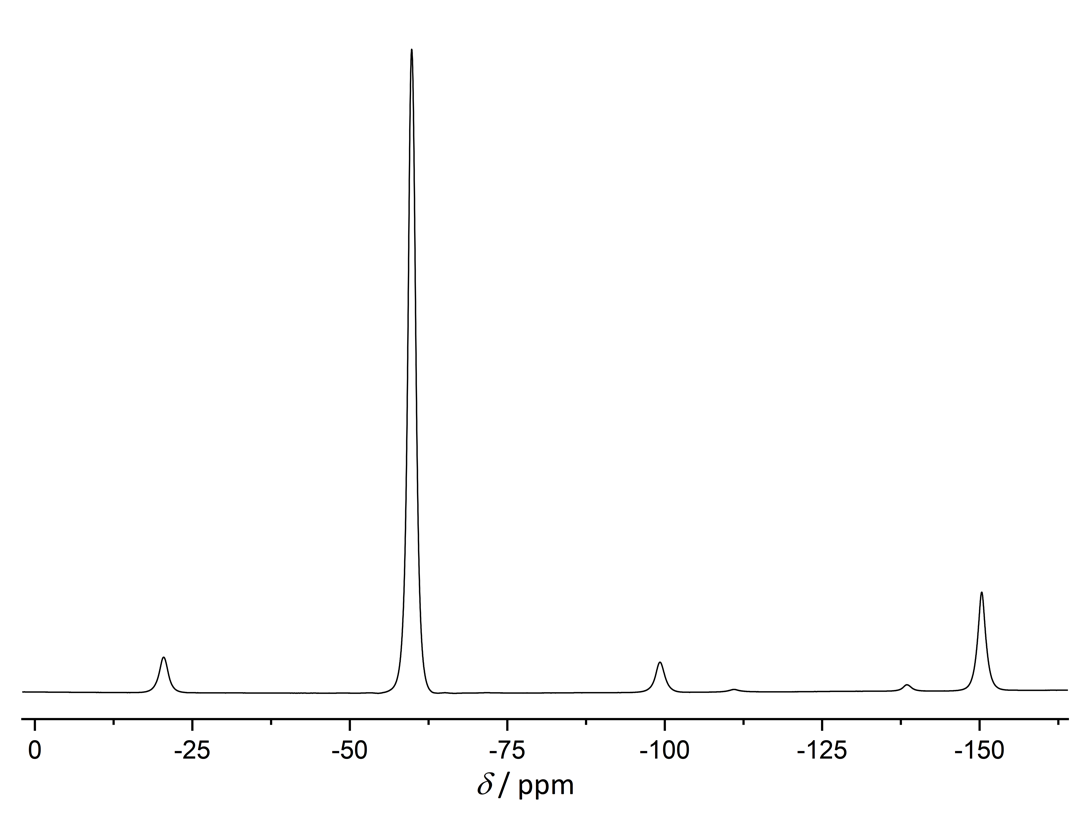


Fig. S14. ^19^F ZG MAS NMR spectrum of [Cu{NCCH_2_N(CF_3_)_2_}_4_)][BF_4_] ([Cu(**1**)_4_][BF_4_]).


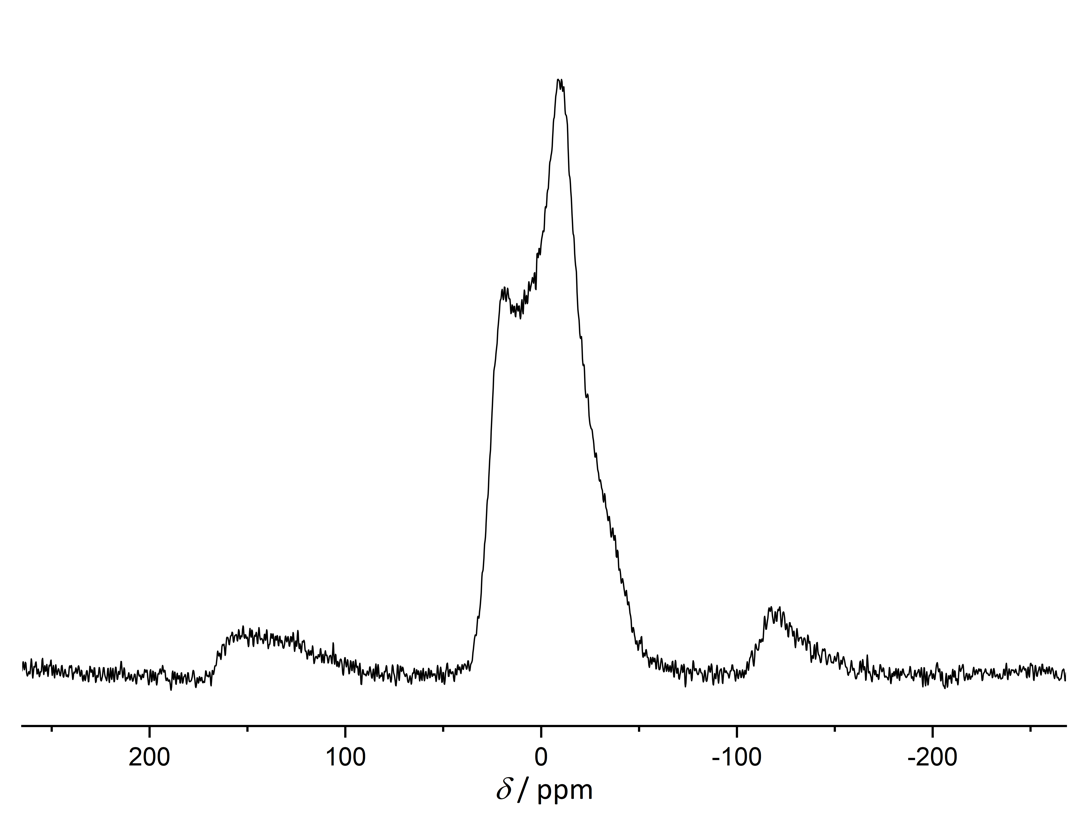


Fig. S14. ^63^Cu RSHE MAS NMR spectrum of [Cu{NCCH_2_N(CF_3_)_2_}_4_)][BF_4_] ([Cu(**1**)_4_][BF_4_]).


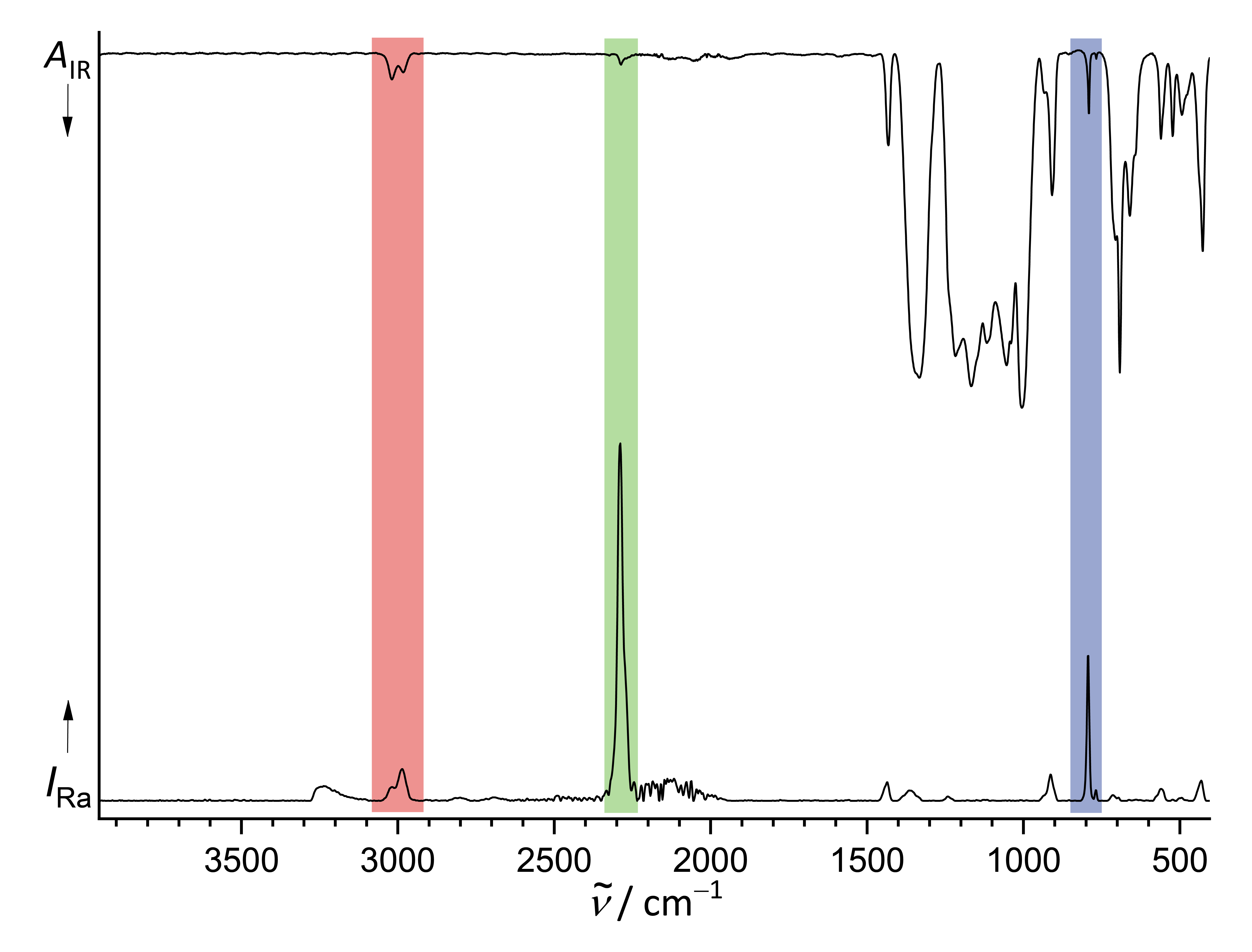


Fig. S15. IR (top) and Raman spectrum (bottom) of [Cu{NCCH_2_N(CF_3_)_2_}_4_)][BF_4_] ([Cu(**1**)_4_][BF_4_]).

Fig. S16. STA study of [Cu{NCCH_2_N(CF_3_)_2_}_4_)][BF_4_] ([Cu(**1**)_4_][BF_4_]).

Fig. S17. STA study of [Cu{NCCH_2_N(CF_3_)_2_}_4_)][BF_4_] ([Cu(**1**)_4_][BF_4_]).

**Synthesis of [Ph_3_PAuCH(CN){N(CF_3_)_2_}] (Au1) and [(Ph_3_Pau)_2_C(CN){N(CF_3_)_2_}] (Au_2_1)**

(F_3_C)_2_NCH_2_CN (166 mg, 863 µmol) was dissolved in tetrahydrofuran (20 mL) and cooled to
–78 °C. *n*-Butyllithium (0.4 mL, 780 µmol, 1.6 M in *n*-hexane) was added dropwise and the reaction mixture was stirred 2 hours at –78 °C and 30 minutes at –30 °C. The deprotonation was monitored by ^19^F NMR spectroscopy. After cooling to –78 °C, a solution of [Ph_3_PAuCl] (371 mg, 750 µmol) in tetrahydrofuran (12 mL) was slowly added and the reaction mixture was stirred while warming up to room temperature overnight. The mixture was studied by ^19^F NMR spectroscopy. The mixture consisted of [Ph_3_PAuCH(CN){N(CF_3_)_2_}] (**Au1**) as main component (80 %) and equimolar amounts of [(Ph_3_PAu)_2_C(CN){N(CF_3_)_2_}] (**Au_2_1**) and **1**.

All volatiles were removed under reduced pressure to give a yellow-orange solid that was suspended in dichloromethane (20 mL). The suspension was filtered to give a yellow solution. The solid remainder on the filter was extracted with dichloromethane (2 × 2 mL). The dichloromethane fractions were combined and most of the solvent was removed under reduced pressure. Addition of pentane (ca. 1 mL) led to precipitation of an off-white solid that contained the two gold(I) complexes **Au1** and **Au_2_1**. Crystallization from dichloromethane afforded a few single-crystals of **Au_2_1** suitable for a SC-XRD study (*vide infra*).

^19^F NMR (377 MHz, CH_2_Cl_2_): δ = −54.8 (s, 6F, N(CF_3_)_2_, **Au_2_1**), −56.6 (d, 6F, N(CF_3_)_2_, ^4^*J*(^19^F,^1^H) = 1.1 Hz, **Au1**), −59.2 ppm (t, 6F, N(CF_3_)_2_, ^4^*J*(^19^F,^1^H) = 1.2 Hz, **1**).

^31^P{^1^H} NMR (162, THF-d_8_): δ = 36.6 ppm (br, Ph_3_PAu).

**2-*N*,*N*-Bis(trifluoromethyl)amino-3-(naphthalen-2-yl)acrylonitrile (2)**

A solution of *N,N*-bis*(*trifluoromethyl)aminoacetonitrile (730 mg, 3.80 mmol; **1**) in THF (2 mL) was added to a solution of *n*-butyllithium (1.5 mL, 3.75 mmol, 2.5 M in hexane) in THF (5 mL) at −78 °C. After ten minutes, a solution of 2-naphthaldehyde (588 mg, 3.77 mmol) was added to THF (2 mL) and the reaction mixture was heated to room temperature for 3 hours. The solvent was removed in vacuum and 2-*N,N*-bis(trifluoromethyl)amino-3-(naphthalene-2-yl)acrylonitrile (**2**) was obtained as a mixture of isomers after sublimation (1 · 10^−3^ mbar,
60 °C) as a colorless solid. Yield: 738 mg (2.24 mmol, 59% based on **1**).

Elemental analysis: calculated (%) for C_15_H_8_F_6_N_2_, C 54.56, H 2.44, N 8.48; found, C 55.17, H 2.58, N 8.38.

HRMS (ASAP+) m/z, calculated for C_15_H_9_F_6_N_2_^+^: 331.0665 (100%), 332.0698 (16.2%), 333.0732 (1.2%); found: 331.0651 (100%), 332.0687 (17.4%), 333.0721 (1.3%).

***E* isomer (86%):**

^1^H NMR (500 MHz, CD_3_CN): *δ* = 8.33 (s, 1H, *Naph*─C*H*), 8.02 (d, 2H, *J*_H,H_ = 1.3 Hz, *Naph*-*H*), 8.00─7.83 (m, 3H, *Naph*-*H*), 7.67 (ddd, 1H, *J*_H,H_ = 8.2, *J*_H,H_ = 6.9, *J*_H,H_ = 1.3 Hz, *Naph*-*H*), 7.61 ppm (ddd, 1H, *J*_H,H_ = 8.2, *J*_H,H_ = 6.9, *J*_H,H_ = 1.3 Hz, *Naph*-*H*).

^13^C{^1^H} NMR (126 MHz, CD_3_CN): *δ* = 155.4 (sept, 1C, ^4^*J*_F,C_ = 1.0 Hz *Naph*─*C*H), 136.1 (s, 1C, *Naph*-*C*), 133.8 (s, 1C, *Naph*-*C*), 133.7 (s, 1C, *Naph*-*C*), 130.1 (s, 1C, *Naph*-*C*), 130.1 (s, 1C, *Naph*-*C*), 130.1 (s, 1C, *Naph*-*C*), 128.9 (s, 1C, *Naph*-*C*), 128.5 (s, 1C, *Naph*-*C*), 128.3 (s, 1C, *Naph*-*C*), 125.2 (s, 1C, *Naph*-*C*), 120.6 (qm, 2C, ^1^*J*_F,C_ = 264 Hz, *C*F_3_), 116.1 (sept, 1C, *J*_F,C_ = 1.3 Hz, *C*≡N oder NC─*C*), 100.8 ppm (sept, 1C, *J*_F,C_ = 0.7 Hz, *C*≡N oder NC─*C*).

^13^C{^19^F} NMR (126 MHz, CD_3_CN): *δ* = 155.4 (ddt, 1C, ^1^*J*_C,H_ = 161, *J*_C,H_ = 5.8, *J*_C,H_ = 2.1 Hz *Naph*─*C*H), 136.1 (m, 1C, *Naph*-*C*), 133.8 (dm, 1C, ^1^*J*_C,H_ = 162 Hz, *Naph*-*C*), 133.7 (m, 1C, *Naph*-*C*), 130.8─129.4 (several m, 3C, *Naph*-*C*), 129.1─127.7 (several m, 3C, *Naph*-*C*), 125.2 (ddd, 1C, ^1^*J*_C,H_ = 164, *J*_C,H_ = 6.7, *J*_C,H_ = 5.4 Hz, *Naph*-*C*), 120.6 (s, 2C, *C*F_3_), 116.1 (d, 1C, *J*_C,H_ = 11.7 Hz, *C*≡N oder NC─*C*), 100.8 ppm (d, 1C, *J*_C,H_ = 5.2 Hz, *C*≡N oder NC─*C*).

^15^N NMR (^15^N-^19^F HMBC, CD_3_CN): *δ* = −282.7 ppm (s, 1N, *N*(CF_3_)_2_).

^19^F NMR (471 MHz, CD_3_CN): *δ* = −57.3 ppm (s, 6F, C*F*_3_).

***Z* isomer (14%):**

^1^H NMR (500 MHz, CD_3_CN): *δ* = 8.33 (s, 1H, *Naph*-C*H*), 8.00─7.87 (m, 4H, *Naph*-*H*), 7.85 (dd, 1H, *J*_H,H_ = 8.7, *J*_H,H_= 1.8 Hz , *Naph*-*H*), 7.66 (ddd, 1H, *J*_H,H_ = 8.2, *J*_H,H_ = 6.9, *J*_H,H_ = 1.3 Hz, *Naph*-*H*), 7.61 ppm (ddd, 1H, *J*_H,H_ = 8.2, *J*_H,H_ = 6.9, *J*_H,H_ = 1.3 Hz, *Naph*-*H*).

^13^C{^1^H} NMR (126 MHz, CD_3_CN): *δ* = 154.0 (sept, 1C, ^4^*J*_F,C_ = 0.9 Hz, *Naph*─*C*H), 135.9 (s, 1C, *Naph*-*C*), 135.2 (s, 1C, *Naph*-*C*), 133.7 (s, 1C, *Naph*-*C*), 130.2 (s, 1C, *Naph*-*C*), 130.2 (s, 1C, *Naph*-*C*), 130.2 (s, 1C, *Naph*-*C*), 129.1 (s, 1C, *Naph*-*C*), 128.8 (s, 1C, *Naph*-*C*), 128.4 (s, 1C, *Naph*-*C*), 125.9 (s, 1C, *Naph*-*C*), 120.5 (qm, 2C, ^1^*J*_F,C_ = 264 Hz, *C*F_3_), 116.7 (sept, 1C, ^3^*J*_F,C_ = 1.2 Hz, *C*≡N oder NC─*C*), 101.2 ppm (sept, 1C, *J*_F,C_ = 0.8 Hz, *C*≡N oder NC─*C*).

^13^C{^19^F} NMR (126 MHz, CD_3_CN): *δ* = 154.0 (dm, 1C, ^1^*J*_C,H_ = 160 Hz, *Naph*─*C*H), 135.8 (several m, 2C, *Naph*-*C*), 133.7 (m, 1C, *Naph*-*C*), 130.9─129.3 (several m, 3C, *Naph*-*C*), 129.1─127.7 (several m, 3C, *Naph*-*C*), 125.8 (dm, 1C, ^1^*J*_C,H_ = 164 Hz, *Naph*-*C*), 120.5 (s, 2C, *C*F_3_), 116.7 (d, 1C, *J*_C,H_ = 5.7 Hz, *C*≡N oder NC─*C*), 101.2 ppm (d, 1C, *J*_C,H_ = 2.6 Hz, *C*≡N oder NC─*C*).

^15^N NMR (^15^N-^19^F HMBC, CD_3_CN): *δ* = −289.1 ppm (s, 1N, *N*(CF_3_)_2_).

^19^F NMR (471 MHz, CD_3_CN): *δ* = −58.2 ppm (s, 6F, C*F*_3_).

**
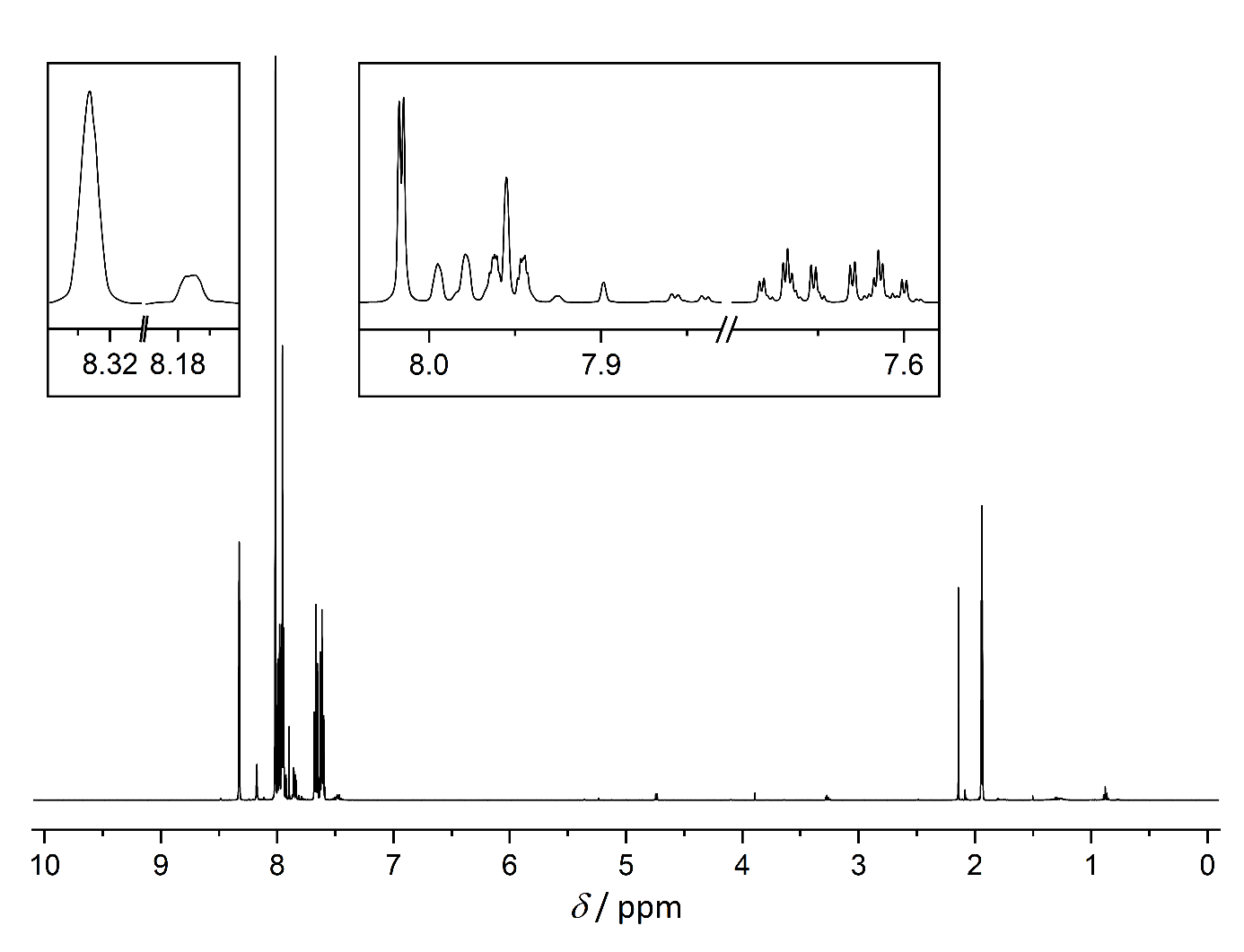
**

Fig. S18. ^1^H NMR spectrum of **2** (mixture of *E* and *Z* isomer).

**
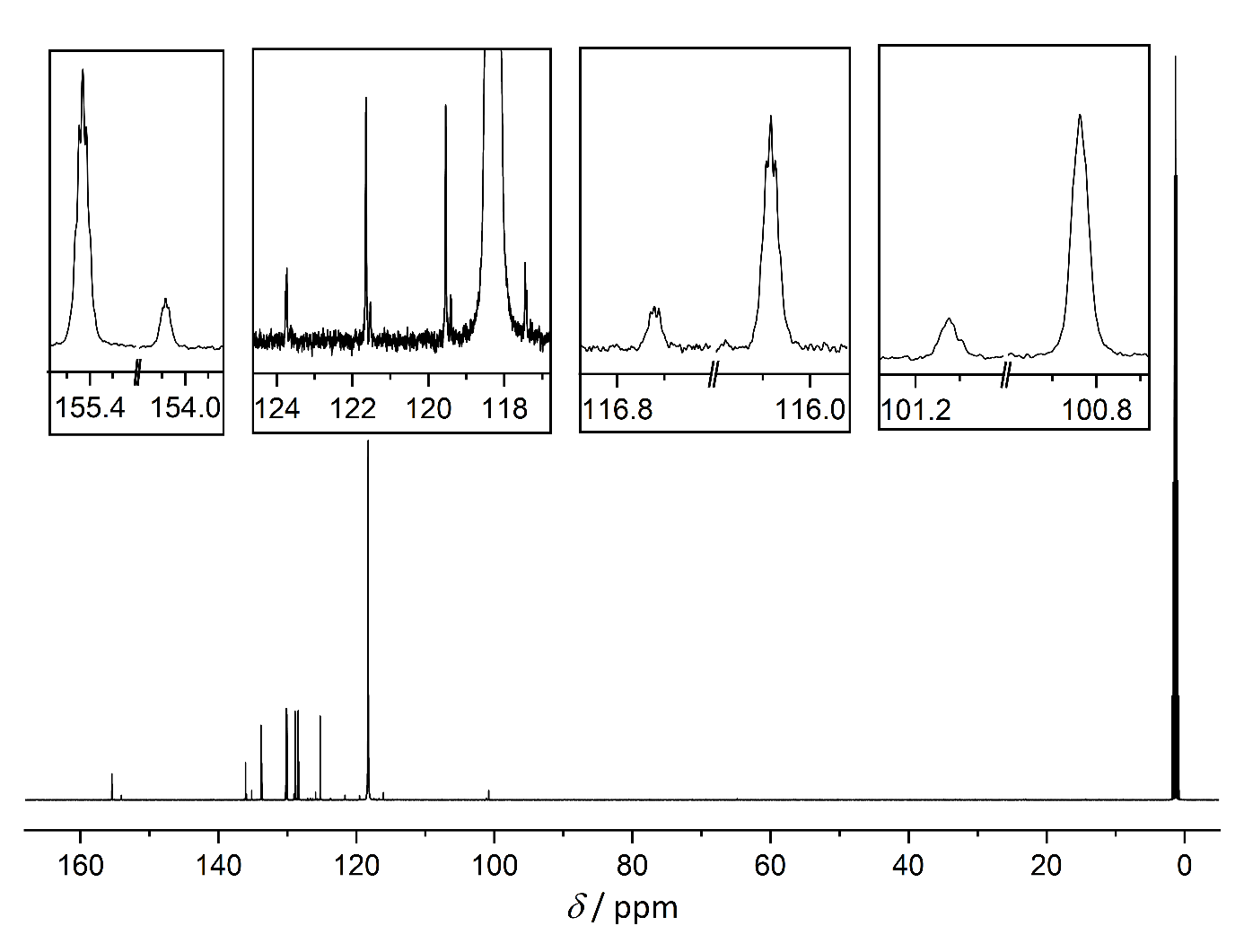
**

Fig. S19. ^13^C{^1^H} NMR spectrum of **2** (mixture of *E* and *Z* isomer).

**
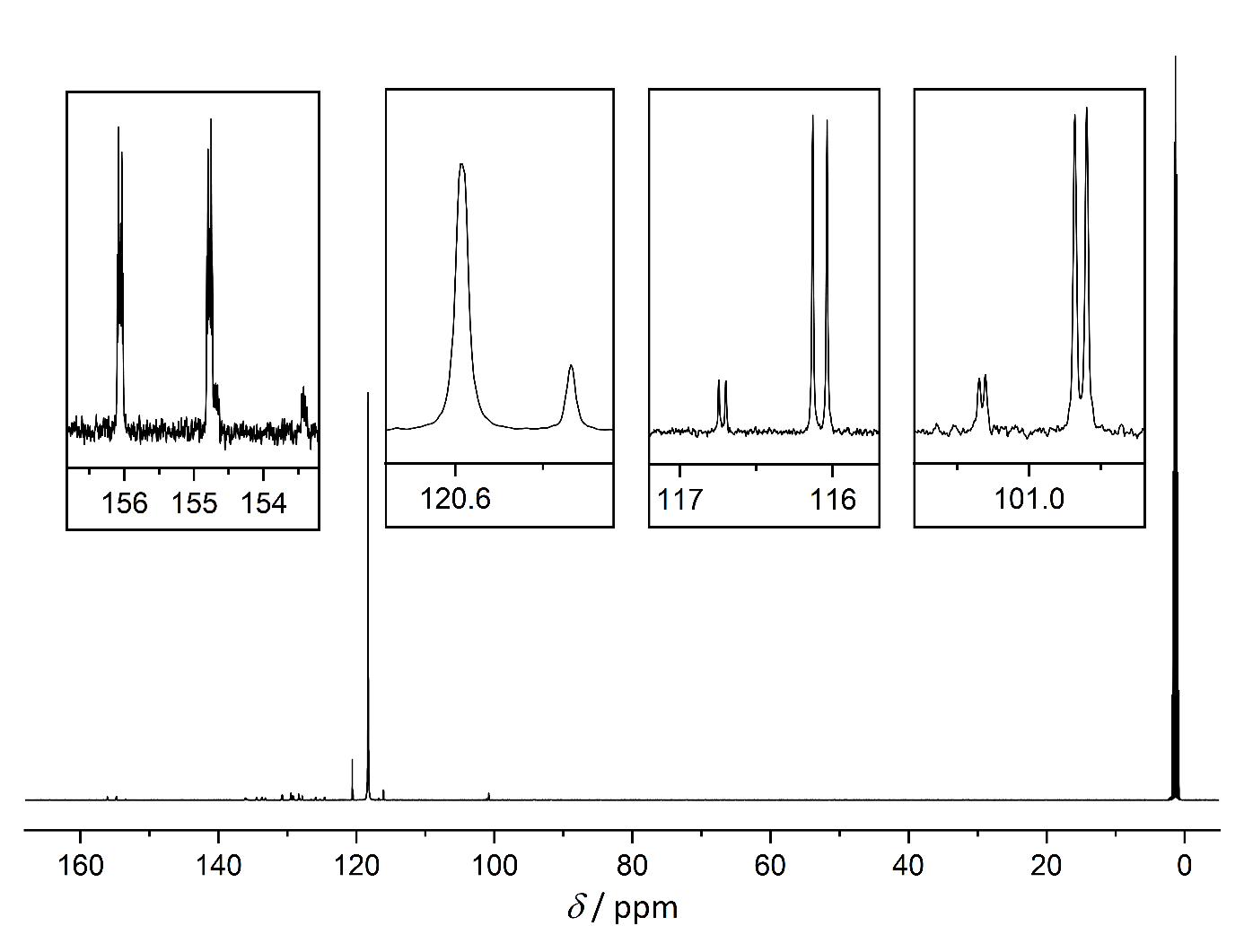
**

Fig. S20. ^13^C{^19^F} NMR spectrum of **2** (mixture of *E* and *Z* isomer).

**
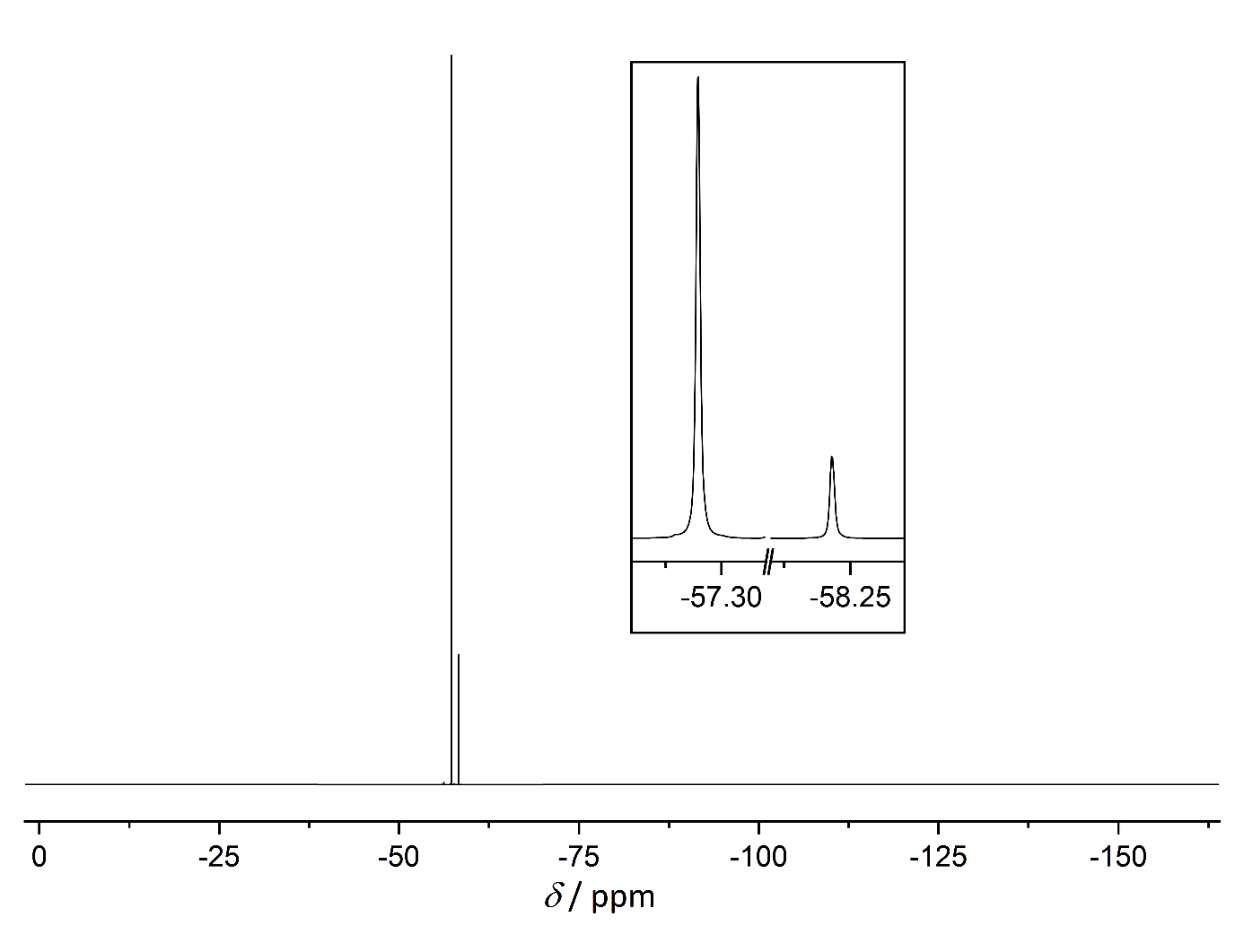
**

Fig. S21. ^19^F NMR spectrum of **2** (mixture of *E* and *Z* isomer).

**
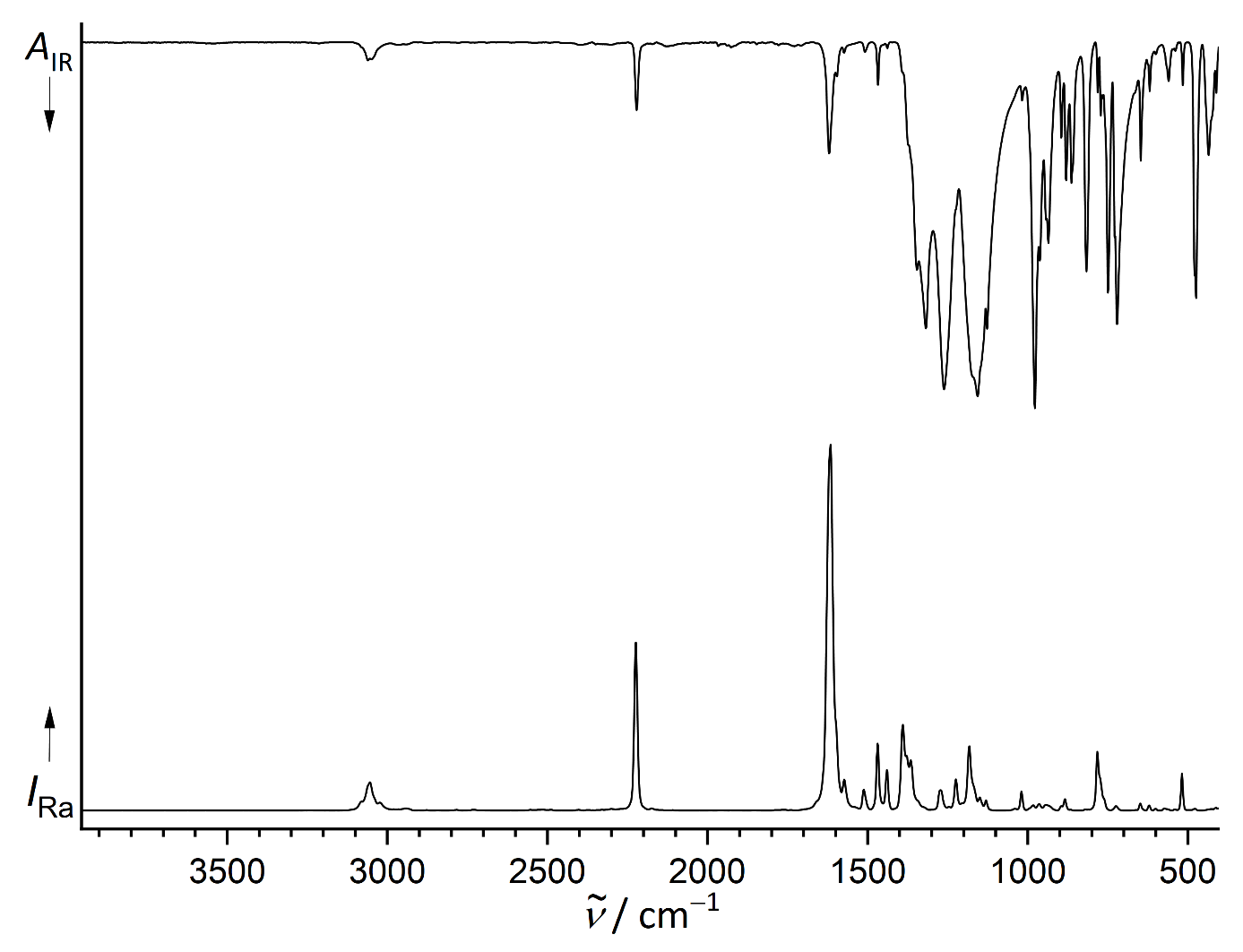
**

Fig. S22. IR (top) and Raman spectrum (bottom) of **2** (mixture of *E* and *Z* isomer).

**
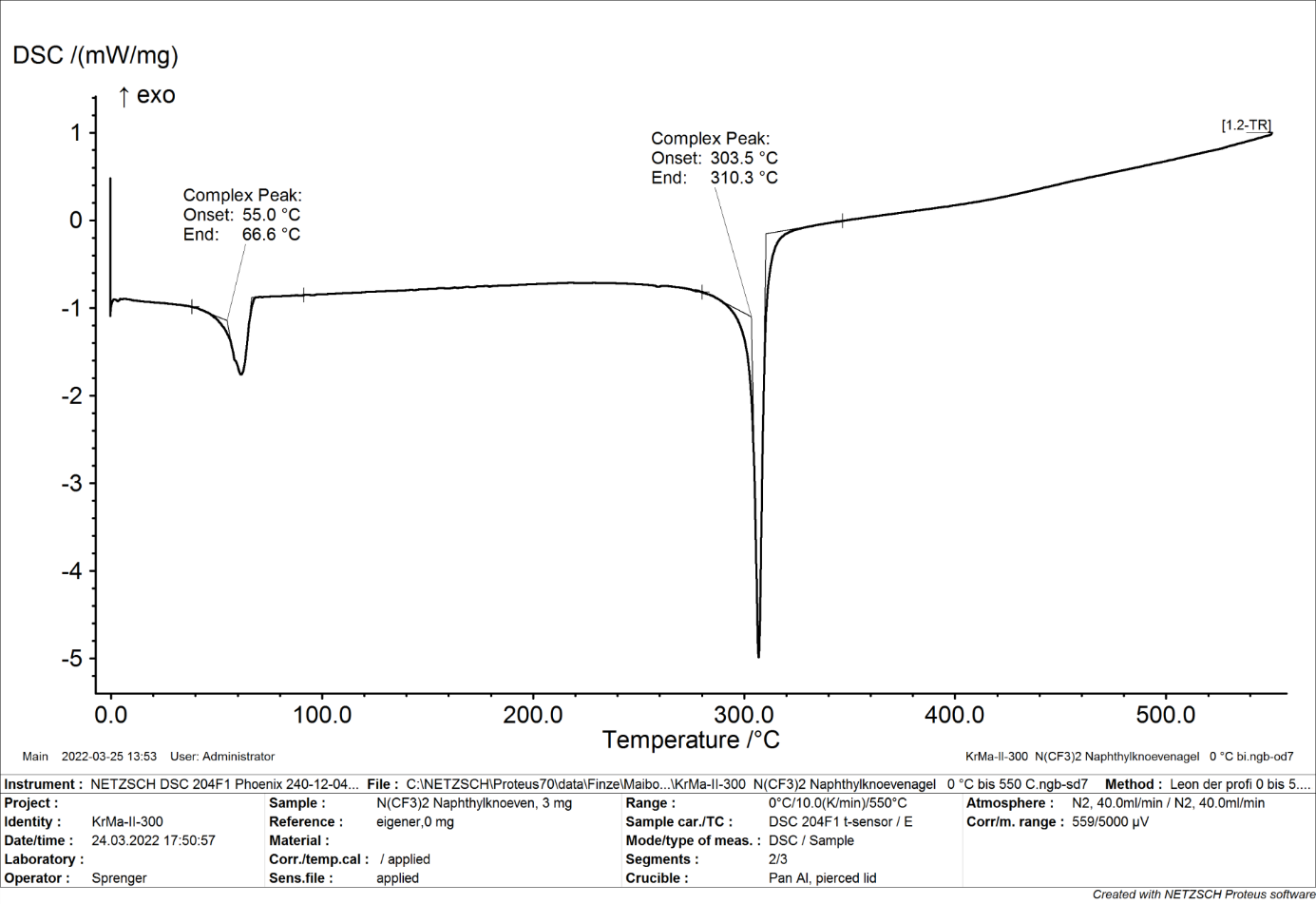
**

Fig. S22. DSC curve of **2** (mixture of *E* and *Z* isomer).

**2-*N*,*N*-Bis(trifluoromethyl)amino-3-(anthracen-9-yl)acrylonitrile (3)**

A solution of *N,N*-bis*(*trifluoromethyl)aminoacetonitrile (720 mg*,* 3.75 mmol; **1**) in THF (2 mL) was added to a solution of *n*-butyllithium (1.5 mL, 3.75 mmol, 2.5 M in hexane) in THF (5 mL) at −78 °C. After ten minutes, a solution of anthracene-9-carb-aldehyde (773 mg, 3.75 mmol) in THF (6 mL) was added and the reaction mixture was stirred at room temperature for three days. The solvent was removed in a vacuum and 2-*N,N*-Bis(trifluoromethyl)amino-3-(anthracene-9-yl)acrylonitrile (**3)** was obtained by column chromatography (DCM : hexane 5: 95) as a yellow solid. Fractions of the pure isomers that are both yellow solids were obtained. Yield: 1.09 g (2.87 mmol, 77% based on **1**).

Elemental analysis: calculated (%) for C_19_H_10_F_6_N_2_, C 60.01, H 2.65, N 7.37; found, C 60.35, H 2.69, N 7.45.

HRMS (ASAP+) m/z, calculated for C_19_H_11_F_6_N_2_^+^: 381.0821 (100%), 382.0855 (20.5%), 383.0889 (2.0%); found: 381.0810 (100%), 382.0849 (18.4%), 383.0883 (1.7%).

***E* isomer (93%):**

^1^H NMR (500 MHz, CD_3_CN): *δ* = 8.75 (d, 1H, *J*_H,H_ = 0.5 Hz, *Anth*─C*H*), 8.68 (s, 1H, *Anth*-*H*), 8.12 (ddd, 2H, *J*_H,H_ = 8.5, *J*_H,H_ = 1.3, *J*_H,H_ = 0.6 Hz, *Anth*-*H*), 7.88 (ddd, 2H, *J*_H,H_ = 8.8, *J*_H,H_ = 1.9, *J*_H,H_ = 0.9 Hz, *Anth*-*H*), 7.66 (ddd, 2H, *J*_H,H_ = 8.8, *J*_H,H_ = 6.6, *J*_H,H_ = 1.3 Hz, *Anth*-*H*), 7.61─7.57 (m, 2H, *Anth*-*H*) ppm.

^13^C{^1^H} NMR (126 MHz, CD_3_CN): *δ* = 156.1 (sept, 1C, *J*_F,C_ = 1.0 Hz, *Anth*─*C*H), 131.9 (s, 1C, *Anth*-*C*), 131.6 (s, 1C, *Anth*-*C*), 130.2 (s, 2C, *Anth*-*C*), 129.9 (s, 2C, *Anth*-*C*), 128.8 (s, 2C, *Anth*-*C*), 127.0 (s, 2C, *Anth*-*C*), 124.8 (s, 2C, *Anth*-*C*), 123.8 (s, 2C, *Anth*-*C*), 120.5 (qm, 2C, ^1^*J*_F,C_ = 266 Hz, *C*F_3_), 114.3 (sept, 1C, *J*_F,C_ = 1.3 Hz, *C*≡N or NC─*C*), 110.0 ppm (sept, 1C, *J*_F,C_ = 0.6 Hz, *C*≡N or NC─*C*).

^13^C{^19^F} NMR (126 MHz, CD_3_CN): *δ* = 156.0 (d, 1C, ^1^*J*_C,H_ = 167 Hz, *Anth*─*C*H), 131.9 (t, 1C, ^3^*J*_C,H_ = 6.8 Hz, *Anth*-*C*), 131.6 (dt, 1C, ^1^*J*_C,H_ = 164, ^3^*J*_C,H_ = 5.9 Hz, *Anth*-*C*), 130.9─129.5 (m, 2C, *Anth*-*C*), 129.9 (dd, 2C, ^2^*J*_C,H_ = 14.4, ^3^*J*_C,H_ = 7.6 Hz, *Anth*-*C*), 128.8 (dd, 2C, ^1^*J*_C,H_ = 162, *J*_C,H_ = 9.0 Hz, *Anth*-*C*), 127.0 (dd, 2C, ^1^*J*_C,H_ = 162, *J*_C,H_ = 8.5 Hz, *Anth*-*C*), 124.8 (dd, 2C, ^1^*J*_C,H_ = 159, ^3^*J*_C,H_ = 6.9 Hz, *Anth*-*C*), 123.9─123.7 (m, 2C, *Anth*-*C*), 120.5 (s, 2C, *C*F_3_), 114.3 (d, 1C, *J*_C,H_ = 11.9 Hz, *C*≡N or NC─*C*), 110.0 ppm (d, 1C, *J*_C,H_ = 5.3 Hz, *C*≡N or NC─*C*).

^15^N NMR (^15^N^19^F-HMBC, CD_3_CN): *δ* = −282.8 ppm (s, 1N, *N*(CF_3_)_2_).

^19^F NMR (471 MHz, CD_3_CN): *δ* = −57.0 ppm (s, 6F, C*F*_3_).

***Z* isomer (7%):**

^1^H NMR (500 MHz, CD_3_CN): *δ* = 8.73 (d, 1H, *J*_H,H_ = 0.9 Hz, Anth─C*H*), 8.63 (s, 1H, *Anth*-*H*), 8.08 (ddd, 2H, *J*_H,H_ = 8.0, *J*_H,H_ = 1.5, *J*_H,H_ = 0.6 Hz, *Anth*-*H*), 7.93 (dm, 2H, *J*_H,H_ = 8.6 Hz, *Anth*-*H*), 7.61─7.57 (m, 2H, *Anth*-*H*), 7.55 ppm (ddd, *J*_H,H_ = 7.9, *J*_H,H_ = 4.6, *J*_H,H_ = 1.2 Hz, *Anth*-*H*).

^13^C{^1^H} NMR (126 MHz, CD_3_CN): *δ* = 153.6 (sept, 1C, ^4^*J*_F,C_ = 1.0 Hz, Anth─*C*H), 131.8 (s, 1C, *Anth*-*C*), 130.9 (s, 1C, *Anth*-*C*), 129.9 (s, 2C, *Anth*-*C*), 129.6 (s, 2C, *Anth*-*C*), 128.0 (s, 2C, *Anth*-*C*), 126.8 (s, 2C, *Anth*-*C*), 125.5 (s, 2C, *Anth*-*C*), 124.2 (s, 2C, *Anth*-*C*), 120.1 (qm, 2C, ^1^*J*_F,C_ = 266 Hz, *C*F_3_), 115.6 (sept, 1C, *J*_F,C_ = 1.5 Hz, *C*≡N or NC─*C*), 109.1 ppm (sept, 1C, *J*_F,C_ = 0.7 Hz, *C*≡N or NC─*C*).

^13^C{^19^F} NMR (126 MHz, CD_3_CN): *δ* = 153.6 (d, 1C, ^1^*J*_C,H_ = 164 Hz, Anth─*C*H), 132.4─129.0 (mehrere m, 6C, *Anth*-*C*), 128.0 (dd, 2C, ^1^*J*_C,H_ = 161, ^3^*J*_C,H_ = 8.5 Hz, *Anth*-*C*), 126.8 (dd, 2C, ^1^*J*_C,H_ = 162, ^3^*J*_C,H_ = 8.2 Hz, *Anth*-*C*), 125.5 (dd, 2C, ^1^*J*_C,H_ = 160, ^3^*J*_C,H_ = 6.9 Hz, *Anth*-*C*), 124.9─123.7 (m, 2C, *Anth*-*C*), 120.1 (s, 2C, *C*F_3_), 115.6 (d, 1C, *J*_C,H_ = 5.7 Hz, *C*≡N oder NC─*C*), 109.1 ppm (d, 1C, *J*_C,H_ = 2.3 Hz, *C*≡N oder NC─*C*).

^15^N NMR (^15^N^19^F-HMBC, CD_3_CN): *δ* = −285.9 ppm (s, 1N, *N*(CF_3_)_2_).

^19^F NMR (471 MHz, CD_3_CN): *δ* = −56.7 ppm (s, 6F, C*F*_3_).

**
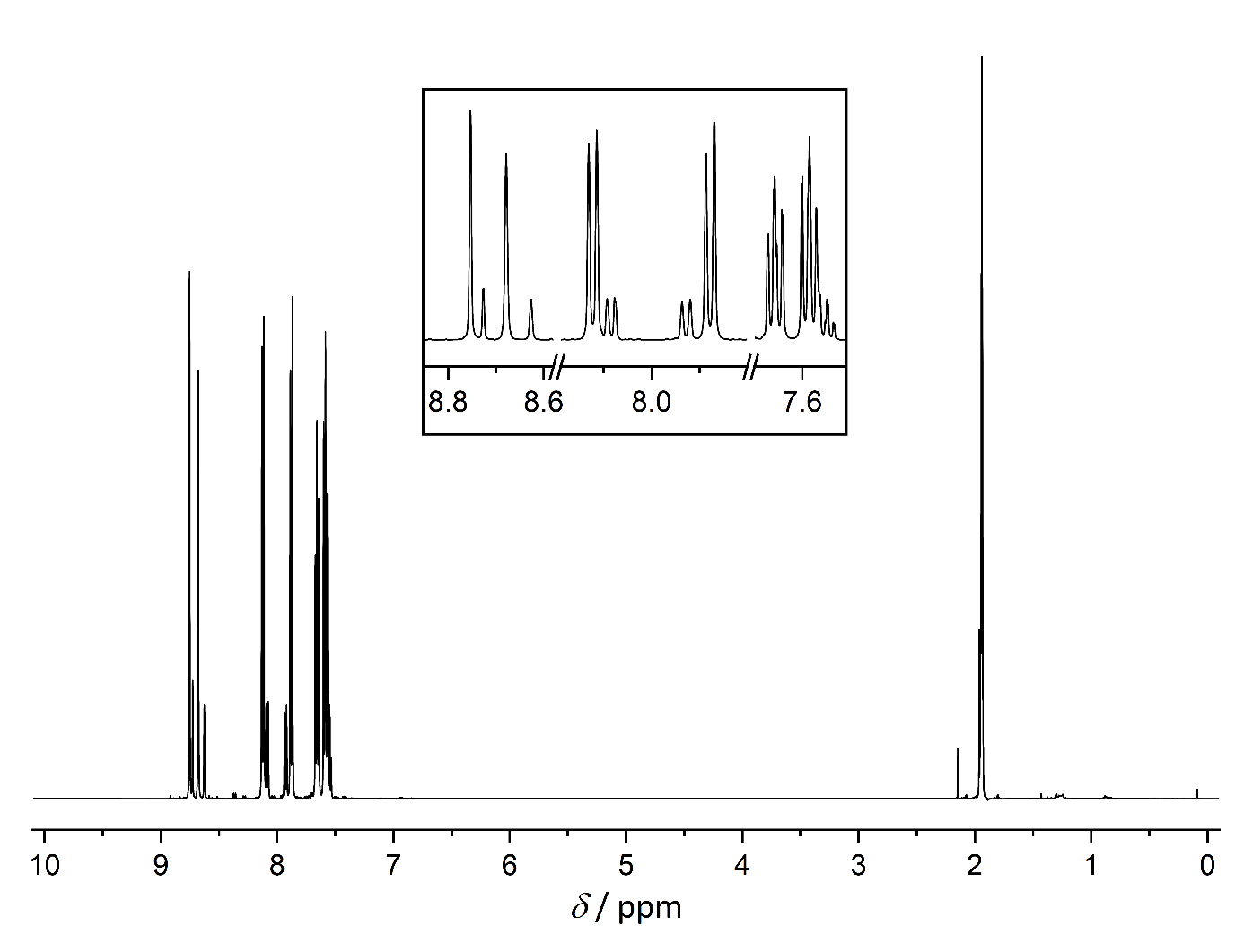
**

Fig. S23. ^1^H NMR spectrum of **3** (mixture of *E* and *Z* isomer).

**
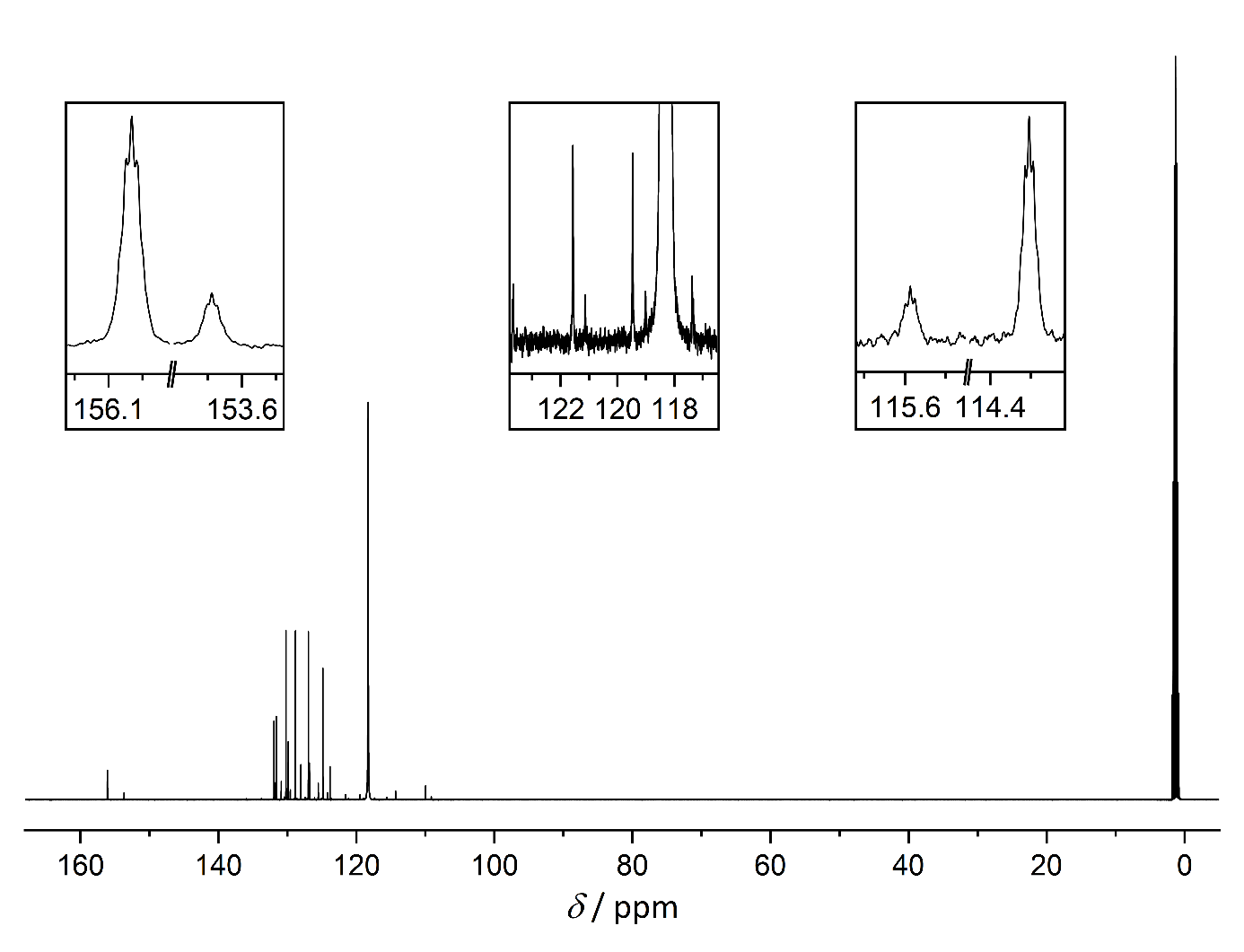
**

Fig. S24. ^13^C{^1^H} NMR spectrum of **3** (mixture of *E* and *Z* isomer).

**
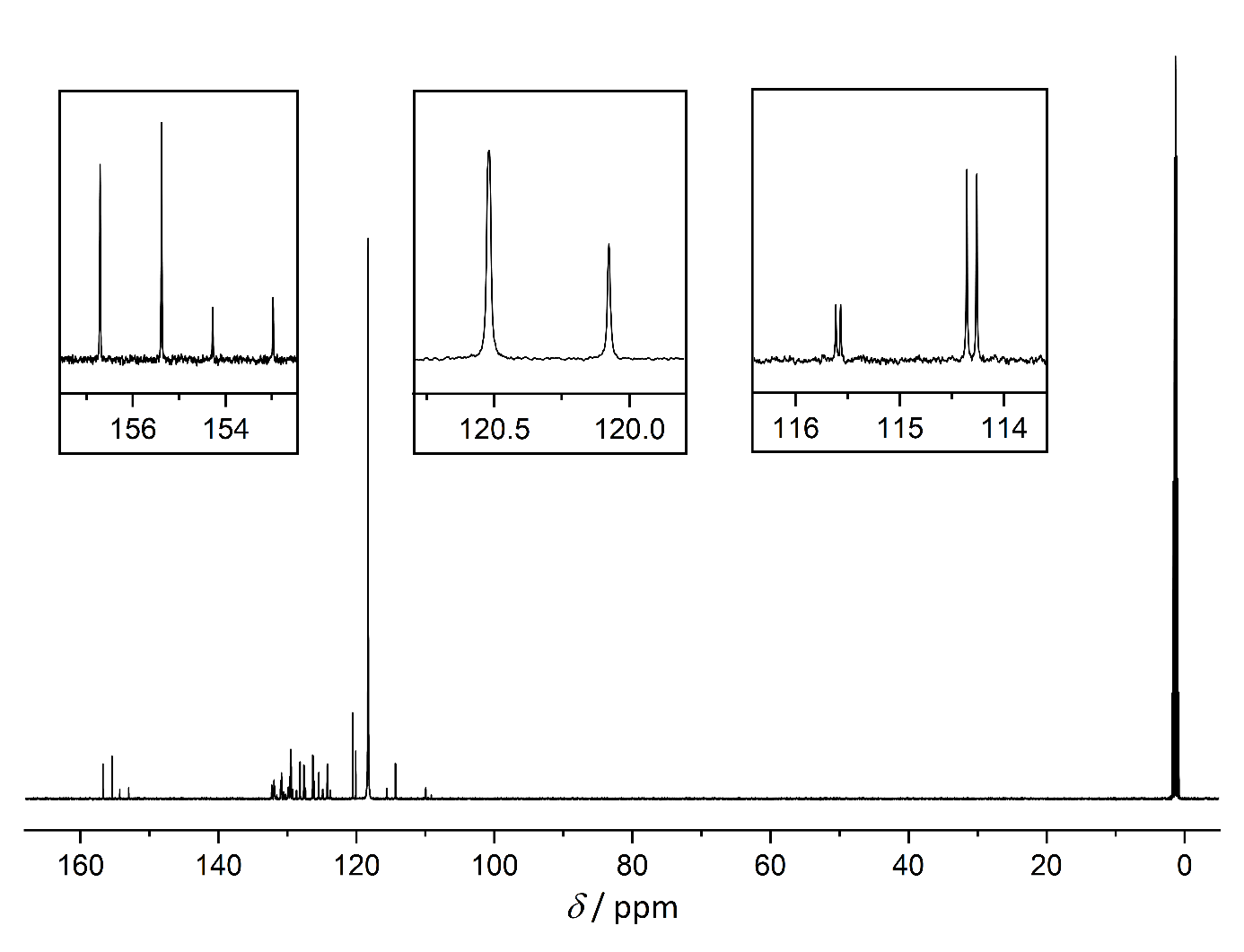
**

Fig. S25. ^13^C{^19^F} NMR spectrum of **3** (mixture of *E* and *Z* isomer).

**
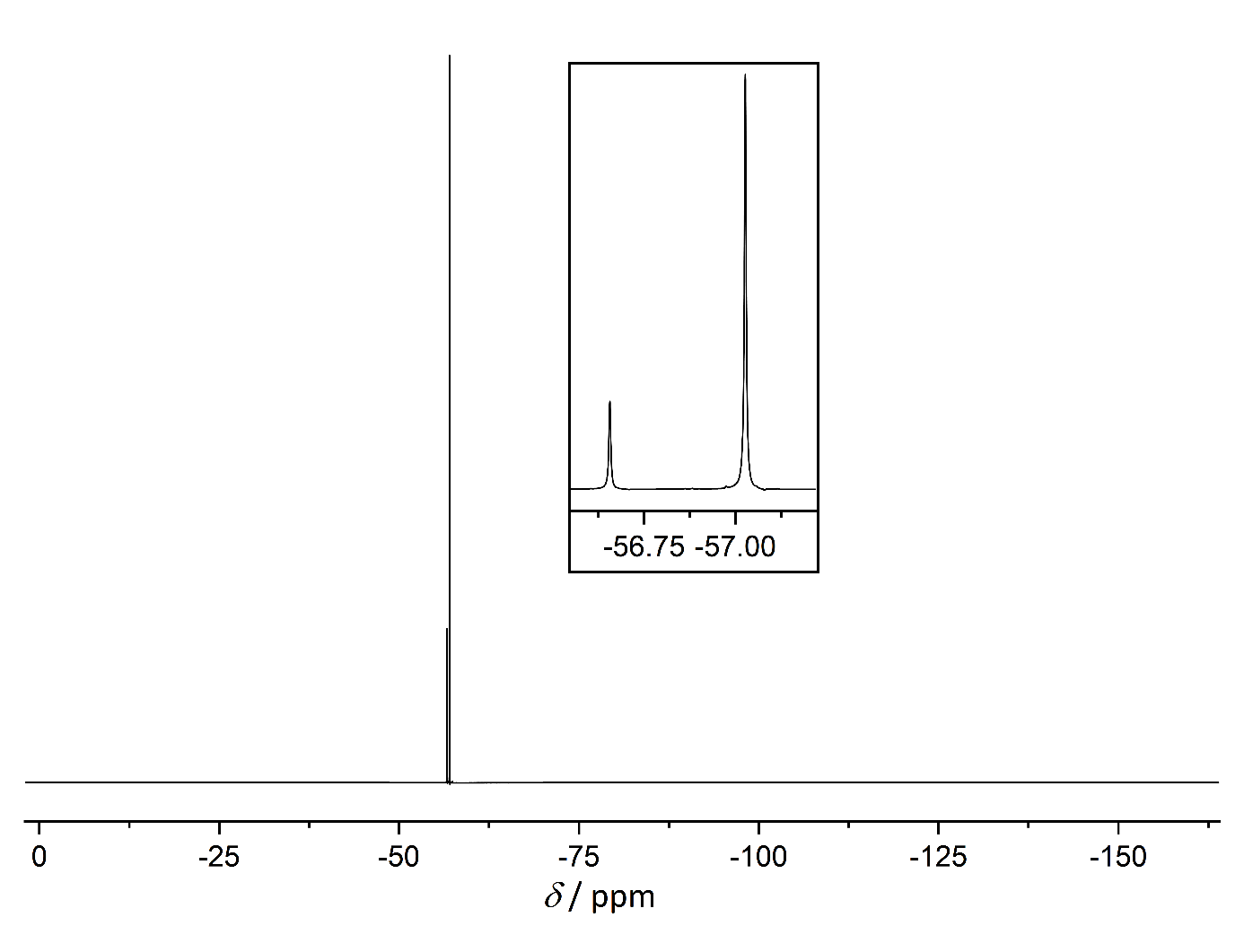
**

Fig. S26. ^19^F NMR spectrum of **3** (mixture of *E* and *Z* isomer).

**
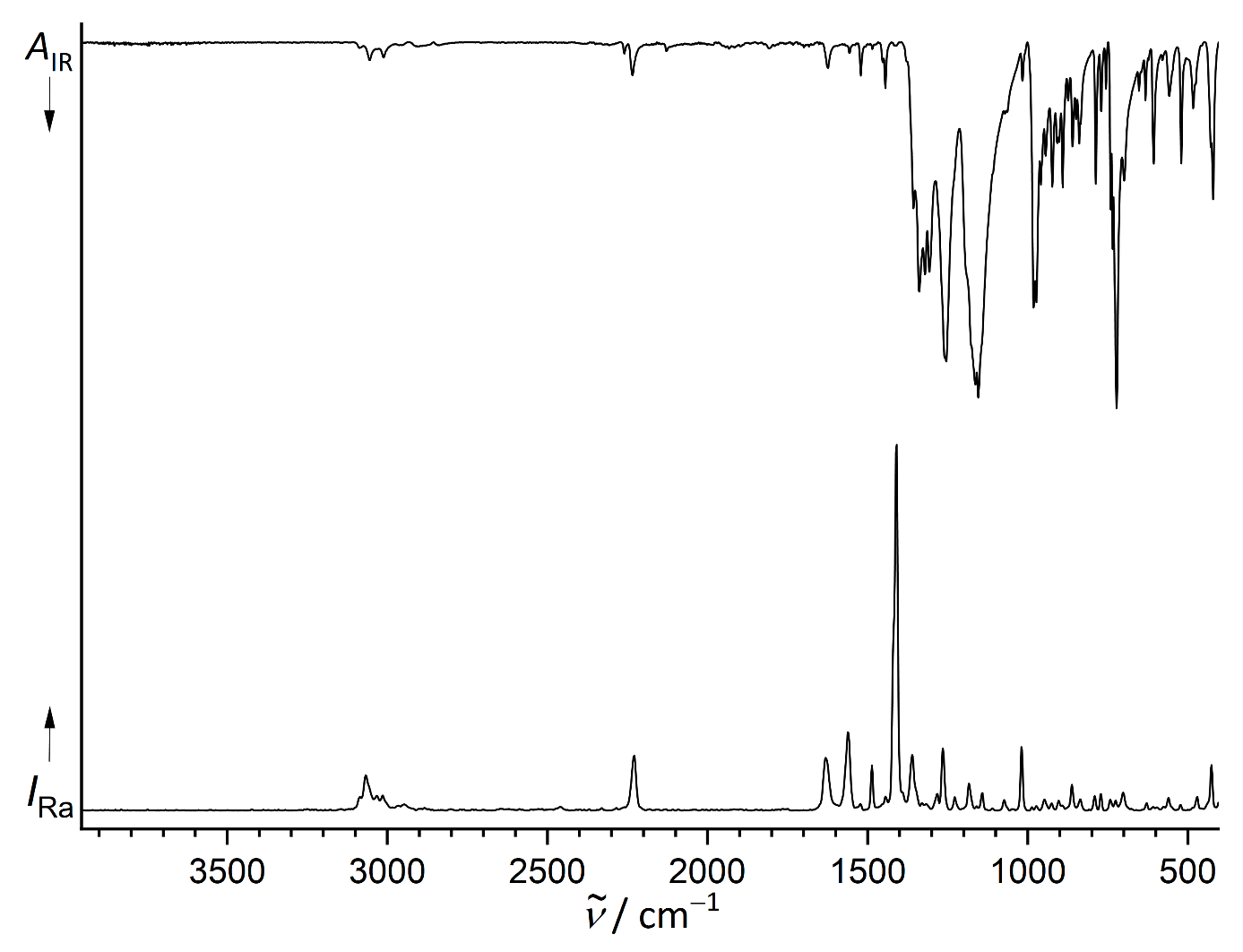
**

Fig. S27. IR (top) and Raman spectrum (bottom) of **3** (mixture of *E* and *Z* isomer).

**
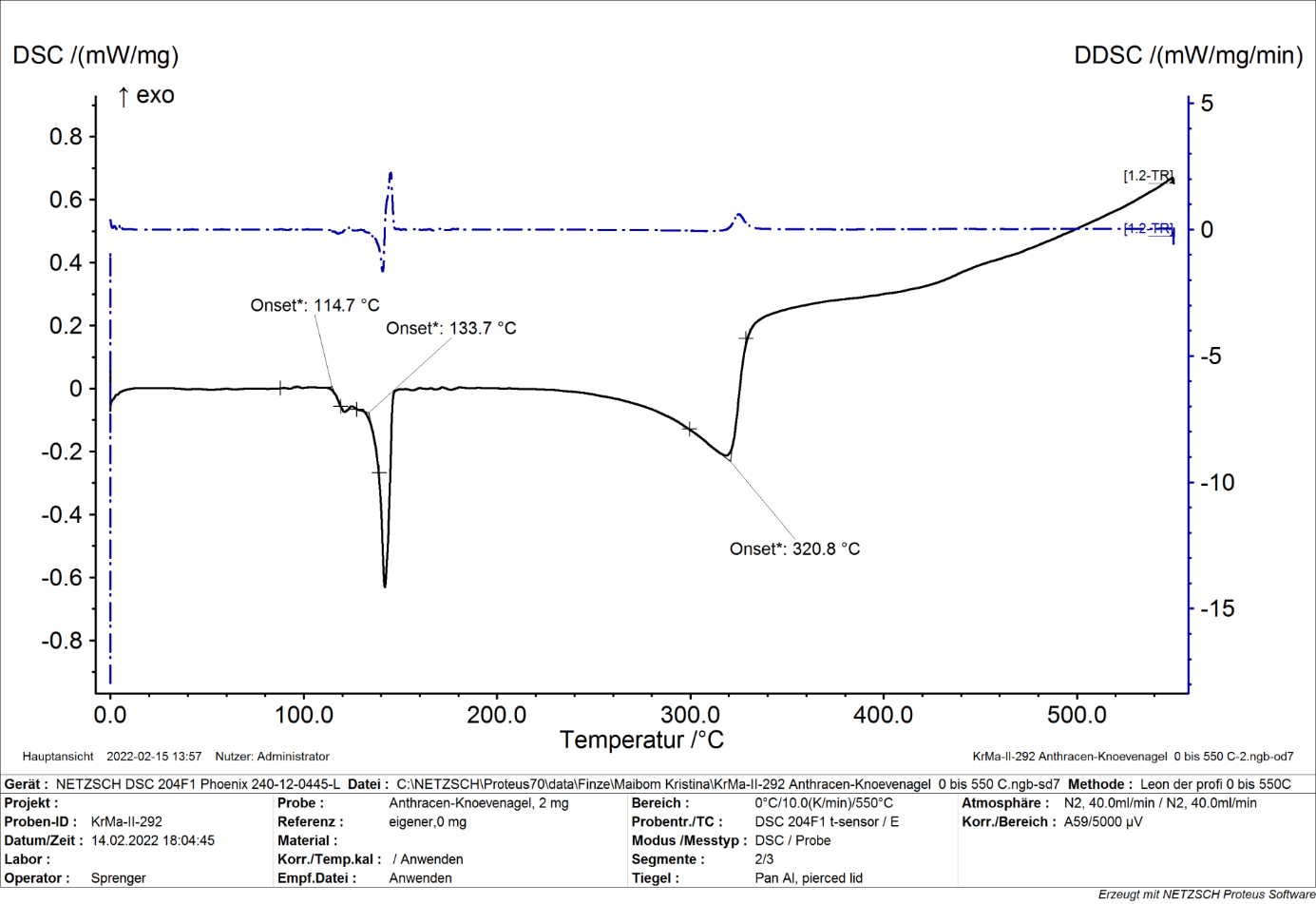
**

Fig. S28. DSC curve of **3** (mixture of *E* and *Z* isomer).

**2-*N*,*N*-Bis(trifluoromethyl)amino-3,3-diphenylacrylonitrile (4)**

A solution of *N,N*-bis*(*trifluoromethyl)aminoacetonitrile (731 mg, 3.81 mmol; **1**) in THF (2 mL) was added to a solution of *n*-butyllithium (1.5 mL, 3.75 mmol, 2.5 M in hexane) in THF (5 mL) at −78 °C. After ten minutes, a solution of benzophenone (690 mg, 3.79 mmol) in THF (2 mL) was added and the reaction mixture was stirred at room temperature for three days. The solvent was removed in a vacuum and 2-*N,N*-bis(trifluoromethyl)amino-3,3-diphenyl­acrylo­nitrile (**4**) was obtained as a colorless liquid by column chromatography (DCM : hexane 1:9). Yield: 882 mg (2.48 mmol, 65% based on **1**).

Elemental analysis: calculated (%) for C_17_H_10_F_6_N_2_, C 57.31, H 2.83, N 7.86; found, C 57.03, H 2.80, N 8.00.

HRMS (ASAP+) m/z, calculated for C_17_H_11_F_6_N_2_^+^: 357.0821 (100%), 358.0855 (18.4%), 359.0889 (1.6%); found: 357.0805 (100%), 358.0843 (16.9%), 359.0878 (1.4%).

^1^H NMR (500 MHz, CD_3_CN): δ = 7.61─7.56 (m, 1H, *Ph*-*H*), 7.54─7.45 (m, 7H, *Ph*-*H*), 7.25─7.21 ppm (m, 2H, *Ph*-*H*).

^13^C{^1^H} NMR (126 MHz, CD_3_CN): δ = 168.8 (sept, 1C, *J*_F,C_ = 0.8 Hz, NC─C─*C*), 137.2 (s, 1C, *Ph*-*C*_ipso_), 136.8 (s, 1C, *Ph*-*C*_ipso_), 132.6 (s, 2C, *Ph*-*C*H), 131.7 (s, 2C, *Ph*-*C*H), 130.6 (s, 2C, *Ph-C*H), 129.9 (s, 2C, *Ph -C*H), 129.8 (s, 1C, *Ph-C*H), 129.6 (s, 1C, *Ph-C*H), 120.5 (qm, 2C, ^1^*J*_F,C_= 263 Hz, *C*F_3_), 116.2 (sept, 1C, *J*_F,C_ = 1.3 Hz, *C*≡N or NC─*C*), 100.5 ppm (s, 1C, *C*≡N or NC─*C*).

^13^C{^19^F} NMR (126 MHz, CD_3_CN): δ = 168.8 (s, 1C, NC─C─*C*), 137.2 (tm, 1C, ^2^*J*_C,H_= 7.8 Hz, *Ph*-*C*_ipso_), 136.8 (tm, 1C, ^2^*J*_C,H_= 7.8 Hz, *Ph*-*C*_ipso_), 132.6 (dt, 2C, ^1^*J*_C,H_= 163, ^2^*J*_C,H_= 7.2 Hz, *Ph*-*C*H), 131.7 (dt, 2C, ^1^*J*_C,H_= 163, ^2^*J*_C,H_= 7.5 Hz, *Ph*-*C*H), 130.6 (dt, 2C, ^1^*J*_C,H_= 162, ^2^*J*_C,H_= 6.8 Hz, *Ph*-*C*H), 130.6─128.9 (several m, 4C, *Ph*-*C*H), 120.5 (s, 2C, *C*F_3_), 116.2 (s, 1C, *C*≡N or NC─*C*), 100.5 ppm (s, 1C, *C*≡N or NC─*C*).

^15^N NMR (^15^N^19^F HMBC, CD_3_CN): δ = −285.4 ppm (s, 1N, *N*(CF_3_)_2_).

^19^F NMR (471 MHz, CD_3_CN): δ = −57.7 ppm (s, 6F, C*F*_3_).


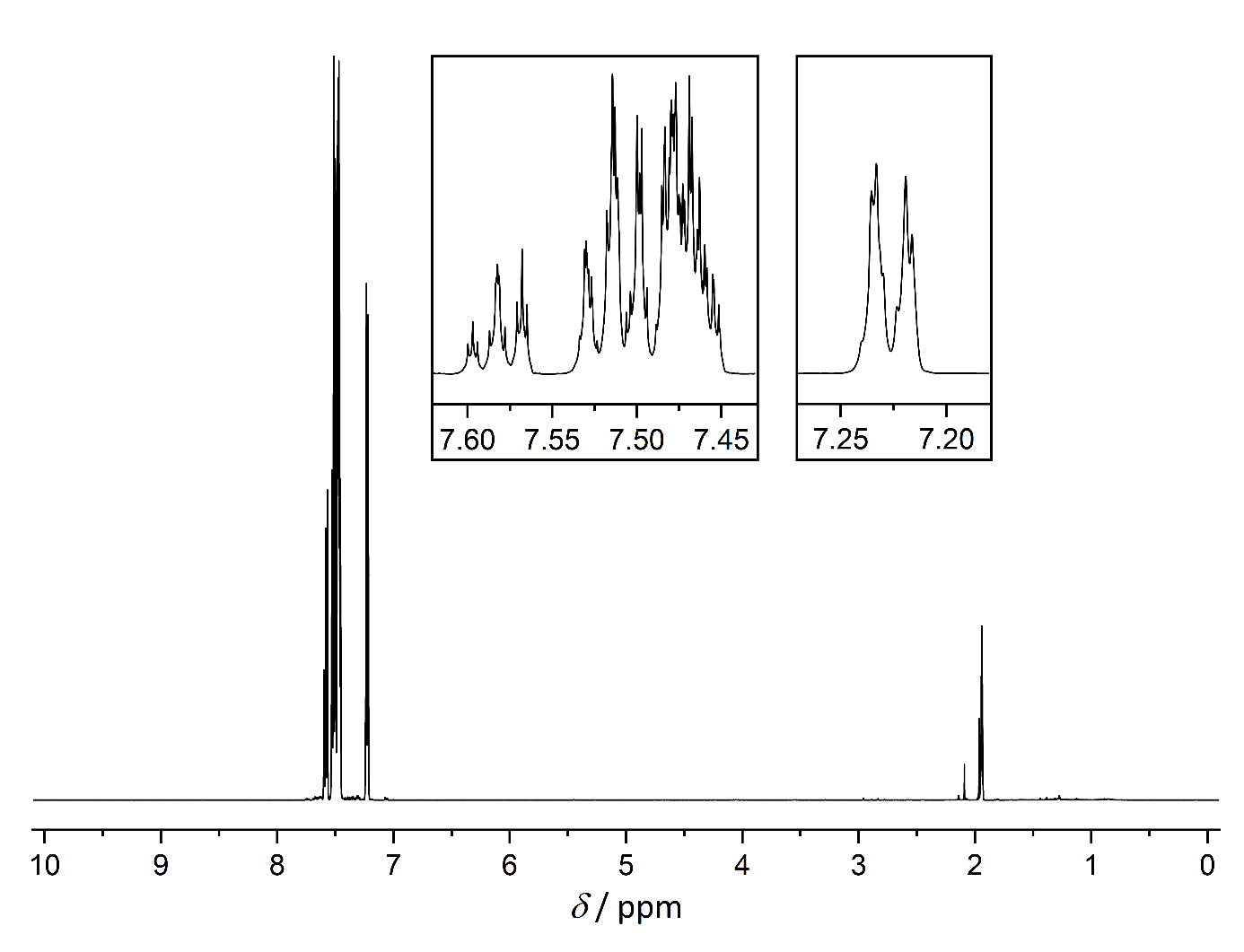


Fig. S29. ^1^H NMR spectrum of **4**.


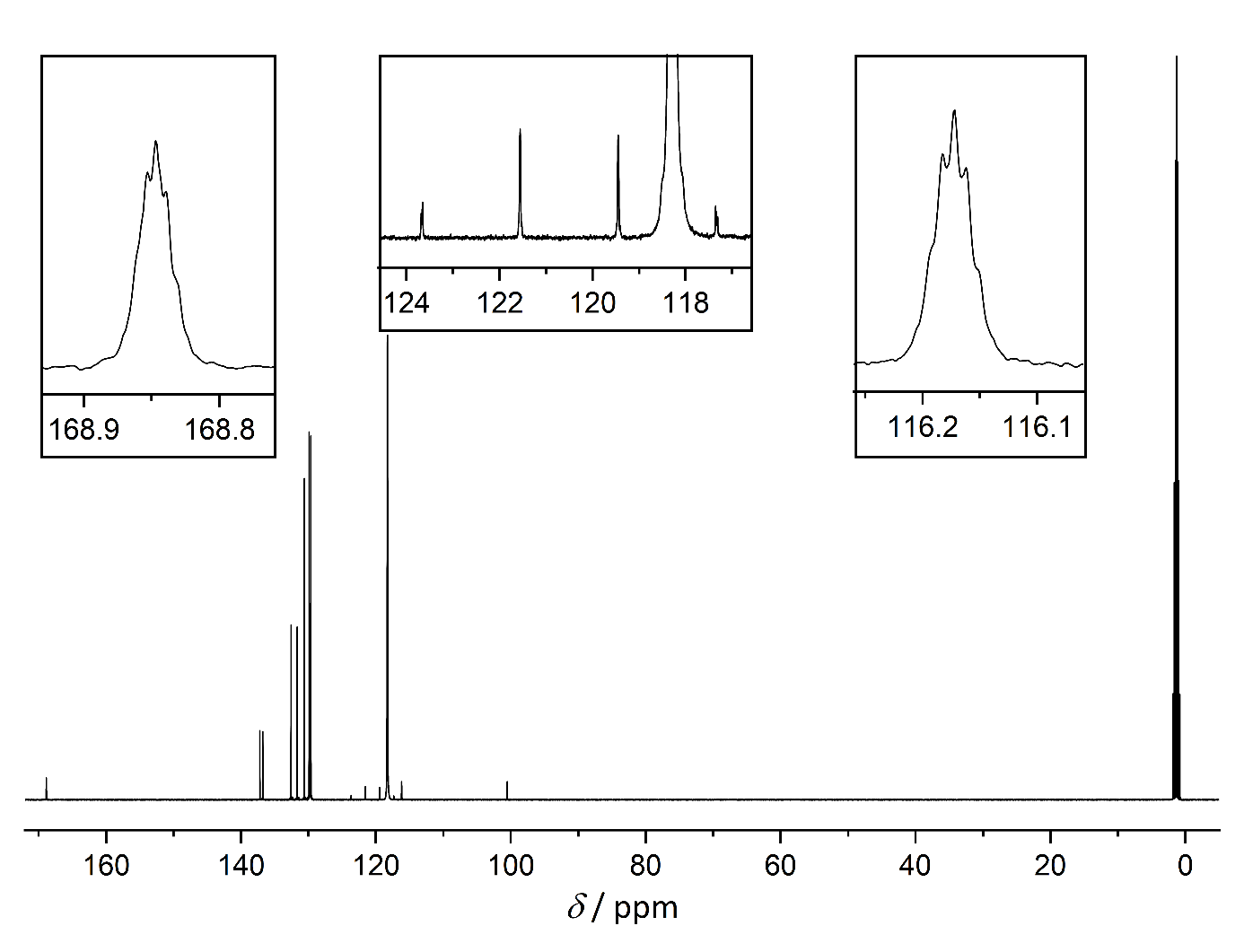


Fig. S30. ^13^C{^1^H} NMR spectrum of **4**.


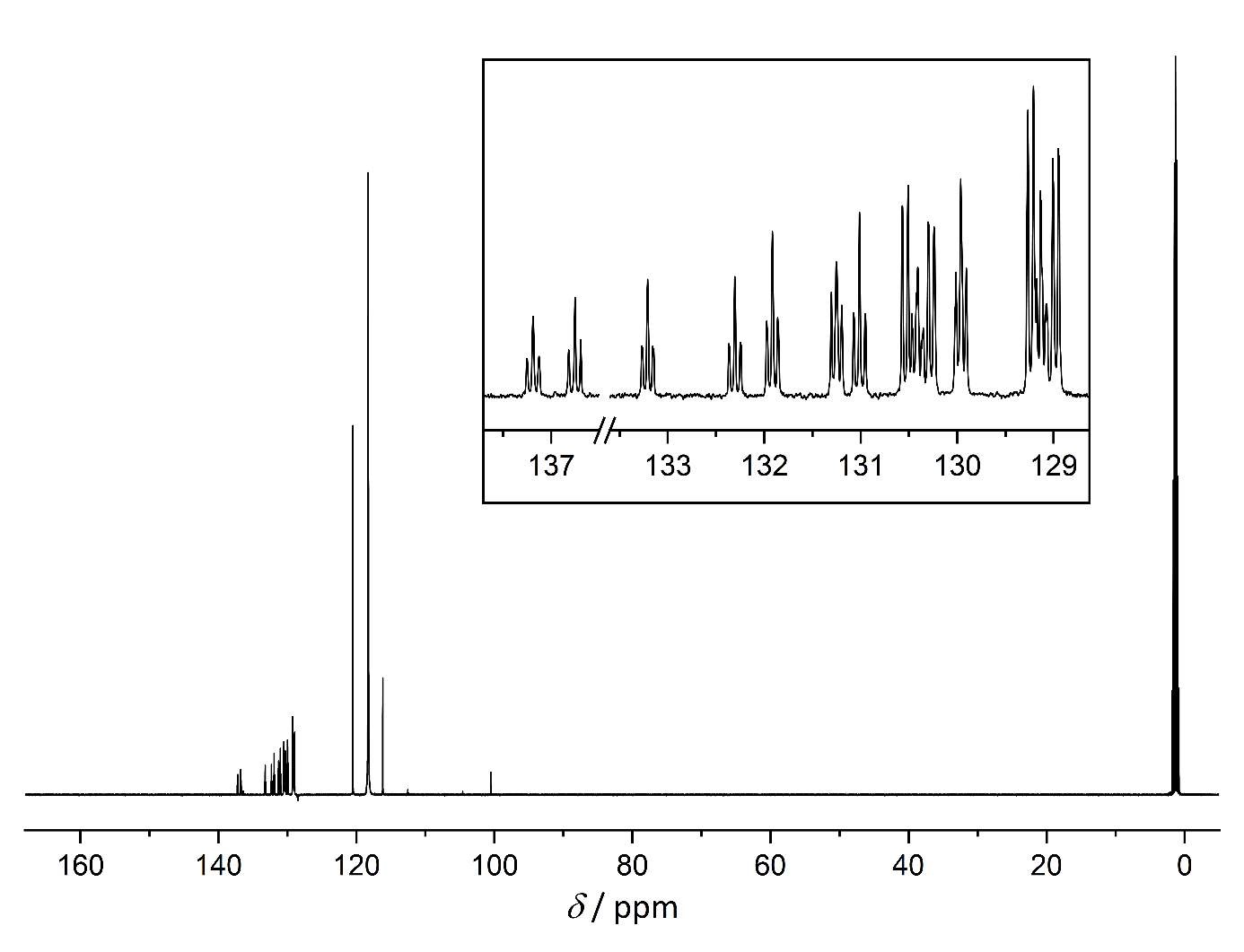


Fig. S31. ^13^C{^19^F} NMR spectrum of **4**.


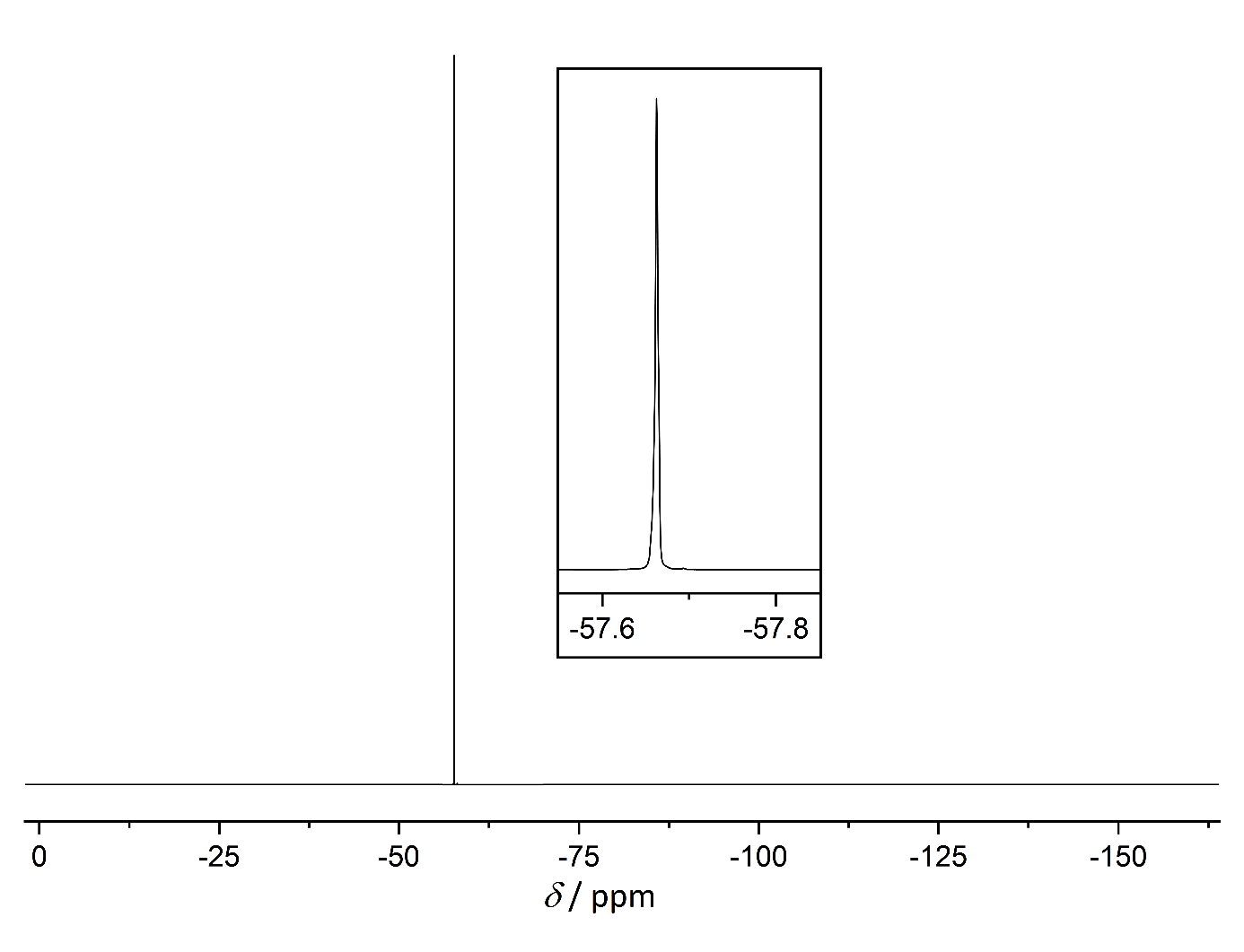


Fig. S32. ^19^F NMR spectrum of **4**.


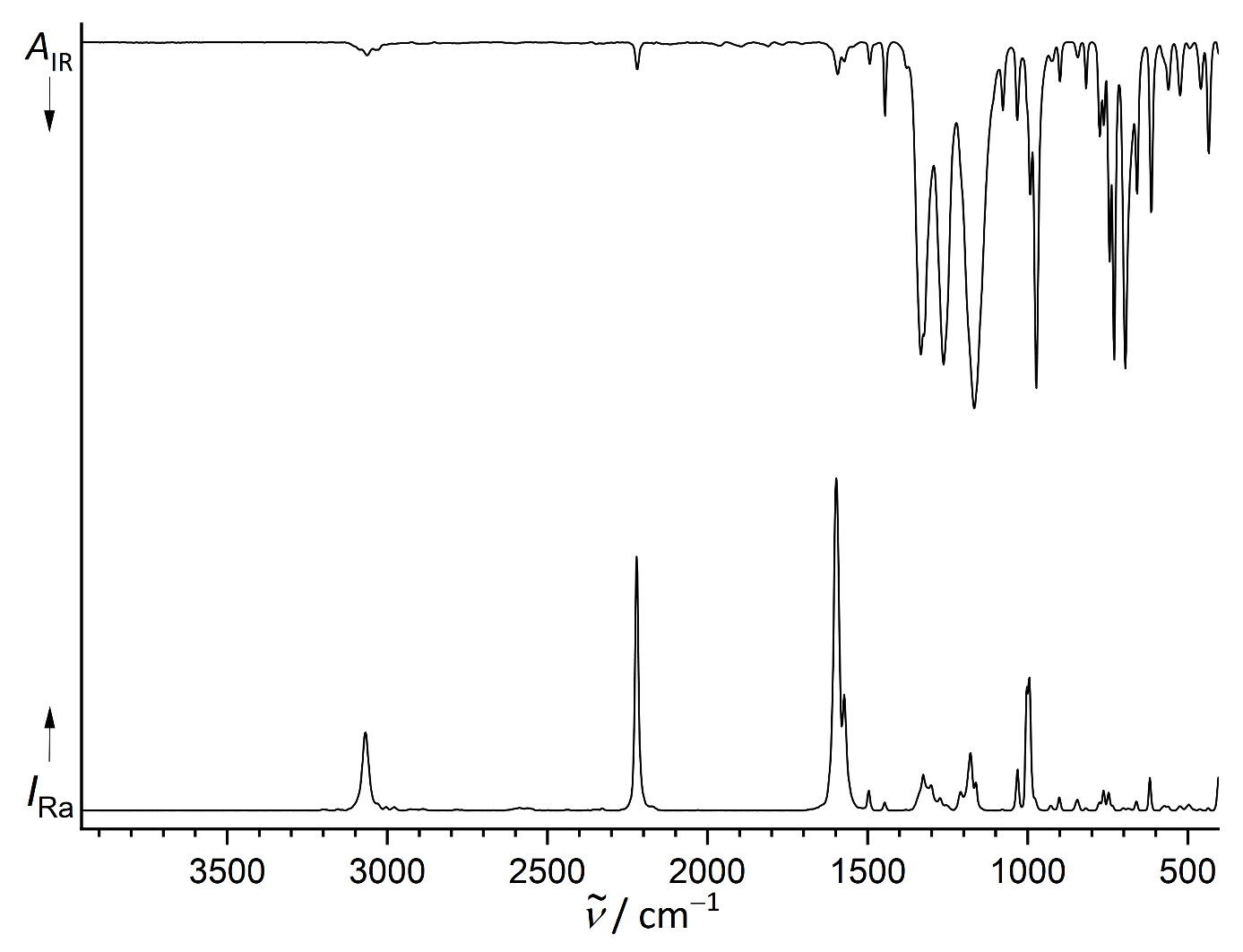


Fig. S33. IR (top) and Raman spectrum (bottom) of **4**.


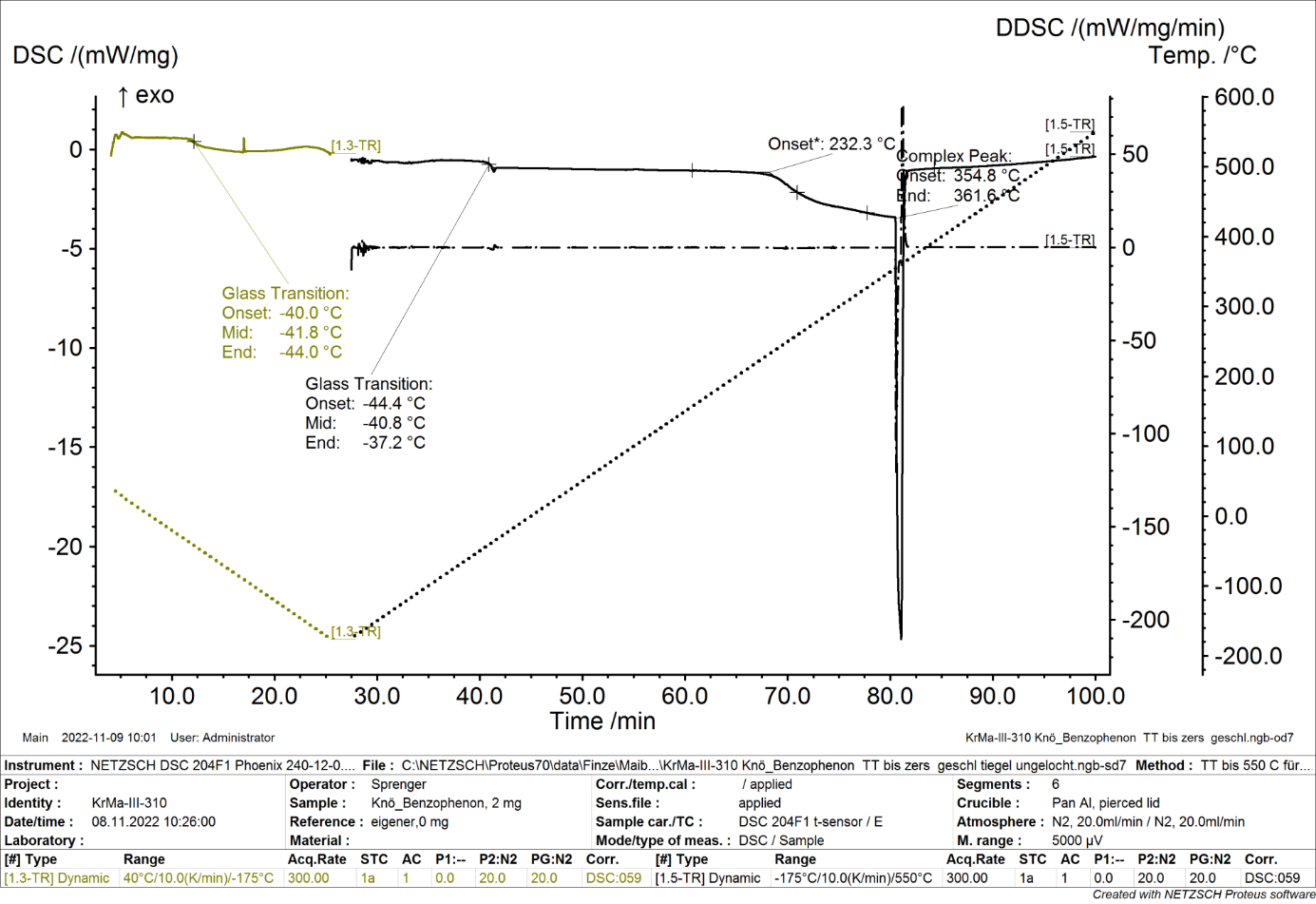


Fig. S34. DSC curve of **4**.

**2-*N*,*N*-Bis(trifluoromethyl)amino-3,3-bis(4-fluorphenyl)acrylonitrile (5)**

A solution of *N,N*-bis*(*trifluoromethyl)aminoacetonitrile (722 mg, 3.76 mmol; **1**) in THF (2 mL) was added to *n*-butyllithium (1.5 mL, 3.75 mmol, 2.5 M in hexane) in THF (5 mL) at −78 °C. After ten minutes, a solution of 4,4 ́-difluorobenzophenone (818 mg, 3.75 mmol) in THF (2 mL) was added and the reaction mixture was stirred at room temperature for seven days. The solvent was removed in a vacuum and 2-*N,N*-bis(trifluoromethyl)amino-3,3-bis(4-fluorophenyl)­acrylonitrile (**5**) was obtained by column chromatography (DCM : hexane 15 : 85) as a colorless liquid that crystallized within one day. Yield: 970 mg (2.47 mmol, 66% based on **1**).

Elemental analysis: calculated (%) for C_17_H_8_F_8_N_2_, C 52.06, H 2.06, N 7.14; found, C 52.11, H 2.21, N 7.07.

HRMS (ASAP+) m/z, calculated for C_17_H_9_F_8_N_2_^+^: 393.0633 (100%), 394.0667 (18.4%), 395.0700 (1.6%); found: 393.0617 (100%), 394.0655 (17.4%), 395.0690 (1.5%).

^1^H NMR (500 MHz, CD_3_CN): *δ* = 7.55─7.50 (m, 2H, *p*F*Ph*-*H*), 7.29─7.19 ppm (m, 6H, *p*F*Ph*-*H*).

^13^C{^1^H} NMR (126 MHz, CD_3_CN): *δ* = 166.5 (sept, 1C, ^3^*J*_F,C_ = 0.9 Hz, NC─C─*C*), 165.6 (d, 1C, ^1^*J*_F,C_ = 251 Hz, *p*F*Ph*-*C*─F), 164.9 (d, 1C, ^1^*J*_F,C_ = 250 Hz, *p*F*Ph*-*C*─F), 133.5 (d, 2C, *J*_F,C_ = 9.2 Hz, *p*F*Ph-C*), 133.2 (d, 1C, ^4^*J*_F,C_ = 3.1 Hz, *p*F*Ph*-*C*_ipso_), 132.8 (d, 1C, ^4^*J*_F,C_ = 3.4 Hz, *p*F*Ph*-*C*_ipso_), 132.6 (d, 2C, *J*_F,C_ = 9.0 Hz, *p*F*Ph*-*C*), 120.5 (qm, 2C, ^1^*J*_F,C_ = 266 Hz, *C*F_3_), 117.2─116.7 (m, 4C, *p*F*Ph*-*C*), 116.1─116.0 (m, 1C, *C*≡N or NC─*C*), 100.6─100.5 ppm (m, 1C, *C*≡N or NC─*C*).

^13^C{^19^F} NMR (126 MHz, CD_3_CN): *δ* = 166.5─166.5 (m, 1C, NC─C─*C*), 165.7─165.4 (m, 1C, *p*F*Ph*-*C*─F), 165.1─164.8 (m, 1C, *p*F*Ph*-*C*─F), 133.5 (dd, 2C, ^1^*J*_C,H_ = 164 Hz, ^2^*J*_C,H_ = 7.4 Hz, *p*F*Ph*-*C*), 133.3─133.2 (m, 1C, *p*F*Ph*-*C*_ipso_), 132.9─132.8 (m, 1C, *p*F*Ph*-*C*_ipso_), 132.6 (dd, 2C, ^1^*J*_C,H_ = 165, ^2^*J*_C,H_ = 7.1 Hz, *p*F*Ph*-*C*), 120.5 (s, 2C, *C*F_3_), 116.9 (dm, 4C, ^1^*J*_C,H_ = 165 Hz, *p*F*Ph*-*C*), 116.1 (s, 1C, *C*≡N or NC─*C*), 100.6─100.5 ppm (m, 1C, *C*≡N or NC─*C*).

^15^N NMR (^15^N-^19^F HMBC, CD_3_CN): *δ* = −285.4 ppm (s, 1N, *N*(CF_3_)_2_).

^19^F NMR (471 MHz, CD_3_CN): *δ* = −57.7 (s, 6F, C*F*_3_), −109.3 (tt, 1F, ^3^*J*_F,H_ = 8.7, ^4^*J*_F,H_ = 5.3 Hz, *p*F*Ph‑F*), −110.7 ppm (tt, 1F, ^3^*J*_F,H_ = 8.6, ^4^*J*_F,H_ = 5.4 Hz, *p*F*Ph‑F*).


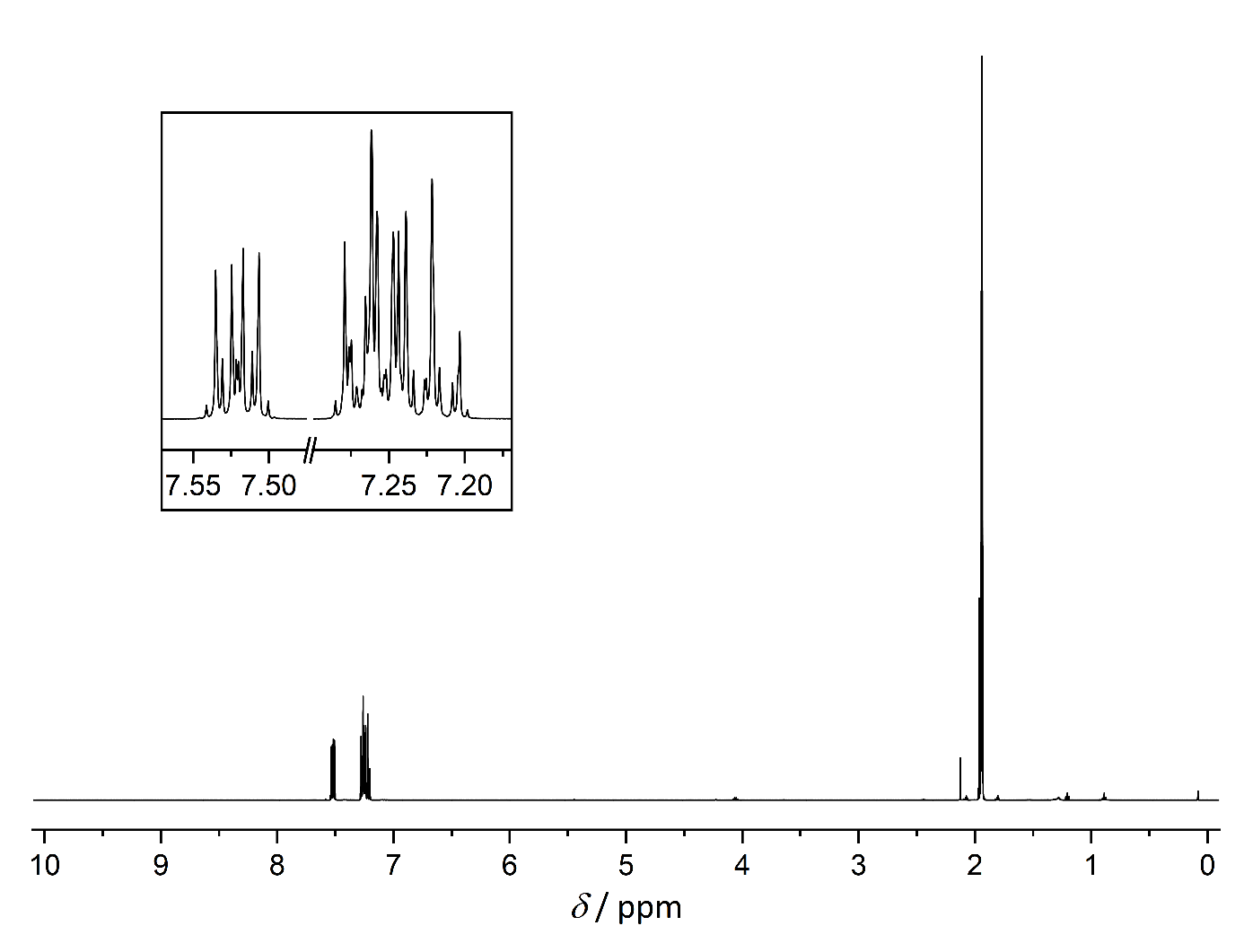


Fig. S35. ^1^H NMR spectrum of **5**.


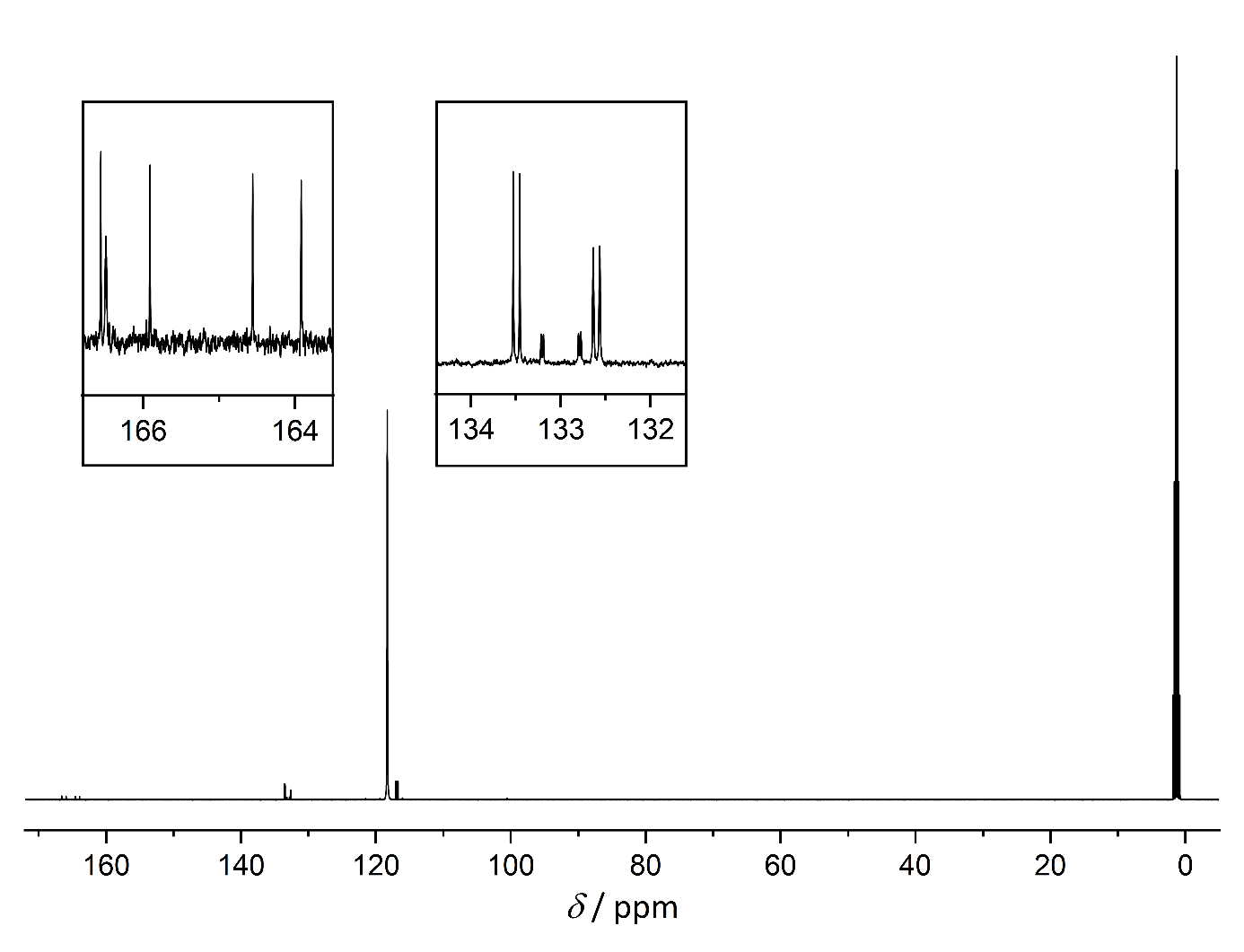


Fig. S36. ^13^C{^1^H} NMR spectrum of **5**.


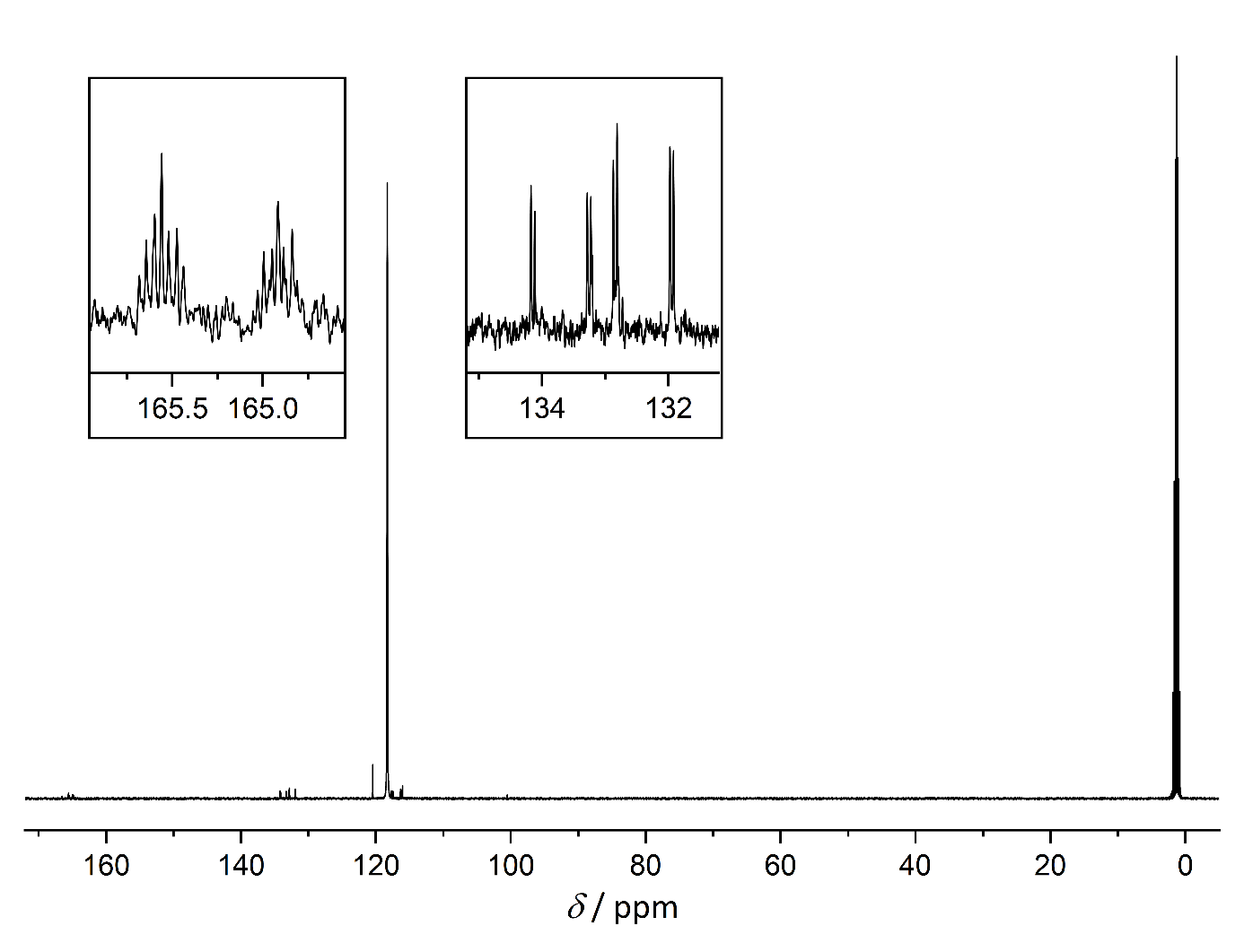


Fig. S37. ^13^C{^19^F} NMR spectrum of **5**.


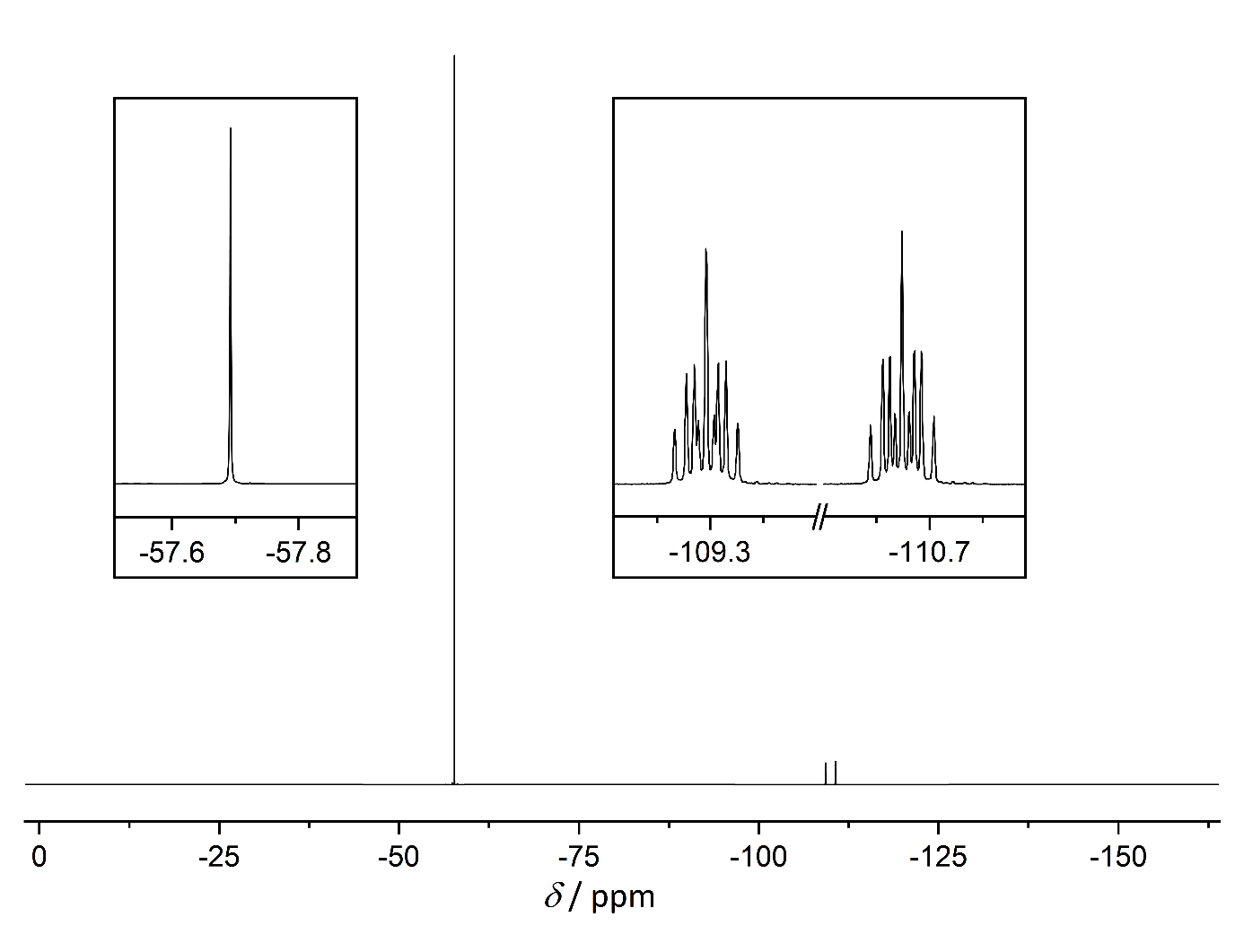


Fig. S38. ^19^F NMR spectrum of **5**.


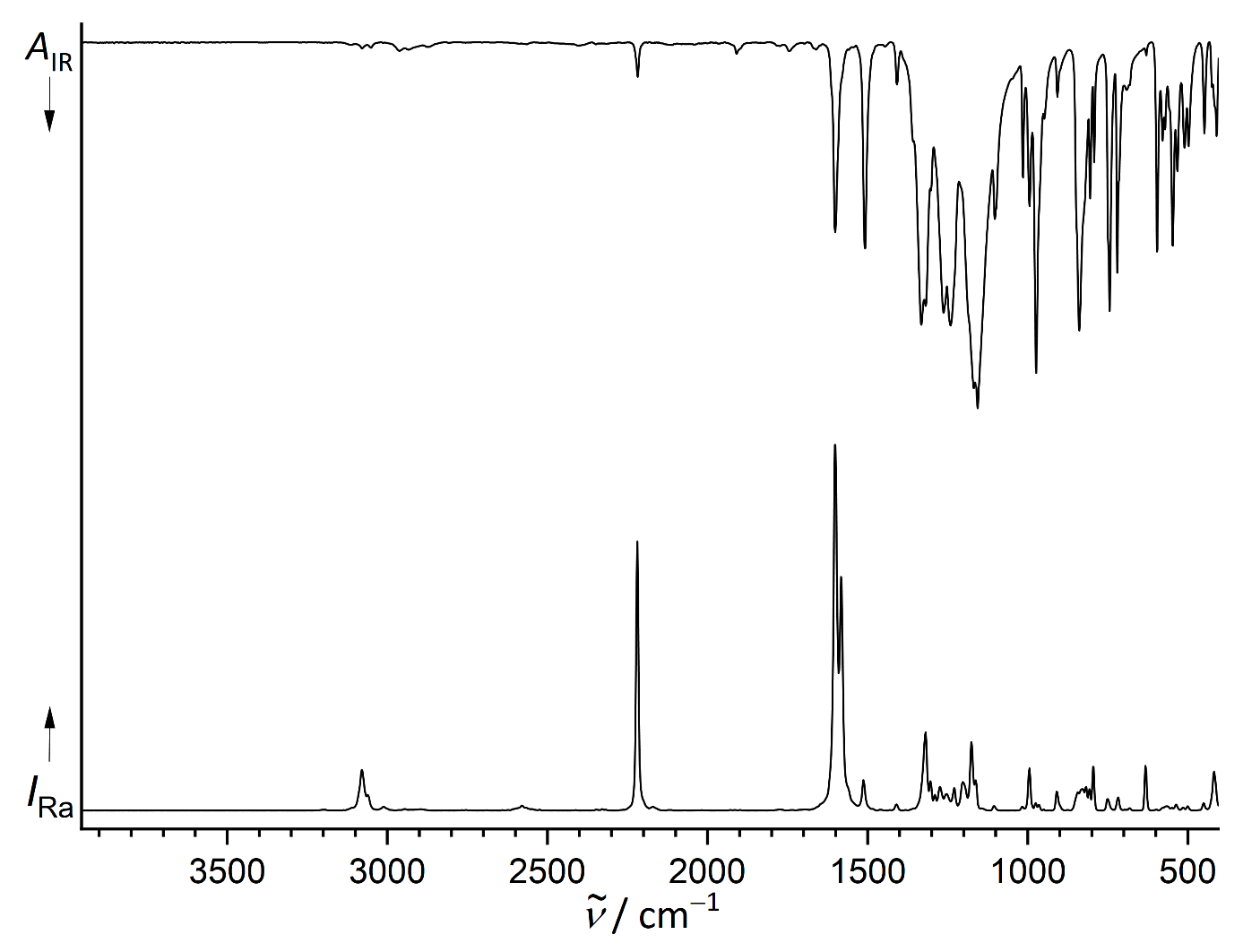


Fig. S39. IR (top) and Raman spectrum (bottom) of **5**.


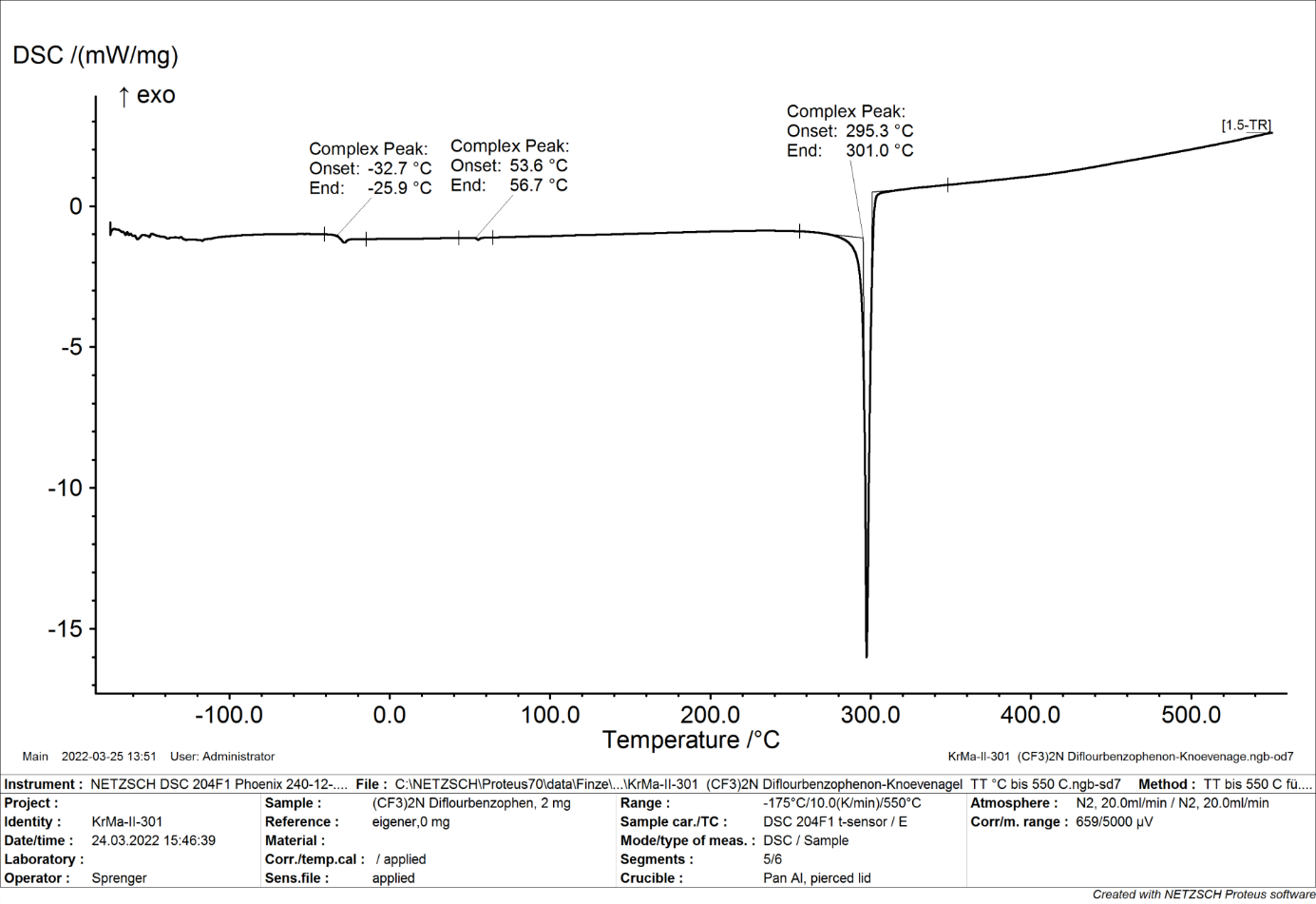


Fig. S40. DSC curve of **5**.

**2-*N*,*N*-Bis(trifluoromethyl)amino-3-(1*H*-indol-3-yl)acrylonitrile (6)**

A solution of *N,N*-bis*(*trifluoromethyl)aminoacetonitrile (907 mg, 4.72 mmol; **1**) in THF (2 mL) was added to a solution of potassium bis(trimethylsilyl)amide (950 mg, 4.76 mmol) in THF (10 mL) at −78 °C. After ten minutes, a solution of 3-(pyrrolidine-1-ylmethylene)-3*H*-indole (930 mg, 4.69 mmol) in THF (10 mL) was added and the reaction solution was stirred at room temperature for three days. The solvent was removed in a vacuum. After column chro­matography (DCM : hexane 1 : 9, 1% NEt_3_) and subsequent sublimation (1 $\cdot$ 10^−3^ mbar, 65 °C), 2-*N,N*-bis(trifluoromethyl)amino-3-(1*H*-indole-3-yl)acrylonitrile (**6**) was obtained as colorless solid consisting of both isomers *E* and *Z*. Yield: 888 mg (2.78 mmol, 59% based on **1**).

Elemental analysis: calculated (%) for C_13_H_7_F_6_N_3_, C 48.92, H 2.21, N 13.16; found, C 49.13, H 2.06, N 13.56.

HRMS (ASAP+) m/z, calculated for C_13_H_8_F_6_N_3_^+^: 320.0617 (100%), 321.0651 (14.1%), 321.0578 (1.1%); found: 320.0608 (100%), 321.0644 (14.2%), 321.0587 (1.0%).

***E* isomer (64%):**

^1^H NMR (500 MHz, CD_3_CN): *δ* = 10.25 (br s, 1H, N*H*), 8.38 (d, 1H, ^3^*J*_H,H_ = 2.7 Hz, *Ind*-*H*^2^), 8.04 (s, 1H, *Ind*─C*H*), 7.84─7.80 (m, 1H, *Ind*-*H*^4^), 7.58─7.54 (m, 1H, *Ind* -*H*^7^), 7.34─7.25 ppm (m, 2H, *Ind*-*H*^5^/-*H*^6^).

^13^C{^1^H} NMR (126 MHz, CD_3_CN): *δ* = 147.1 (sept, 1C, ^4^*J*_F,C_ = 0.9 Hz, *Ind*─*C*H), 137.1 (s, 1C, *Ind*-*C*^3a^), 130.8 (s, 1C, *Ind*-*C*^2^), 128.0 (s, 1C, *Ind*-*C*^7a^), 123.0 (s, 2C, *Ind*-*C*^5^ und *Ind*-*C*^6^), 120.9 (qm, 2C, ^1^*J*_F,C_ = 263 Hz, *C*F_3_), 119.3 (s, 1C, *Ind*‑*C*^4^), 113.6 (s, 1C, *Ind*-*C*^7^), 108.4 (s, 1C, *Ind*-*C*^3^), 93.0─93.0 ppm (m, 1C, *C*≡N or NC─*C*).

^13^C{^19^F} NMR (126 MHz, CD_3_CN): *δ* = 147.2 (d, 1C, ^1^*J*_C,H_ = 157 Hz, *Ind*─*C*H), 137.4─136.9 (m, 1C, *Ind*-*C*^3a^), 131.7─130.0 (m, 1C, *Ind*-*C*^2^), 128.2─127.9 (m, 1C, *Ind*-*C*^7a^), 124.7 (ddd, 1C, ^1^*J*_C,H_ = 160, ^2^*J*_C,H_ = 7.8, ^3^*J*_C,H_ = 1.6 Hz, *Ind*-*C*^5^ or *Ind*-*C*^6^), 123.0 (ddd, 1C, ^1^*J*_C,H_ = 160, ^2^*J*_C,H_ = 7.5, ^3^*J*_C,H_ = 1.1 Hz, *Ind*-*C*^5^ oder *Ind*-*C*^6^), 120.9 (s, 2C, *C*F_3_), 120.0─118.5 (m, 1C, *Ind*‑*C*^4^), 114.3─112.8 (m, 1C, *Ind*-*C*^7^), 108.4 (tm, 1C, ^2^*J*_C,H_ = 6.6 Hz, *Ind*-*C*^3^), 93.0 ppm (dd, 1C, ^2^*J*_C,H_ = 5.6, ^4^*J*_C,H_ = 0.7 Hz, *C*≡N or NC─*C*).

^15^N NMR (^15^N-^1^H HMBC, CD_3_CN): *δ* = −236.3 ppm (s, 1N, *N*─H).

^15^N NMR (^15^N-^19^F HMBC, CD_3_CN): *δ* = −284.2 ppm (s, 1N, *N*(CF_3_)_2_).

^19^F NMR (471 MHz, CD_3_CN): *δ* = −57.6 ppm (s, 6F, C*F*_3_).

***Z* isomer (36%):**

^1^H NMR (500 MHz, CD_3_CN): *δ* = 10.25 (br s, 1H, N*H*), 8.07 (d, 1H, ^3^*J*_H,H_ = 2.7 Hz, *Ind*-*H*^2^), 8.06 (s, 1H, *Ind*─C*H*), 7.84─7.80 (m, 1H, *Ind*-*H*^4^), 7.58─7.54 (m, 1H, *Ind*-*H*^7^), 7.34─7.25 ppm (m, 2H, *Ind*-*H*^5^/-*H*^6^).

^13^C{^1^H} NMR (126 MHz, CD_3_CN): *δ* = 145.7 (sept, 1C, ^4^*J*_F,C_ = 0.8 Hz, *Ind*─*C*H), 136.7 (s, 1C, *Ind*-*C*^3a^), 132.3 (s, 1C, *Ind*-*C*^2^), 128.0 (s, 1C, *Ind*-*C*^7a^), 124.7 (s, 1C, *Ind*-*C*^5^ or -*C*^6^), 123.2 (s, 1C, *Ind*-*C*^5^ or -*C*^6^), 120.9 (qm, 2C, ^1^*J*_F,C_ = 264 Hz, *C*F_3_), 119.2 (s, 1C, *Ind*-*C*^4^), 113.5 (s, 1C, *Ind*-*C*^7^), 109.0 (s, 1C, *Ind*-*C*^3^), 94.1─94.1 ppm (m, 1C, *C*≡N or NC─*C*).

^13^C{^19^F} NMR (126 MHz, CD_3_CN): *δ* = 145.7 (d, 1C, ^1^*J*_C,H_ = 155 Hz, *Ind*─*C*H), 136.9─136.5 (m, 1C, *Ind*-*C*^3a^), 133.2─131.6 (m, 1C, *Ind*-*C*^2^), 128.2─127.9 (m, 1C, *Ind*-*C*^7a^), 124.7 (ddd, 1C, ^1^*J*_C,H_ = 160, ^2^*J*_C,H_ = 7.8, ^3^*J*_C,H_ = 1.6 Hz, *Ind*-*C*^5^ or -*C*^6^), 123.2 (ddd, 1C, ^1^*J*_C,H_ = 160, ^2^*J*_C,H_ = 7.5, ^3^*J*_C,H_ = 1.2 Hz, *Ind*-*C*^5^ or -*C*^6^), 120.9 (s, 2C, *C*F_3_), 120─118.5 (m, 1C, *Ind*-*C*^4^), 114.3─112.8 (m, 1C, *Ind*-*C*^7^), 108.4 (tm, 1C, ^2^*J*_C,H_ = 7.5 Hz, *Ind*-*C*^3^), 94.1 ppm (dd, 1C, ^2^*J*_C,H_ = 2.6, ^4^*J*_C,H_ = 0.7 Hz, *C*≡N or NC─*C*).

^15^N NMR (^15^N-^1^H HMBC, CD_3_CN): *δ* = −234.6 ppm (s, 1N, *N*─H).

^15^N NMR (^15^N-^19^F HMBC, CD_3_CN): *δ* = −288.0 ppm (s, 1N, *N*(CF_3_)_2_).

^19^F NMR (471 MHz, CD_3_CN): *δ* = −58.5 ppm (s, 6F, C*F*_3_).

**
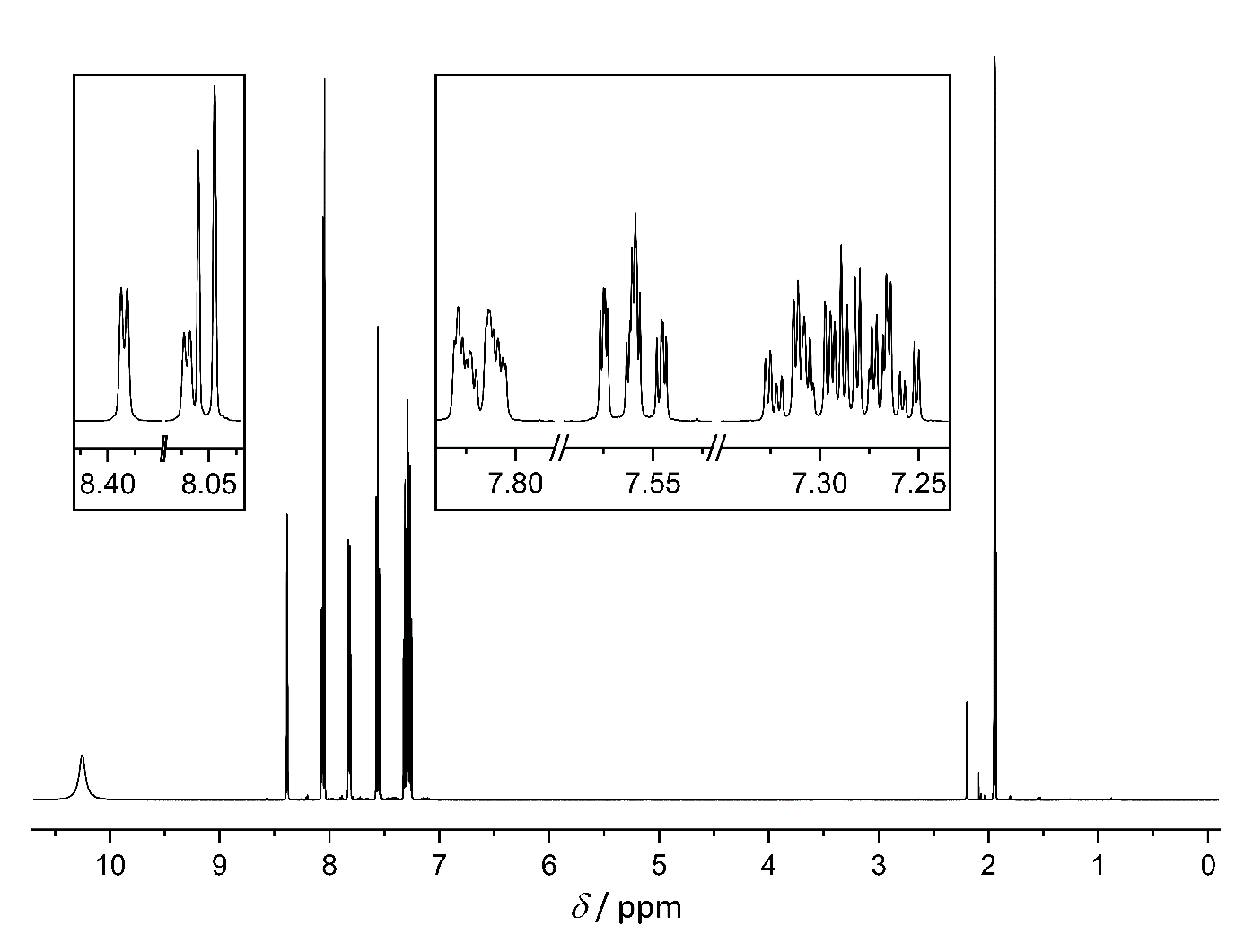
**

Fig. S41. ^1^H NMR spectrum of **6** (mixture of *E* and *Z* isomer).

**
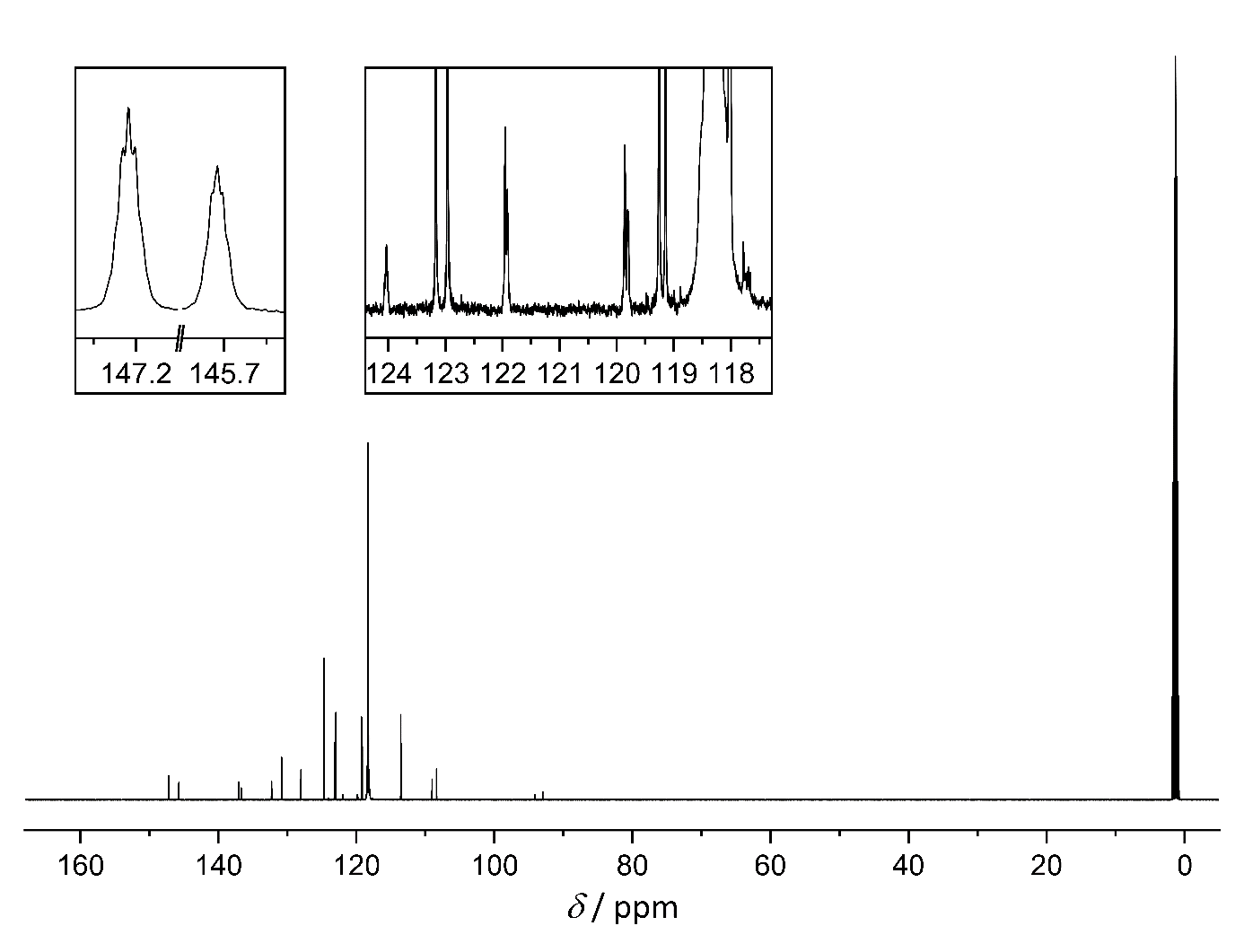
**

Fig. S41. ^13^C{^1^H} NMR spectrum of **6** (mixture of *E* and *Z* isomer).

**
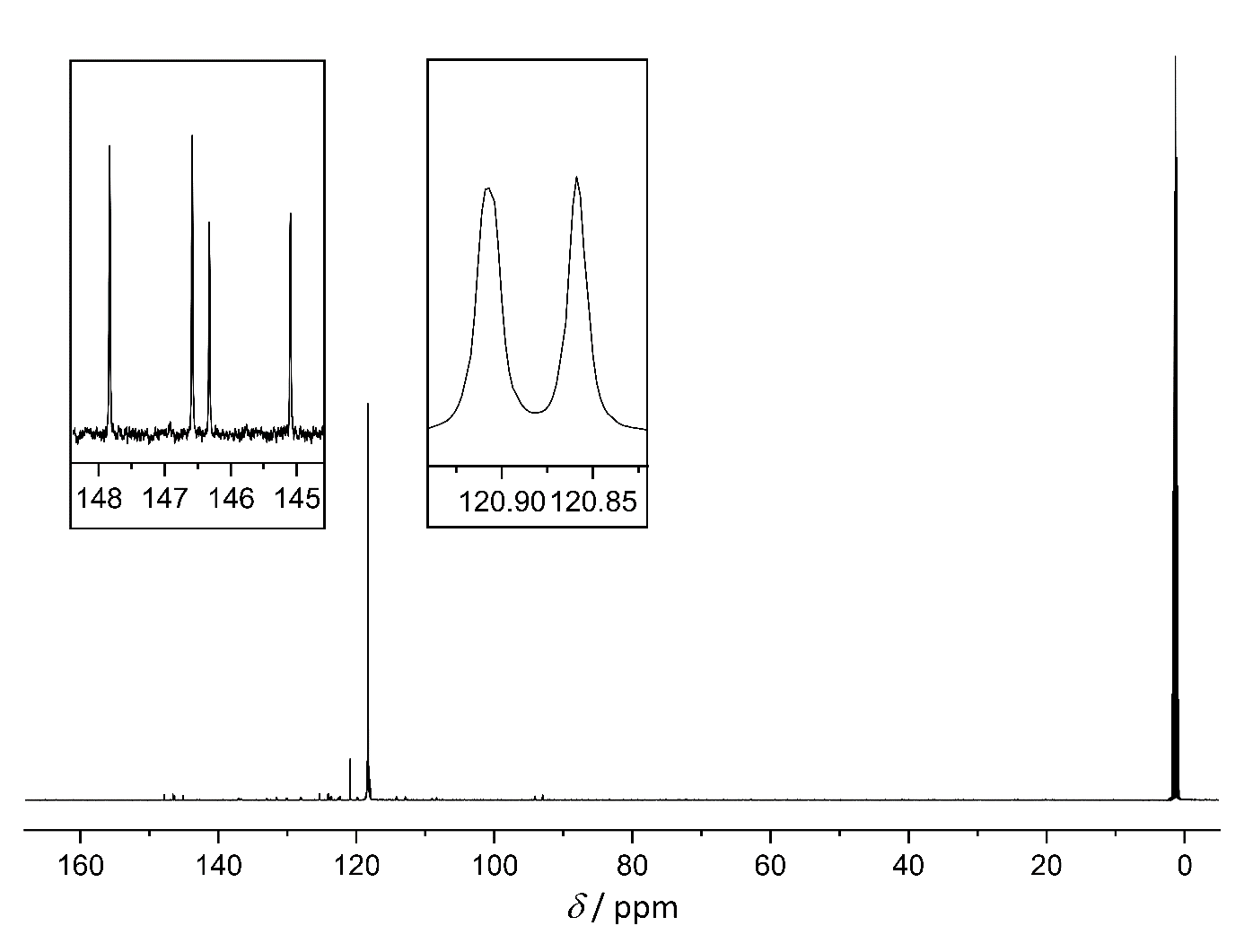
**

Fig. S42. ^13^C{^19^F} NMR spectrum of **6** (mixture of *E* and *Z* isomer).

**
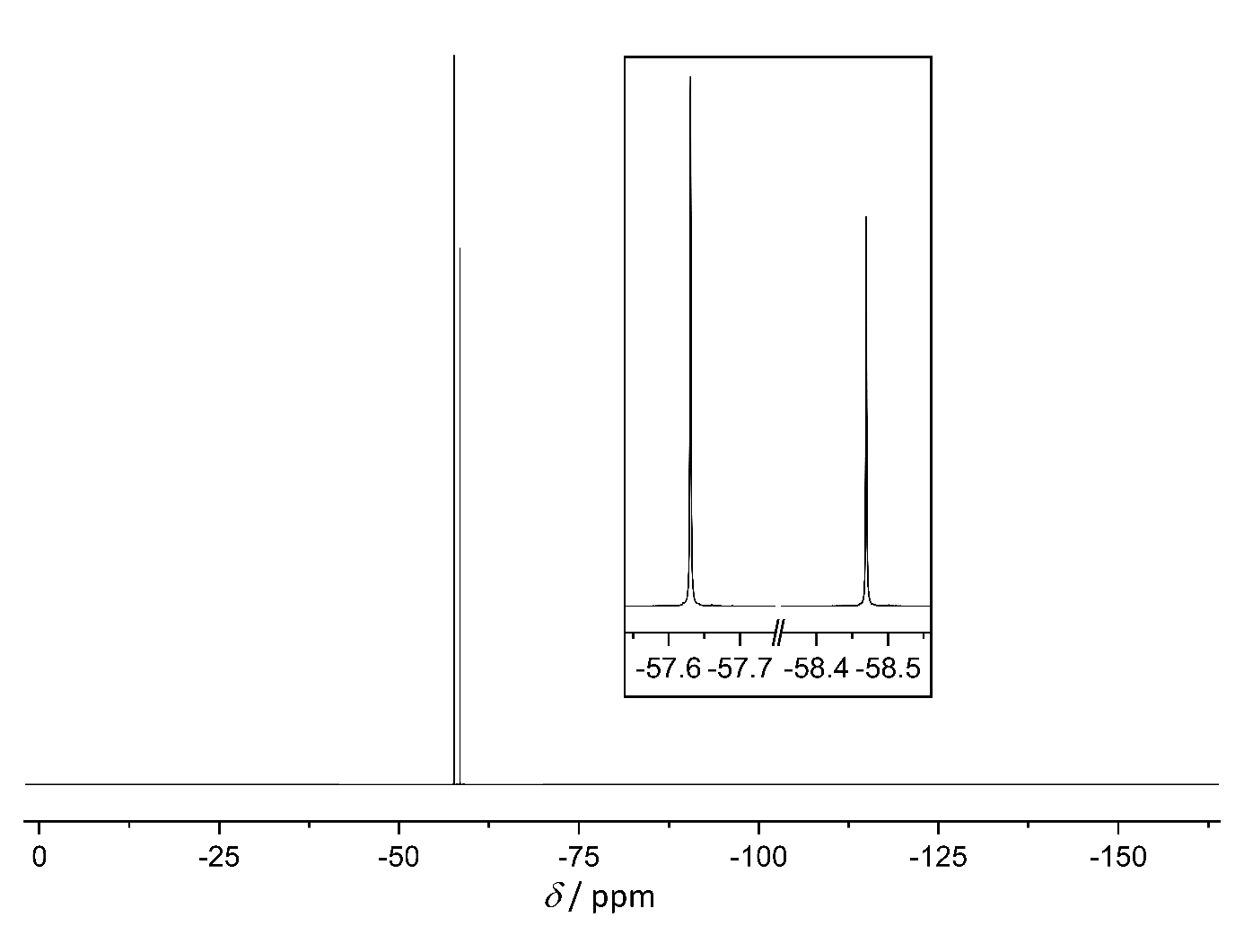
**

Fig. S43. ^19^F NMR spectrum of **6** (mixture of *E* and *Z* isomer).

**
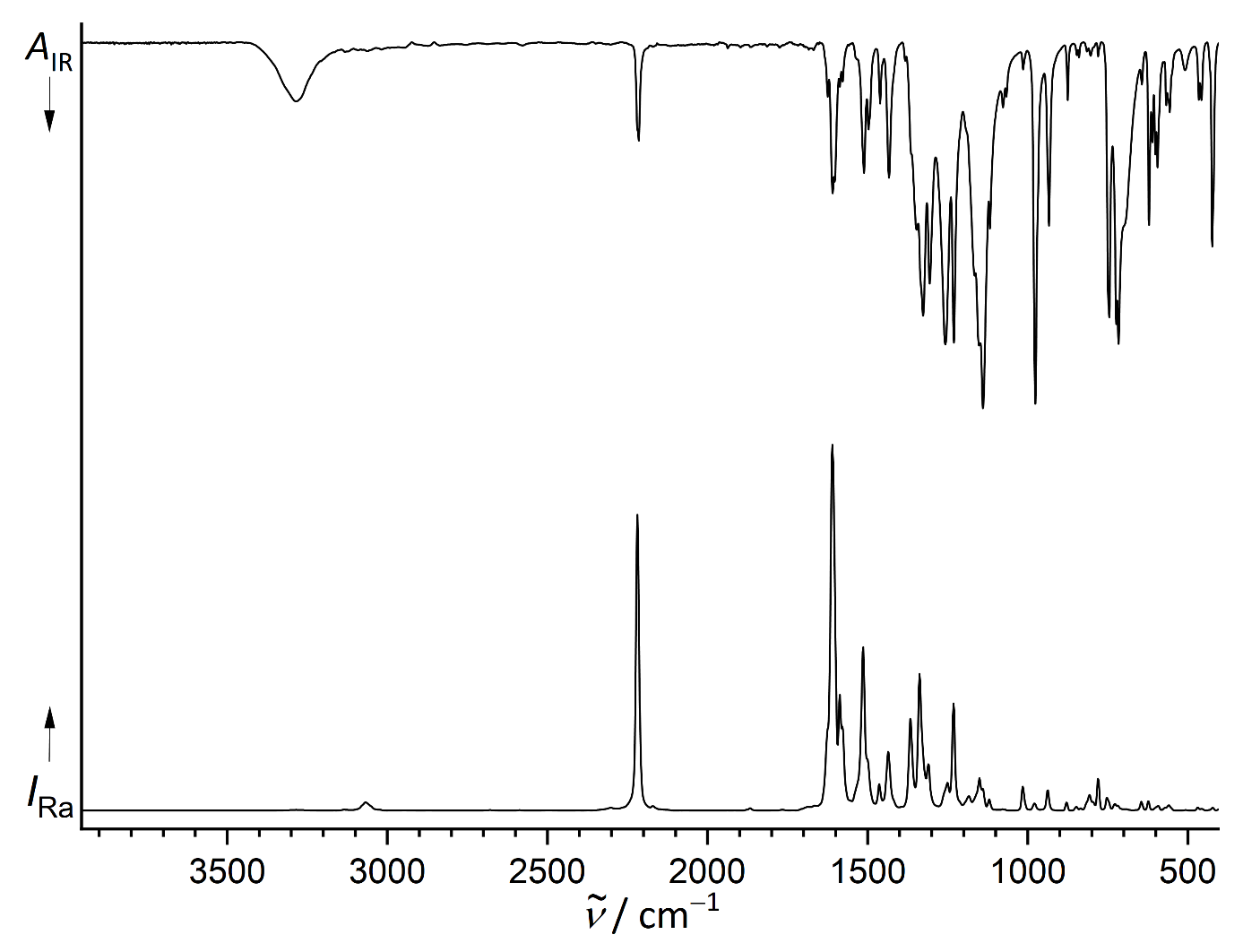
**

Fig. S44. IR (top) and Raman spectrum (bottom) of **6** (mixture of *E* and *Z* isomer).

**
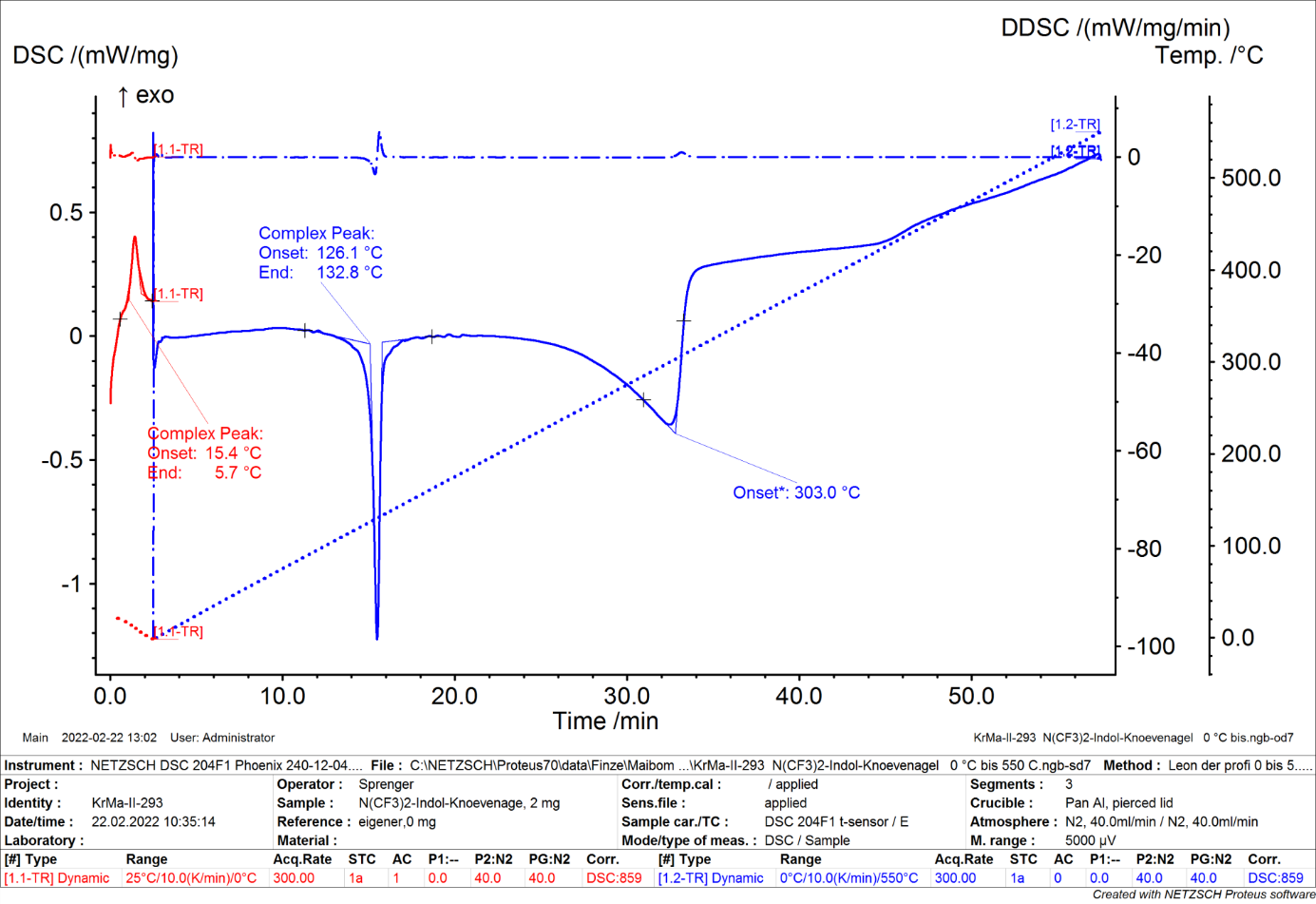
**

Fig. S45. DSC curve of **6** (mixture of *E* and *Z* isomer).

**9-(Cyanoethynyl)anthracene (7)**

2-*N*,*N*-Bis(trifluoromethyl)amino-3-(anthracen-9-yl)acrylonitrile (110 mg, 0.29 mmol; **3**; mixture of *E* and *Z* isomer) and KHMDS (169 mg, 0.85 mmol) were taken up into THF ( 2.0 mL) and stirred at room temperature for 1 d. Dichloromethane (5 mL) was added and the reaction mixture was washed with water (2 mL). The aqueous phase was separated and extracted with dichloromethane (3 x 4 mL). The combined organic phases were washed with brine (5 mL), dried with MgSO_4_, and all volatiles were removed under reduced pressure to yield crude **7**. Column chromatography (silica gel, hexane/DCM 99:1) gave pure **7** as a yellow solid. Yield: 40 mg (0.18 mmol, 62%).

Repeated attempts to get significant elemental analysis failed even with crystalline substance

HRMS (ASAP+) m/z, calculated for C_17_H_10_N ^+^: 228.0808; found: 228.0800.

^1^H NMR (500 MHz, CD_3_CN): *δ* = 8.82 (s, 1H, *Anth*-*H*), 8.48 (ddd, 2H, *J*_H,H_ = 8.8, *J*_H,H_ = 1.0, *J*_H,H_ = 1.0 Hz, *Anth*-*H*), 8.2 (ddd, 2H, *J*_H,H_ = 8.5, *J*_H,H_ = 0.7, *J*_H,H_ = 1.3 Hz *Anth*-*H*), 7.76 (ddd, 2H, *J*_H,H_ = 8.7, *J*_H,H_ = 6.6, *J*_H,H_ = 1.3 Hz, *Anth*-*H*), 7.64 ppm (ddd, 2H, *J*_H,H_ = 8.5, *J*_H,H_ = 6.6*, J*_H,H_ = 1.0 Hz *Anth*-*H*).

^13^C{^1^H} NMR (126 MHz, CD_3_CN): *δ* = 136.0 (s, 2C, *Anth*-*C*), 133.9 (s, 1C, *Anth*-*C*), 131.8 (s, 2C, *Anth*-*C*), 130.5 (s, 2C, *Anth*-*C*), 130.2 (s, 2C, *Anth*-*C*), 127.5 (s, 2C, *Anth*-*C*), 126.2 (s, 2C, *Anth*-*C*), 110.9 (s, 1C, *Anth*-*C*), 106.8 (s, 1C, *C*≡N), 82.0 and 73.9 ppm (s, 2C, *C*≡*C*).


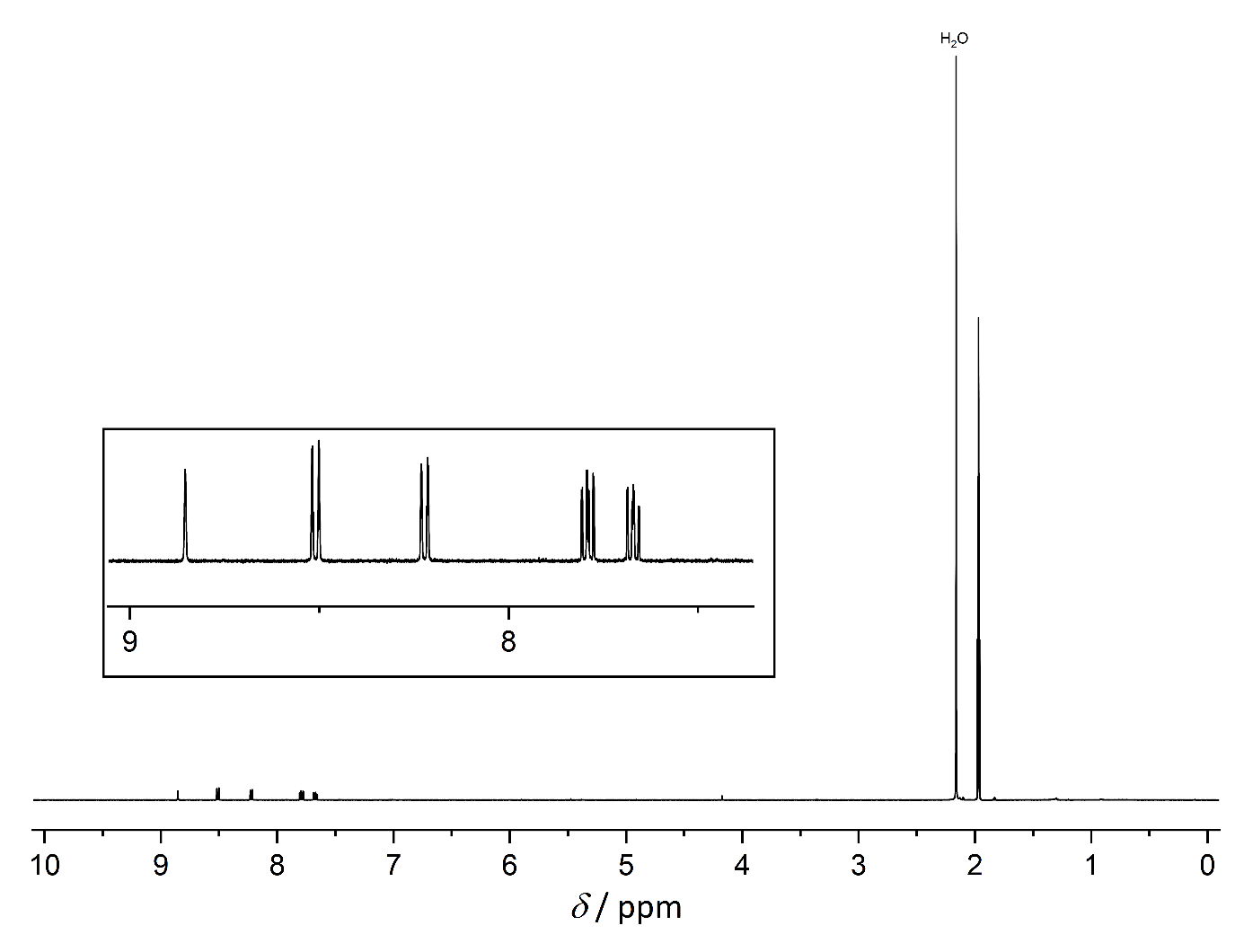


Fig. S46. ^1^H NMR spectrum of **7**.


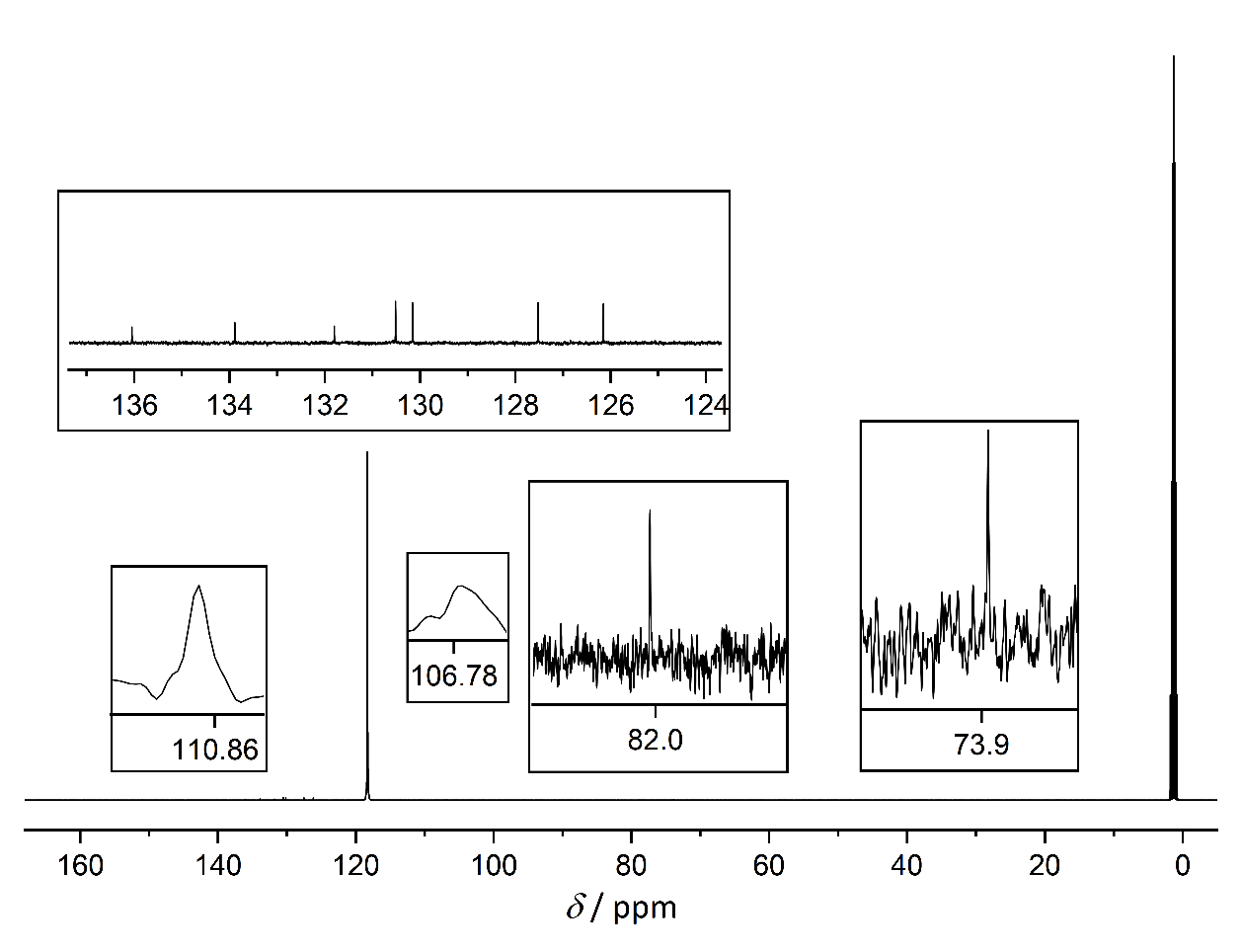


Fig. S47. ^13^C{^1^H} NMR spectrum of **7**.


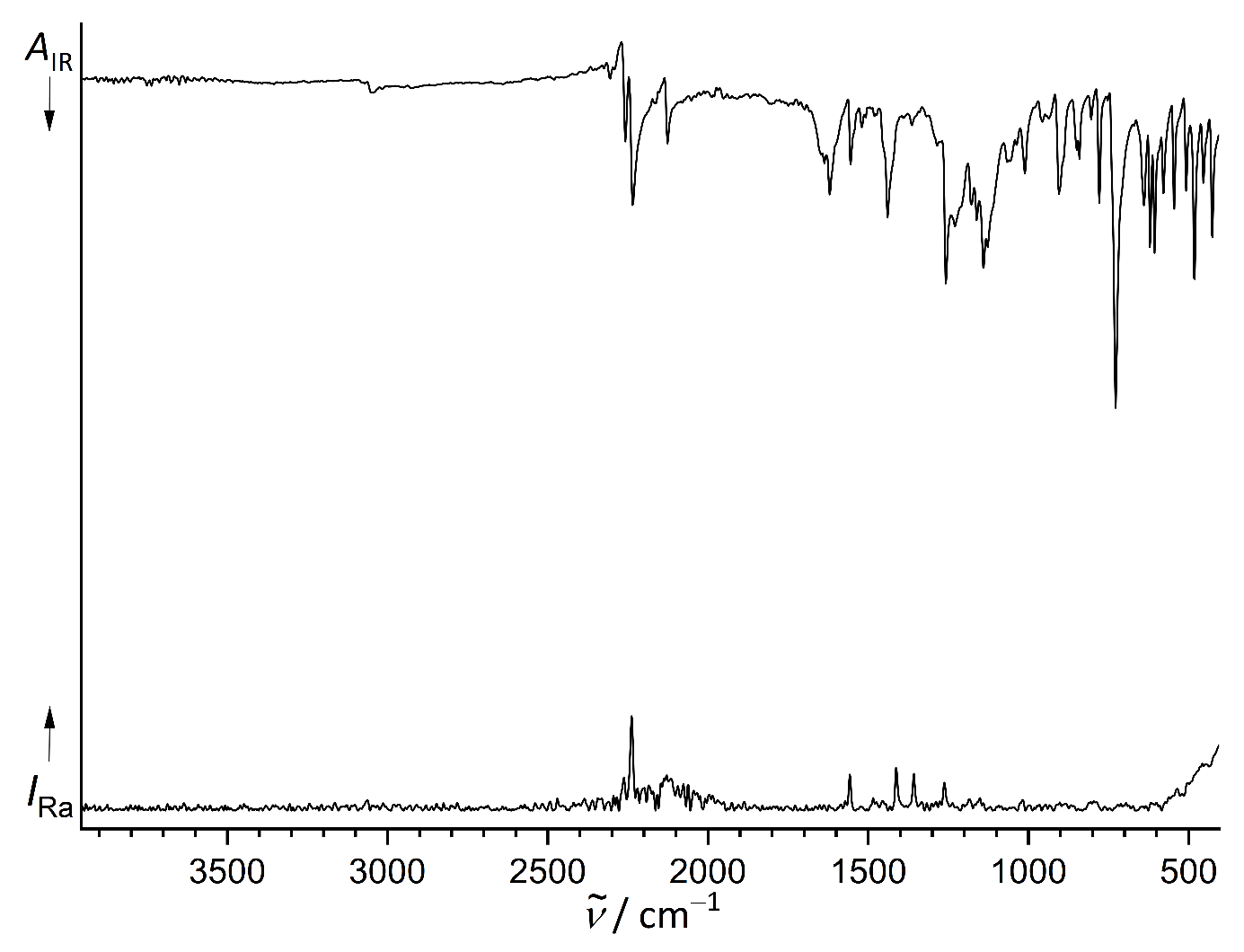


Fig. S48. IR (top) and Raman spectrum (bottom) of **7**.


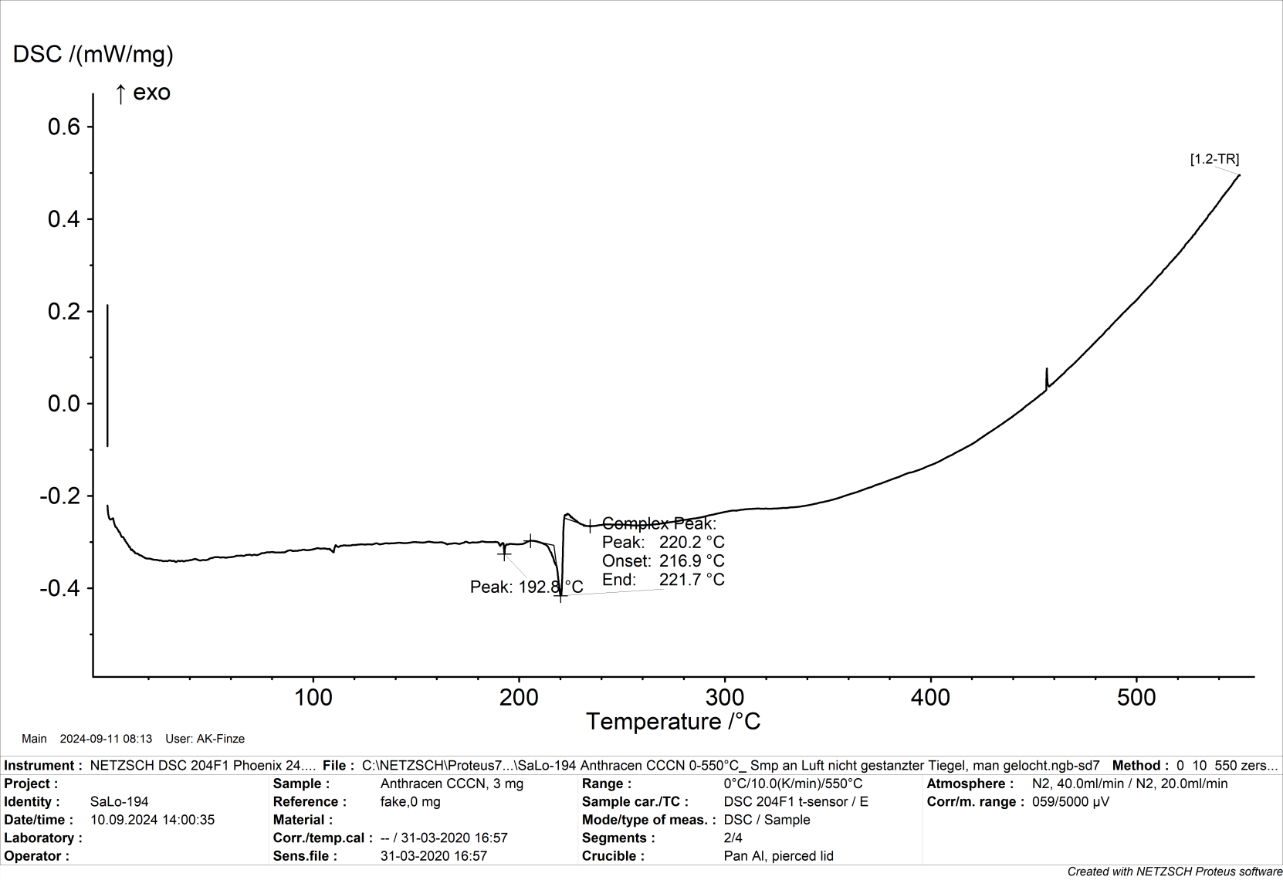


Fig. S49. DSC curve of **7**.

**(4,5-Dihydro-*1H*-imidazol-2-yl)methyl-*N*,*N*-bis(trifluoromethyl)amine (8)**

Ethane-1,2-diamine (14.0 g, 233 mmol) was added to a suspension of elemental sulfur (730 mg, 22 mmol) in *N*,*N*-bis(trifluoromethyl)aminoacetonitrile (10.74 g, 55.9 mmol; **1**) and the mixture was stirred at 130 °C for 1 h. The reaction mixture was cooled to room temperature and the solid material that had formed was filtered off, washed with water (4 x 10 mL), and dried under reduced pressure to give **8** as a colorless solid. Yield: 10,05 g (42.7 mmol, 76%).

Elemental analysis: calculated (%) for C_6_H_7_F_6_N_3_, C 30.65, H 3.00, N 17.87; found, C 30.74, H 2.84, N 17.98.

HRMS (ESI+) m/z, calculated forC_6_H_8_F_6_N_3_^+^: 3236.0617 (100%), 237.0651 (6.5%), 237.0588 (1.1%); found: 236.0610 (100%), 237.0643 (5.5%), 237.0583 (0.9%).

^1^H NMR (500 MHz, CD_3_CN): *δ* = 5.09 (br s, 1H, N*H*), 4.04 (m, 2H, C*H*_2_─N(CF_3_)_2_), 3.50 ppm (s, 4H, C*H*_2_─C*H*_2_).

^13^C{^1^H} NMR (126 MHz, CD_3_CN): *δ* = 162.9 (s, 1C, N=*C*─N), 121.6 (qm, 2C, ^1^*J*_F,C_ = 262 Hz, *C*F_3_), 50.7 (br s, 2C, *C*H_2_─*C*H_2_), 43.0 ppm (s, 1C, *C*H_2_─N(CF_3_)_2_).

^13^C{^19^F} NMR (126 MHz, CD_3_CN): *δ* = 162.9 (dt, 1C, ^2^*J*_C,H_ = 9.3, ^2^*J*_C,H_ = 4.6 Hz, N=*C*─N), 121.6 (t, 2C, ^3^*J*_C,H_ = 4.2 Hz, *C*F_3_), 52.8─48.8 (m, 2C, *C*H_2_─*C*H_2_), 43.0 ppm (t, 1C, ^1^*J*_C,H_ = 143 Hz, *C*H_2_).

^15^N NMR (^19^F-^15^N HMBC, CD_3_CN): *δ* = −300.0 ppm (s, 1N, *N*(CF_3_)_2_).

^19^F NMR (471 MHz, CD_3_CN): *δ* = −58.1 ppm (t, 6F, ^4^*J*_F,H_ 1.3 Hz, C*F*_3_).


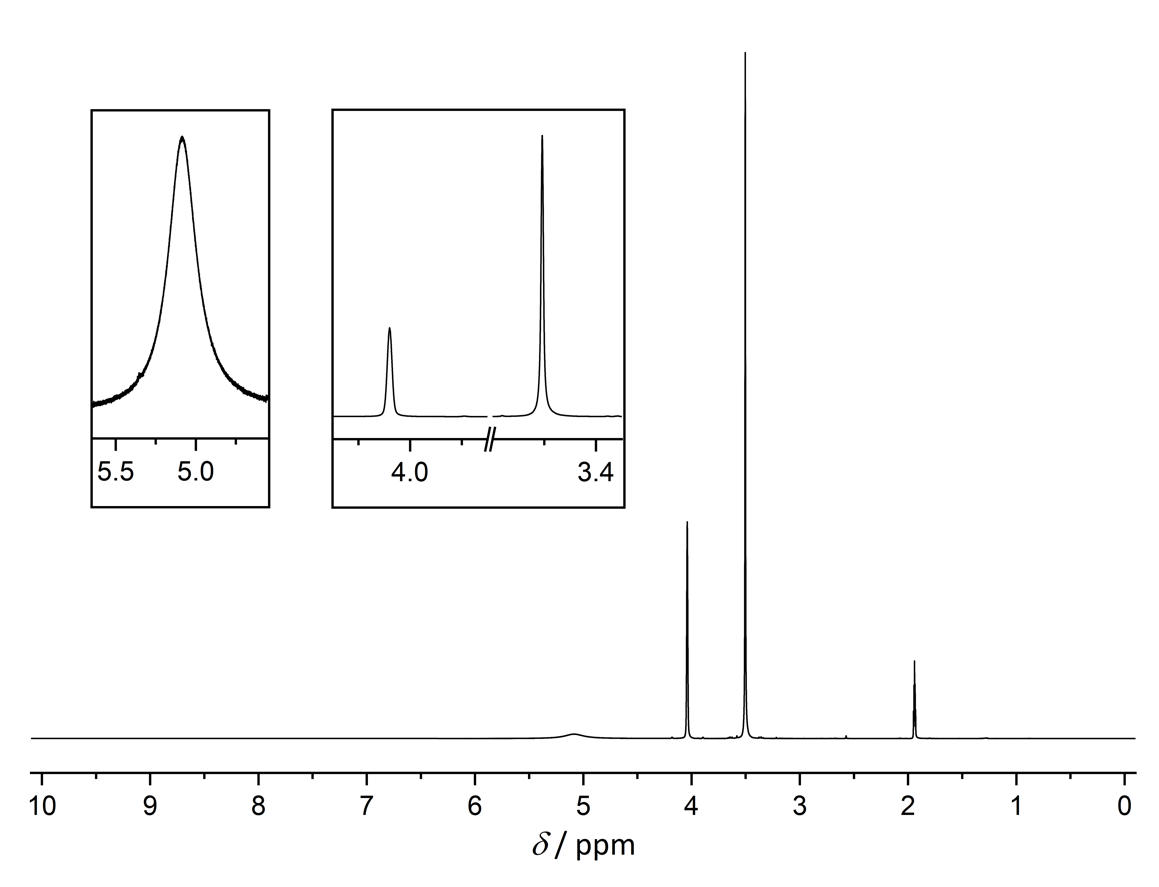


Fig. S50. ^1^H NMR spectrum of **8**.


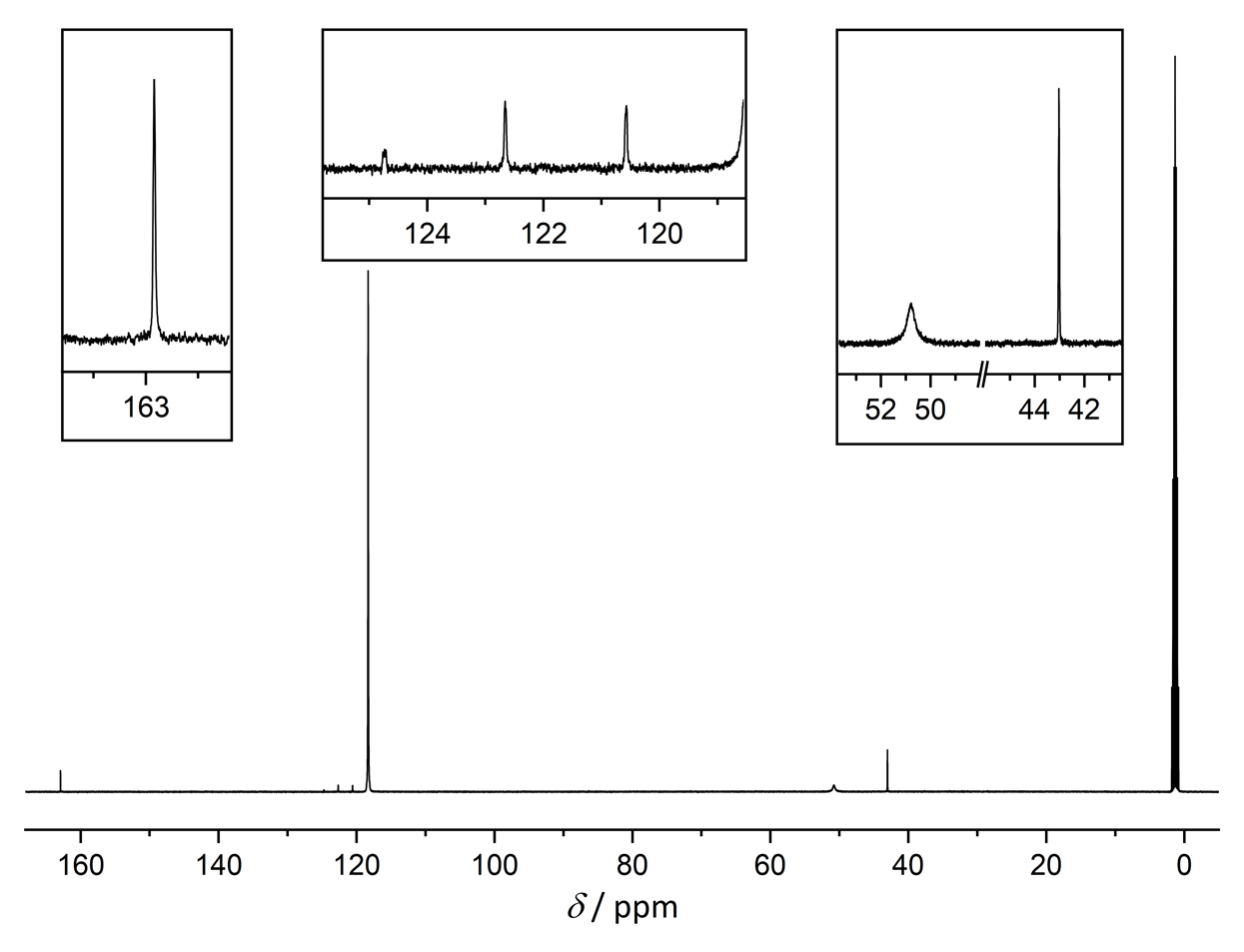


Fig. S51. ^13^C{^1^H} NMR spectrum of **8**.


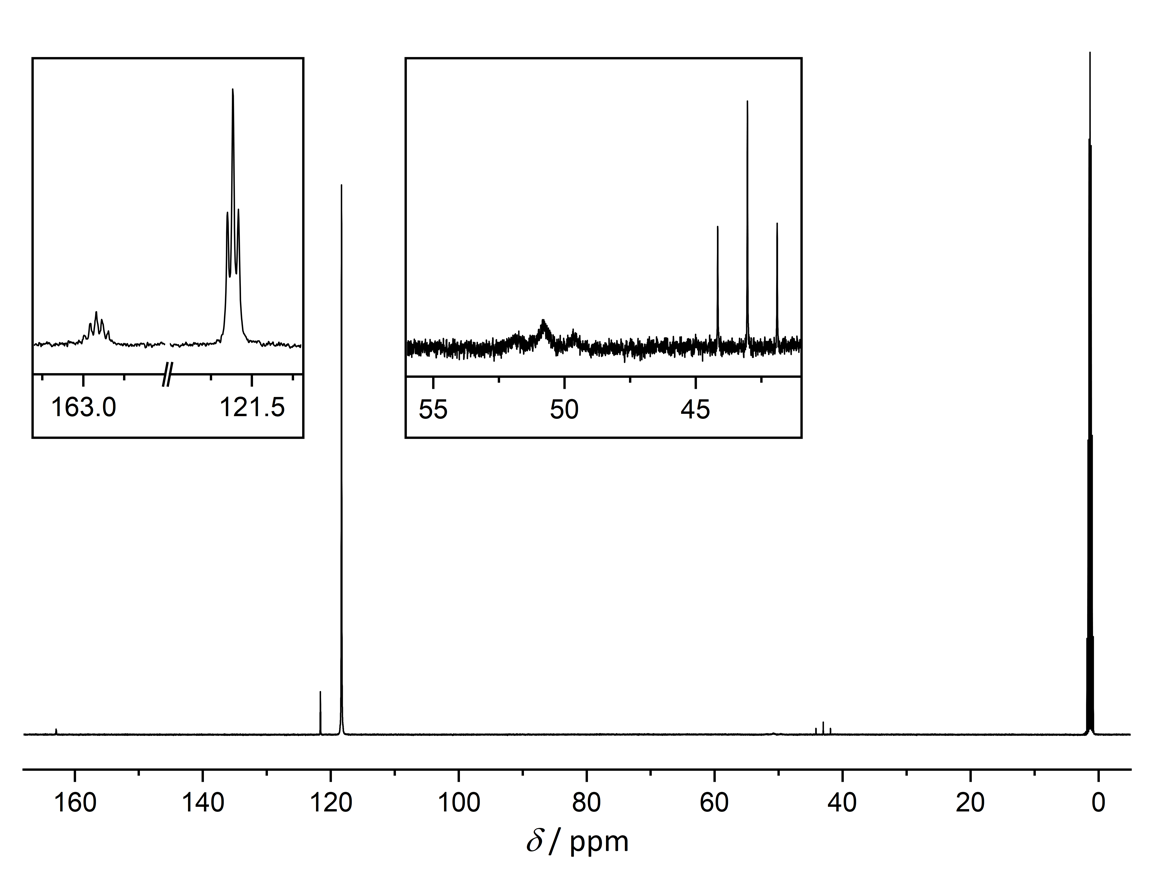


Fig. S52. ^13^C{^19^F} NMR spectrum of **8**.


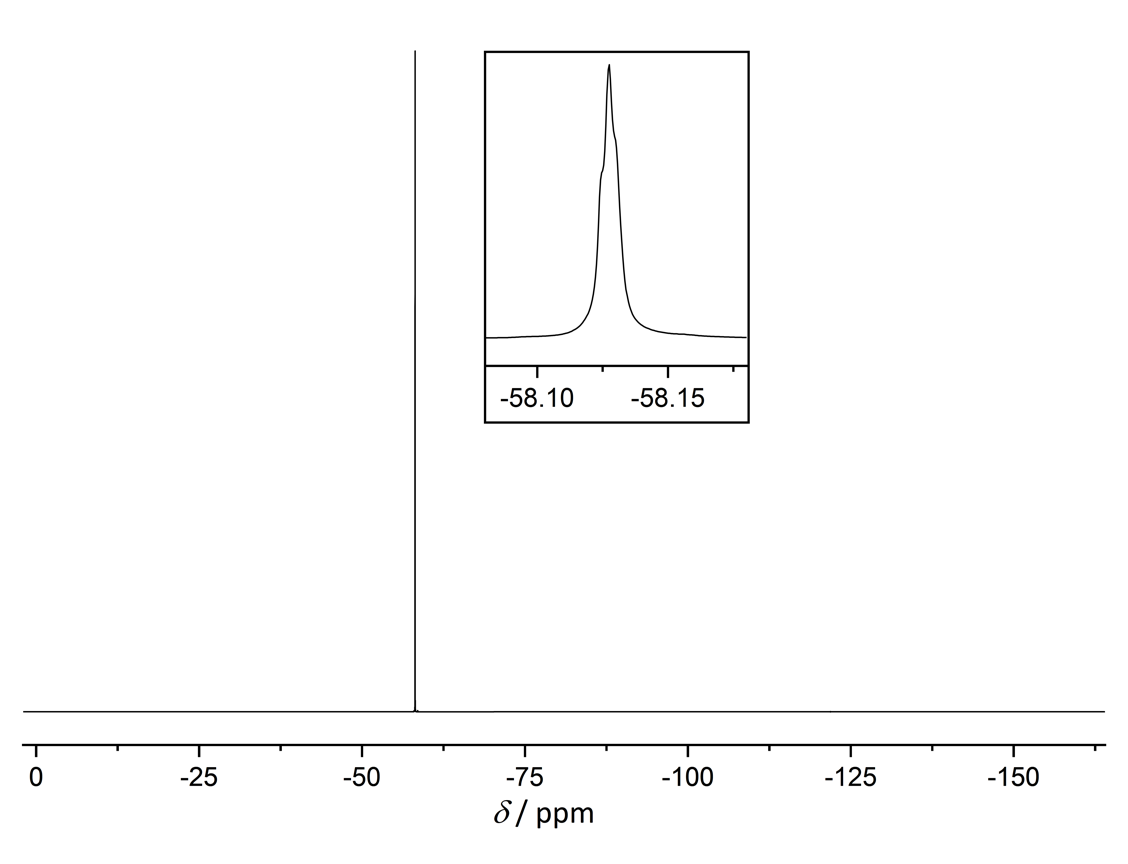


Fig. S53. ^19^F NMR spectrum of **8**.


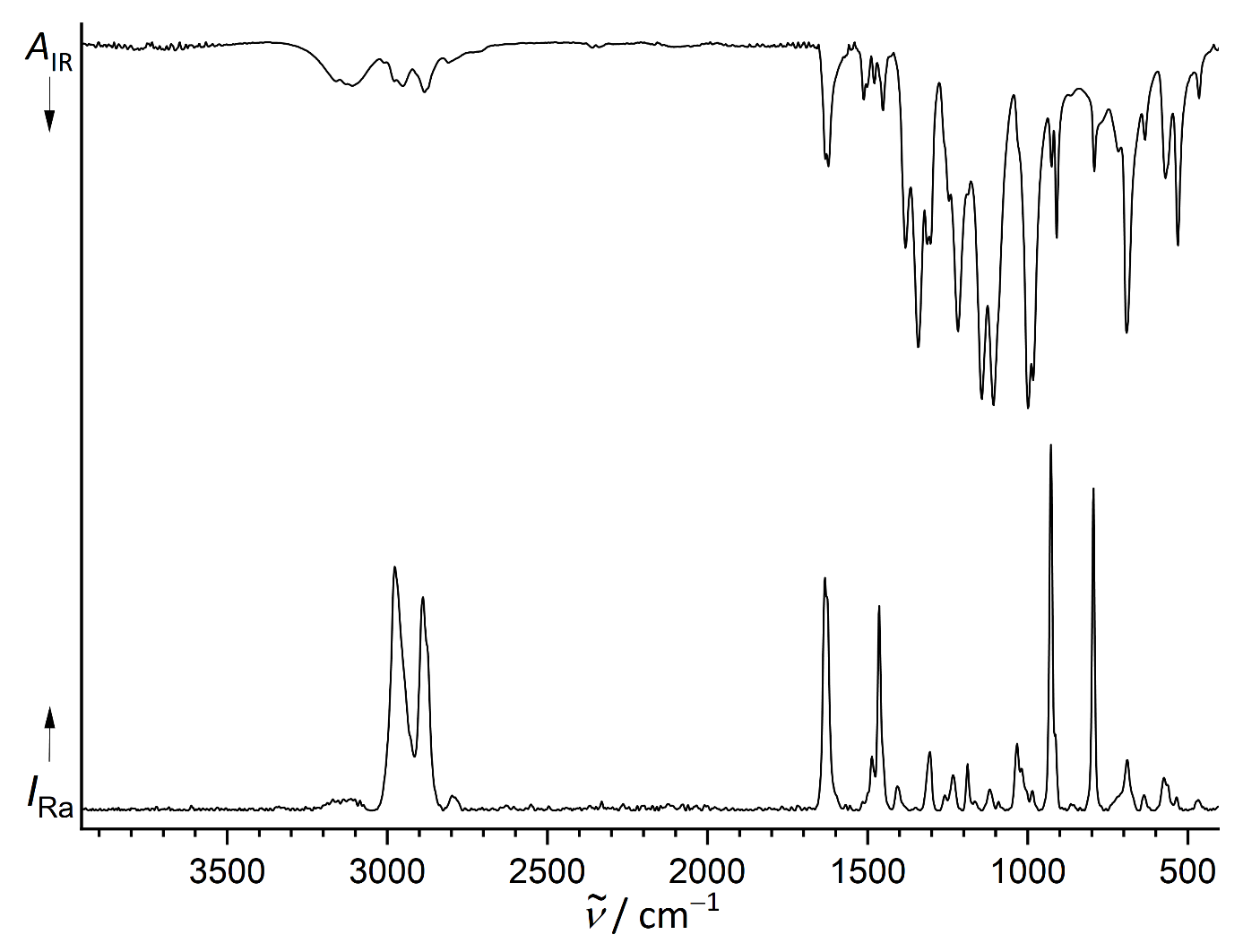


Fig. S54. IR (top) and Raman spectrum (bottom) of **8**.


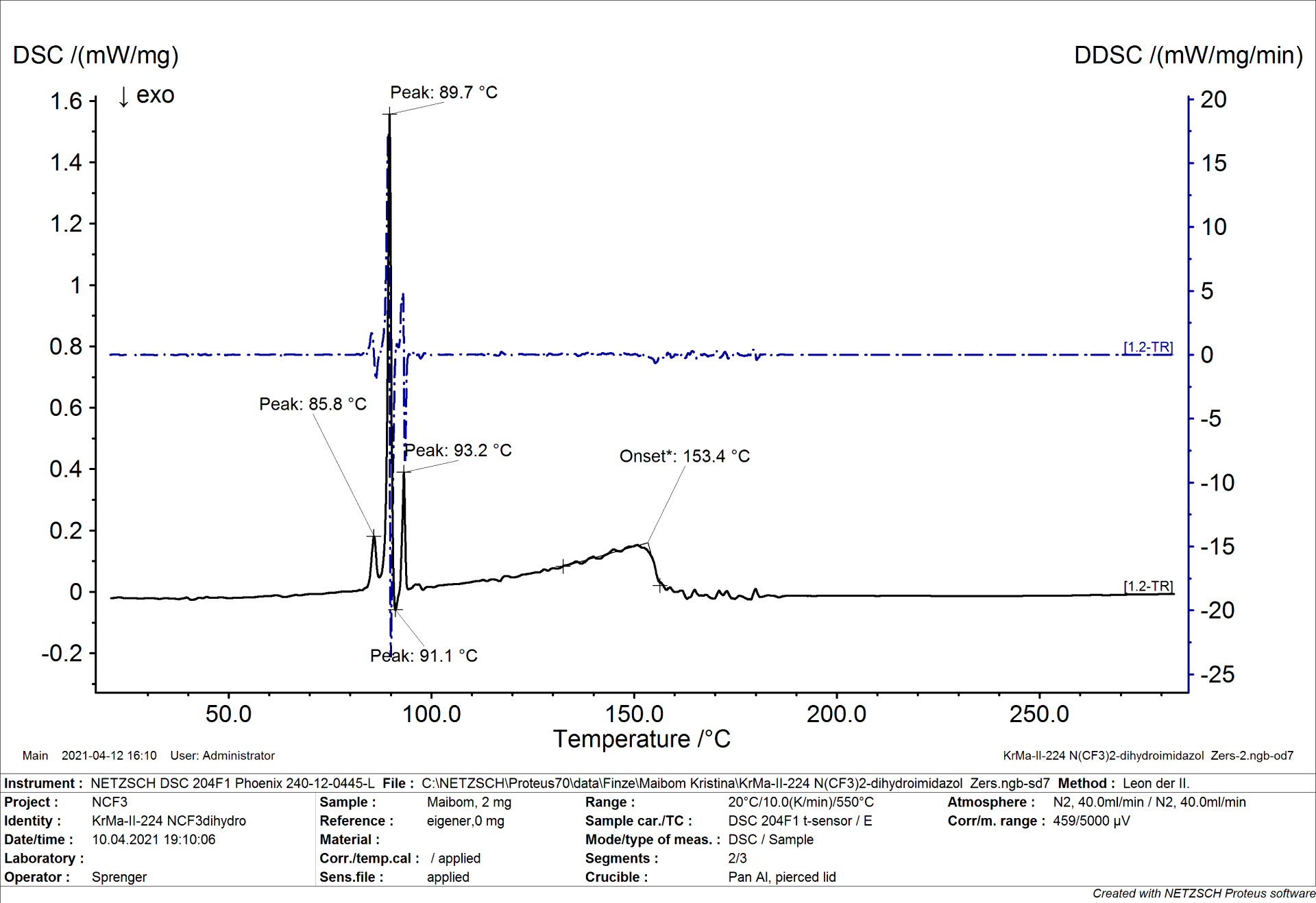


Fig. S55. DSC curve of **8**.

**(1,4,5,6-Tetrahydropyrimidin-2-yl)methyl-*N*,*N*-bis(trifluoromethyl)amine (9)**

Propane-1,3-diamine (967 mg, 13.0 mmol) was added to a suspension of elemental sulfur (41.0 mg, 1.28 mmol) in *N*,*N*-bis(trifluoromethyl)aminoacetonitrile (610 mg, 3.18 mmol; **1**) and the mixture was stirred at 130 °C for 1 h. The reaction mixture was cooled to room temperature and the solid material that had formed was filtered off, washed with water (4 x 1 mL), and dried under reduced pressure to give **9** as a colorless solid. Yield: 451 mg (1.81 mmol, 57%).

Elemental analysis: calculated (%) for C_7_H_9_F_6_N_3_, C 33.74, H 3.64, N 16.87; found, C 33.40, H 3.48, N 16.96.

HRMS (ESI+) m/z, calculated forC_6_H_8_F_6_N_3_^+^: 250.0774 (100%), 251.0807 (7.6%), 251.0744 (1.1%); found: 250.0766 (100%), 251.0800 (7.4%), 251.0741 (1.0%).

^1^H NMR (500 MHz, CD_3_CN): *δ* = 5.11 (br s, 1H, N*H*), 3.80 (m, 2H, C*H*_2_─N(CF_3_)_2_), 3.28─3.15 (m, 4H, C*H*_2_─CH_2_─C*H*_2_), 1.66 ppm (m, 2H, CH_2_─C*H*_2_─CH_2_).

^13^C{^1^H} NMR (126 MHz, CD_3_CN): *δ* = 151.4 (s, 1C, N=*C*─N), 121.8 (qm, 2C, ^1^*J*_F,C_ = 262 Hz, *C*F_3_), 48.0 (s, 1C, *C*H_2_─N(CF_3_)_2_), 42.3 (br s, 2C, *C*H_2_─CH_2_─*C*H_2_), 21.6 ppm (s, 1C, CH_2_─*C*H_2_─CH_2_).

^13^C{^19^F}-NMR (126 MHz, CD_3_CN): *δ* = 151.6─151.3 (m, 1C, N=*C*─N), 121.8 (t, 2C, ^3^*J*_C,H_ = 4.2 Hz, *C*F_3_), 48.0 (t, 1C, ^1^*J*_C,H_ = 142 Hz, *C*H_2_─N(CF_3_)_2_), 42.3 (tm, 2C, ^1^*J*_C,H_ = 136 Hz, *C*H_2_─CH_2_─*C*H_2_), 21.6 ppm (t, 1C, ^1^*J*_C,H_ = 142 Hz, CH_2_─*C*H_2_─CH_2_).

^15^N NMR (^19^F^­^­-^15^N HMBC, CD_3_CN): *δ* = −299.3 ppm (s, 1N, *N*(CF_3_)_2_).

^19^F NMR (471 MHz, CD_3_CN): *δ* = −58.4 ppm (t, 6F, ^4^*J*_F,H_ = 1.4 Hz, C*F*_3_).


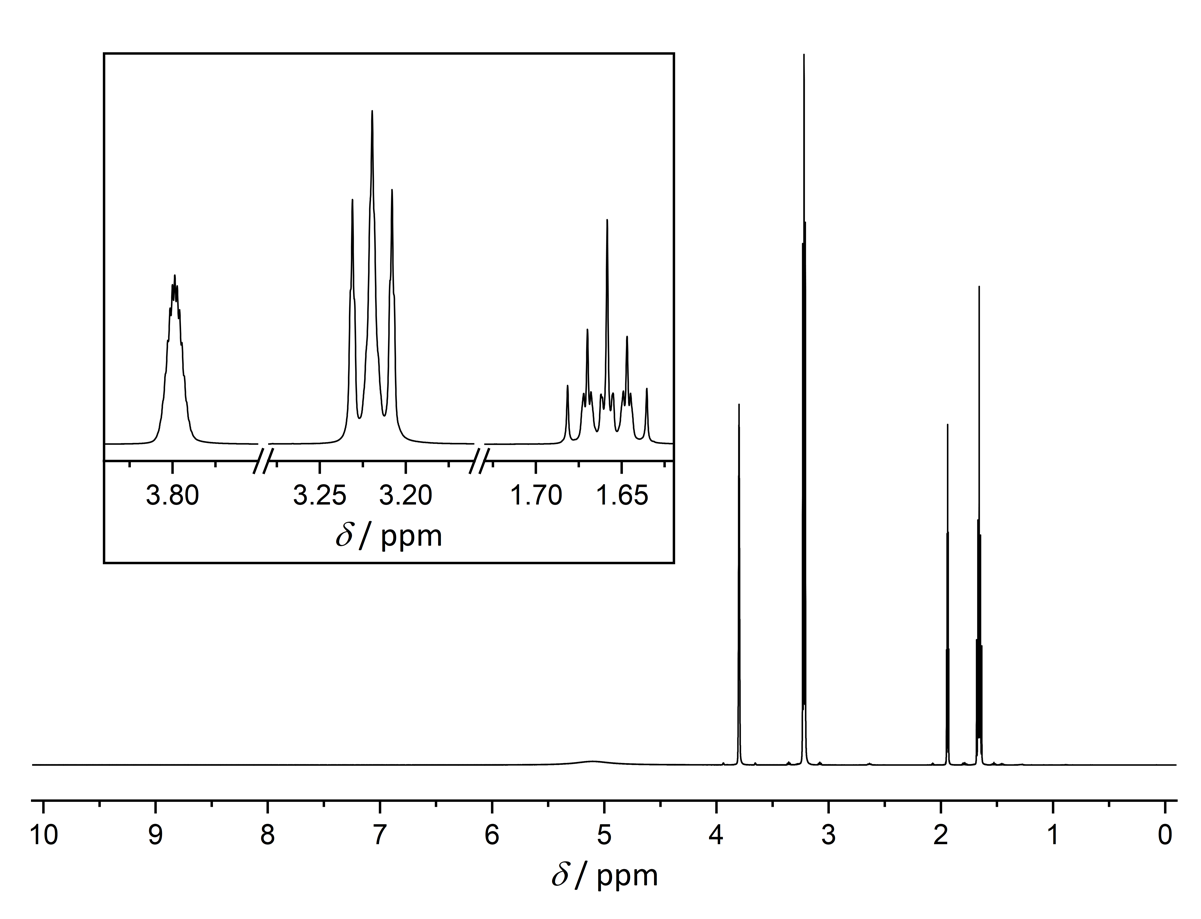


Fig. S56. ^1^H NMR spectrum of **9**.


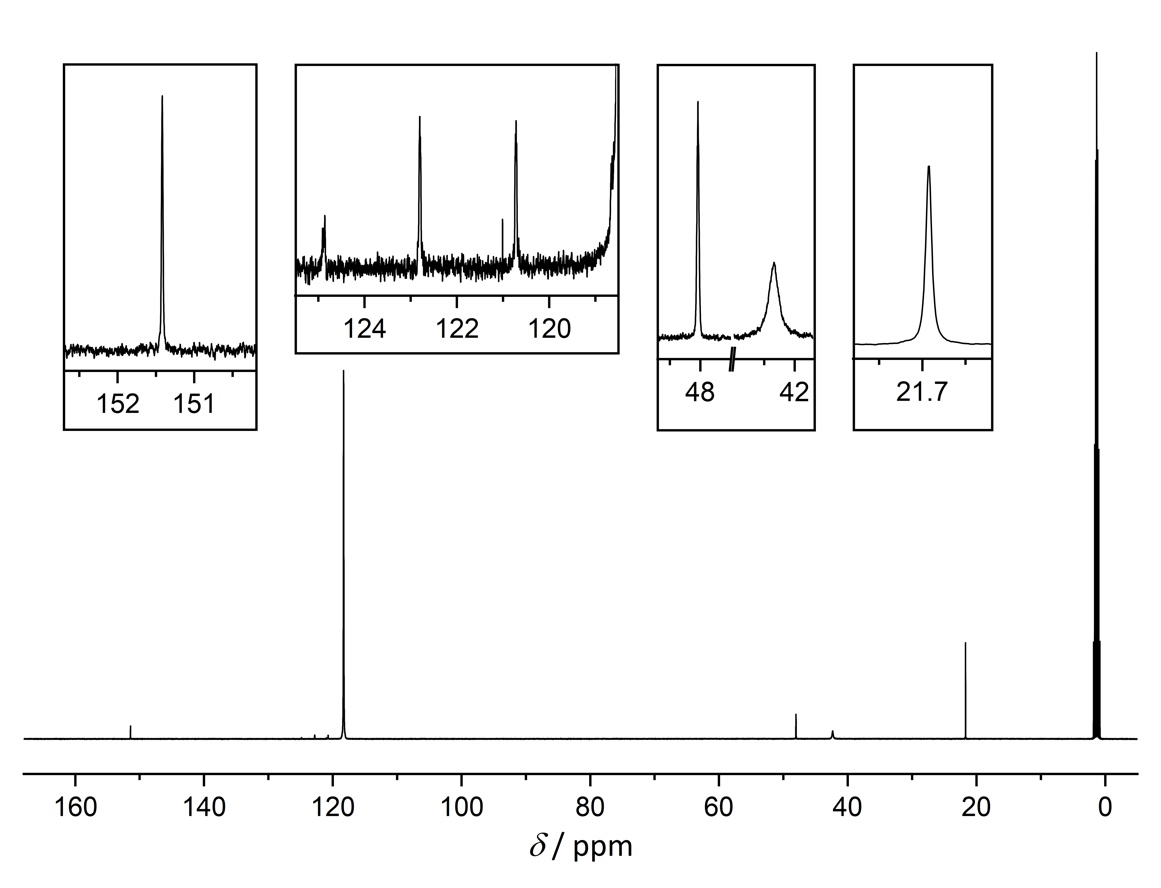


Fig. S57. ^13^C{^1^H} NMR spectrum of **9**.


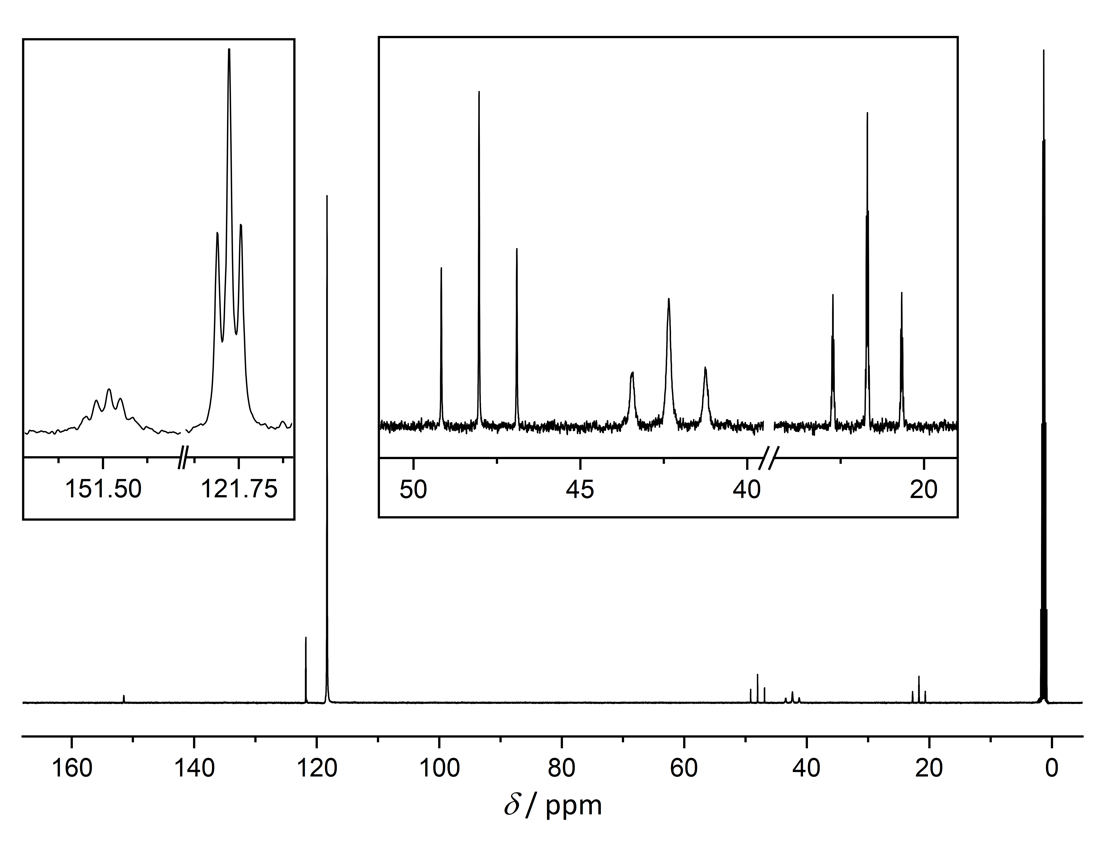


Fig. S58. ^13^C{^19^F} NMR spectrum of **9**.


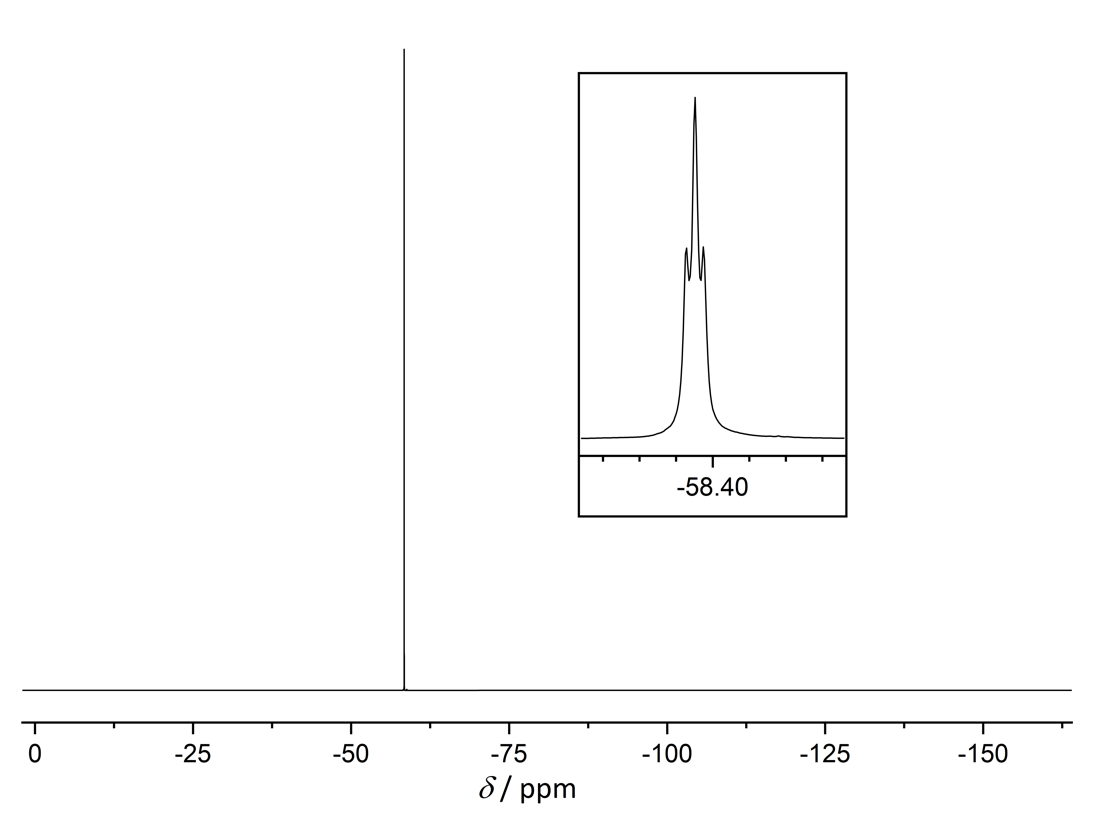


Fig. S59. ^19^F NMR spectrum of **9**.


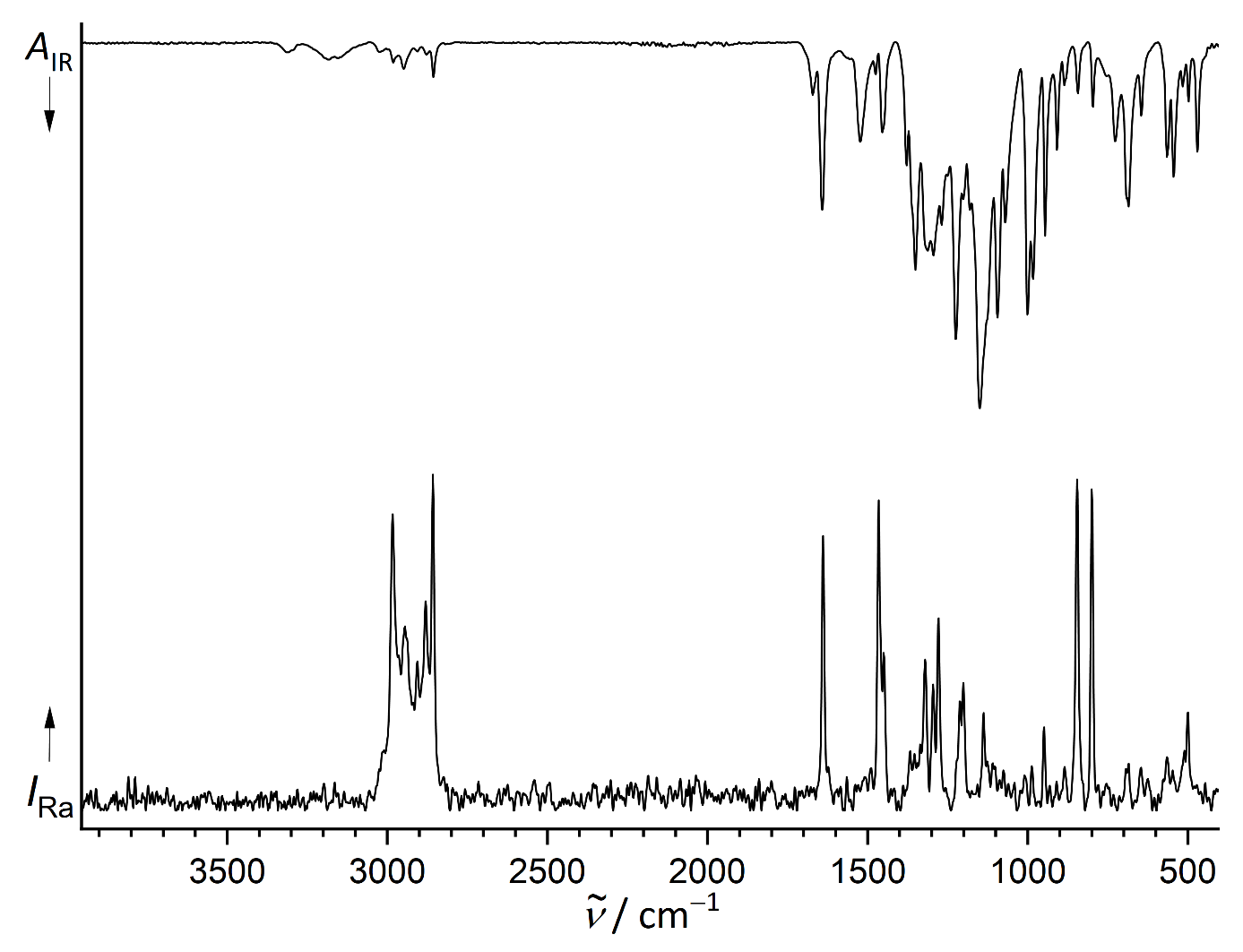


Fig. S60. IR (top) and Raman spectrum (bottom) of **9**.


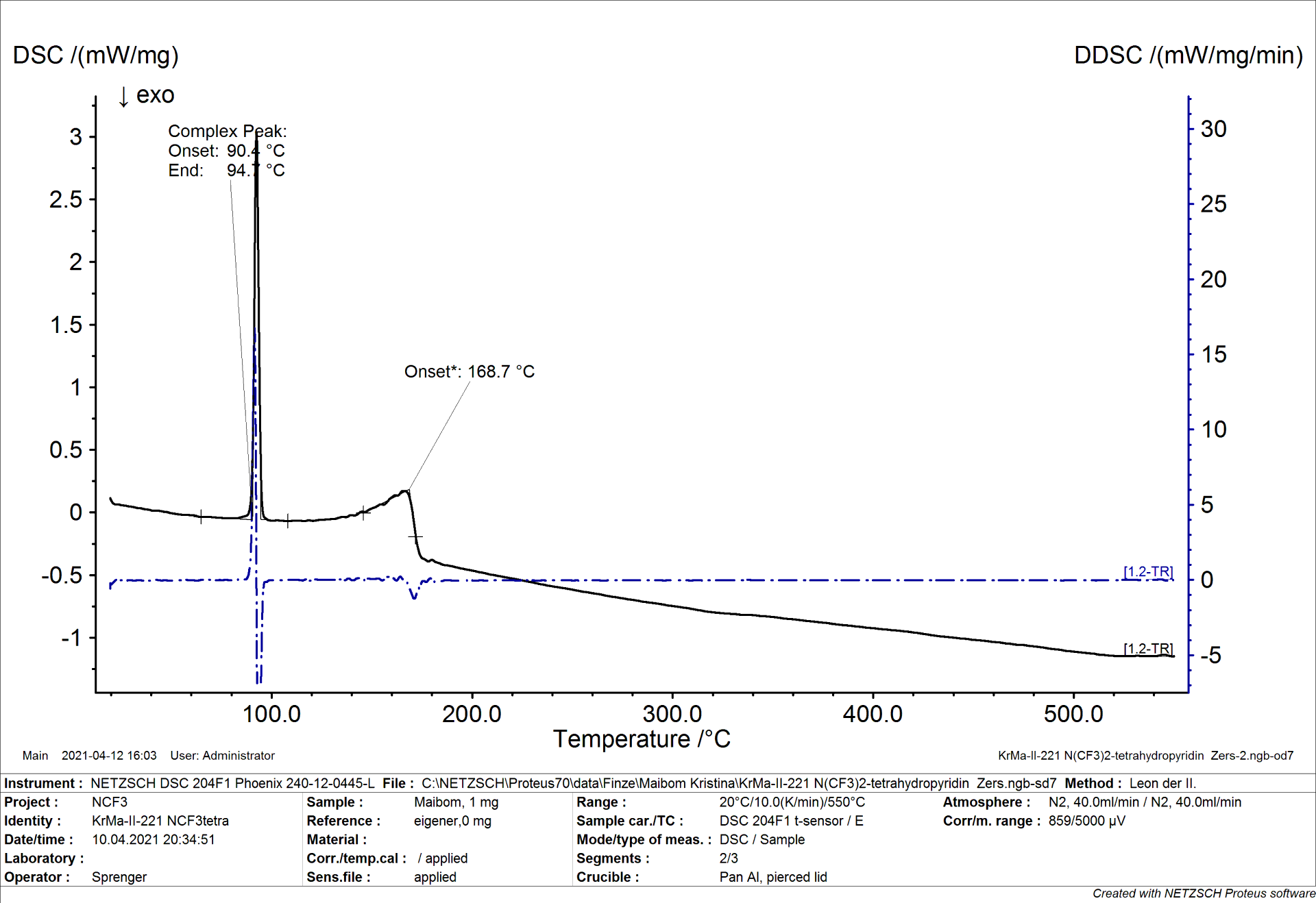


Fig. S61. DSC curve of **9**.

**1-(3a,4,5,6,7,7a-Hexahydro-1*H*-benzo[d]imidazol-2-yl)-*N*,*N*-bis(trifluoromethyl)amine (10)**

*1R*,*2S*-Cyclohexane-1,2-diamine (1.43 g, 12.5 mmol) was added to a suspension of elemental sulfur (40.7 mg, 1.27 mmol) in *N*,*N*-bis(trifluoromethyl)aminoacetonitrile (603 mg, 3.14 mmol; **1**) and the mixture was stirred at 150 °C for 1 h. The reaction mixture was cooled to room temperature and the solid material that had formed was filtered off, washed with water (24 mL), and dried under reduced pressure to give crude **10**. Colorless solid **10** was obtained after column chromatography (MeOH/ethyl acetate 1:9, 2% triethylamine). Yield: 536 mg (1.85 mmol, 59%).

Elemental analysis: calculated (%) for C_10_H_13_F_6_N_3_, C 41.20, H 4.63, N 14.03; found, C 41.53, H 4.53, N 14.53.

HRMS (ASAP+) m/z, calculated for C_10_H_14_F_6_N_3_^+^: 290.1074 (100%), 291.1112 (10.6%), 291.1053 (0.9%); found: 290.1087 (100%), 291.1120 (10.8%), 291.1057 (1.1%).

^1^H NMR (500 MHz, CD_3_CN): *δ* = 5.67─2.81 (br s, 1H, N*H*), 4.03 (s, 2H, C*H*_2_─N(CF_3_)_2_), 3.65─3.58 (m, 2H, C*H*), 1.72─1.62 (m, 2H, CH─C*H*_2_), 1.52─1.44 (m, 2H, CH─C*H*_2_), 1.44─1.37 (m, 2H, CH_2_─C*H*_2_─CH_2_), 1.34─1.23 ppm (m, 2H, CH_2_─C*H*_2_─CH_2_).

^13^C{^1^H} NMR (126 MHz, CD_3_CN): *δ* = 162.2 (sept, 1C, ^4^*J*_F,C_ = 0.6 Hz, N─*C*=N), 121.6 (qm, 2C, ^1^*J*_F,C_ = 261 Hz, *C*F_3_), 62.0─60.8 (m, 2C, *C*H), 43.5 (sept, 1C, ^3^*J*_F,C_ = 1.4 Hz, *C*H_2_─N(CF_3_)_2_), 28.9 (s, 2C, CH─*C*H_2_), 21.8 ppm (s, 2C, CH_2_─*C*H_2_─CH_2_).

^13^C{^19^F} NMR (126 MHz, CD_3_CN): *δ* = 162.3─162.2 (m, 1C, N─*C*=N), 121.6 (t, 2C, ^3^*J*_C,H_ = 4.1 Hz, *C*F_3_), 61.4 (dm, 2C, ^1^*J*_C,H_ = 136 Hz, *C*H), 43.5 (t, 1C, ^1^*J*_C,H_ = 143 Hz, *C*H_2_─N(CF_3_)_2_), 28.9 (tm, 2C, ^1^*J*_C,H_ = 126 Hz, CH─*C*H_2_), 21.8 ppm (tm, 2C, ^1^*J*_C,H_ = 130 Hz, CH_2_─*C*H_2_─CH_2_).

^15^N NMR (^19^F^­^­-^15^N HMBC, CD_3_CN): *δ* = −299.7 ppm (s, 1N, *N*(CF_3_)_2_).

^19^F NMR (471 MHz, CD_3_CN): *δ* = −58.2 ppm (t, 6F, ^4^*J*_F,H_ = 1.3 Hz, C*F*_3_).


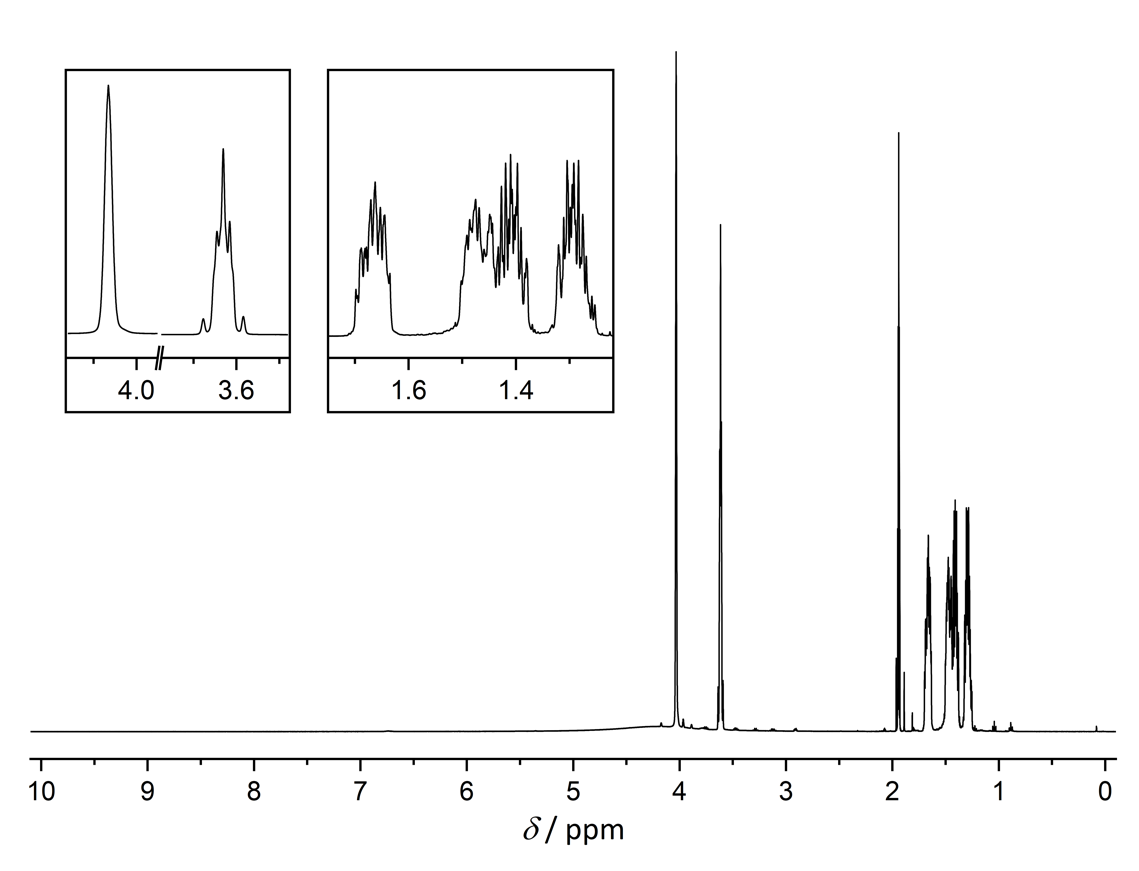


Fig. S62. ^1^H NMR spectrum of **10**.


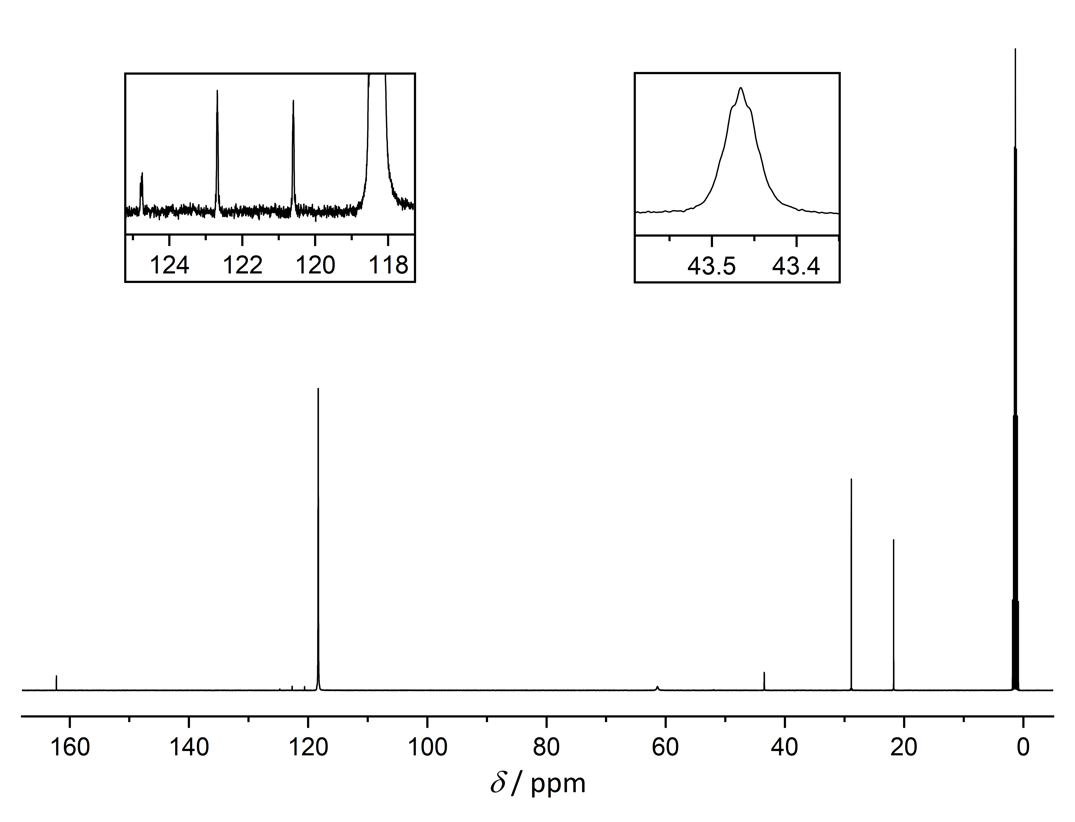


Fig. S63. ^13^C{^1^H} NMR spectrum of **10**.


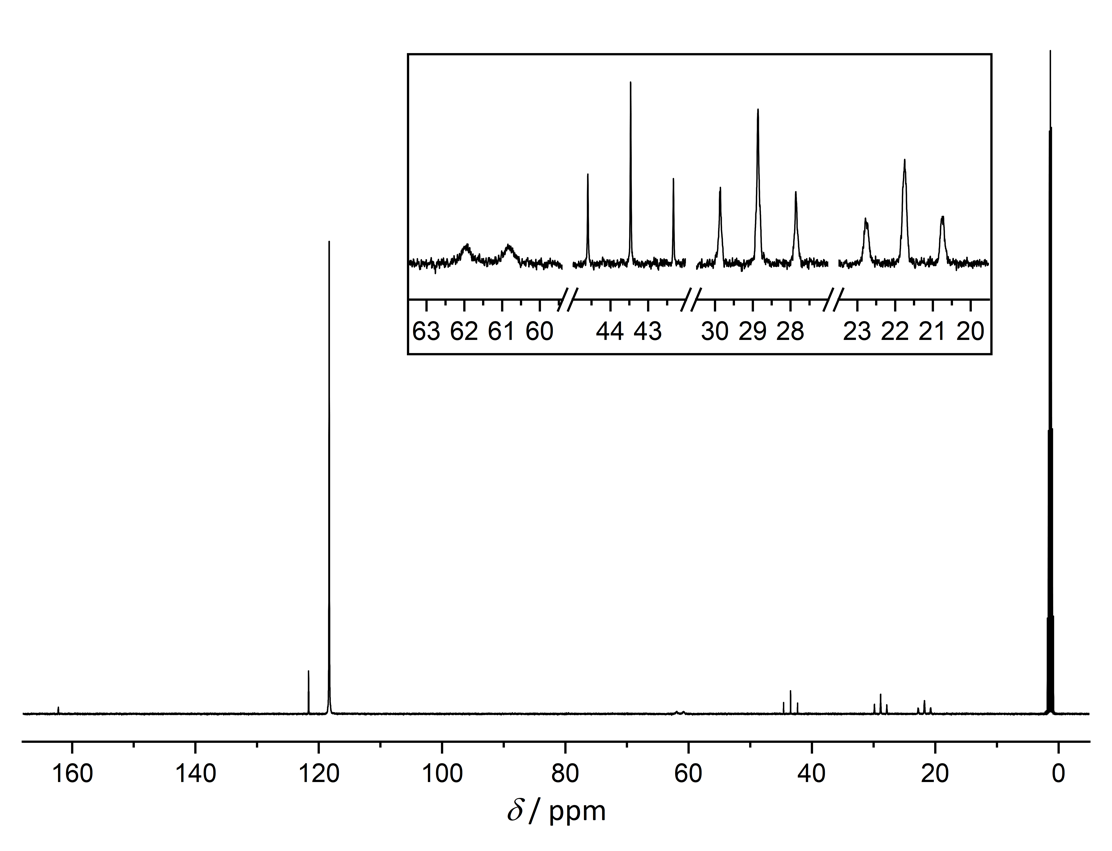


Fig. S64. ^13^C{^19^F} NMR spectrum of **10**.


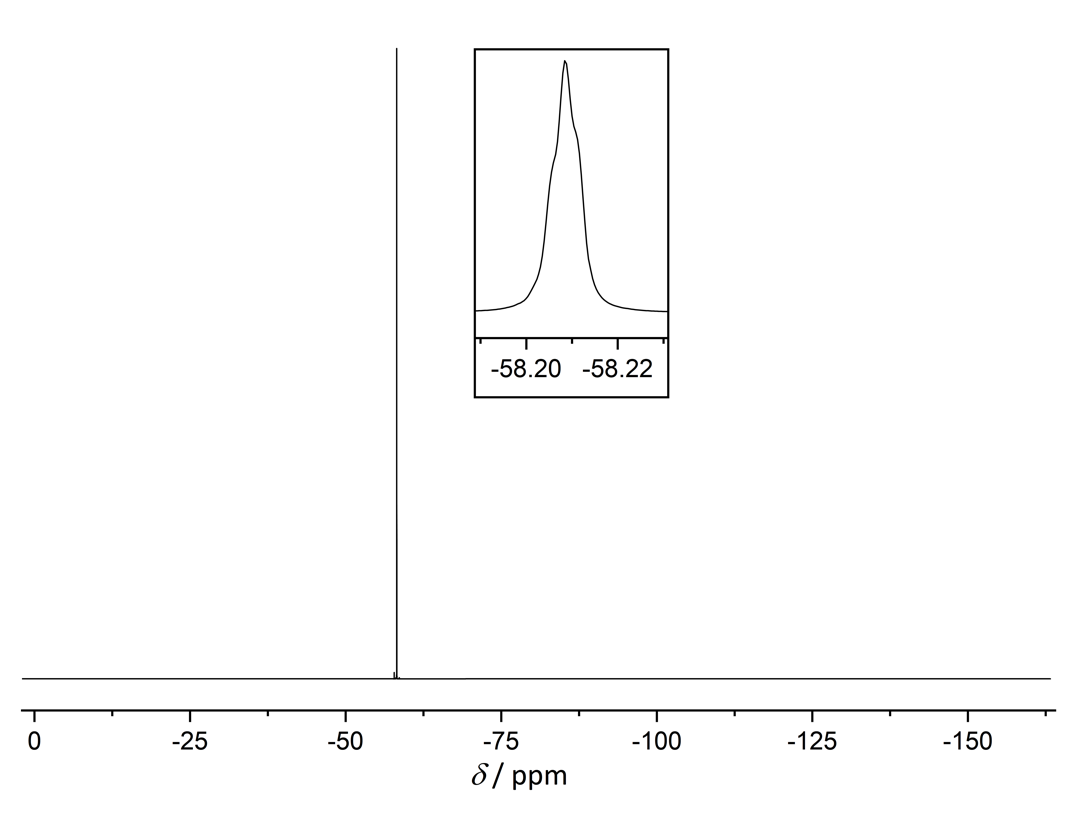


Fig. S65. ^19^F NMR spectrum of **10**.


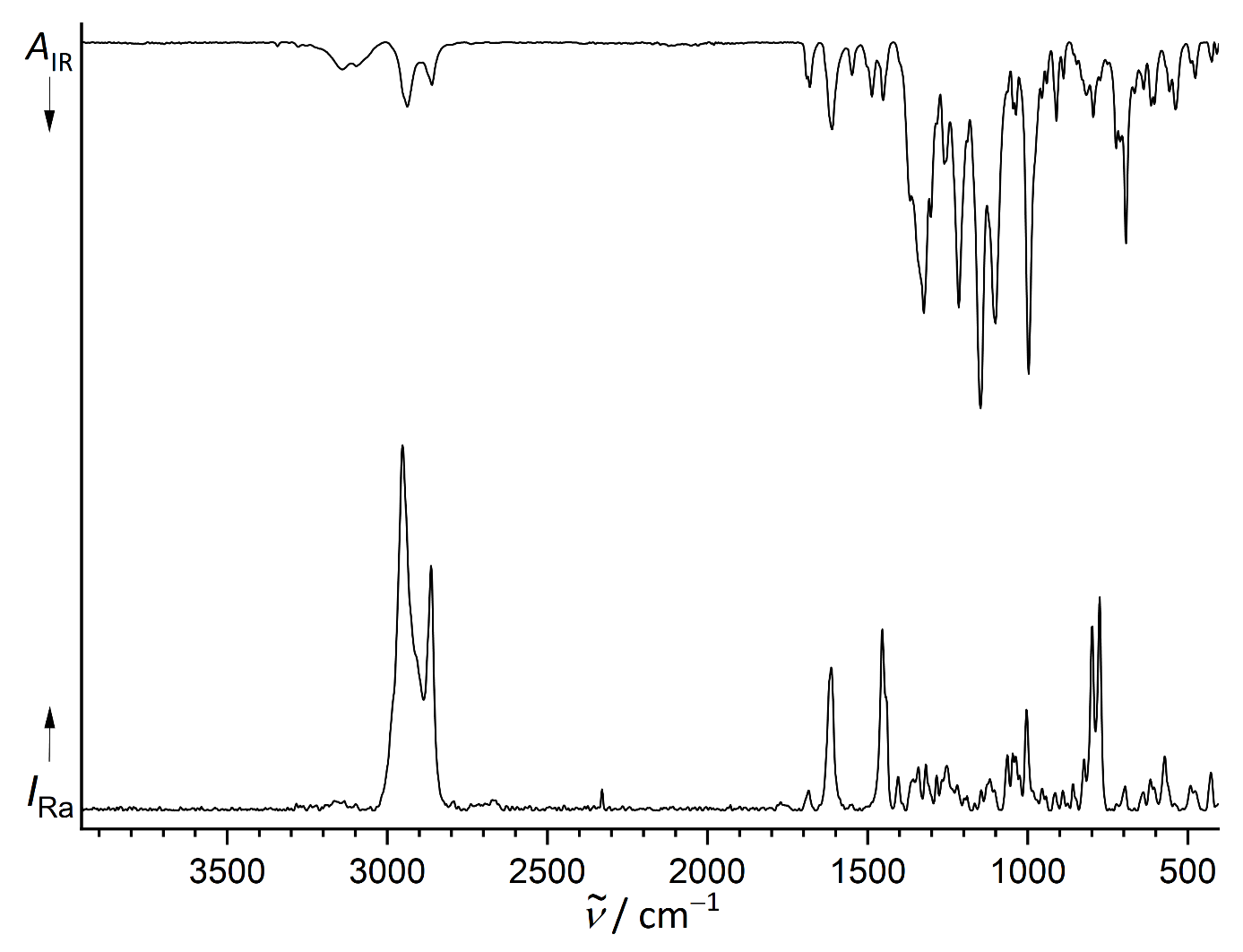


Fig. S66. IR (top) and Raman spectrum (bottom) of **10**.


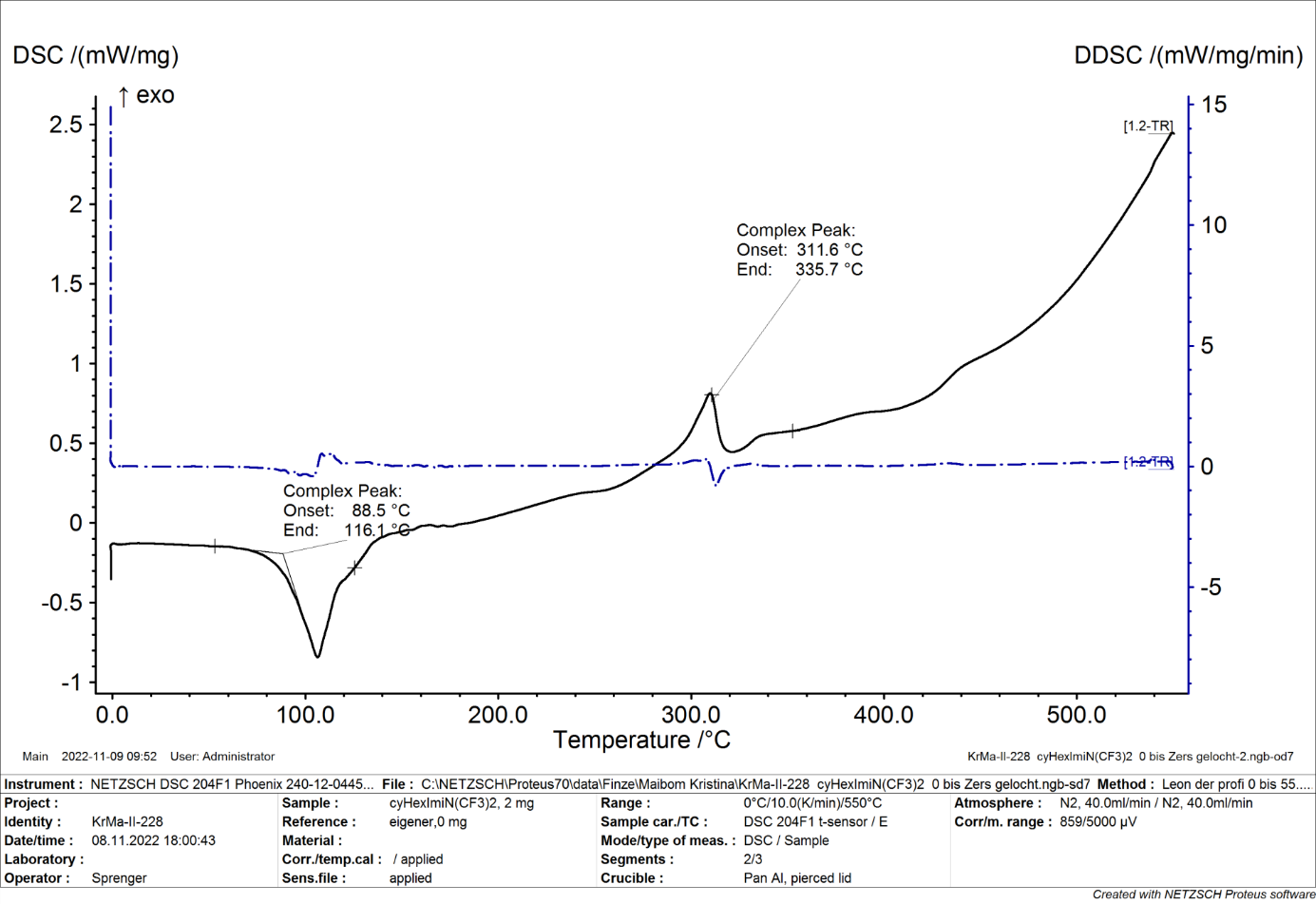


Fig. S67. DSC curve of **10**.

**2-Bis(trifluoromethyl)amino-*N*-(2-hydroxyethyl)acetamide (11)**

Ethanolamine (1.10 mL, 18.4 mmol) was added to a suspension of elemental sulfur (63.3 mg, 1.97 mmol) in *N,N*-bis*(*trifluoromethyl)aminoacetonitrile (920 mg, 4.79 mmol; **1**). The reaction mixture was stirred at 130 °C for one hour. The mixture was cooled to room temperature and poured onto water (10 mL). The precipitate was isolated by filtration and washed with water (3 x 1 mL) and dried under reduced pressure. The solid was dissolved in DCM (3 x 10 mL). The mixture was washed with water (3 x 10 mL), dried with MgSO_4_, and all volatiles were removed under reduced pressure. The crude product was purified by column chromatography (silica gel, DCM/acetonitrile 1:1) followed by sublimation (23 °C, 1 $\cdot$ 10^-3^ mbar) to yield colorless **11**. Yield: 350 mg (1.48 mmol, 31%).

Elemental analysis: calculated (%) for C_6_H_8_F_6_N_2_O_2_, C 28.36, H 3.17, N 11.02; found, C 28.89, H 3.59, N 10.69.

HRMS (ASAP+) m/z, calculated for C_6_H_9_F_6_N_2_O_2_^+^: 255.0563; found: 255.0557.

^1^H NMR (500 MHz, CD_3_CN): *δ* = 6.77 (s, 1H, N*H*), 3.95 (sept, 2H, ^4^*J*_F,H_ = 1.6 Hz, C*H_2_*N(CF_3_)_2_), 3.52 (t, 2H, ^3^*J*_H,H_ = 5.5 Hz, C*H_2_*OH), 3.28 (dt, 2H, ^3^*J*_H,H_ = 5.5 Hz, ^3^*J*_H,H_ = 5.7 Hz, C*H_2_*NH), 2.8 ppm (s, 1H, O*H*, FWHH = 230 Hz).

^13^C{^1^H} NMR (126 MHz, CD_3_CN): *δ*= 167.2 (s, 1C, *C*O), 121.5 (q, 2C, ^1^*J*_F,C_ = 263 Hz, *C*F_3_), 61.4 (s, 1C, *C*H_2_OH), 46.8 (sept, 1C, ^3^*J*_F,H_ = 1.2 Hz, *C*H_2_N(CF_3_)_2_), 42.8 ppm (s, 1C, *C*H_2_NH).

^13^C{^19^F} NMR (126 MHz, CD_3_CN): *δ* = 167.2 (m, 1C, *CO*), 121.5 (t, 2C, ^3^*J*_C,H_ = 4.2 Hz, *C*F_3_), 61.4 (tt, 1C, ^1^*J*_C,H_ = 141 Hz, ^2^*J*_C,H_ = 3.4 Hz, *C*H_2_OH), 46.8 (t, 1C, ^1^*J*_C,H_ = 142 Hz, *C*H_2_N(CF_3_)_2_), 42.8 ppm (ttd, 1C, ^1^*J*_C,H_ = 138 Hz, ^2^*J*_C,H_ = 1.9 Hz, ^2^*J*_C,H_ = 1.5 Hz *C*H_2_NH).

^15^N NMR (^15^N-^1^H HMBC and ^15^N-^1^H HSQC, CD_3_CN): *δ* = −273.2 ppm (s, 1N, *N*H).

^15^N NMR (^15^N-^19^F HMQC, CD_3_CN): *δ* = −300.4 ppm (s, 1N, *N*(CF_3_)_2_).

^19^F NMR (471 MHz, CD_3_CN): *δ* = −58.1 ppm (t, 6F, ^4^*J*_F,H_ = 1.6 Hz, C*F*_3_).


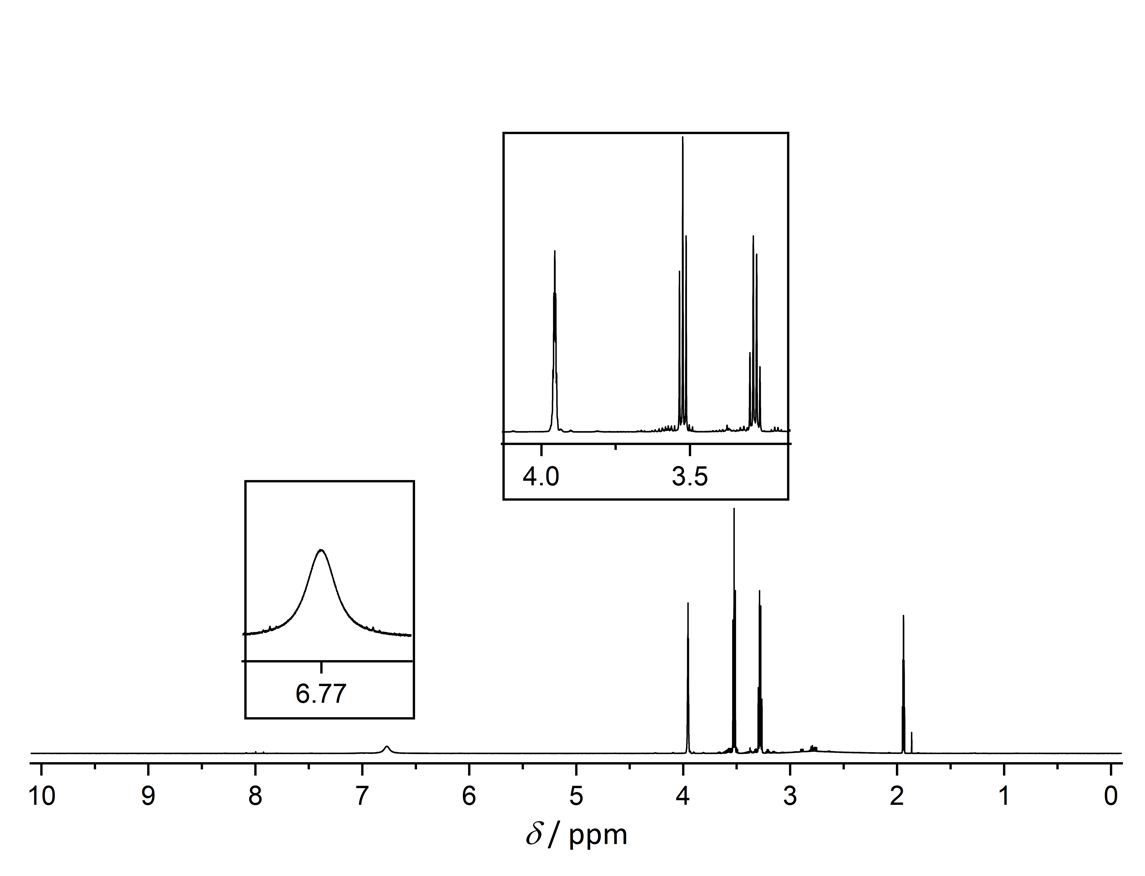


Fig. S68. ^1^H NMR spectrum of **11**.


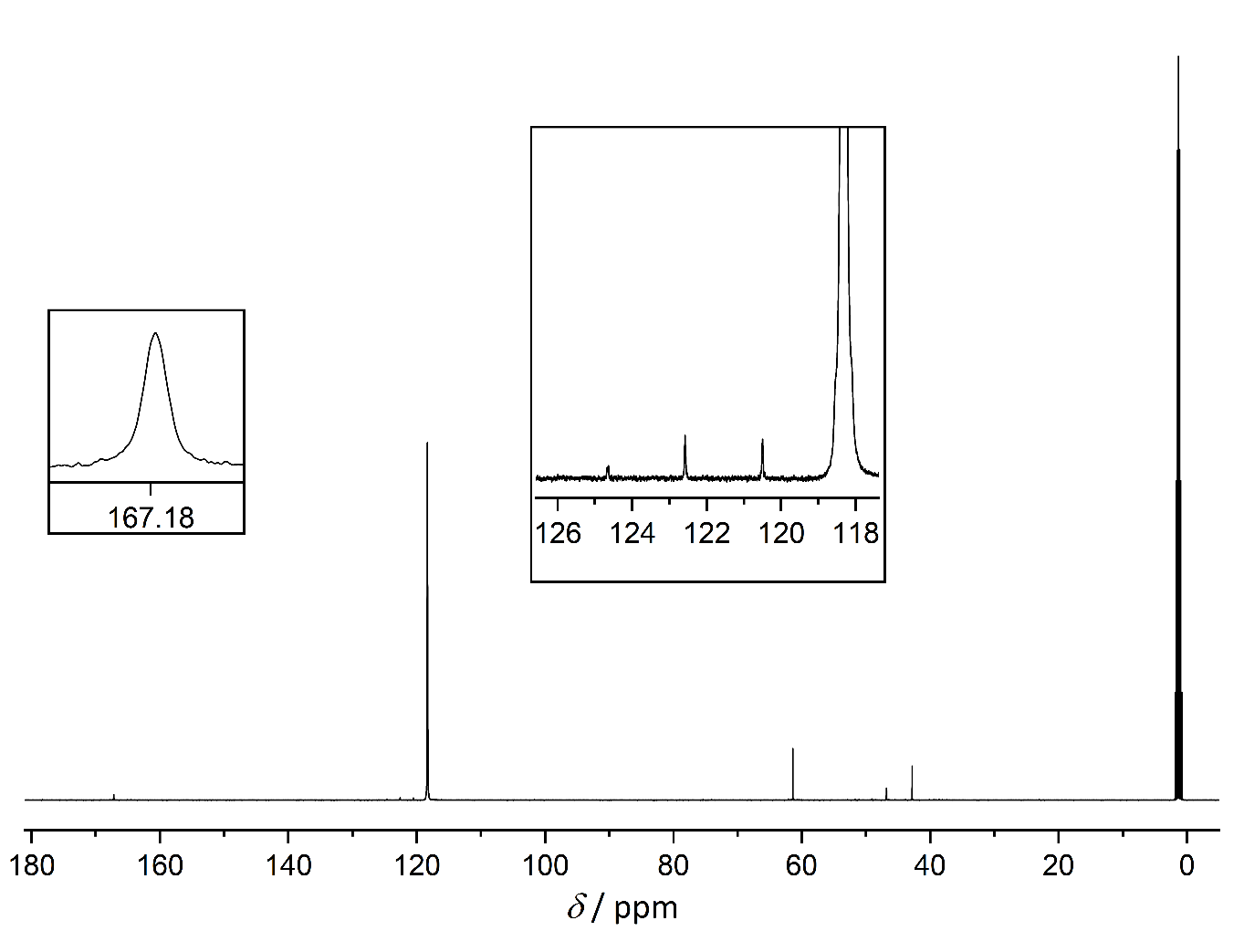


Fig. S69. ^13^C{^1^H} NMR spectrum of **11**.


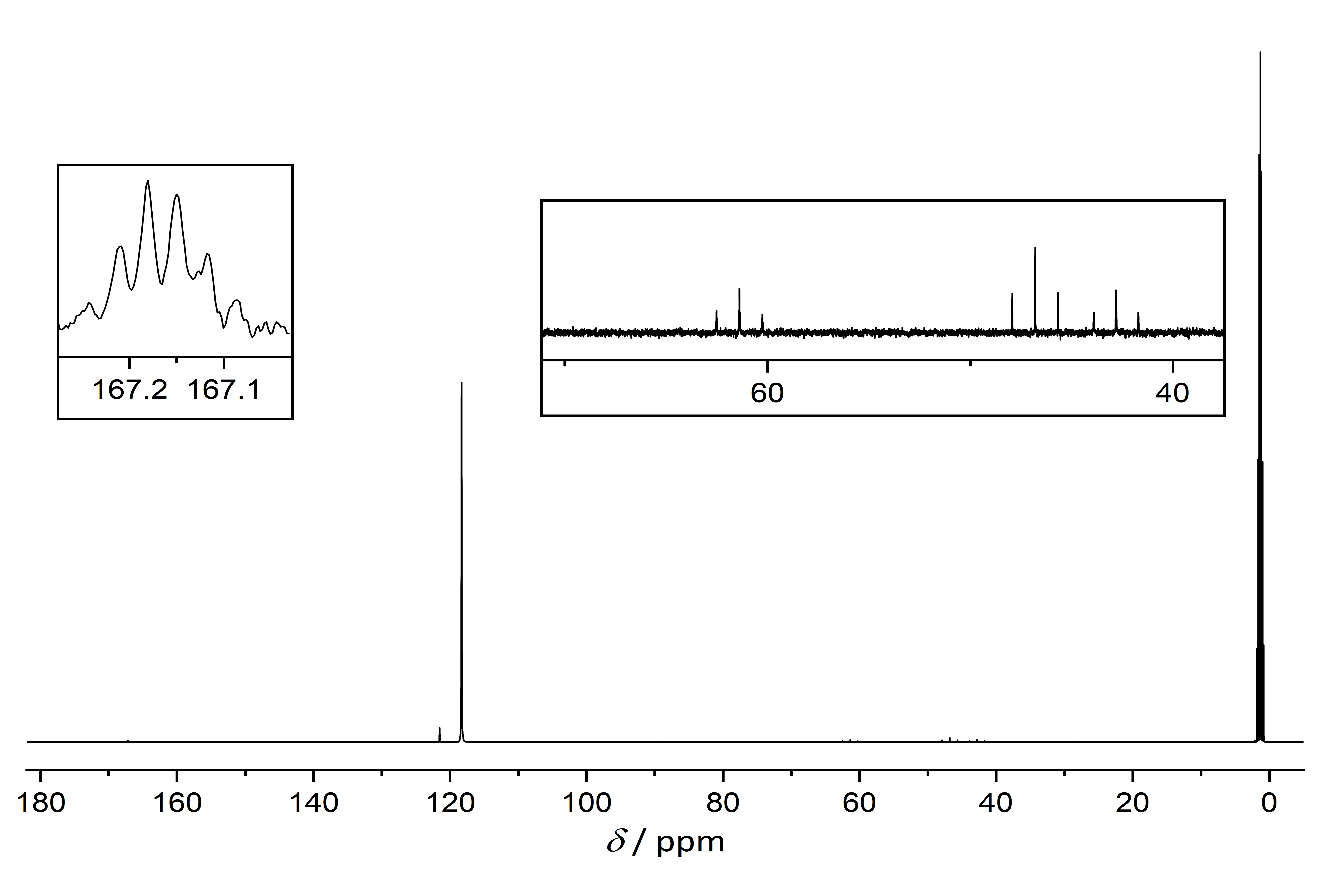


Fig. S70. ^13^C{^19^F} NMR spectrum of **11**.


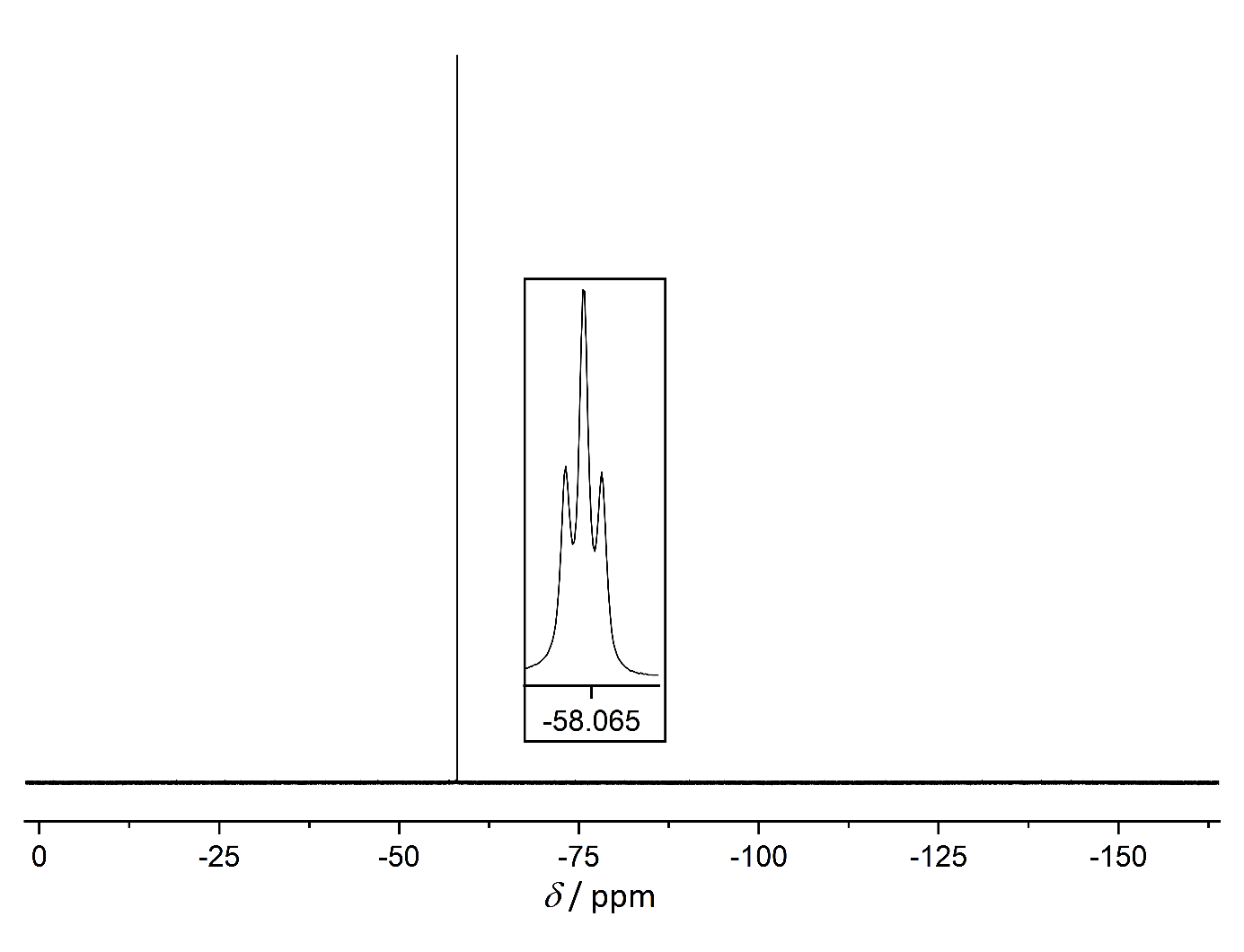


Fig. S71. ^19^F NMR spectrum of **11**.


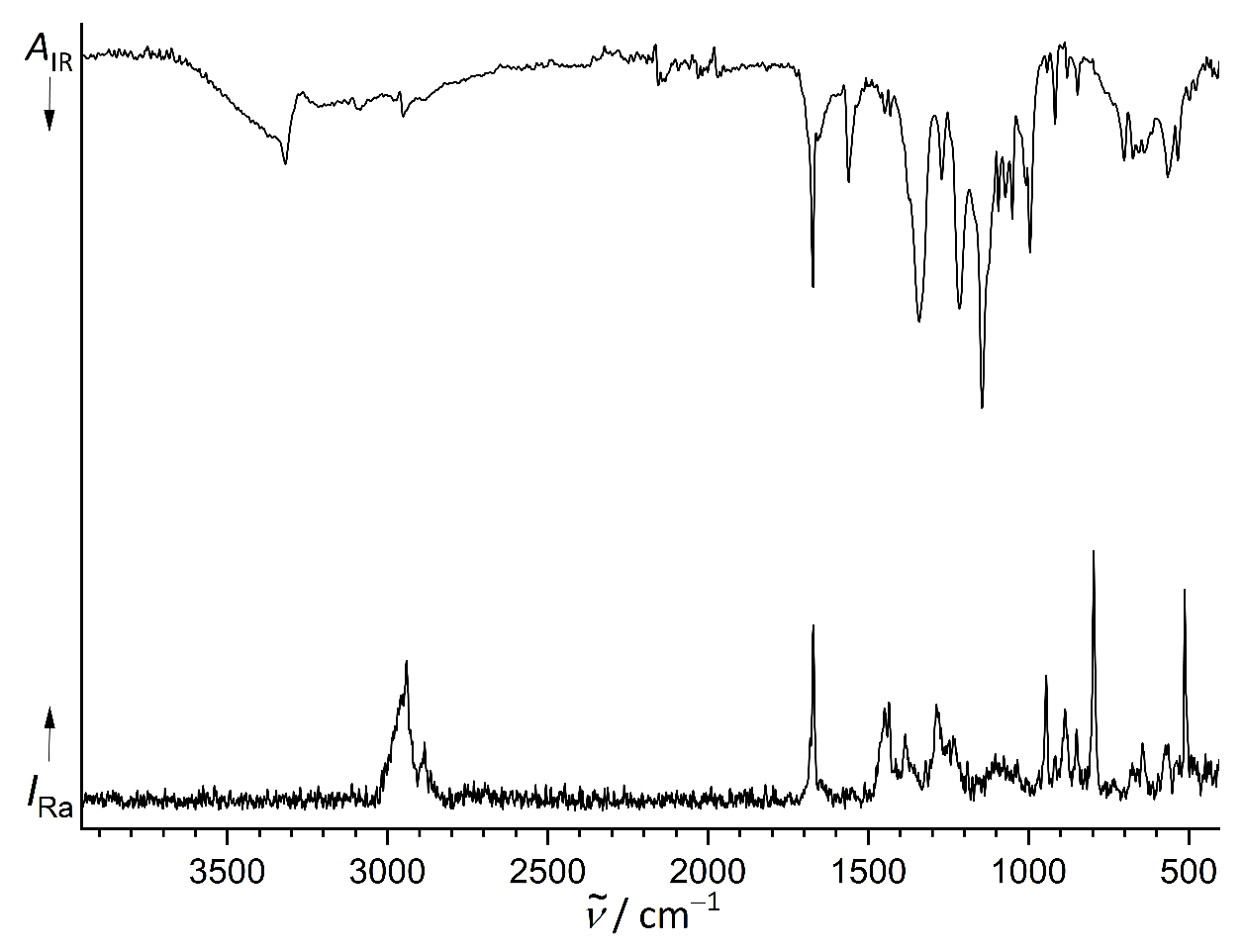


Fig. S72. IR (top) and Raman spectrum (bottom) of **11**.


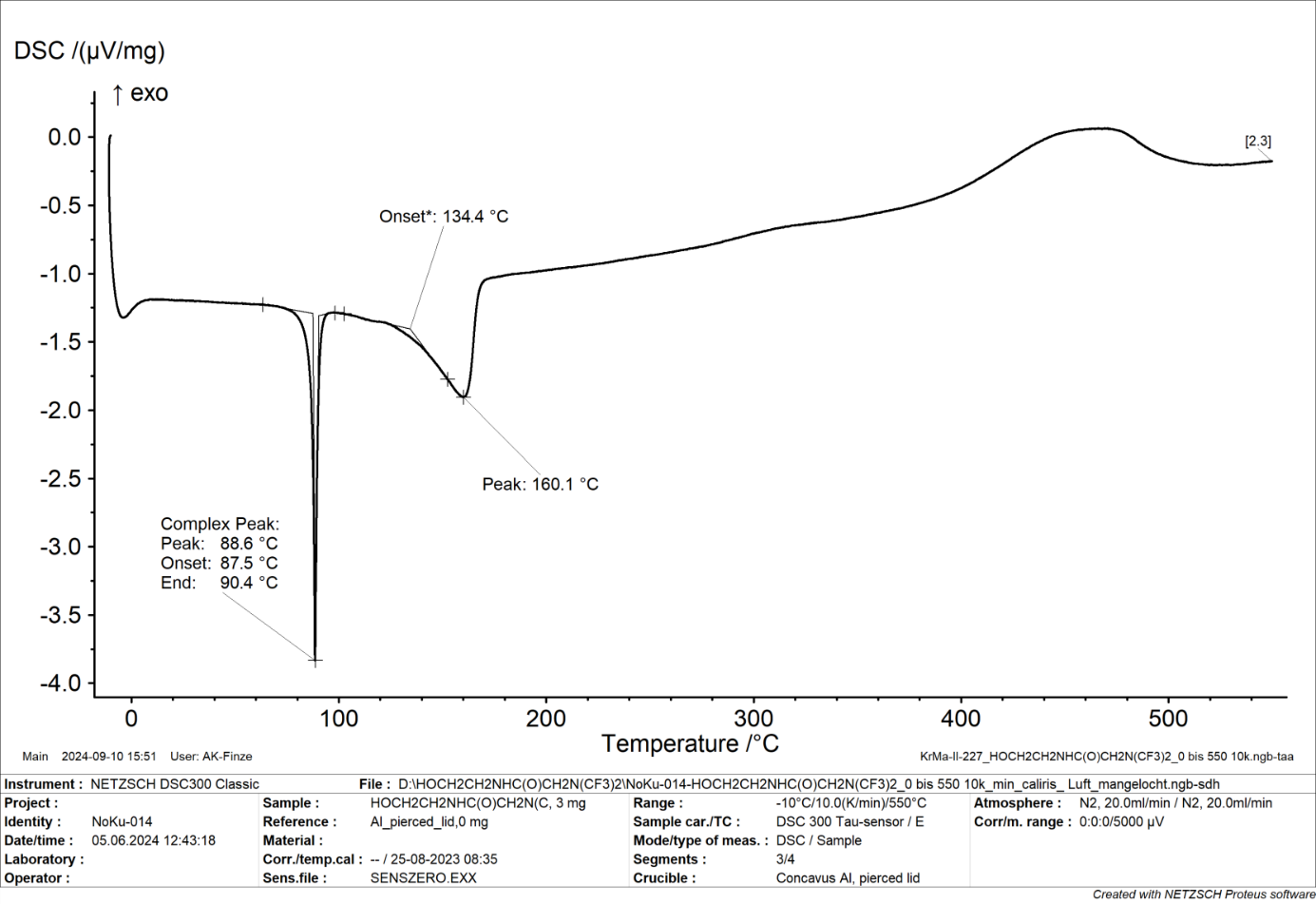


Fig. S73. DSC curve of **11**.

**(*1H*-Tetrazol-5-yl)methyl-*N*,*N*-bis(trifluoromethyl)amine (12)**

*N,N*-bis*(*trifluoromethyl)aminoacetonitrile (1.10 g, 5.73 mmol; **1**) was added to a mixture of sodium azide (400 mg, 6.70 mmol) and trimethylammonium hydrochloride (600 mg, 6.70  mmol) in toluene (6 mL). The reaction mixture was heated to reflux for 18 hours. Cooling to room temperature led to precipitation of a colorless solid that was removed by filtration. The solid was dried in air and purified by sublimation (50 °C, 3 $\cdot$ 10^-3^ mbar). Yield: 530 mg (2.25 mmol, 39%).

Elemental analysis: calculated (%) for C_4_H_3_F_6_N_5_, C 20.44, H 1.29, N 29.79; found, C 20.48, H 1.06, N 29.11.

HRMS (ASAP+) m/z, calculated for C_4_H_4_F_6_N_5_^+^: 236.0365; found:236.0350.

^1^H NMR (500 MHz, CD_3_CN): *δ* = 12.0 (s, 1H, N*H,* FWHH = 193Hz), 4.93 ppm (sept, 2H, ^4^*J*_F,H_ = 1.4 Hz, C*H_2_*N(CF_3_)_2_).

^13^C{^1^H} NMR (126 MHz, CD_3_CN): *δ* = 156.0 (s, 1C, *C*N *Tetrazole*), 121.6, (q, 2C, ^1^*J*_F,C_ = 263 Hz, *C*F_3_), 38.9 ppm (sept, 1C, ^3^*J*_F,H_ = 1.6 Hz, *C*H_2_N(CF_3_)_2_).

^13^C{^19^F} NMR (126 MHz, CD_3_CN): *δ* = 156.0 (t, 1C, ^2^*J*_C,H_ = 5.4 Hz, *C*N *Tetrazole*), 121.6 (t, 2C, ^3^*J*_C,H_ = 4.2 Hz, *C*F_3_), 38.9 ppm (t, 1C, ^1^*J*_H,H_ = 146 Hz *C*H_2_).

^15^N{^1^H} NMR, (CD_3_CN): *δ* = −14.1 (s, 2N, *NN* *Tetrazole*), −88.9 (s, 2N, C*N Tetrazole*), −136.4 ppm (s, 1N, *CD_3_CN*).

^15^N NMR (^15^N-^19^F HMQC, CD_3_CN): *δ* = −298.5 ppm (s, 1N, *N*(CF_3_)_2_).

^19^F NMR (471 MHz, CD_3_CN): *δ* = −58.0 ppm (t, 6F, ^4^*J*_F,H_ 1.3 Hz, C*F*_3_).


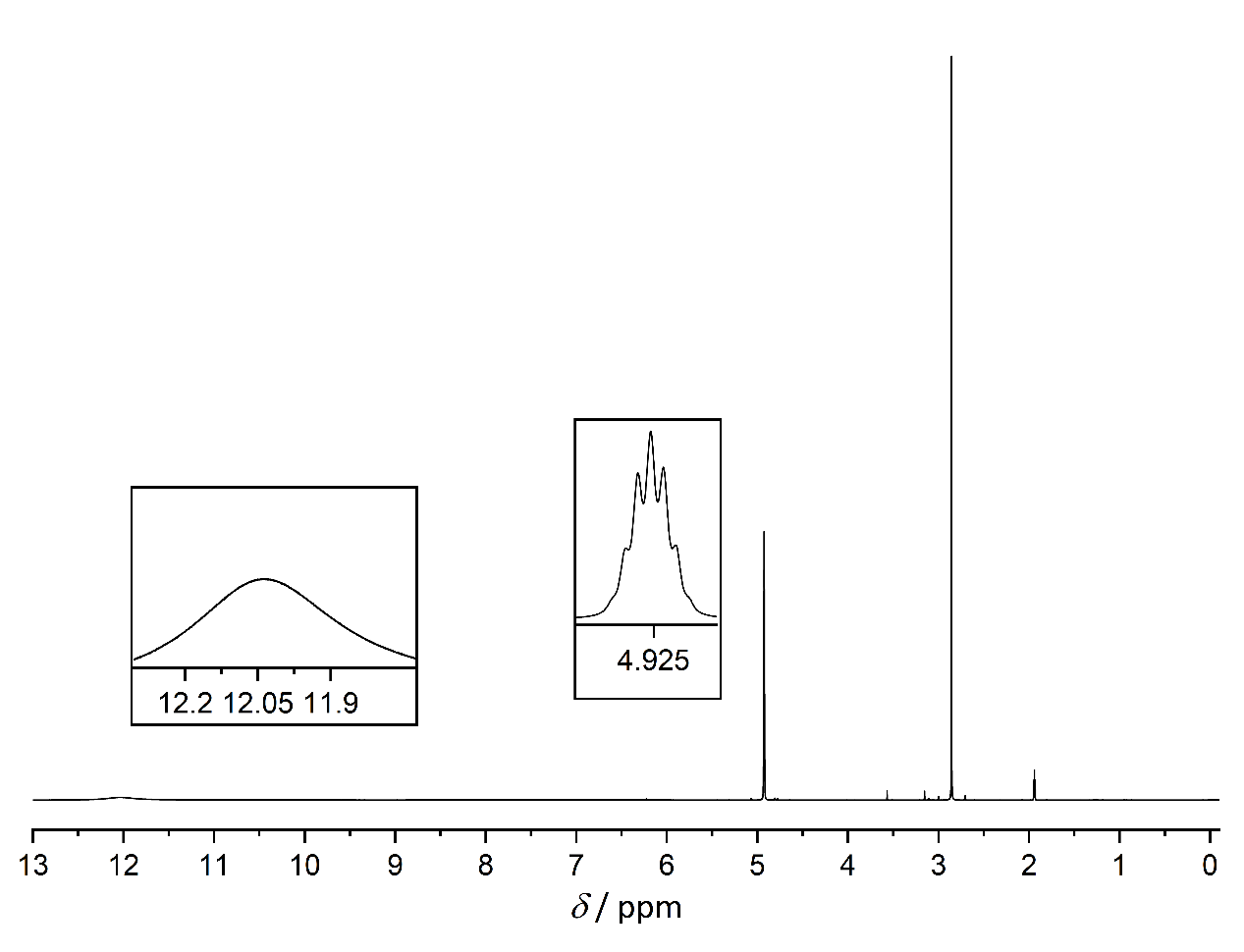


Fig. S74. ^1^H NMR spectrum of **12**.


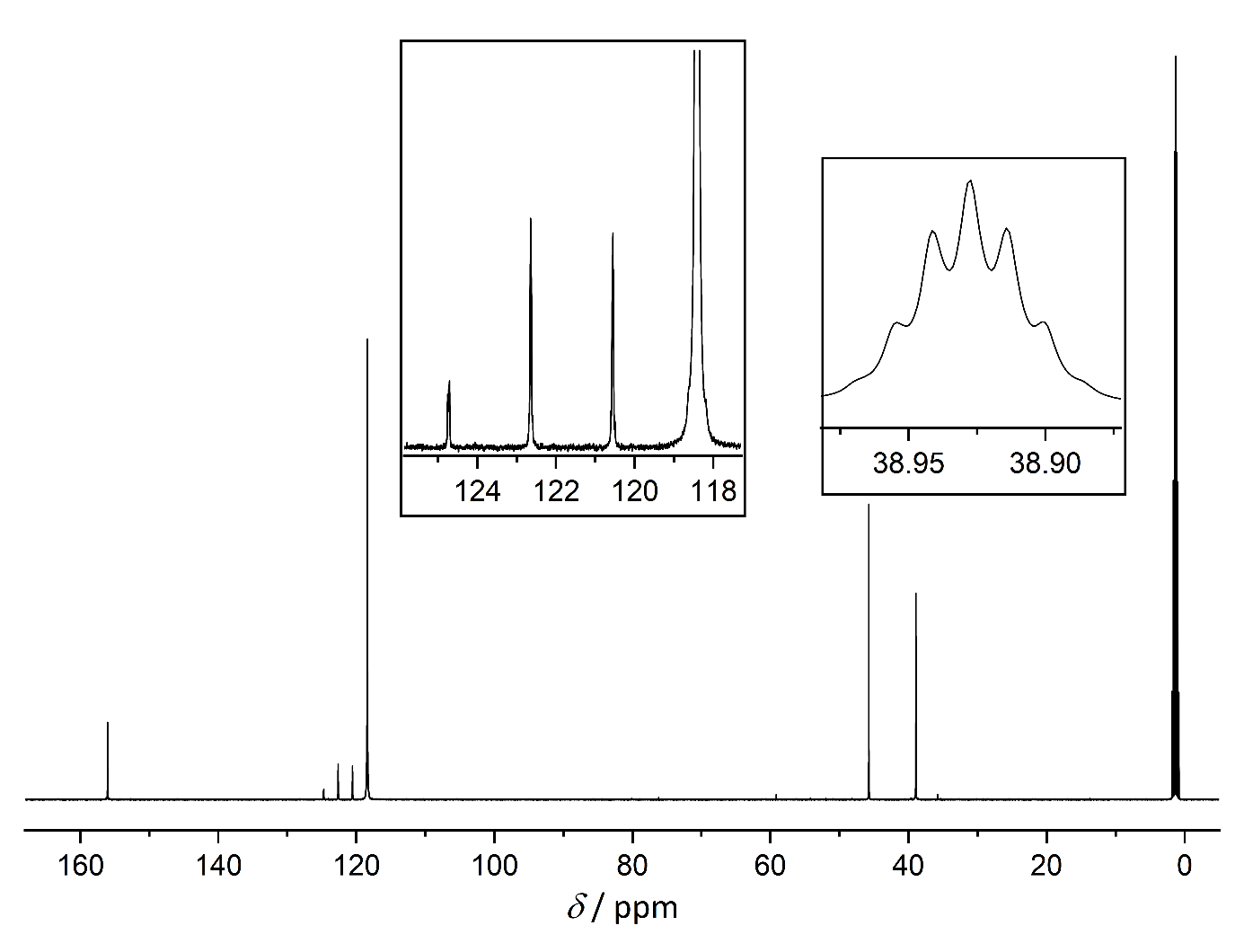


Fig. S75. ^13^C{^1^H} NMR spectrum of **12**.


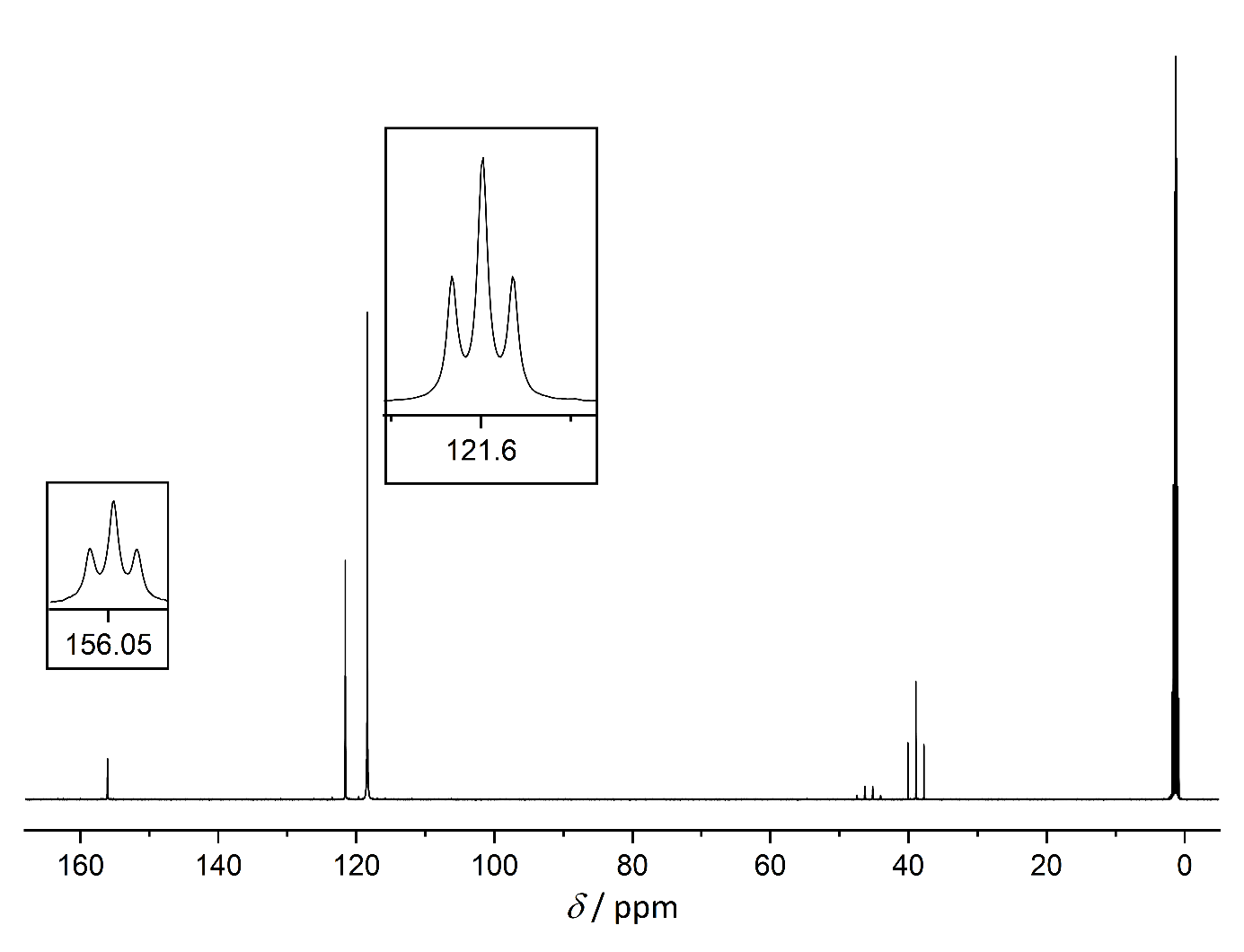


Fig. S76. ^13^C{^19^F} NMR spectrum of **12**.


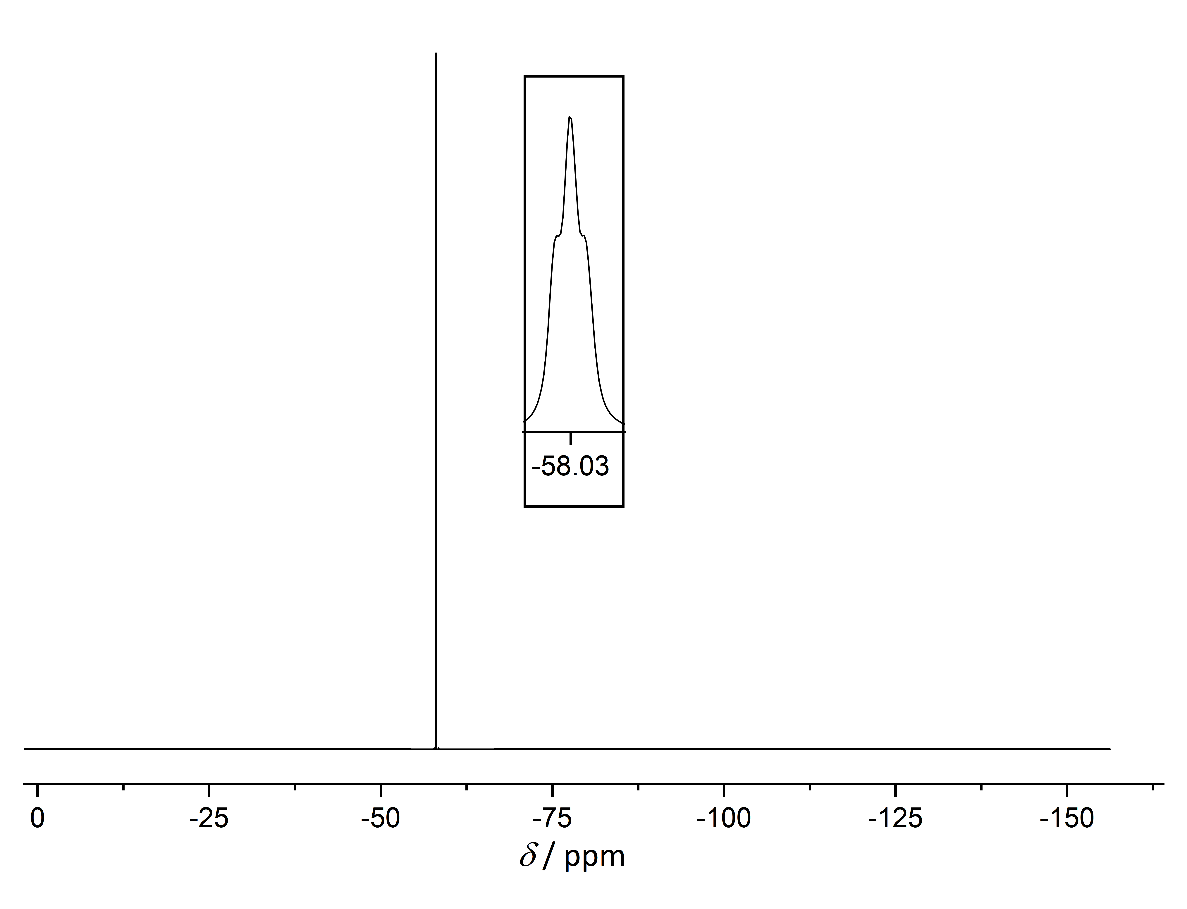


Fig. S77. ^19^F NMR spectrum of **12**.


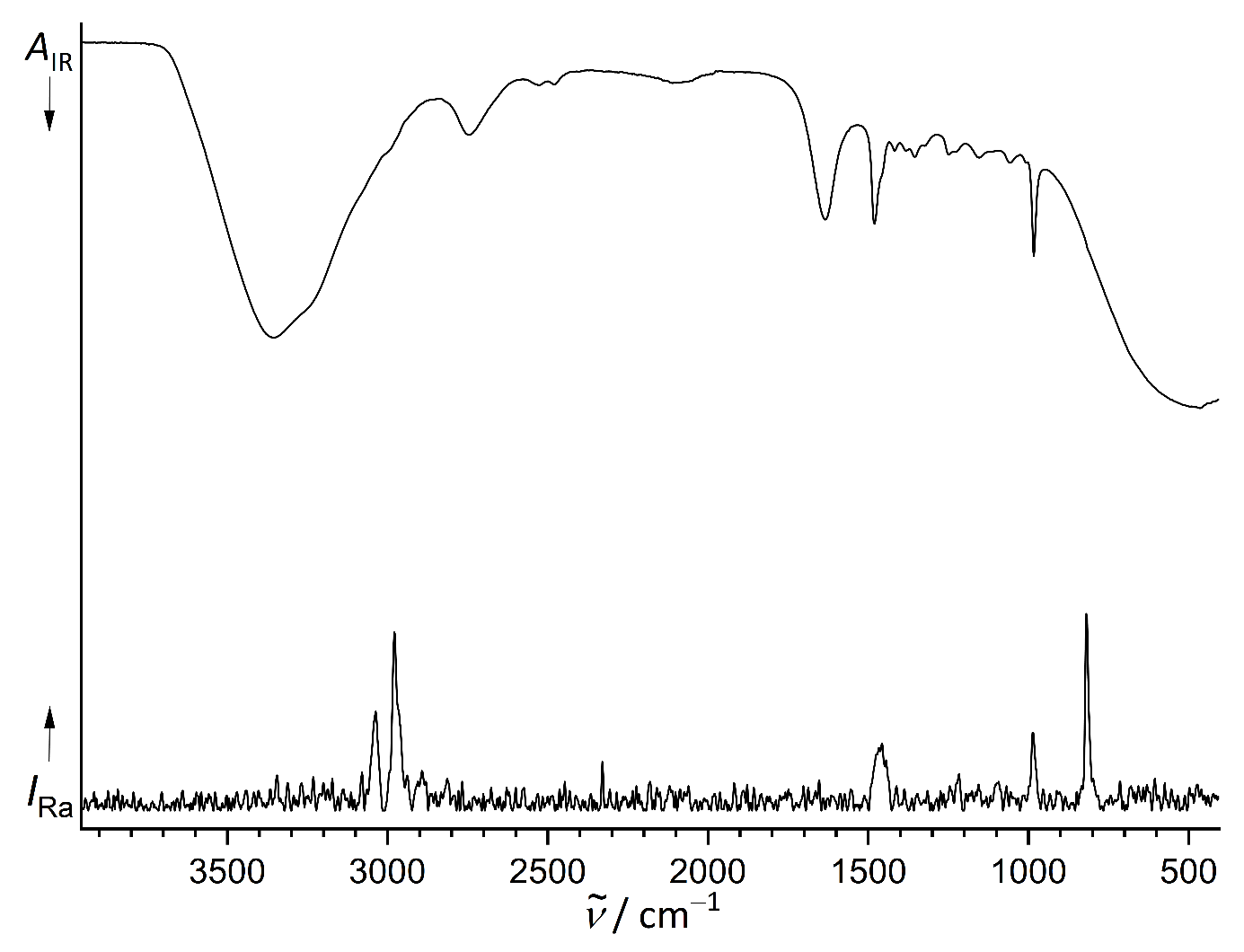


Fig. S78. IR (top) and Raman spectrum (bottom) of **12**.


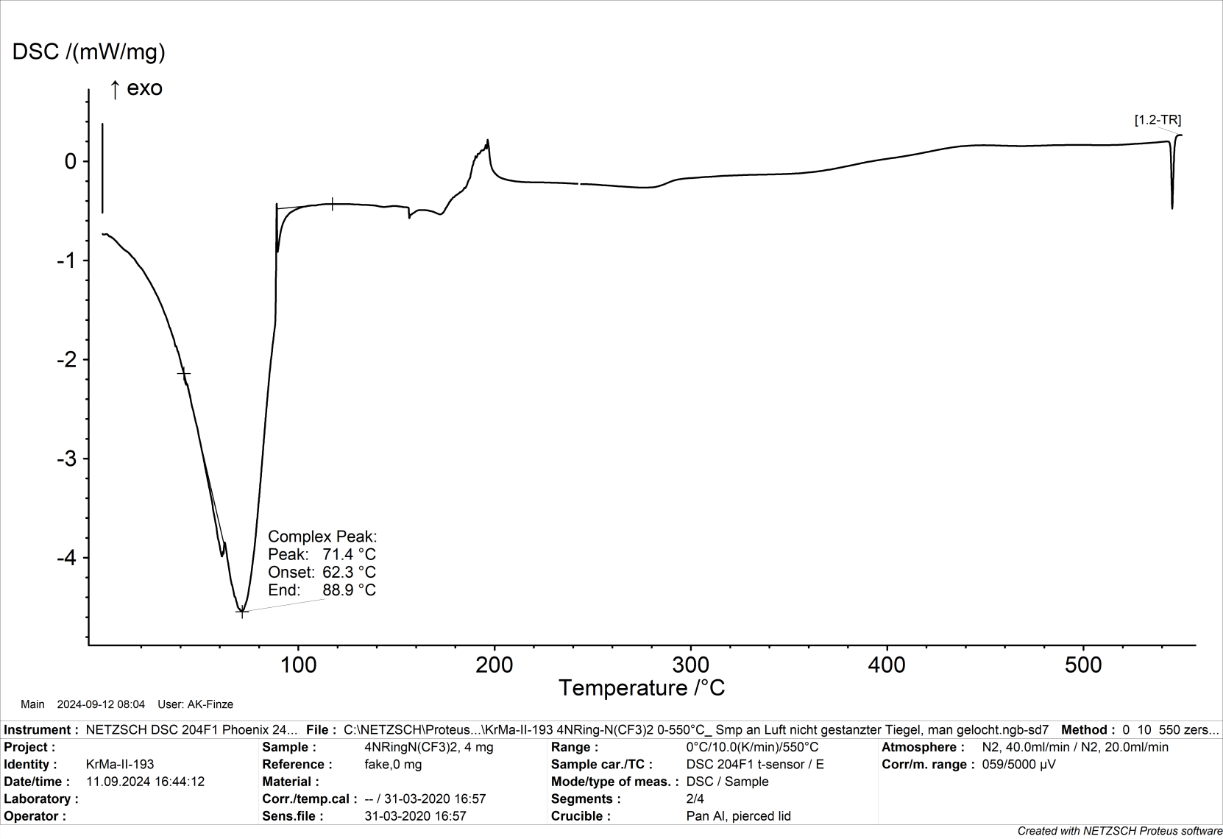


Fig. S79. DSC curve of **12**.

**(*1H*-Imidazol-2-yl)methyl-*N*,*N*-bis(trifluoromethyl)amine (13)**

DMSO (10.8 g, 138 mmol) was added dropwise to a solution of oxalyl chloride (9.30 g, 73.4 mmol) in CH2Cl2 (80 mL) at –78 °C within 30 min. (4,5-Dihydro-*1H*-imidazol-2-yl)methyl-*N*,*N*-bis­(tri­fluoro­methyl)­amine (10.0 g, 42.5 mmol; **8**) was added dropwise within 1 hour. Immediate­ly, a colorless precipitate formed. Subsequently, triethylamine (22.7 g, 224 mmol) was added dropwise at – 78 °C. The reaction mixture was warmed to room temperature overnight. The mixture was washed with water (5 x 4 mL). The organic phase was dried with MgSO_4_ and all volatiles were removed under reduced pressure. The crude product was purified by sublimation (85 °C, 12 mbar) to give colorless **13**. Yield: 4.20 g (18.0 mmol, 42%).

Elemental analysis: calculated (%) for C_6_H_5_F_6_N_3_, C 30.91, H 2.16, N 18.03; found, C 31.64, H 2.71, N 17.51.

HRMS (ASAP+) m/z, calculated for C_6_H_6_F_6_N_3_^+^: 234.0460; found: 234.0452.

^1^H NMR (500 MHz, CD_3_CN): *δ =*10.3 (s, 1H, N*H*, FWHH = 90 Hz), 7.00 (s, 2H, C*H*), 4.56 ppm (sept, 2H, ^4^*J*_F,H_ = 1.4 Hz, C*H_2_*N(CF_3_)_2_).

^13^C{^1^H} NMR (126 MHz, CD_3_CN): *δ* = 142.5 (s, 1C, *C*2), 129.2 (s, 2C, N*CC*N, FWHH = 200 Hz) 121.6 (q, 2C, ^1^*J*_F,C_ = 263 Hz, *C*F_3_), 42.2 ppm (sept, 1C, ^3^*J*_F,H_ = 1.6 Hz, *C*H_2_N(CF_3_)_2_).

^13^C{^19^F} NMR (126 MHz, CD_3_CN): *δ* = 142.6–142.3 (m, 1C, *C*2), 129.2 (s, 2C, N*CC*N, FWHH = 300 Hz), 121.6 (t, 2C, ^3^*J*_C,H_ = 4.1 Hz, *C*F_3_), 42.2 ppm (t, ^1^*J*_C,H_ = 144 Hz, 1C, *C*H_2_N(CF_3_)_2_).

^15^N NMR (^15^N-^1^H HMBC, CD_3_CN): *δ* = −236.7 ppm (2N, *N* Imidazole)

^15^N NMR (^15^N-^19^F HMQC, CD_3_CN): *δ* = −296.9 ppm (s, 1N, *N*(CF_3_)_2_).

^19^F NMR (471 MHz, CD_3_CN): *δ* = −57.7 ppm (t, 6F, ^4^*J*_F,H_ =  1.3 Hz, C*F*_3_).


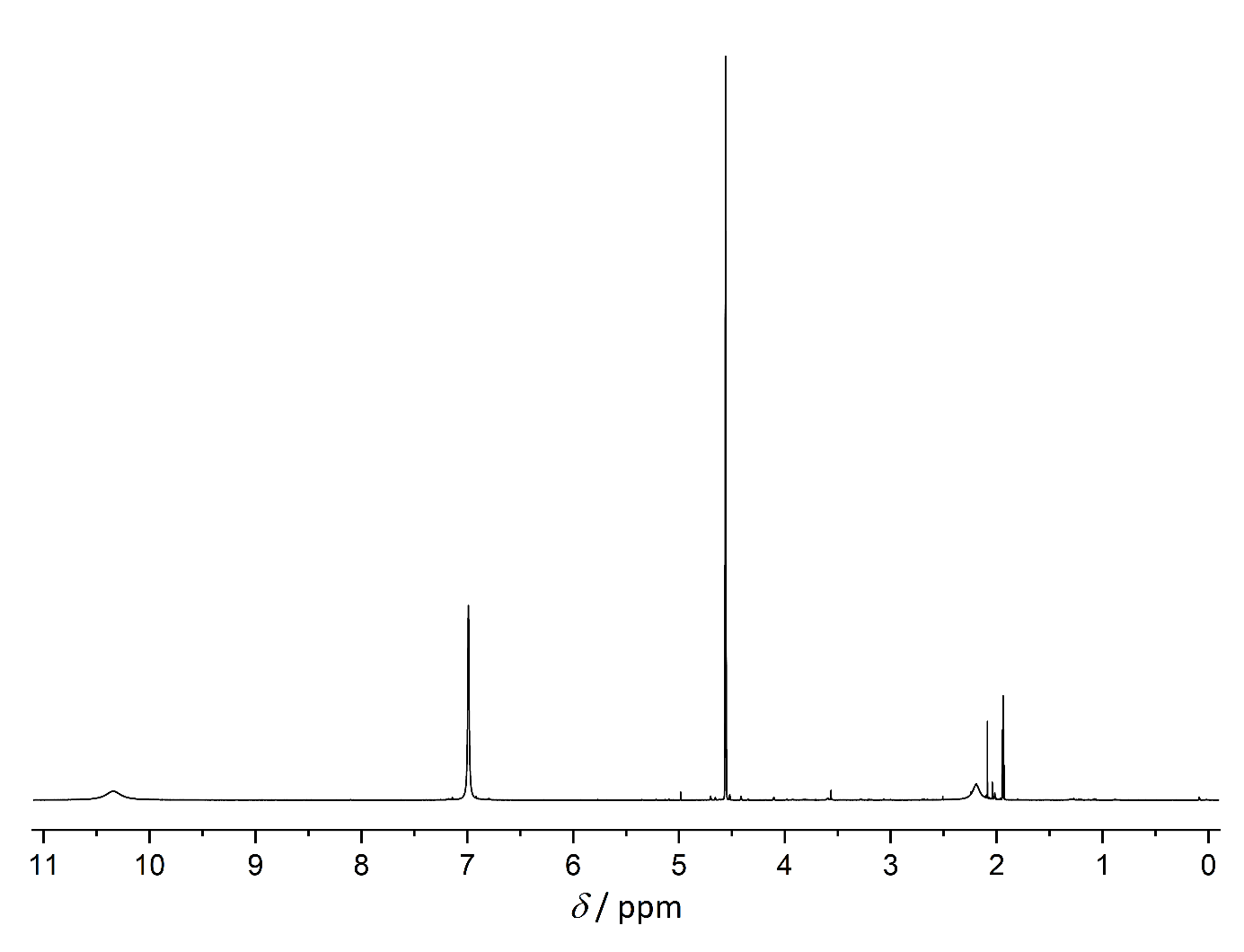


Fig. S80. ^1^H NMR spectrum of **13**.


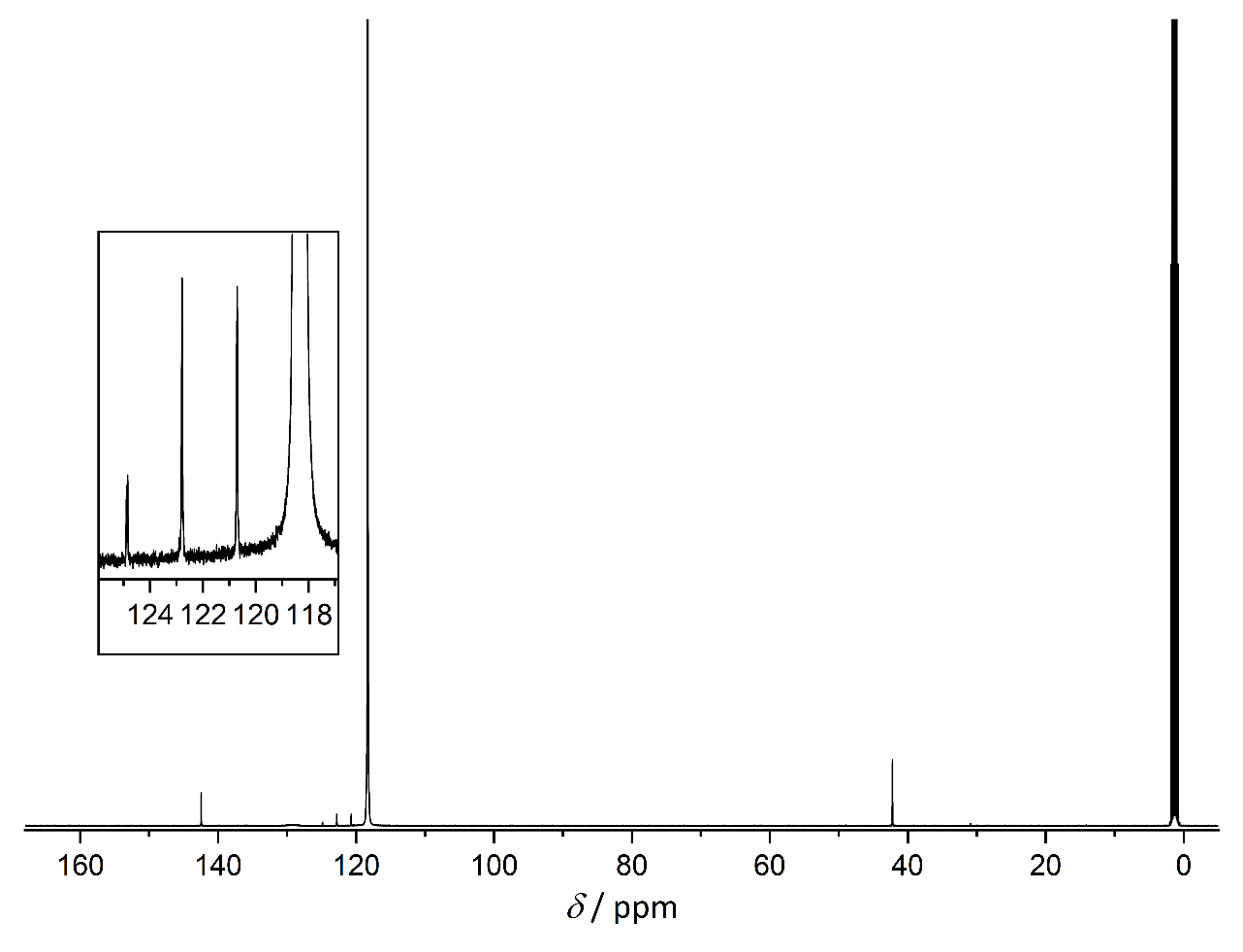


Fig. S81. ^13^C{^1^H} NMR spectrum of **13**.


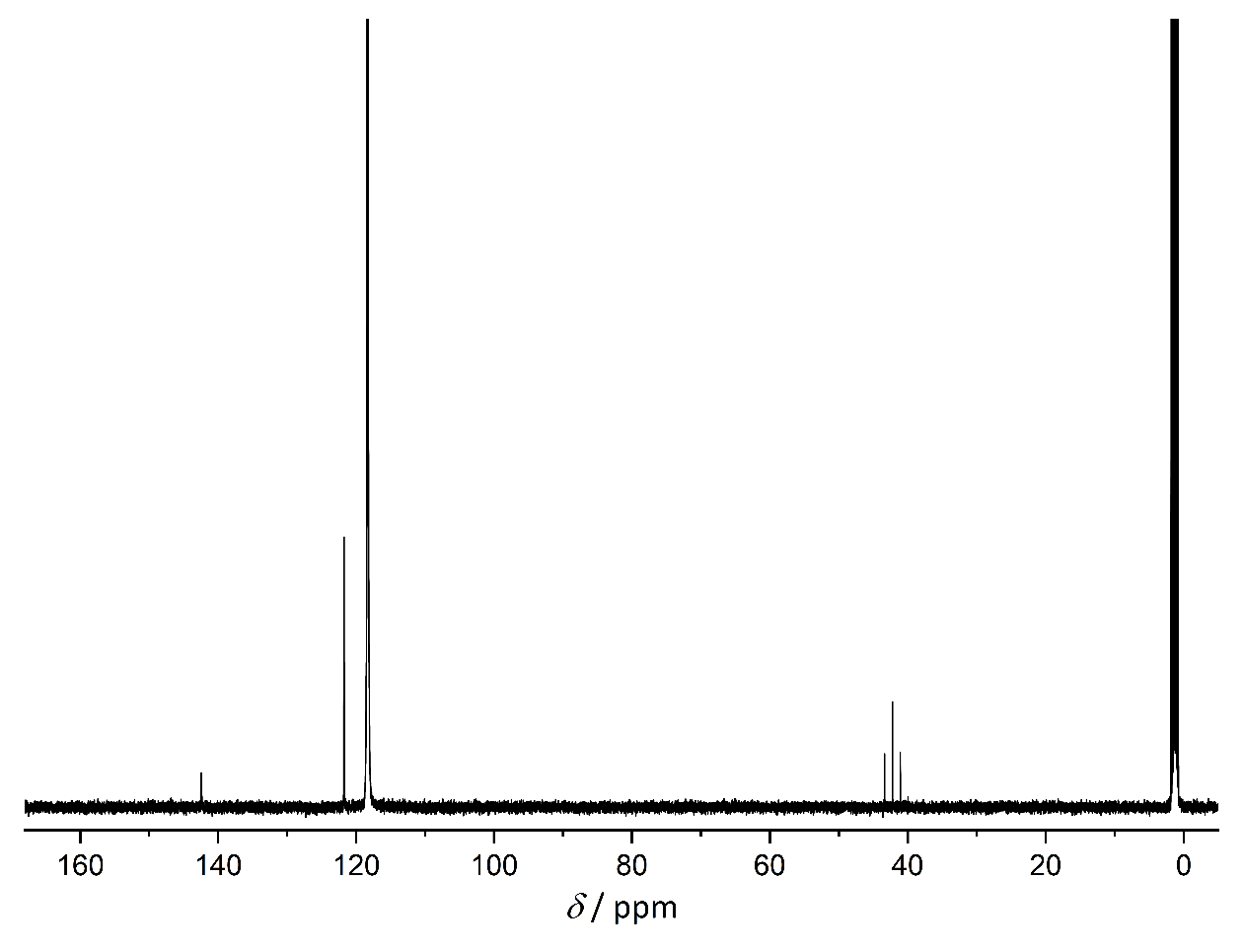


Fig. S82. ^13^C{^19^F} NMR spectrum of **13**.


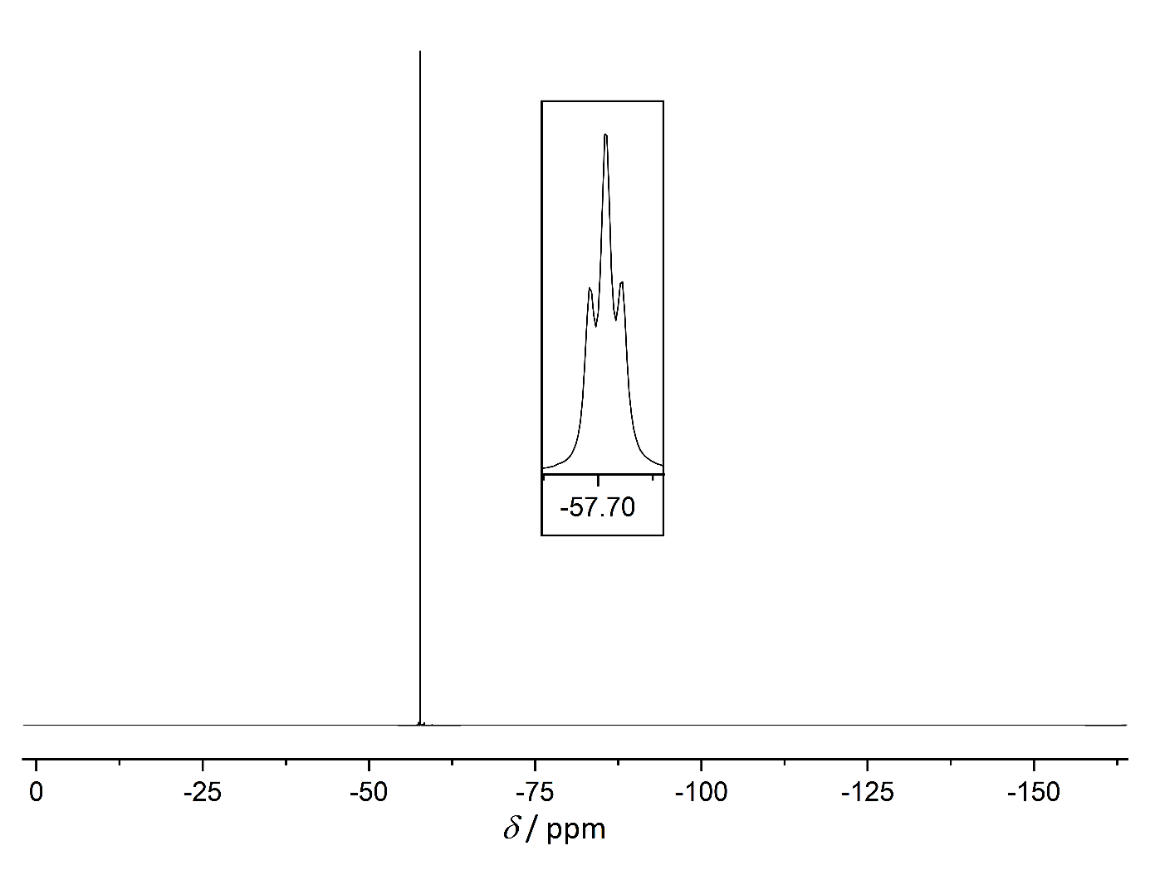


Fig. S83. ^19^F NMR spectrum of **13**.


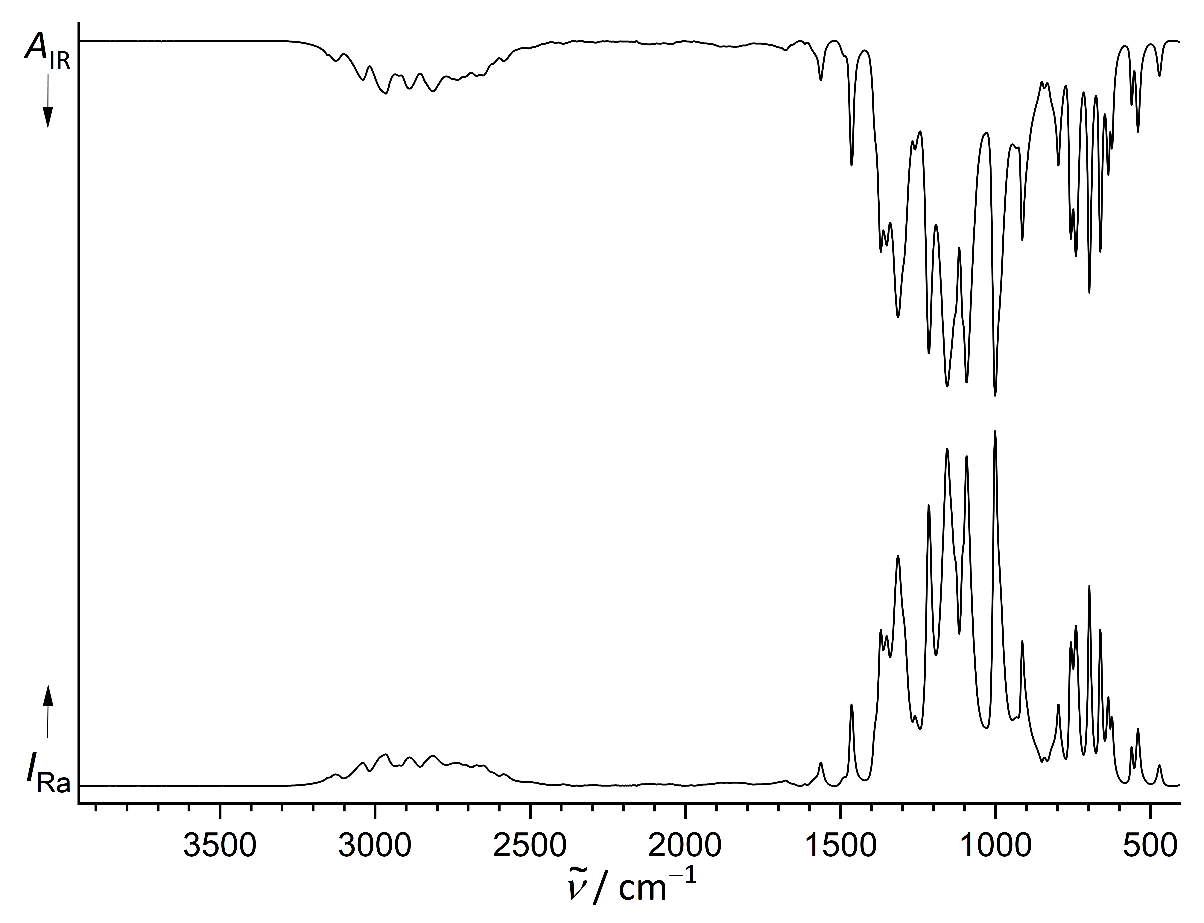


Fig. S84. IR (top) and Raman spectrum (bottom) of **13**.


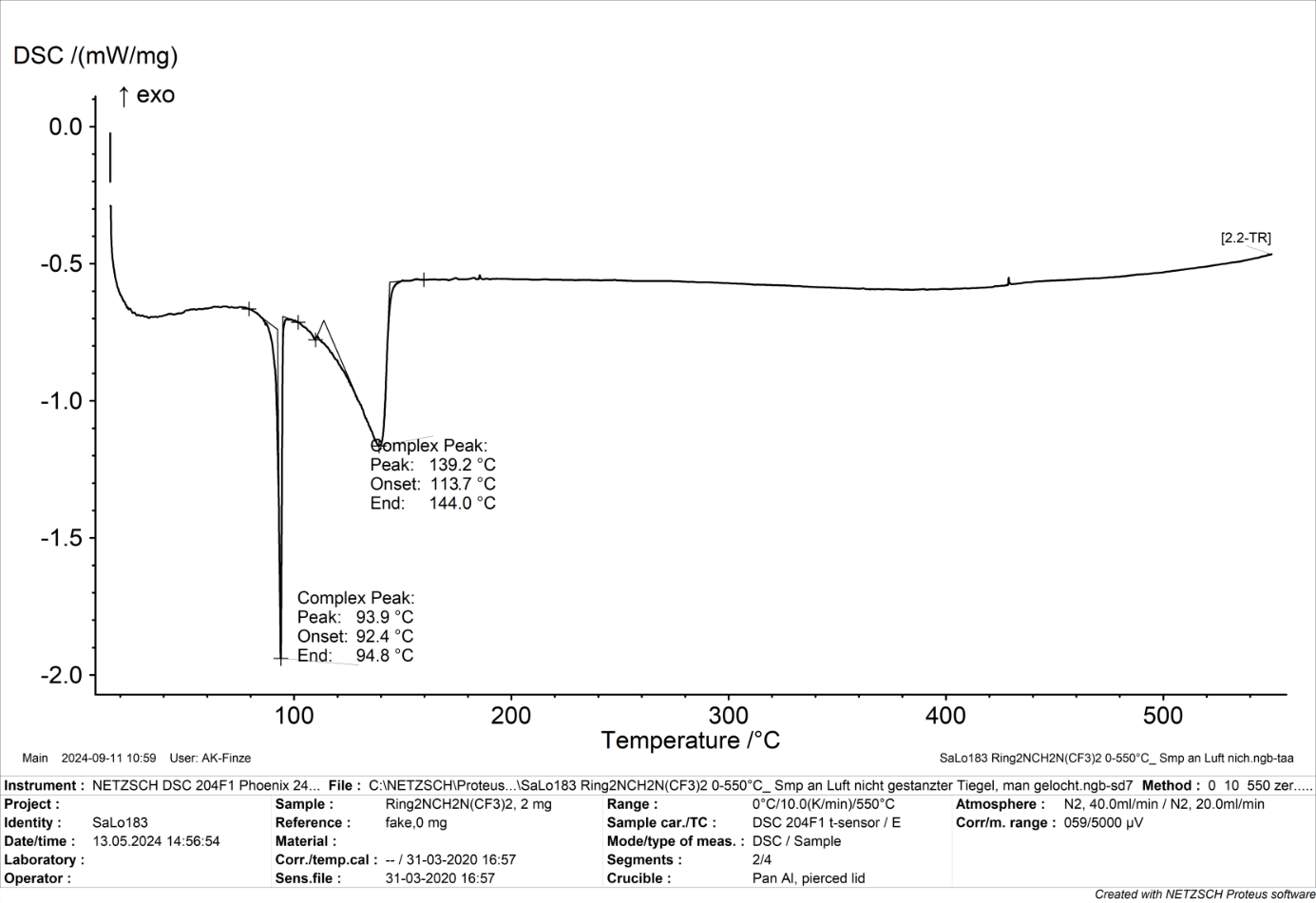


Fig. S85. DSC curve of **13**.

**(4,5-Diiodo-*1H*-imidazol-2-yl)methyl-*N*,*N*-bis(trifluoromethyl)amine (14)**

(*1H*-Imidazol-2-yl)methyl-*N*,*N*-bis(trifluoromethyl)amine (1.32 g, 5.6 mmol; **13**) was dissolved in dichloromethane (30 mL) and K2CO3 (1.90 g, 13.8 mmol) was added. Elemental iodine (2.90 g, 11.4 mmol) was added at room temperature and the deep red solution was stirred for 3.5 hours. The reaction mixture was diluted with ethyl acetate (30 mL). An aqueous solution of sodium disulfite (20%) was added until the mixture was colorless. The pH of the mixture was adjusted to 7. The organic phase was dried with MgSO_4_ and all volatiles were removed under reduced pressure to give **14** as a pale yellow solid. Yield: 2.43 g (5.01 mmol, 89%).

Elemental analysis: calculated (%) for C_6_H_3_F_6_I_2_N_3_, C 14.86, H 0.62, N 8.67; found, C 15.49, H 0.58, N 8.62.

HRMS (ASAP+) m/z, calculated for C_6_H_4_F_6_I_2_N_3_^+^: 485.8393; found: 485.886

^1^H NMR (500 MHz, CD_3_CN): *δ*= 6.11 (s, N*H*+*H_2_*O, FWHH = 45 Hz), 4.62 ppm (sept, 2H, ^4^*J*_F,H_ = 1.2 Hz, C*H_2_*N(CF_3_)_2_).

^13^C{^1^H} NMR (126 MHz, CD_3_CN): *δ* = 148.9 (s, 1C, *C*2), 121.5 (q, 2C, ^1^*J*_F,C_ = 264 Hz, *C*F_3_), 86.4 (s, 2C, *C*4), 41.5 ppm (sept, 1C, ^3^*J*_F,H_ = 1.6 Hz, *C*H_2_N(CF_3_)_2_).

^13^C{^19^F} NMR (126 MHz, CD_3_CN): *δ* = 148.9 (t, 1C, ^2^*J*_C,H_ = 5.6 Hz, *C*2), 121.5 (t, 2C, ^3^*J*_C,H_ = 4.2 Hz, *C*F_3_), 86.4 (br. s, 2C, *C*4), 41.7 ppm (t, 1C, ^1^*J*_C,H_ = 145 Hz, *C*H_2_).

^15^N NMR (^15^N-^1^H HMBC, CD_3_CN): *δ* = −296.9 (1N, *N*(CF_3_)_2_), − 159.3 ppm (2N, *N* Imidazole).

^15^N NMR (^15^N-^19^F HMQC, CD_3_CN): *δ* = −296.9 ppm (1N, *N*(CF_3_)_2_).

^19^F NMR (471 MHz, CD_3_CN): *δ* = −58.1 ppm (t, 6F, ^4^*J*_F,H_ =  1.3 Hz, C*F*_3_).


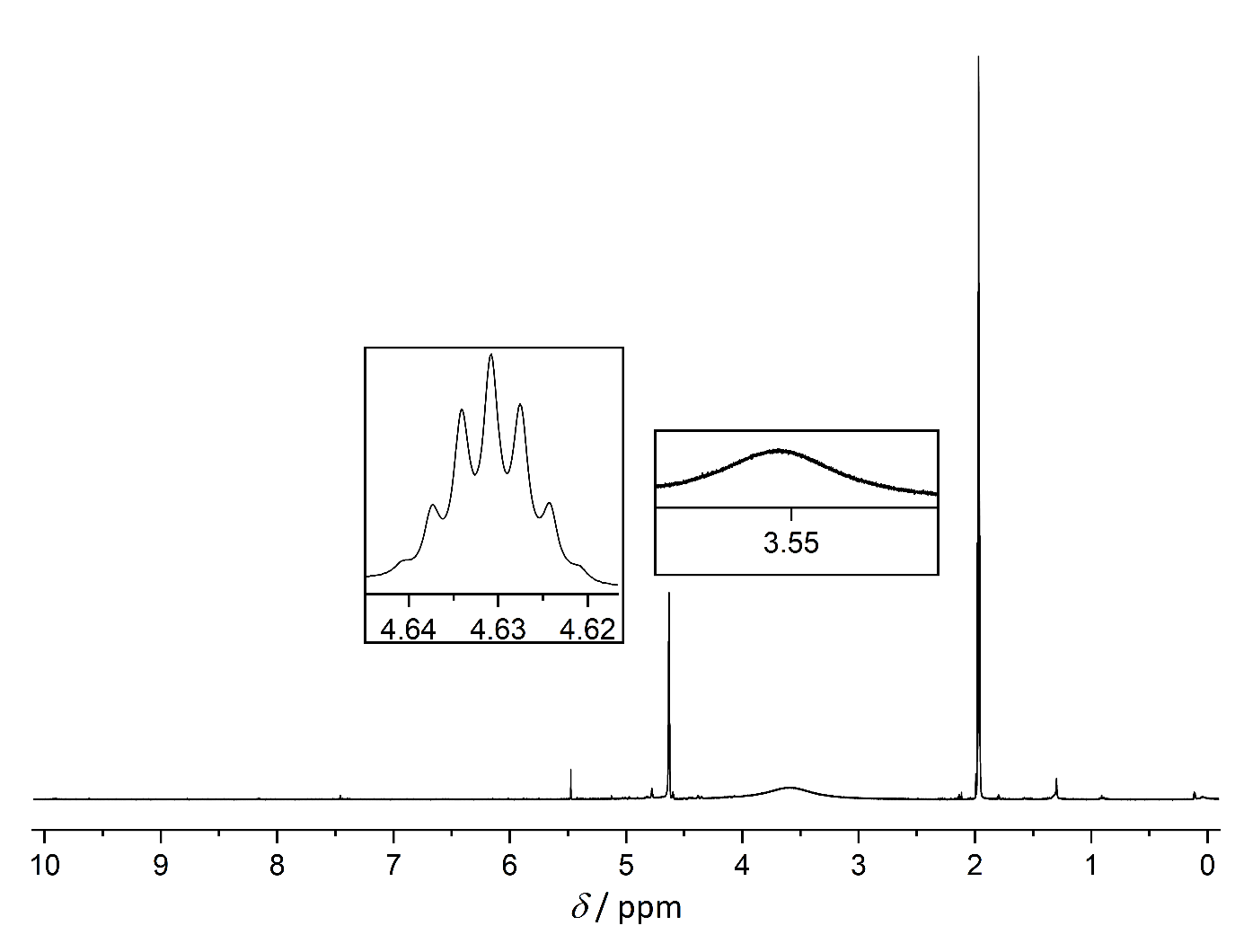


Fig. S86. ^1^H NMR spectrum of **14**.


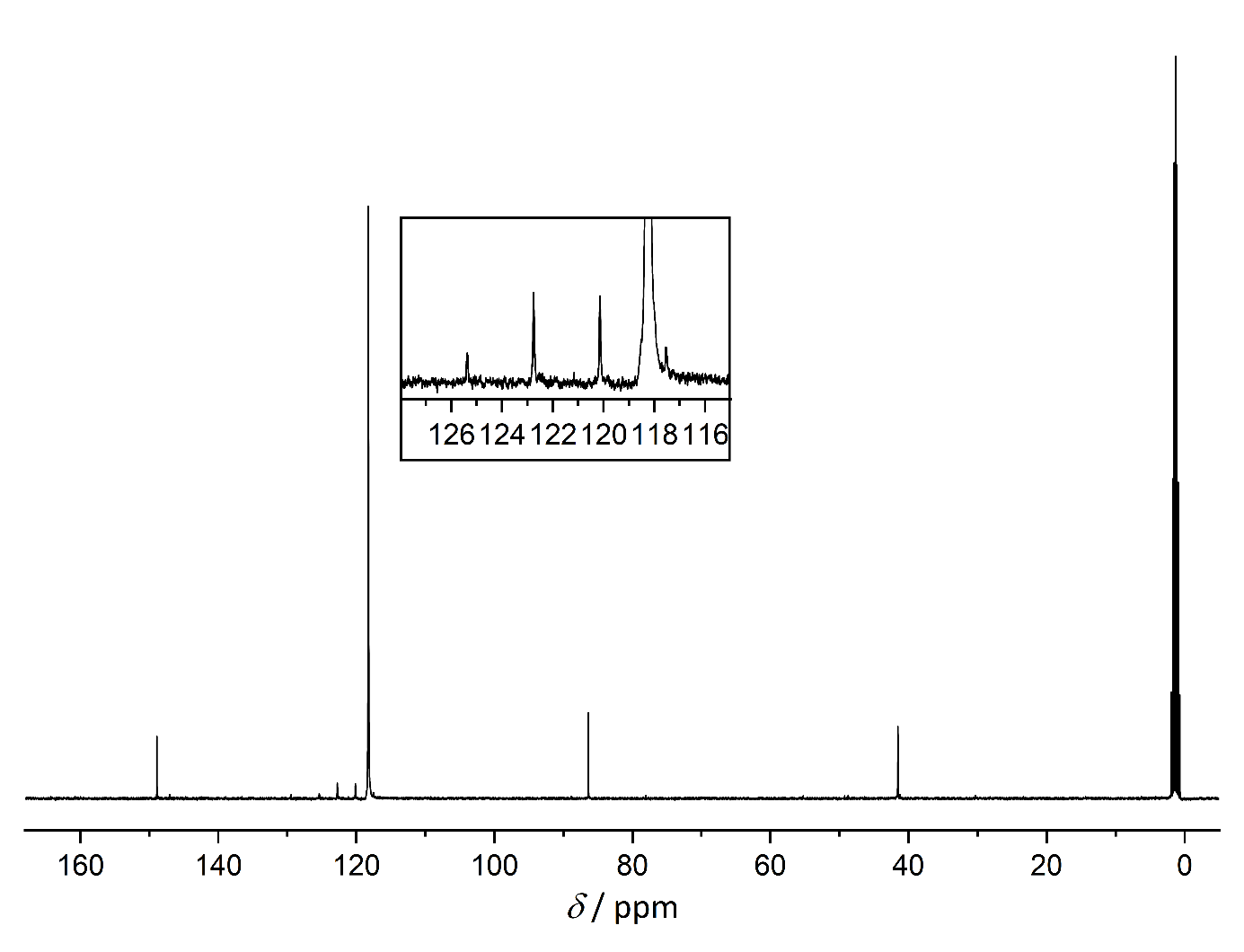


Fig. S87. ^13^C{^1^H} NMR spectrum of **14**.


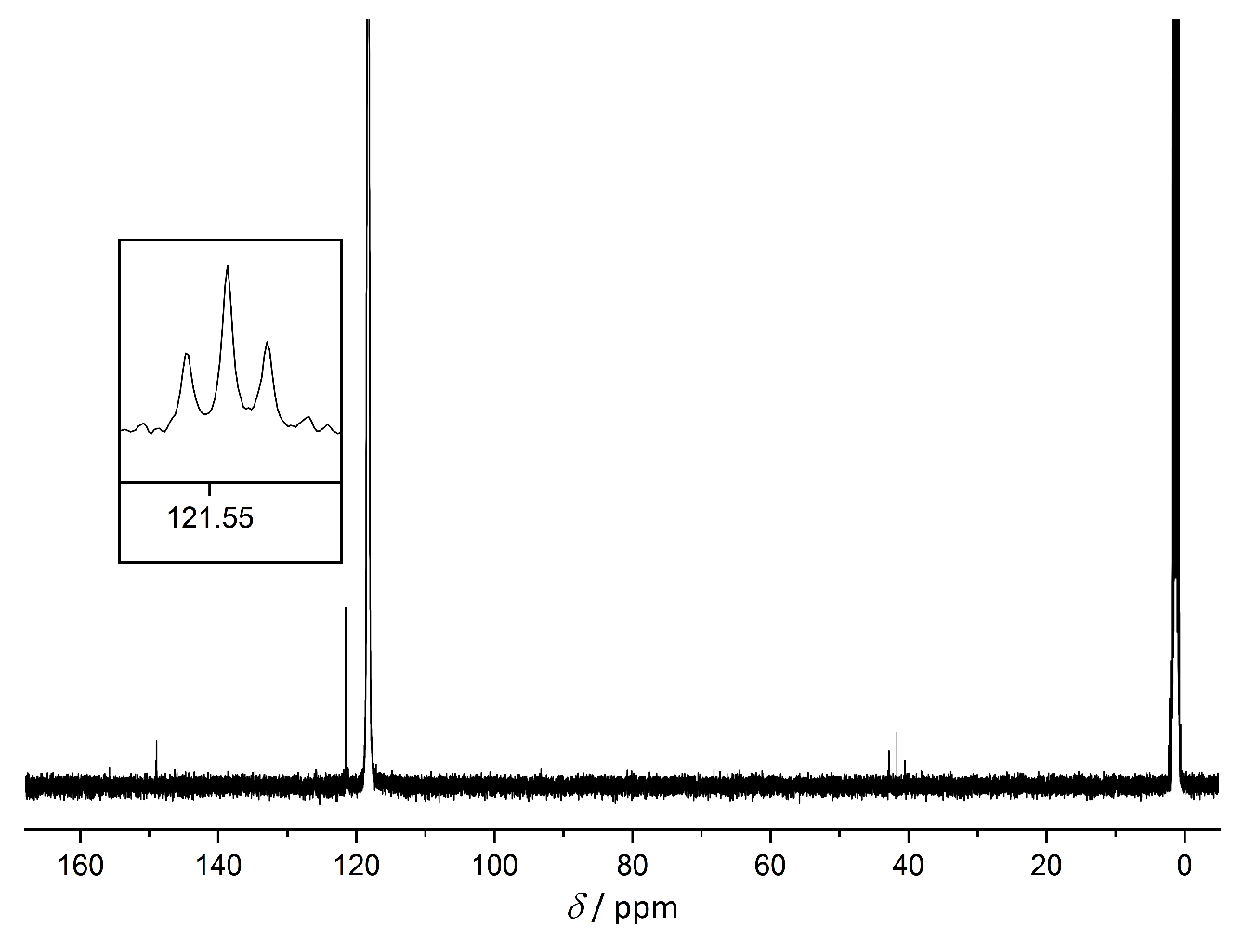


Fig. S88. ^13^C{^19^F} NMR spectrum of **14**.


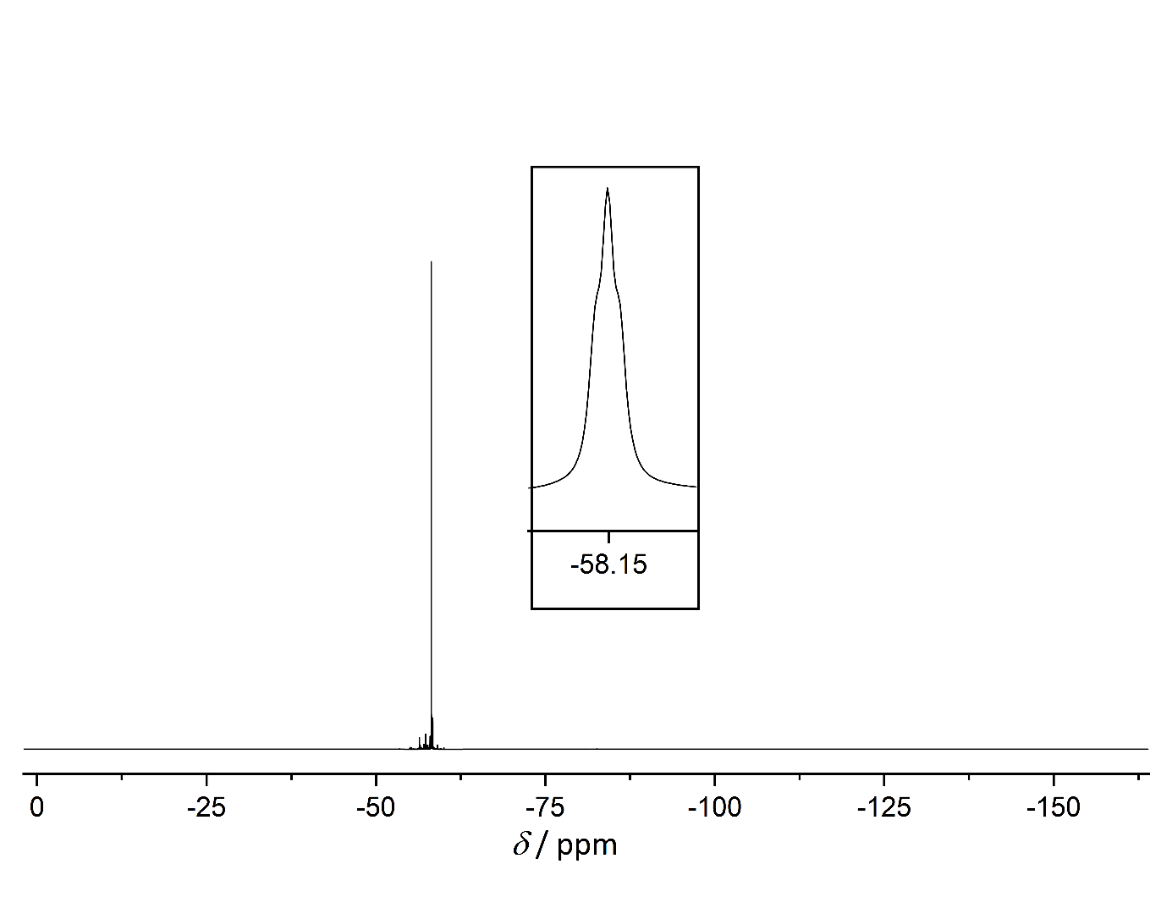


Fig. S89. ^19^F NMR spectrum of **14**.


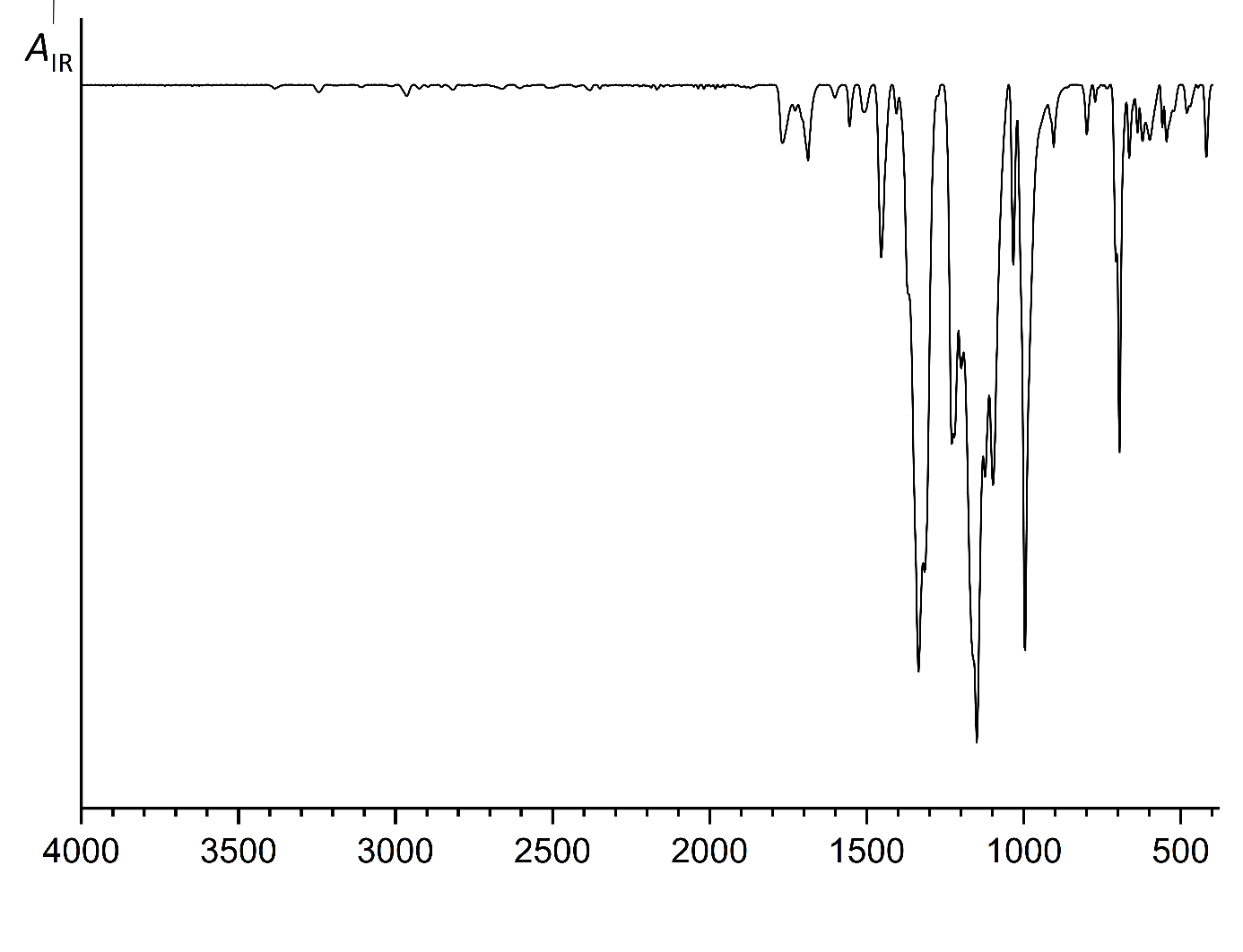
Fig. S90. IR.


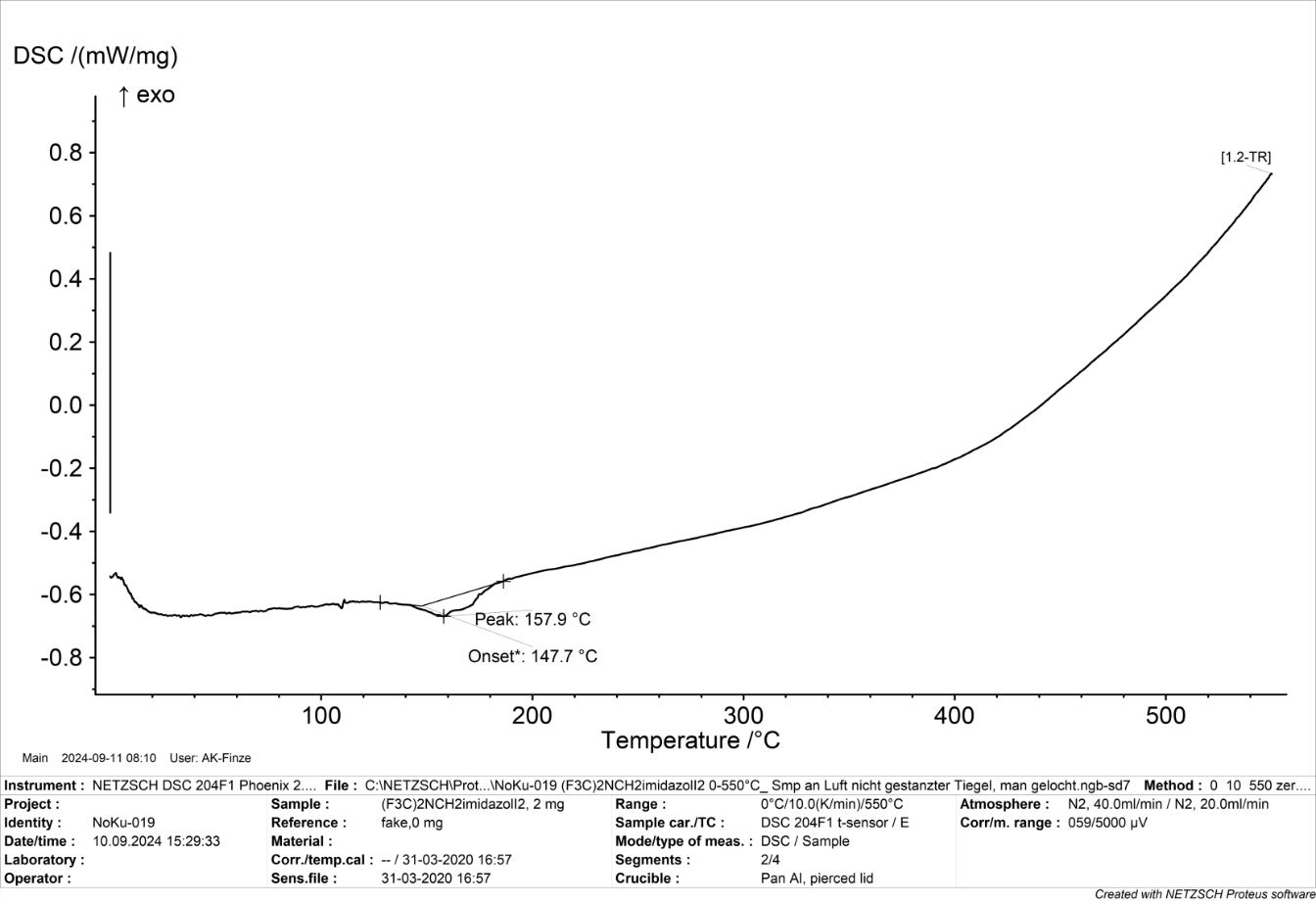
Fig. S91. DSC curve of **14**.

**Sodium *N*,*N*-bis(trifluoromethyl)aminoacetate (Na1^acetate^)**

Na{(CF_3_)_2_NCH_2_CO_2_}

Hydrochloric acid (37% v/v, 5.0 mL, 60.0 mmol) was added to *N*,*N*‑bis(trifluoromethyl)­amino­acetonitrile (**1**; 508 mg, 2.65 mmol) and the mixture was stirred at 80 °C for two days. The reaction mixture was cooled to room temperature, diethyl ether (5 mL) was added and the mixture was stirred for 30 minutes. The solid formed that had formed was removed by filtration and washed with diethyl ether (3 x 2 mL). The filtrate was separated, and the aqueous phase was extracted with diethyl ether (5 x 5 mL). The combined organic phases were dried with MgSO_4_ and most of the diethyl ether was removed at 45 °C at normal pressure. The liquid, viscous residue was characterized by ^1^H and ^19^F NMR spectroscopy. The mixture was compos­ed of (CF_3_)_2_NCH_2_C(O)OH (**1^acid^**) diethyl ether and a small amount of HF (705 mg, 31% **1^acid^** in diethyl ether, 1.02 mmol, 38% yield).

Water (0.5 mL) and diethyl ether (0.7 mL) were added to the solution of *N,N*-bis­(trifluoro­methyl)­glycine (1.02 mmol). A two-phase system was obtained, and the aqueous layer showed a pH of 1. A sodium hydroxide solution (0.375 mol L^–1^, 2.72 mL, 1.02 mmol) was slowly added until the pH reached 8. The solution was stirred 30 minutes at room temperature. All volatiles were removed in vacuum. The solid (185 mg) was taken up into acetone (3 x 2 mL) and the suspension was separated via centrifugation. The solid that was separated was identified to be NaF as assessed by ^19^F NMR spectroscopy in D_2_O. (A chloride test was negative). The liquid phase was separated, and all volatiles were removed in vacuum to yield Na**1^acetate^** (171 mg, 0.73 mmol, 28% based on **1**).

Elemental analysis: calculated (%) for C_4_H_2_F_6_NO_2_Na C 20.62, H 0.87, N 6.01; found, C 22.03, H 1.28, N 5.67.

HRMS (ESI–) m/z, calculated for C_4_H_2_F_6_NO_2_^–^: 209.999; found: 209.999.

^1^H NMR (500 MHz, d_6_-DMSO): δ = 3.55 ppm (s, 2H, C*H*_2_).

^13^C{^1^H} NMR (126 MHz, d_6_-DMSO): *δ* = 168.7 (s, 1C, *C*O), 120.6 (qm, 2C, ^1^*J*_F,C_ = 256 Hz, *C*F_3_), 47.1 ppm (s, 1C, *C*H_2_).

^13^C{^19^F} NMR (126 MHz, d_6_-DMSO): *δ* = 168.7 (s, 1C, *C*O), 120.6 (t, 2C, ^3^*J*_C,H_ = 4.1 Hz, *C*F_3_), 47.1 ppm (t, 1C, ^1^*J*_C,H_ = 141 Hz, *C*H_2_).

^15^N NMR (^19^F-^15^N HMBC, d_6_-DMSO): *δ* = −297.5 ppm (s, 1N, *N*(CF_3_)_2_).

^19^F NMR (471 MHz, d_6_-DMSO): *δ* = −54.9 ppm (t, 6F, ^4^*J*_F,H_ 1.3 Hz, C*F*_3_).


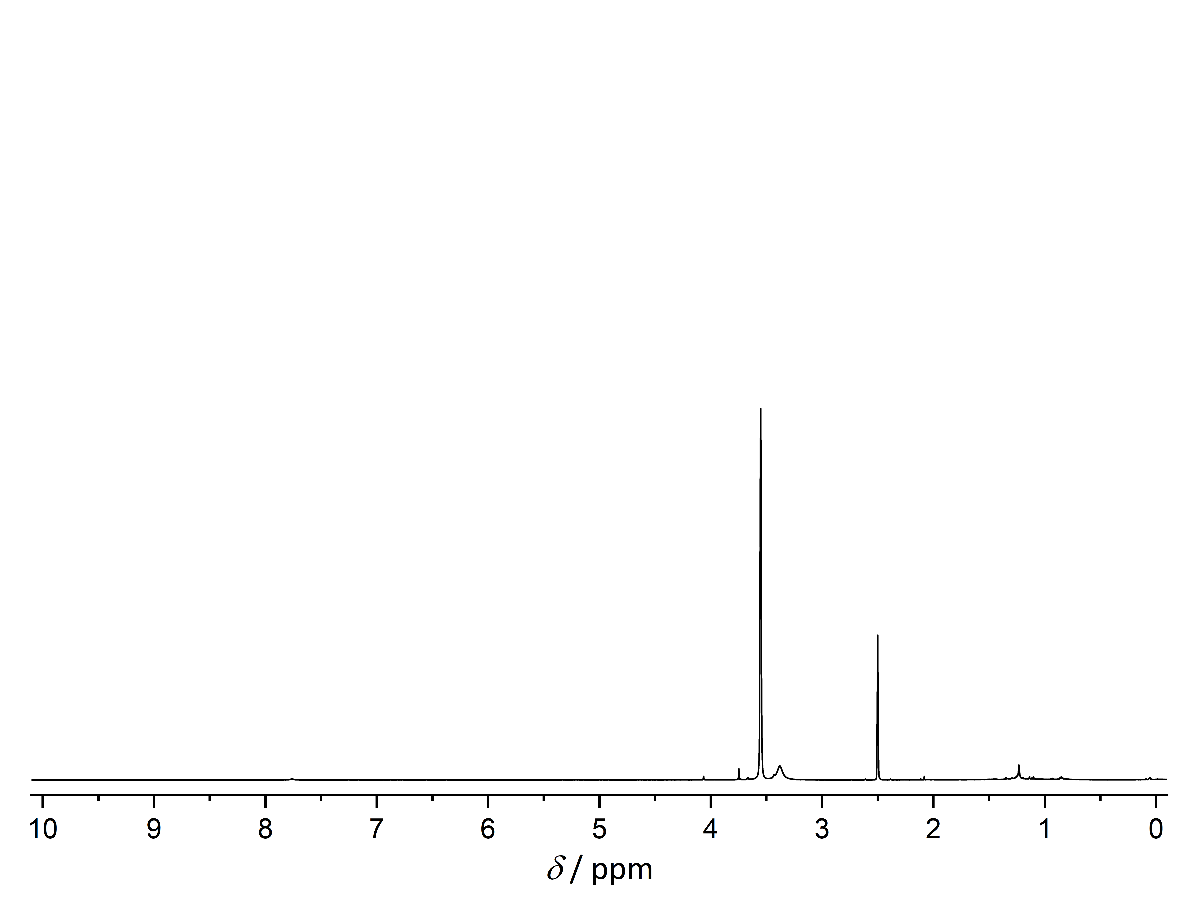


Fig. S92. ^1^H NMR spectrum of Na**1^acetate^**.


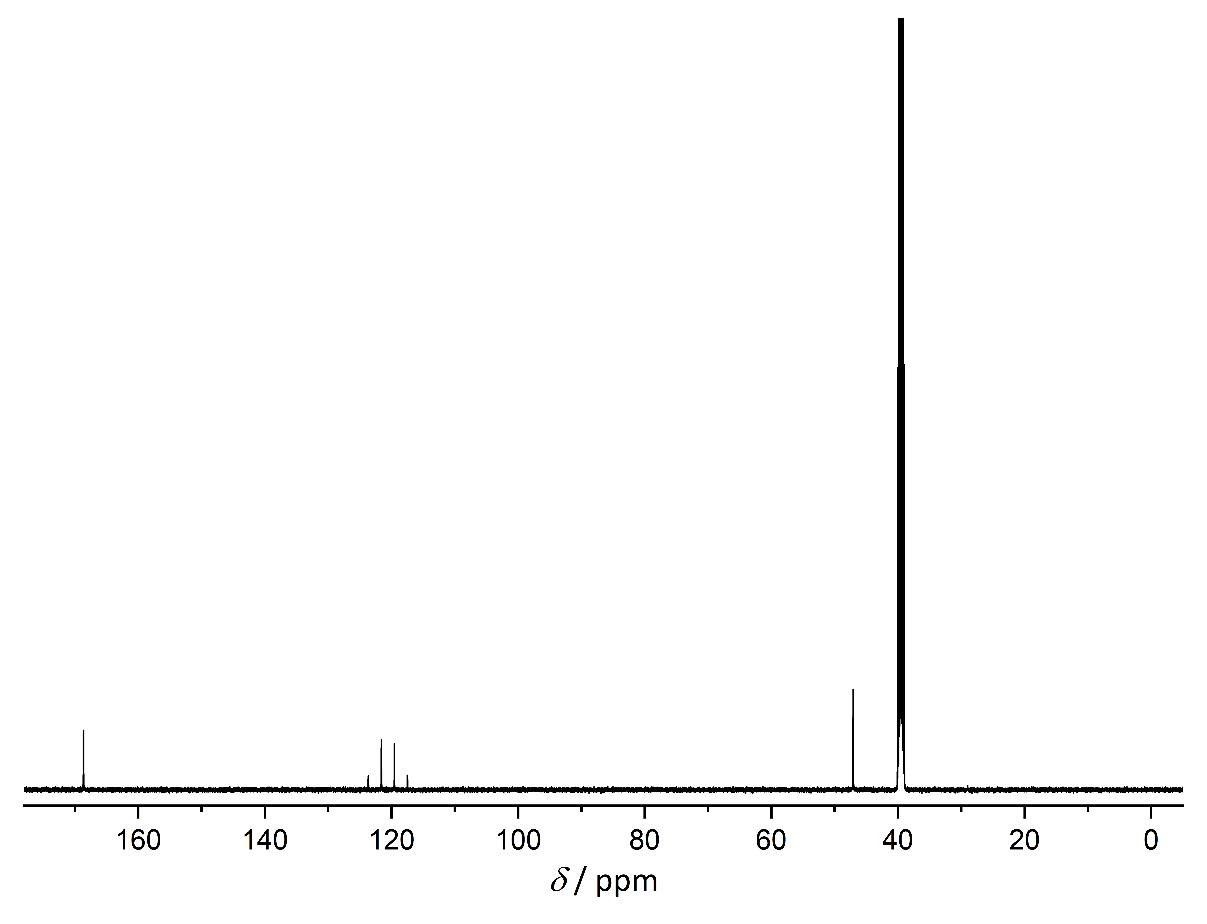


Fig. S93. ^13^C{^1^H} NMR spectrum of Na**1^acetate^**.


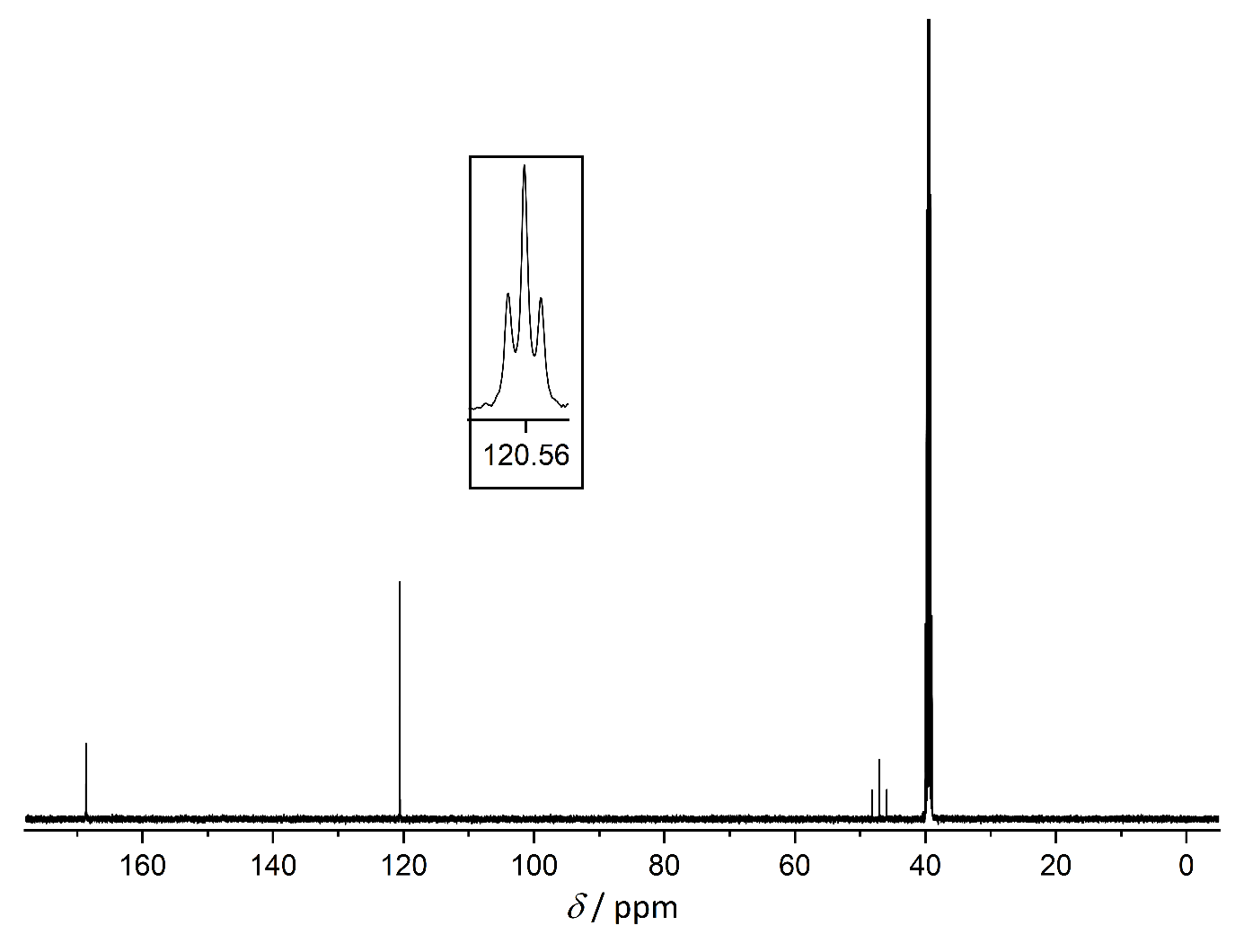


Fig. S94. ^13^C{^19^F} NMR spectrum of Na**1^acetate^**.


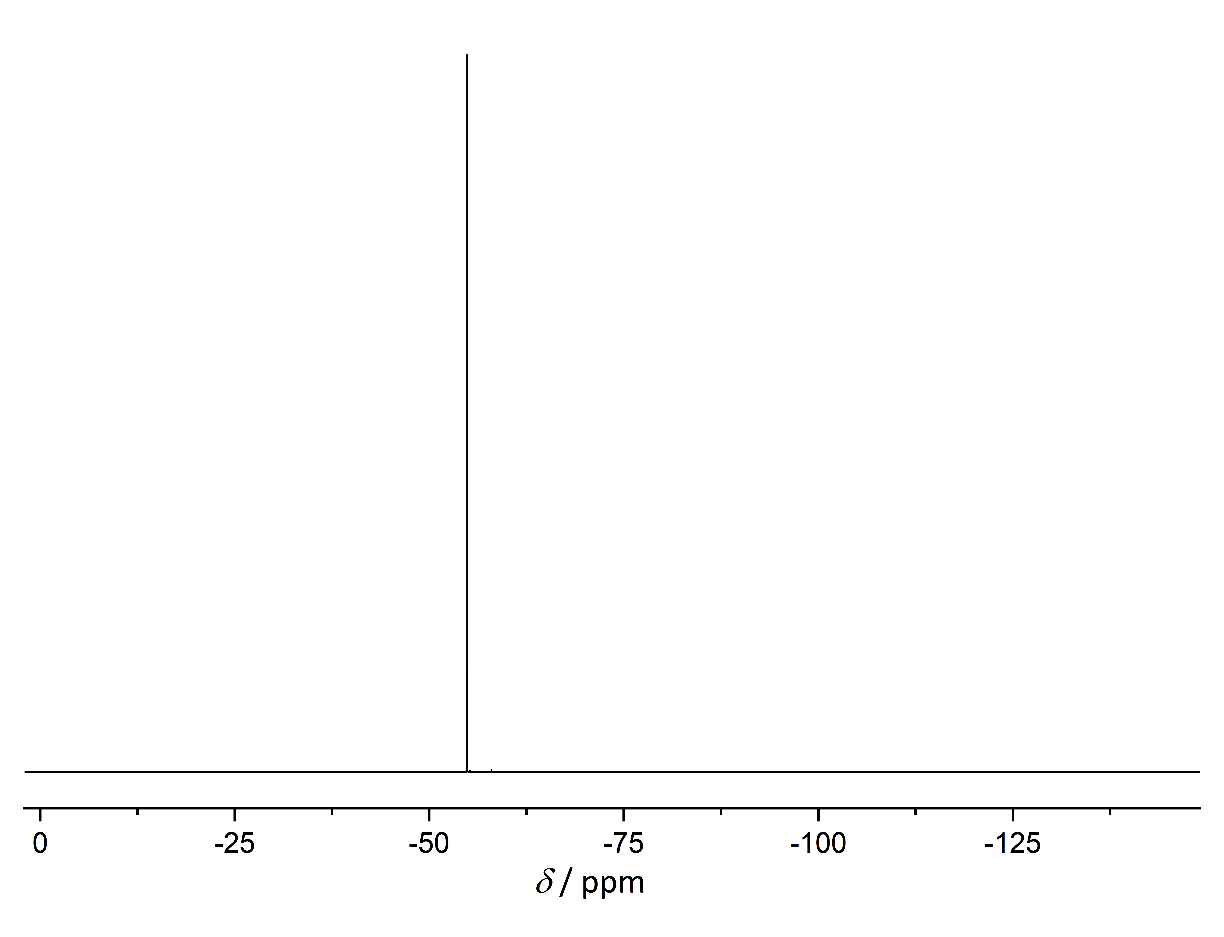


Fig. S95. ^19^F NMR spectrum of Na**1^acetate^**.


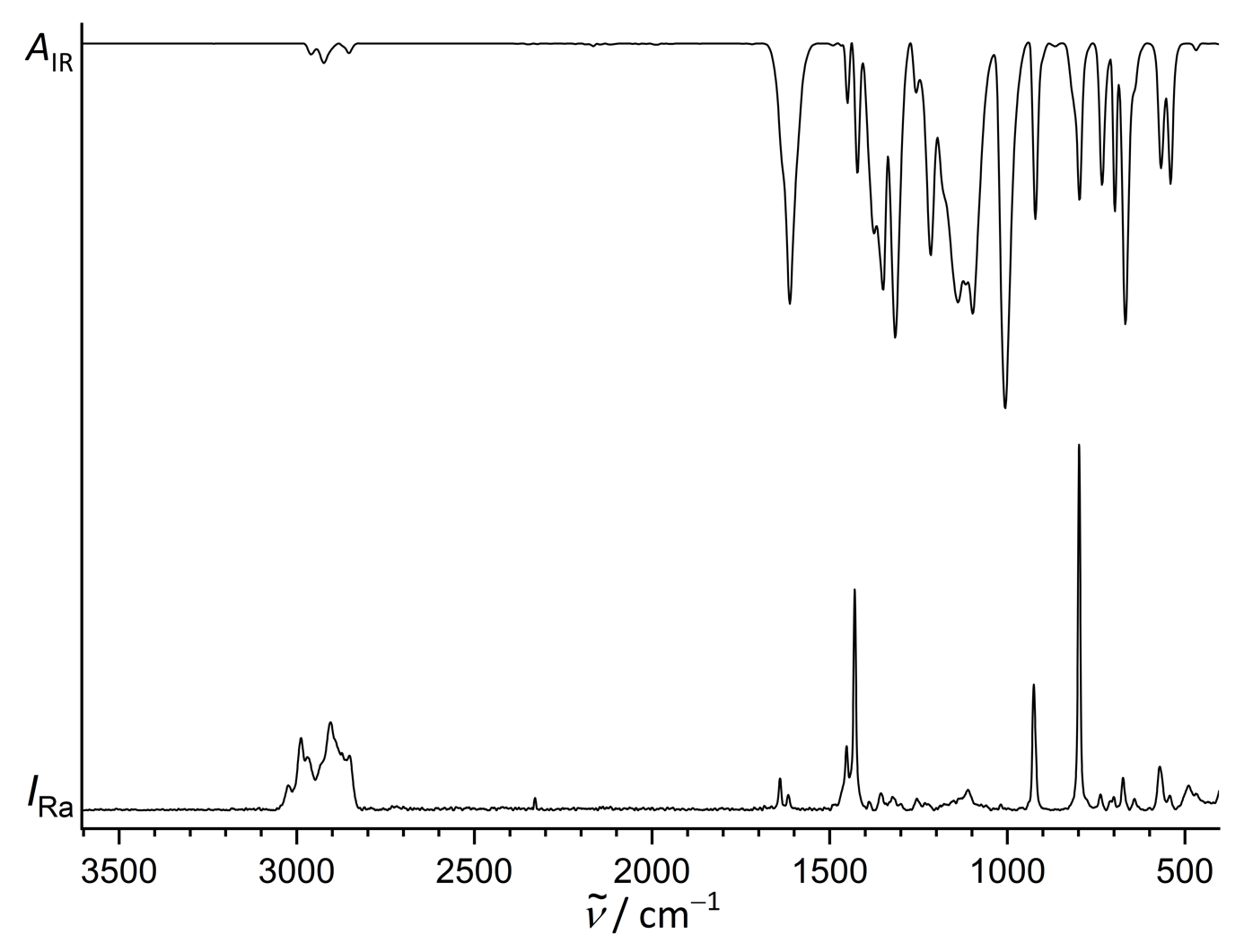


Fig. S96. IR (top) and Raman spectrum (bottom) of Na**1^acetate^**.

**5. Quantum Chemical Calculations**

Density functional calculations (DFT)^[6]^ were carried out using the B3LYP functional^[7-9]^ with the Gaussian16 program suite.^[10]^ Geometries were optimized, and energies were calculated with the def2-TZVPP basis sets.^[11]^ All calculated structures represent true minima with no imaginary frequency on the respective hypersurface. Calculated energies are summarized in Table S1.

**Table S1:** Calculated electronic energies (zero-point corrections, *E*), enthalpies (H),and free energies (*G*) at the B3LYP/def2-TZVPP level of theory.

| Species | Symmetry | *E*_0_ / a.u. | *H* / a.u. | *G* / a.u. |
| --- | --- | --- | --- | --- |
| (CF_3_)_2_NCH_2_CN (**1**) | *C*_1_ | -862.471464 | -862.459661 | -862.510051 |
| (CH_3_)_2_NCH_2_CN | *C*_s_ | -266.700996 | -266.693001 | -266.731594 |
| **2*E*** | *C*_1_ | -1285.278403 | -1285.258338 | -1285.330590 |
| **2*Z*** | *C*_1_ | -1285.276670 | -1285.256771 | -1285.327115 |
| **3*E*** | *C*_1_ | -1438.911401 | -1438.888664 | -1438.965263 |
| **3*Z*** | *C*_1_ | -1438.908716 | -1438.886172 | -1438.961304 |
| **6*E*** | *C*_1_ | -1263.226230 | -1263.206917 | -1263.276040 |
| **6*Z*** | *C*_1_ | -1263.224040 | -1263.204754 | -1263.273416 |

**6. Crystal Structure Determination**

In the case of compound **1**, which is a liquid at room temperature, a single crystal suitable for X-ray diffraction study was grown in situ by manual crystallization on the diffractometer. The general procedure was reported previously.^[12-14]^ A sample of the respective liquid was loaded into a glass capillary with an inside diameter of 0.28 mm. The filled capillary was mounted on the diffractometer and was cooled in a nitrogen gas stream of an Oxford Cryostream attachment. The following steps until data collection were monitored manually using an optical microscope with polarizer. After repeated freezing and heating of the sample resulting in a polycrystalline state, it was heated just below its melting point to slow down the nucleation. Next, the sample was heated manually with a thin copper wire to reduce the number of crystalline domains. Ideally, only one single crystal is left in the end. By slow cooling the crystal grows slowly until a single specimen fills the entire cooled area, afterwards it was cooled to the respective temperature of data collection. Crystal data was collected on a XtaLAB Synergy, Dualflex diffractometer with a hybrid pixel array detector, using Cu_Kα_ radiation (micro-focus sealed X-ray tube, λ_Cu_ = 1.54184 Å). The crystal data of compounds [Cu(**1**)_4_][BF_4_], Na**1^acetate^**, **2*E***, **3*E***, **3*Z***, **(3*Z*)_2_**, **5**, **6*E***, **8**, **9**, **10**, **11** and **13** were collected on the same system. Au_2_(**1**) was collected on a XtaLAB Synergy, Dualflex diffractometer with a hybrid pixel array detector as well but using Mo_Kα_ radiation (micro-focus sealed X-ray tube, λ_Mo_ = 0.71073 Å). Crystal data of **7**, **12** and **14** was collected on a XtaLAB Synergy-R diffractometer with a hybrid pixel array detector, using Cu_Kα_ radiation (rotating-anode X-ray tube, λ_Cu_ = 1.54184 Å). The structures were solved by intrinsic phasing methods (SHELXT).^[15-16]^ Refinement is based on full-matrix least-squares calculations on *F*^2^ (SHELXL).^[16-17]^ All non-hydrogen atoms were refined anisotropically. Unless specified differently, for CH idealized bond lengths and angles were used.

In the case of [Cu(**1**)_4_][BF_4_] the data was incomplete due to a degradation of the crystal during measurement. Therefore, it was necessary to use DFIX, SIMU, ISOR, EADP as well as EADX restrains. The benzene rings in **Au_2_(1)** were flattened and equalized using SADI, FLAT, SIMU, RIGU and SAME restrains. **2*E*** was refined as a 2-component twin with the second component rotated by −179.9875 degrees around [0.99 −0.00 −0.11] (reciprocal) or [1.00 −0.00 −0.00] (direct). The N(CF_3_)_2_ group is twofold disordered and was restrained with SIMU and RIGU. The 1,2-distances between C and F of the CF_3_ groups were restrained using SADI. The N(CF_3_)_2_ moiety **3*E*** was disordered, thus SIMU, RIGU and SADI restrains were introduced to ensure a stable refinement of this unit. Dimer **(3Z)_2_** showed a disorder at one of the CF_3_ groups, thus SIMU and RIGU restrains were introduced here as well. Compound **8** and **9** both showed a disorder of both CF_3_ groups. SIMU, RIGU and SADI restrains were used to resolve this issue. One CF_3_ group of **12** was also disordered, so SIMU, RIGU, EADP and EXYZ restrains were necessary to compensate for this. As for component **14** SIMU and ISOR restrains had to be introduced to ensure a stable refinement.

Calculations were carried out using the ShelXle graphical interface.^[18]^ Molecular structure diagrams were drawn with the program Diamond 5.0.0.^[19]^

Crystallographic data have been deposited with the Cambridge Crystallographic Data Centre. Experimental details, crystal data, and CCDC numbers are collected in Tables S2 and S3. These data can be obtained free of charge from The Cambridge Crystallographic Data Centre via [www.ccdc.cam.ac.uk/data_request/cif](http://www.ccdc.cam.ac.uk/data_request/cif).

**Table S2.** Crystal data and structure refinement details of **1**, [Cu(**1**)_4_][BF_4_], **Au_2_(1)**, **2*E***, **3*E***, **3*Z***, **(3*Z*)_2_**, **5**, **6*E*** and **7**.

| compound | **1** | [Cu(**1**)_4_][BF_4_] | **Au_2_1** | Na**1^acid^** | **2*E*** | **3*E*** | **3*Z*** | **(3*Z*)_2_** | **5** |
| --- | --- | --- | --- | --- | --- | --- | --- | --- | --- |
|  |  |  |  |  |  |  |  |  |  |
| empirical formula | C_4_H_2_F_6_N_2_ | C_16_H_8_CuF_28_N_8_B | C_40_H_30_Au_2_F_6_N_2_P_2_ | C_8_H_4_F_12_N_2_Na_2_O_4_ | C_15_H_8_F_6_N_2_ | C_19_H_10_F_6_N_2_ | C_19_H_10_F_6_N_2_ | C_38_H_20_F_12_N_4_ | C_17_H_8_F_8_N_2_ |
| *M*_W_ [g mol^–1^] | 192.08 | 918.60 | 1108.53 | 466.11 | 330.23 | 380.29 | 380.29 | 760.58 | 392.25 |
| colour | colourless | colourless | colourless | colourless | colourless | yellow | yellow | yellow | colourless' |
| *T* [K] | 100 | 100 | 100 | 100 | 100 | 100 | 100 | 100 | 100 |
| crystal system | tetragonal | monoclinic | monoclinic | triclinic | monoclinic | monoclinic | monoclinic | triclinic | monoclinic |
| space group | *P*4_2_/*n* | *Pn* | *Cc* | *P*-1 | *P*2_1_/*c* | *P*2_1_/*n* | *P*2_1_/*c* | *P*–1 | *P*2_1_/*c* |
| *a* [Å] | 15.5380(2) | 10.2505(2) | 19.2987(7) | 6.48140(10) | 6.6743(2) | 14.02940(10) | 11.06180(10) | 7.7154(2) | 10.8304(3) |
| *b* [Å] | 15.5380(2) | 25.3144(9) | 9.4973(3) | 9.2345(2) | 7.0570(2) | 6.87970(10) | 12.05290(10) | 10.3071(3) | 9.0027(2) |
| *c* [Å] | 5.5418(2) | 24.3933(4) | 21.0588(8) | 12.6636(2) | 29.1353(9) | 17.3701(2) | 13.1388(2) | 10.8709(3) | 16.9782(4) |
| *α* [°] |  |  |  | 88.528(2) |  |  |  | 83.254(2) |  |
| *β* [°] |  | 101.988(2) | 103.289(4) | 75.539(2) | 91.513(3) | 100.1600(10) | 105.7400(10) | 82.271(2) | 108.438(3) |
| *γ* [°] |  |  |  | 88.214(2) |  |  |  | 69.000(2) |  |
| *V* [Å^3^] | 1337.95(6) | 6191.7(3) | 3756.4(2) | 733.45(2) | 1371.81(7) | 1650.24(3) | 1686.07(3) | 797.49(4) | 1570.44(7) |
| Z | 8 | 2 | 4 | 2 | 4 | 4 | 4 | 1 | 4 |
| *ρ*_calcd_ [Mg m^–3^] | 1.907 | 1.971 | 1.960 | 2.111 | 1.599 | 1.531 | 1.498 | 1.584 | 1.659 |
| *μ* [mm^–1^] | 2.209 | 2.879 | 7.949 | 2.874 | 1.367 | 1.224 | 1.198 | 1.266 | 1.493 |
| *F*(000) [e] | 752 | 3568 | 2104 | 456 | 664 | 768 | 768 | 384 | 784 |
| *θ* range [°] | 4.024–77.106 | 2.545–52.125 | 2.169–30.937 | 3.605–78.067 | 3.035–73.816 | 3.743–76.819 | 4.152–76.954 | 4.116–77.073 | 5.493–77.732 |
| reflections collected | 10266 | 11885 | 21719 | 40677 | 3932 | 30673 | 31668 | 15141 | 16333 |
| independent reflections [*I* > 2*σ*(*I*)] | 1367 | 8232 | 7316 | 2834 | 3932 | 3415 | 3520 | 3225 | 3214 |
| *R*(int) | 0.0799 | 0.0282 | 0.0554 | 0.0221 | 0.0722 | 0.0315 | 0.0371 | 0.0457 | 0.0589 |
| data / restraints / parameters | 1367 / 0 / 115 | 8232 / 378 / 2068 | 8424 / 1715 / 283 | 3093 / 365 / 582 | 3932 / 429 / 291 | 3415 / 433 / 326 | 3520 / 0 / 274 | 3225 / 30 / 303 | 3214 / 0 / 244 |
| *R*1 [*I* > 2*σ*(*I*)] | 0.0393 | 0.0609 | 0.0437 | 0.0385 | 0.0577 | 0.0336 | 0.0384 | 0.0452 | 0.0414 |
| *wR*2 (all data) | 0.1076 | 0.1278 | 0.1002 | 0.1090 | 0.1686 | 0.0982 | 0.1102 | 0.1224 | 0.1137 |
| GOF on *F*^2^ | 1.073 | 1.239 | 1.038 | 1.077 | 1.097 | 0.883 | 1.054 | 1.032 | 1.036 |
| largest diff. peak / hole [e Å^–3^] | 0.277 / −0.233 | 0.613 / −0.391 | 2.863 / −1.668 | 0.420 / –0.276 | 0.266 / −0.375 | 0.199 / −0.274 | 0.297 / −0.277 | 0.296 / −0.275 | 0.262 / −0.262 |
| CCDC number | 2409465 | 2409480 | 2409481 | 2443819 | 2409466 | 2409467 | 2409468 | 2409469 | 2409470 |

**Table S3.** Crystal data and structure refinement details of **8**, **9**, **10**, **11**, **12**, **13** and **14**.

| compound | **6*E*** | **7** | **8** | **9** | **10** | **11** | **12** | **13** | **14** |
| --- | --- | --- | --- | --- | --- | --- | --- | --- | --- |
|  |  |  |  |  |  |  |  |  |  |
| empirical formula | C_13_H_7_F_6_N_3_ | C_17_H_9_N | C_6_H_7_F_6_N_3_ | C_7_H_9_F_6_N_3_ | C_10_H_13_F_6_N_3_ | C_6_H_8_F_6_N_2_O_2_ | C_4_H_3_F_6_N_5_ | C_6_H_5_F_6_N_3_ | C_6_H_3_F_6_I_2_N_3_ |
| *M*_W_ [g mol^–1^] | 319.22 | 227.25 | 235.15 | 249.17 | 289.23 | 254.14 | 235.11 | 233.13 | 484.91 |
| colour | colourless | yellow | colourless | colourless | colourless | colourless | colourless | colourless | colourless |
| *T* [K] | 100 | 100 | 100 | 100 | 100 | 100 | 100 | 100 | 100 |
| crystal system | monoclinic | monoclinic | monoclinic | monoclinic | orthorhombic | tetragonal | monoclinic | monoclinic | monoclinic |
| space group | *P*2_1_/*c* | *Cc* | *I*2/*a* | *P*2_1_/*c* | *Pbca* | *P*–4 | *P*2_1_/*c* | *P*2_1_/*c* | *Cc* |
| *a* [Å] | 11.09600(10) | 18.4257 | 9.60130(10) | 9.00450(10) | 9.7985(2) | 14.2766(2) | 13.25040(10) | 13.4719(4) | 13.6128(2) |
| *b* [Å] | 5.68060(10) | 3.7656 | 17.7570(2) | 10.75000(10) | 13.7787(2) | 14.2766(2) | 6.45240(10) | 6.74150(10) | 13.5985(2) |
| *c* [Å] | 20.9622(3) | 16.7955 | 10.6844(2) | 10.07510(10) | 17.8068(3) | 4.73110(10) | 9.55050(10) | 10.0423(2) | 25.5927(2) |
| *α* [°] |  |  |  |  |  |  |  |  |  |
| *β* [°] | 100.1600(10) | 107.060(7) | 98.6080(10) | 100.9160(10) |  |  | 95.9870(10) | 110.969(3) | 90.0640(10) |
| *γ* [°] |  |  |  |  |  |  |  |  |  |
| *V* [Å^3^] | 1300.57(3) | 1114.06(12) | 1801.07(4) | 957.607(17) | 2404.11(7) | 964.30(3) | 812.084(16) | 851.65(4) | 4737.56(11) |
| Z | 4 | 4 | 8 | 4 | 8 | 4 | 4 | 4 | 16 |
| *ρ*_calcd_ [Mg m^–3^] | 1.630 | 1.355 | 1.734 | 1.728 | 1.598 | 1.751 | 1.923 | 1.818 | 2.719 |
| *μ* [mm^–1^] | 1.439 | 0.613 | 1.799 | 1.730 | 1.469 | 1.861 | 2.076 | 1.902 | 42.367 |
| *F*(000) [e] | 640 | 472 | 944 | 504 | 1184 | 512 | 464 | 464 | 3520 |
| *θ* range [°] | 4.048–77.778 | 5.022–74.668 | 4.871–77.693 | 5.002–77.484 | 4.967–80.290 | 3.095–76.935 | 3.354–74.770 | 3.513–76.884 | 3.454–74.987 |
| reflections collected | 35137 | 9808 | 26377 | 28530 | 31842 | 18421 | 27526 | 15080 | 80903 |
| independent reflections [*I* > 2*σ*(*I*)] | 2743 | 2122 | 1919 | 2040 | 2613 | 1991 | 1601 | 1733 | 9060 |
| *R*(int) | 0.0611 | 0.0573 | 0.0448 | 0.0504 | 0.0945 | 0.0738 | 0.0410 | 0.0373 | 0.0825 |
| data / restraints / parameters | 2743 / 0 / 202 | 2122 / 2 / 163 | 1919 / 278 / 214 | 2040 / 302 / 223 | 2613 / 0 / 176 | 1991 / 0 / 151 | 1601 / 21 / 167 | 1733 / 0 / 151 | 9060 / 182 / 614 |
| *R*1 [*I* > 2*σ*(*I*)] | 0.0406 | 0.0603 | 0.0321 | 0.0288 | 0.0612 | 0.0742 | 0.0294 | 0.0362 | 0.0648 |
| *wR*2 (all data) | 0.1106 | 0.1688 | 0.0888 | 0.0791 | 0.1641 | 0.1683 | 0.0780 | 0.1002 | 0.1627 |
| GOF on *F*^2^ | 1.069 | 1.007 | 1.057 | 1.045 | 1.125 | 2.069 | 1.039 | 1.067 | 1.068 |
| largest diff. peak / hole [e Å^–3^] | 0.229 / −0.315 | 0.317 / −0.217 | 0.254 / −0.259 | 0.221 / −0.248 | 0.441 / −0.381 | 0.569 / –0.329 | 0.221 / −0.261 | 0.275 / −0.235 | 2.613 / –2.411 |
| CCDC number | 2409471 | 2409472 | 2409473 | 2409474 | 2409475 | 2409476 | 2409477 | 2409478 | 2409479 |

**7. References**

[1] R. K. Harris, E. D. Becker, S. M. Cabral de Menezes, R. Goodfellow, P. Granger, *Pure Appl. Chem.* **2001**, *73*, 1795–1818.

[2] G. R. Fulmer, A. J. M. Miller, N. H. Sherden, H. E. Gottlieb, A. Nudelman, B. M. Stoltz, J. E. Bercaw, K. I. Goldberg, *Organometallics* **2010**, *29*, 2176–2179.

[3] P. Sartori, N. Ignat’ev, S. Datsenko, *J. Fluorine Chem.* **1995**, *75*, 157–161.

[4] N. Ignat'ev, in *Modern Synthesis Processes and Reactivity of Fluorinated Compounds* (Eds.: H. Groult, F. Leroux, A. Tressaud), Elsevier Inc., London, UK, **2016**.

[5] T. Moriya, K. Hagio, N. Yoneda, *Chem. Pharm. Bull.* **1980**, *28*, 1711–1721.

[6] W. Kohn, L. J. Sham, *Physical Review A* **1965**, *140*, 1133–1138.

[7] A. D. Becke, *Physical Review A* **1988**, *38*, 3098–3100.

[8] A. D. Becke, *Journal of Chemical Physics* **1993**, *98*, 5648–5652.

[9] C. Lee, W. Yang, R. G. Parr, *Physical Review B: Condensed Matter* **1988**, *37*, 785–789.

[10] Gaussian 16 Revision A.03, M. J. Frisch, G. W. Trucks, H. B. Schlegel, G. E. Scuseria, M. A. Robb, J. R. Cheeseman, G. Scalmani, V. Barone, G. A. Petersson, H. Nakatsuji, X. Li, M. Caricato, A. V. Marenich, J. Bloino, B. G. Blanesko, R. Gomperts, B. Mennucci, H. P. Hratchian, J. V. Ortiz, A. F. Izmaylov, J. L. Sonnenberg, D. Williams-Young, F. Ding, F. Lipparini, F. Egidi, J. Goings, B. Peng, A. Petrone, T. Henderson, D. Ranasinghe, V. G. Zakrzewski, J. Gao, N. Rega, G. Zheng, W. Liang, M. Hada, M. Ehara, K. Toyota, R. Fukuda, J. Hasegawa, M. Ishida, T. Nakajima, Y. Honda, O. Kitao, H. Nakai, T. Vreven, K. Throssell, J. J. A. Montgomery, J. E. Peralta, F. Ogliaro, M. J. Bearpark, J. J. Heyd, E. N. Brothers, K. N. Kudin, V. N. Staroverov, T. A. Keith, R. Kobayashi, J. Normand, K. Raghavachari, A. P. Rendell, J. C. Burant, S. S. Iyengar, J. Tomasi, M. Cossi, J. M. Millam, M. Klene, C. Adamo, R. Cammi, J. W. Ochterski, R. L. Martin, K. Morokuma, O. Farkas, J. B. Foresman, D. J. Fox, Gaussian, Inc., 2016.

[11] F. Weigend, R. Ahlrichs, *Phys. Chem. Chem. Phys. (PCCP)* **2005**, *7*, 3297–3305.

[12] M. Bujak, H.-G. Stammler, S. Blomeyer, N. W. Mitzel, *Chem. Commun.* **2019**, *55*, 175–178.

[13] M. T. Kirchner, D. Bläser, R. Boese, *Chem. Eur. J.* **2010**, *16*, 2131–2146.

[14] D. A. Bond, *Chem. Commun.* **2003**, 250–251.

[15] SHELXT, Program for Crystal Structure Solution, G. M. Sheldrick, Universität Göttingen, 2014.

[16] G. M. Sheldrick, *Acta Cryst.* **2008**, *A64*, 112–122.

[17] SHELXL-97, Program for Crystal Structure Refinement, G. M. Sheldrick, 1997.

[18] C. B. Hübschle, G. M. Sheldrick, B. Dittrich, *J. Appl. Crystallogr.* **2011**, *44*, 1281–1284.

[19] Diamond 5.0.0, K. Brandenburg, Crystal Impact GbR, 1997–2023.
